# Interaction of Temperature and Photoperiod Increases Growth and Oil Content in the Marine Microalgae *Dunaliella viridis*

**DOI:** 10.1371/journal.pone.0127562

**Published:** 2015-05-19

**Authors:** Soundarya Srirangan, Marie-Laure Sauer, Brian Howard, Mia Dvora, Jacob Dums, Patrick Backman, Heike Sederoff

**Affiliations:** Department of Plant and Microbial Biology, North Carolina State University, Raleigh, North Carolina, United States of America; Washington State University, UNITED STATES

## Abstract

Eukaryotic marine microalgae like *Dunaliella spp*. have great potential as a feedstock for liquid transportation fuels because they grow fast and can accumulate high levels of triacylgycerides with little need for fresh water or land. Their growth rates vary between species and are dependent on environmental conditions. The cell cycle, starch and triacylglycerol accumulation are controlled by the diurnal light:dark cycle. Storage compounds like starch and triacylglycerol accumulate in the light when CO_2_ fixation rates exceed the need of assimilated carbon and energy for cell maintenance and division during the dark phase. To delineate environmental effects, we analyzed cell division rates, metabolism and transcriptional regulation in *Dunaliella viridis* in response to changes in light duration and growth temperatures. Its rate of cell division was increased under continuous light conditions, while a shift in temperature from 25°C to 35°C did not significantly affect the cell division rate, but increased the triacylglycerol content per cell several-fold under continuous light. The amount of saturated fatty acids in triacylglycerol fraction was more responsive to an increase in temperature than to a change in the light regime. Detailed fatty acid profiles showed that *Dunaliella viridis* incorporated lauric acid (C12:0) into triacylglycerol after 24 hours under continuous light. Transcriptome analysis identified potential regulators involved in the light and temperature-induced lipid accumulation in *Dunaliella viridis*.

## Introduction

Marine microalgae have been investigated for their great potential as feedstocks for renewable liquid transportation fuels because they could replace petroleum-derived fuels without competing for resources like land or fresh water required for food and feed production [1,2]. *Dunaliella spp*. grow in salt lakes and marine environments worldwide and have adapted to their specific environments [3,4]. Several *Dunaliella spp*. grow well under high levels of salt, which reduces culture contamination with other microorganisms or pathogens [5,6]. *Dunaliella spp*. grow in media containing an extremely wide range of NaCl concentrations from 0.05 M to 5.5 M NaCl [7]. The ability of *D*. *salina* to produce high levels of carotenoids made it commercially viable as a nutritional supplement [8]. In contrast to *D*. *salina*, *D*. *viridis* does not accumulate large amounts of β-carotene, but produces oxygenated carotenoids [9]. *Dunaliella spp*. lack a cell wall [4], which enables the extraction of oil droplets by osmotic shock in freshwater as one of the most inexpensive and environmental friendly ways to extract oil from algae cultures [10]. Despite all these useful traits of *Dunaliella* for bioenergy production, production of algae oil for liquid transportation fuels is commercially not viable. Technoeconomic analysis identified algae oil content and growth rates as the two top cost modifiers of fuel production [11]. We therefore focused our research on the regulation of cell division rates and oil content in *Dunaliella* by environmental control.

Photosynthetic organisms accumulate carbohydrates, sugars and/or triacylglycerides (TAGs) during the light period to store assimilated carbon and energy, which is then used for consumption during the night, or when environmental conditions limit their growth [12,13]. Cell division rates and TAG accumulation exhibit an inverse relationship because cell division rates are maximal under optimal growth conditions while TAG as carbon and energy storage component accumulates during conditions that limit cell division [14]. Nitrogen deprivation, salt or light stress and high CO_2_ concentration have been shown to increase oil accumulation in different algal species [15–19]. The response of different *Dunaliella* species or even strains to oil-inducing growth conditions varies greatly. Nitrogen deprivation can induce increases in lipid content in *D*. *viridis* and *D*. *tertiolecta* [15,19] or can have no effect on lipid content of the cells of *D*. *salina* [20].

Because regulation of oil accumulation and cell division are diurnally regulated in algae [21–23], we studied the responses of *Dunaliella spp*. to changes in photoperiod as well as temperature, and the combination of both environmental effects. A shift from light:dark cycles to continuous light should eliminate the need of the cells to degrade their carbon and energy reserves during the dark period.

The aim of this research was to characterize the time-resolved physiological, metabolic and genomic response of *Dunaliella viridis* to photoperiod, growth temperature and the integration of changes in both environmental conditions. Our results show that changes in photoperiod and temperature have both, specific and combined effects on gene expression and metabolism. While cell division is synchronized by the dark period, the cellular protein content only responds to temperature, not light period. Starch metabolism on the other hand is controlled via transcriptional regulation of its degradation enzymes and does not respond to temperature changes under light:dark cycles, but is sensitive to temperature under continuous light conditions. Accumulation of TAG has two distinct and independent components—a photoperiod responsive component that increased oil content more than five-fold, and a temperature-dependent component responsible for a doubling of oil content. These effects were independent, so that the combination of continuous light and elevated temperature led to a tenfold increase in oil content. Transcriptome analysis showed that fatty acid (FA) biosynthesis for increases in oil content under elevated temperature is not transcriptionally regulated, while TAG biosynthesis is driven by transcriptional up-regulation of lipases involved in the recycling of FAs from membrane lipids.

## Materials and Methods

### Strain and growth conditions

The *Dunaliella spp*. strains (*Dunaliella viridis* dumsii, *D*. *salina* LB200, *D*. *tertiolecta* LB999 and *D*. *primolecta* LB1000) used in this study were obtained from the Center for Applied Aquatic Ecology at North Carolina State University. The same growth media was used for all strains and subsequent experiments and was modified from [24] using 1M NaCl with the addition of 10 mM Tris and pH 7.5. For semi-quantitative assessment of oil accumulation (S1 Fig), different species of *Dunaliella* were grown in 1 M NaCl growth media in 250 mL Erlenmeyer flasks for 142 hours under two different light conditions (12 hr light/ 12 hr dark cycle, LD; or continuous light, LL) and temperatures (25°C or 35°C). The cells were grown under cool white fluorescent lights (3340 lux) without shaking or provision of external air or CO_2_. For semi-quantitative assessment of oil accumulation, osmotic lysis was performed by adding 1 mL of fresh water to a pellet containing 100 million cells in a sterile microcentrifuge tube and vortexed thoroughly. Nile red to a final concentration of 2.6 μM was added to the cell debris, vortexed and centrifuged at 13000 rpm for 5 minutes and the Nile red stained oil bodies were visualized under blue light (S1 Fig).

**Fig 1:**
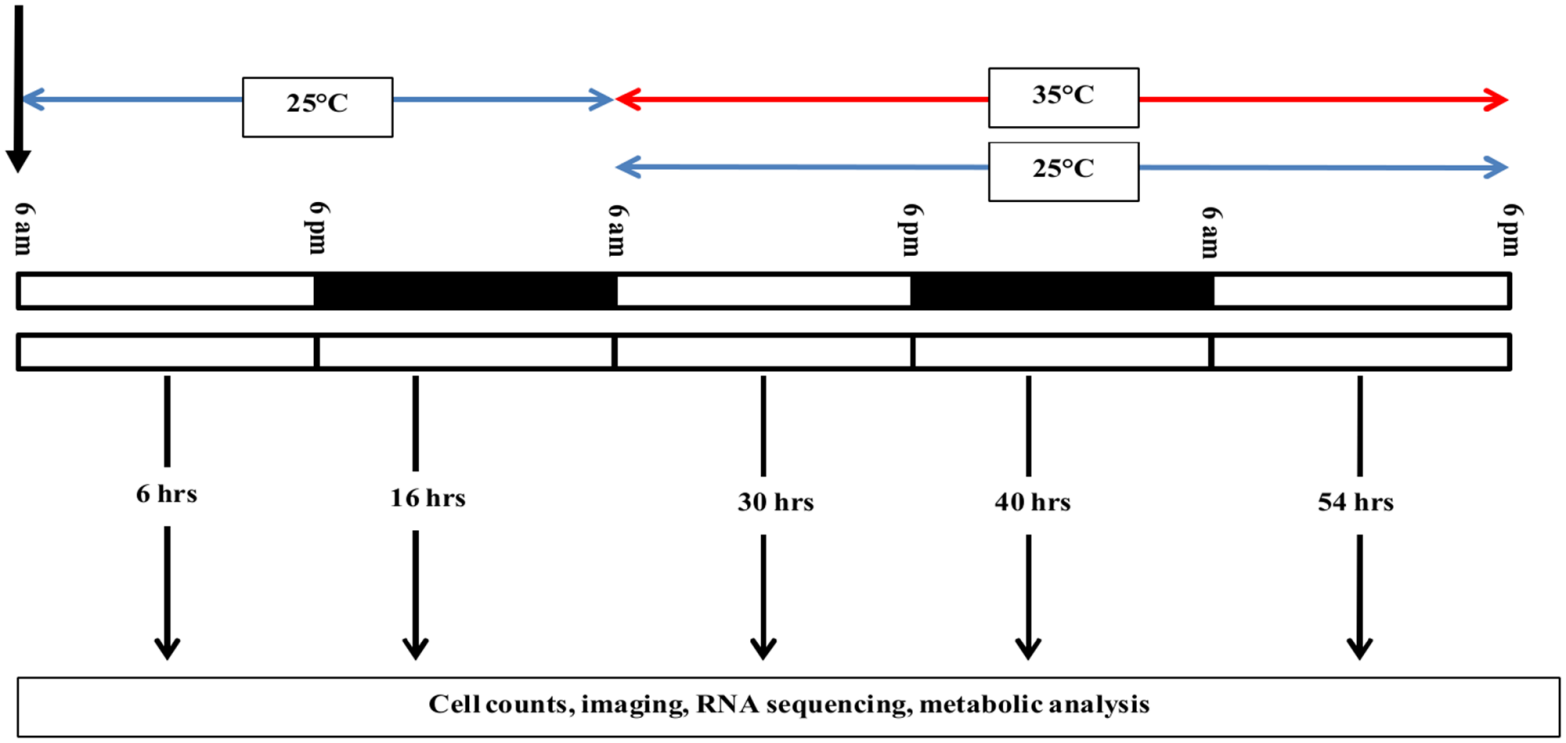
Schematic of experimental design. *D*. *viridis* cultures were grown either under 12 hrs light/ 12 hrs dark cycle (LD) or under continuous light (LL) at 25°C. The temperature was increased after 24 hrs to 35°C or kept at 25°C. The experiment was carried out in 3 biological replicates with three technical replicates for each biological repeat. In this study, biological replicates refer to the independent experiments done at different times and technical replicates refer to the sample size included under each biological repeat. Samples at five time points (6, 16, 30, 40, and 54 hours) were harvested from all the technical replicates of each biological repeat. For measurements of cell density, cell size, imaging, chlorophyll, and total soluble protein, we analyzed all the technical replicates for each biological repeat across all growth conditions at all five time points. The starch, lipids, and RNA-Sequencing were all highly labor intensive and hence we analyzed only one technical replicate for each condition tested out of each biological repeat at all five time points.

A more detailed metabolic and genomic analysis of the responses to environmental changes was carried out for *Dunaliella viridis* dumsii. The experimental design is presented in (Fig 1). The inoculum was grown in Erlenmeyer flasks for 1 week under standard conditions of 12 hr light/ 12 hr dark cycle (cool white fluorescent lights, 3340 lux) at 25°C without shaking or provision of external air or CO_2_. Experimental *D*. *viridis* cultures (0.5 million cells/mL) in Erlenmeyer flasks were subjected to either a light:dark cycle (12 hr light/ 12 hr dark cycle (LD) or continuous light (LL). In addition, the cultures were kept for the entire course of the experiment at 25°C, or the temperature of the incubator was increased from 25°C to 35°C after 24 hrs from the start of the experiment. Flasks were not shaken or provided with external air or CO_2_ during the experiment. At time points (6, 16, 30, 40 and 54 hrs), 10 μL of cell culture was removed to analyze cell density and diameter (TC10 Automatic Cell Counter, Bio-Rad), 20 mL for transcriptome analysis, 1 mL for TAG content examination by Nile Red staining and microscopy (S1 Protocol), 250 μL for total chlorophyll analysis, 2 mL for soluble protein analysis, 400 mL for total lipid and starch analysis.

### Chlorophyll analysis

Total chlorophyll was determined using a modified protocol as described by Arnon [25]. 1 mL of 100% ethanol was added to 250 μL of algae culture, vortexed, and incubated at room temperature for 10 minutes. Samples were vortexed again and pelleted (13000 rpm, 10 min) and the supernatant containing the extracted chlorophyll was transferred to a fresh microcentrifuge tube. The chlorophyll content was measured as absorption at 652 nm against the growth media undergoing the same extraction steps as that of algae culture. The total chlorophyll amount (μg/mL) was calculated as A_652_/36 (36 = extinction coefficient). Total chlorophyll was measured from three biological replicates with three technical replicates each.

### Protein analysis

Total soluble protein was extracted using a modified protocol as described by Barbarino and Lourenco [26]. 2 mL of culture was pelleted (13000 rpm, 5 min), the supernatant removed and the pellet frozen in liquid nitrogen and stored at -80°C. 1 mL of deionized water was added to the frozen pellet, centrifuged (13000 rpm, 5 min, 4°C), and the supernatant, containing crude protein extract, was collected. The pellet was re-extracted with 1 mL of 0.1 N NaOH with 0.5% β-mercaptoethanol (v/v), incubated for 1 hour at room temperature with intermittent vortexing, and centrifuged (13000 rpm, 5 min, 4°C). The supernatants were pooled and stored at 4°C. Protein concentration was determined using the microplate procedure of the bicinchoninic acid (BCA) protein assay kit (Pierce Thermo Scientific, Catalog # 23235) following the manufacturer’s instructions. Briefly, about 150 μL of the reagent was mixed with 150 μL of the extracted protein sample and incubated at 37°C for 2 hours followed by measurement of absorbance at 562 nm on a plate reader (Synergy HT multi-mode). The concentration of the extracted protein was determined against the standard curve generated with bovine serum albumin (BSA) stock provided in the kit using 1X phosphate buffered saline (8 mM Na_2_HPO_4_, 2 mM KH_2_PO_4_, 137 mM NaCl, 2.7 mM KCl, pH 7.4). Total soluble protein was measured from three biological replicates with three technical replicates each.

### Lipid analysis

#### Total lipid extraction

Algae pellets (4000 rpm, 5 min) from 400 mL of culture were frozen in liquid nitrogen and placed at -80°C. Total lipids were extracted using a modified protocol as described by Bligh and Dyer [27]. Frozen pellet was resuspended in 1 mL of water followed by extraction with 3.75 mL of methanol:chloroform (2:1, v/v) and vortexed intermittently over the course of 75 min. The suspension was pelleted (2000 rpm, 5 min), the supernatant was collected and the pellet resuspended in 1 mL of 1M NaCl and re-extracted with methanol:chloroform. The suspension was vortexed, pelleted, and the supernatants pooled. 2.5 mL of chloroform and 2.5 mL of water were added, vortexed, and centrifuged (2000 rpm, 5 min). The chloroform phase was collected, dried under vacuum and then nitrogen, and stored at -20°C for lipid analysis. The pellet was stored at -20°C for starch analysis. Total lipid content was measured from three independent biological replicates.

#### Thin layer chromatography (TLC)

The TLC protocol used for the separation of lipids was done using a modified protocol as described by Moellering *et al*. [28]. The total lipids were resuspended in chloroform:methanol (2:1, v/v) to a final concentration of 10 mg/mL for lipid fractionation by TLC. Samples (1.5 mg per lane) were spotted and run along with a lane of standard mix (Diacylglycerol, tocopherol, linoleic acid and glyceryl trioleate, 1.6 mg/mL each, prepared fresh, 30 μL per lane) on silica gel adsorption plates (Whatman K5 150 Å) using petroleum ether:diethyl ether:glacial acetic acid (80:20:1) as the mobile phase. Plates were dried and then developed using iodine crystals. Fractions for free fatty acids (FFA), triacylglycerol (TAG), and diacylglycerol (DAG) were scraped off the plates and placed into separate tubes.

#### Fatty acid methyl esters (FAMES) preparation

FAMES were prepared from the different TLC lipid fractions using a modified protocol as described by Laurens *et al*. [29]. 50 μg of tridecanoic acid (Sigma) was added as standard to each fraction. Transesterification was carried out by adding 0.2 mL of chloroform:methanol (2:1, v/v) and 0.3 mL of HCl/Methanol (5%, v/v) to each fraction, followed by incubation for 1 hour at 85°C. 1ml of hexane was added to the samples and incubated at room temperature for 1 hour. The hexane phase was collected, dried under vacuum and then nitrogen, and stored at -20°C.

#### GC/MS analysis

The FAMES were resuspended in 200 μl of hexane, and 1 μL of sample was injected (splitless mode) in a Hewlett Packard 5890 series II plus GC/MS (Hewlett Packard 5972 series mass selective detector, Hewlett Packard 7673 injector and Rtx-5 GC column). The Agilent Enhanced ChemStation G1701BA Version B.01.00 software for HP5890GC was used for spectral analysis (Wiley7Nist05.L library).

#### Polar lipid analysis

Polar lipid analysis was performed at the Kansas Lipidomics Research Center (KLRC). Total lipids were extracted from the 54 hours time point from three independent biological replicates under each condition, dried and sent for plant polar lipid analysis. The polar lipids species analyzed at KLRC were PG (phosphatidylglycerol), PA (phosphatidic acid), PE (phosphatidylethanolamine), PI (phosphatidylinositol), PS (phosphatidylserine), PC (phosphatidylcholine), Lyso-PE (lysophosphatidylethanolamine), Lyso-PC (lysophosphatidylcholine), Lyso-PG (lysophosphatidylglycerol), MGDG (monogalactosyldiacyl-glycerol), and DGDG (digalactosyldiacylglycerol). This protocol does not include diacylglycerol-N-trimethylhomoserine (DGTS) analysis.

### Starch analysis

Starch was extracted from the pellet left over from the lipid extraction using a modified protocol as described by Winter [30]. The pellet was resuspended in 2.8 mL of cold extraction buffer (3.5 mL methanol, 1.5 mL chloroform and 0.6 mL phosphate buffer (20 mM KH_2_PO_4_, pH 7.0), 5 mM EGTA, 20 mM NaF), and thoroughly vortexed. After centrifugation (4000 rpm, 5 min), the organic phase was removed and the pellet was re-extracted with 2.8 mL of cold extraction buffer. The pellet was dried, solubilized in 2 mL of 0.2 N KOH, and placed in a shaking water bath at 80°C for 4 hrs. The sample pH was adjusted to 4.5–5.0 with glacial acetic acid at room temperature, and placed in a sonicator bath for 10 min. 100 μL aliquots from the sonicated samples were mixed with 400 μL of starch digestion buffer (50 mM sodium acetate, pH 4.8, 33 nkat amylase, 33 nkat amyloglucosidase), incubated at 37°C for 16 hours for hydrolysis of starch to glucose. Samples were centrifuged (13000 rpm, 5 min) and the collected supernatant was used for the glucose assay. 50 μL of supernatant mixed with 950 μL of reaction buffer (50 mM imidazole, 1.5 mM MgCl_2_ anhydrous, pH 6.9), 100 mM nicotinamide adenine dinucleotide phosphate (NADP), 200 mM adenosine triphosphate (ATP) and 50 nkat of glucose-6-phosphate dehydrogenase. Absorbance changes at 334 nm were determined before and after the addition of 50 nkat of hexokinase. The change in absorbance due to the reduction of NADP to NADPH was recorded and compared to a starch standard curve. Starch content was measured from three independent biological replicates.

### RNA extraction

20 mL of cells were centrifuged (4000 rpm, 5 min) and RNA was extracted from the pellet using 1 mL of TRIzol reagent (Invitrogen, Carlsbad, CA) according to the manufacturer’s instructions. The quality and concentration of the RNA was assessed using an Agilent Bioanalyzer on an RNA Pico Chip and used for preparation of cDNA libraries for RNA-Sequencing and analysis (S2 Protocol). In order to confirm the results from the RNA-sequencing data, expression of specific genes were tested by qPCR (S2 Protocol) using gene-specific primers (S9 Table).

## Results

Several *Dunaliella spp*. (*D*. *viridis*, *D*. *salina* LB200, *D*. *tertiolecta* LB999, *D*. *primolecta* LB1000) were screened for their growth and accumulation of oil in response to temperature and light changes (S1 Fig). The four different *Dunaliella* species showed different responses to those changes in light duration or temperature (S1 Fig). The screen showed that on a cellular basis, only *D*. *salina* and *D*. *viridis* produced significant amounts of oil as indicated by Nile red fluorescence under elevated temperature. *D*. *tertiolecta* had a slight amount of oil produced at 25°C under continuous light, but not at 35°C. *D*. *primolecta* showed no detectable oil accumulation under either condition. *D*. *salina* produced more biomass at 25°C compared to 35°C, while all other species showed the same or slightly more biomass production at elevated temperature (S1 Fig). This shows that the responses to environmental factors in *Dunaliella* are genotype specific. For a detailed metabolic and genomic analysis of the regulation underlying this response, we selected *Dunaliella viridis* dumsii because it showed the highest oil increase under induction when grown under LL at elevated temperature in comparison to other strains. We designed a time-course experiment to analyze growth, metabolite contents and transcriptome changes to characterize the stress specific responses as well as responses to a combined stress in *Dunaliella viridis* dumsii (Fig 1).

### Cell division and growth

**Fig 2:**
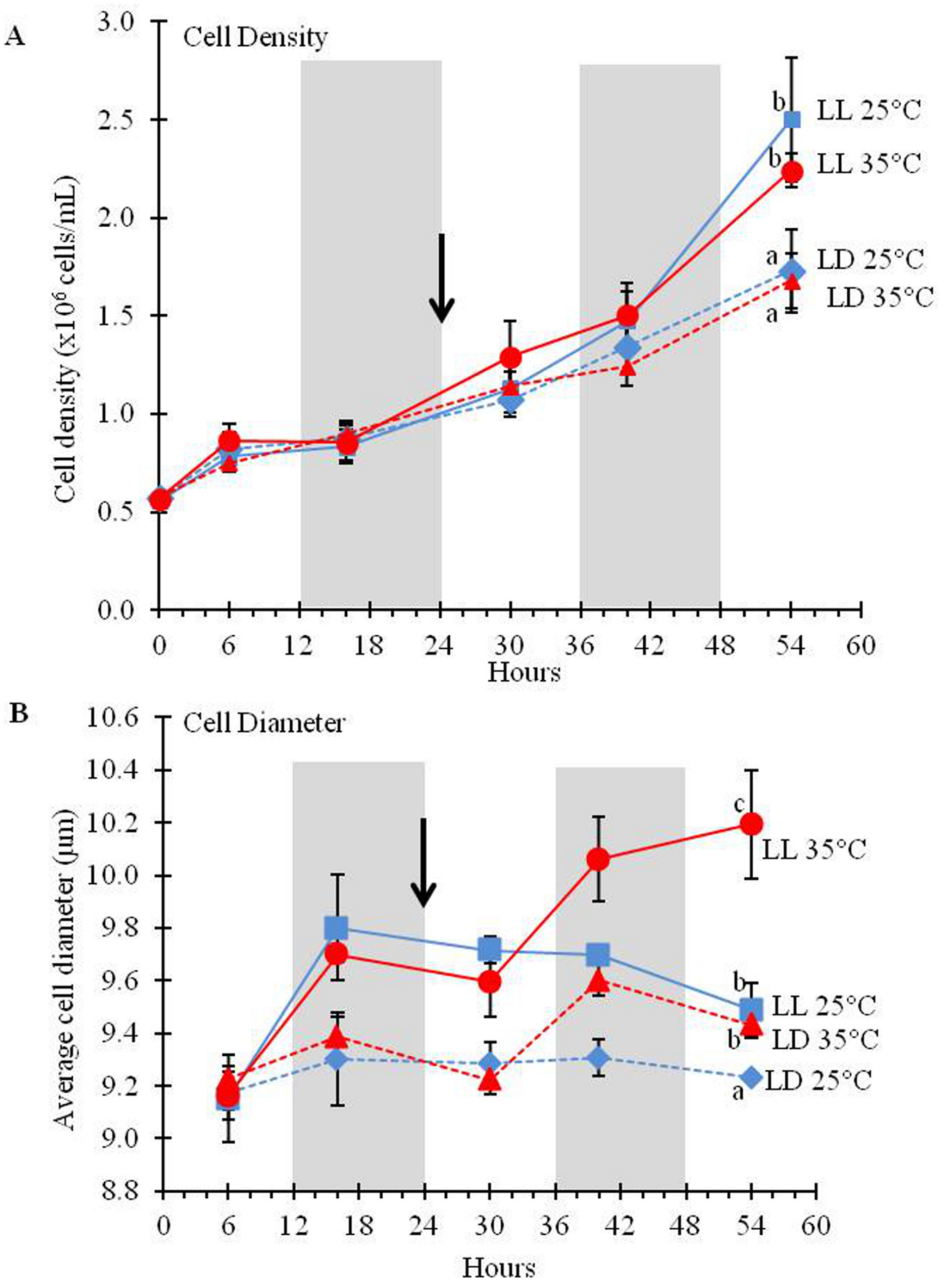
Cell division rate and cell size increases under LL versus LD cycles. *D*. *viridis* cultures were grown either under LD (dashed lines) or LL (solid lines) at 25°C for the first 24 hours and then either remained at 25°C (blue lines) or the temperature was raised to 35°C (red lines). The temperature shift occurred after 24hrs (black arrow). Cell counts (A) and maximal cell diameters (B) were measured in three biological replicates with three technical replicates each. The error bars represent the standard deviation. Statistical significance was assessed by unpaired, two-tailed, Student’s *t*-test. The values with the same letters are not significantly different at 54 hrs (p<0.05).

To assess the effect of light duration and temperature on cell growth we quantified cell numbers and cell diameters at each time point. Cells grown under LL divided faster than those grown under LD (Fig 2A). The number of cells tripled under LD and increased 4–5 times under LL, but the difference in cell density was significant only after 54 hours. A separate experiment with higher temporal resolution under the same conditions, showed that cell division occurs during the dark period under LD regime while cells grown under LL showed a constant growth rate (S2 Fig). Higher temperature under LL did not have an apparent effect on cell division rates during the time of our experiment (Fig 2A); however, it had a significant effect on cell diameters (Fig 2B). The estimated volume of the cells, assuming that all cells have the shape of prolate spheroids, cells grown under LL at 35°C was 135 fl compared to an average volume of 112 fl of cells grown at LL 25°C after 54 hrs; a cell volume increase of ca. 21% (Fig 2B). Micrographs of representative cells from the cultures are shown in (S3 Fig).

### Cellular chlorophyll and protein content is affected by temperature

**Fig 3:**
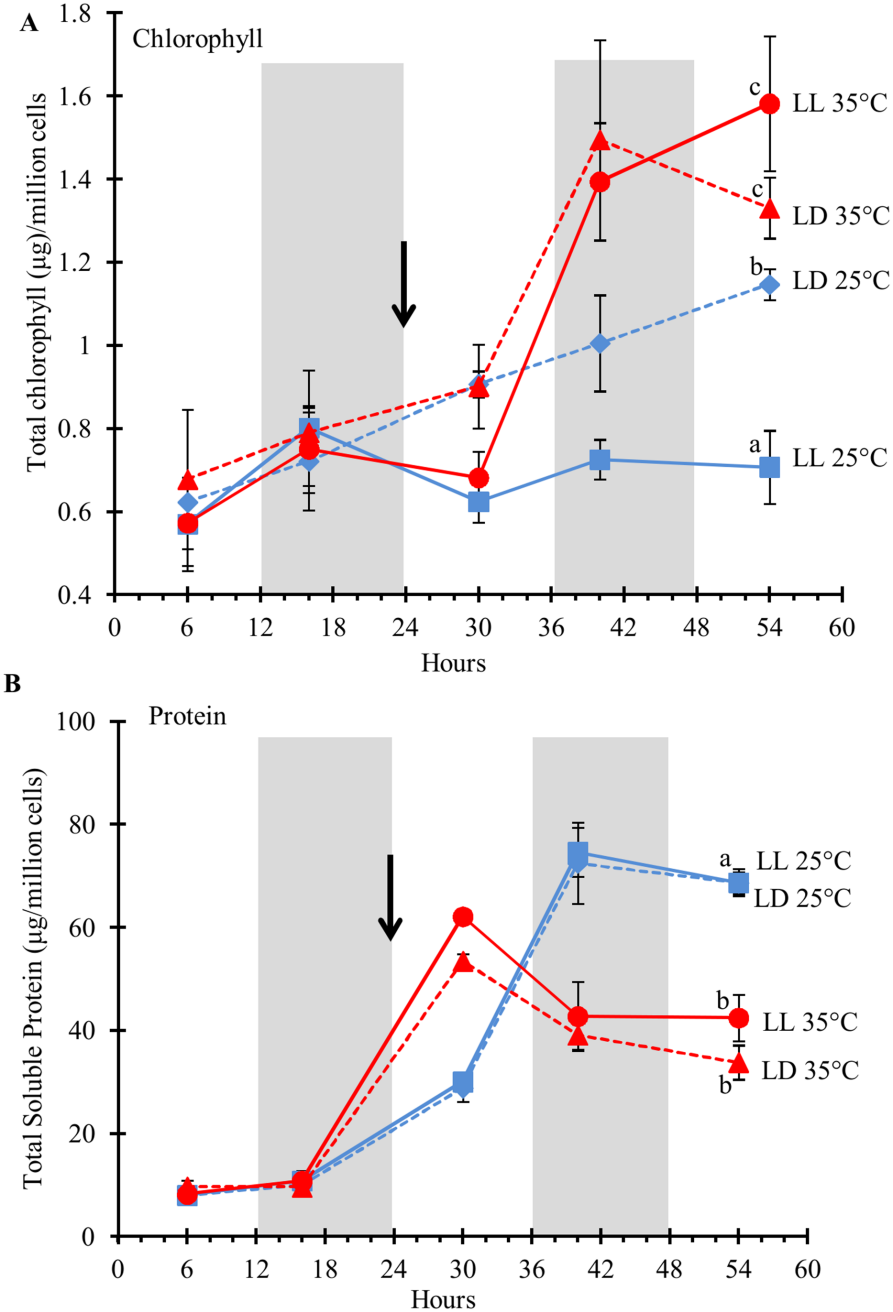
Total chlorophyll (A) and soluble protein (B) content in *D*. *viridis* in response to light duration and temperature. While chlorophyll content increases in response to elevated temperature, soluble protein content in those cells decreases. *D*. *viridis* cultures were grown either under LD (dashed lines) or LL (solid lines). All cultures were grown at 25°C for the first 24 hours and then either remained at 25°C (blue lines) or the temperature in the growth chamber was raised to 35°C (red lines). Temperature shift is indicated by the black arrow. Total chlorophyll content, soluble protein and cell counts were measured in three biological replicates with three technical replicates each. The data were normalized to 1 million cells. The error bars represent the standard deviation. Statistical significance was assessed by unpaired, two-tailed, Student’s *t*-test. The values with the same letters are not significantly different at 54 hrs (p<0.05).

Higher temperature had opposite effects on chlorophyll (Fig 3A) and soluble protein (Fig 3B) contents: 30 hrs after the temperature shift (54hrs), cells grown at 35°C had more chlorophyll and less soluble protein compared to those grown at 25°C. The difference in the chlorophyll content was the most striking and happened very rapidly after the increase in temperature. At 54 hrs under LL, cells that were transitioned to 35°C had more than twice the amount of chlorophyll compared to cells grown at 25°C. Interestingly, cells grown at 25°C had significantly more chlorophyll when grown under LD than LL. The photoperiod did not have any effect on soluble protein content in cells grown at 25°C, and only a minor, but significant effect in cells grown at 35°C.

### Starch is more affected by light duration than temperature

**Fig 4:**
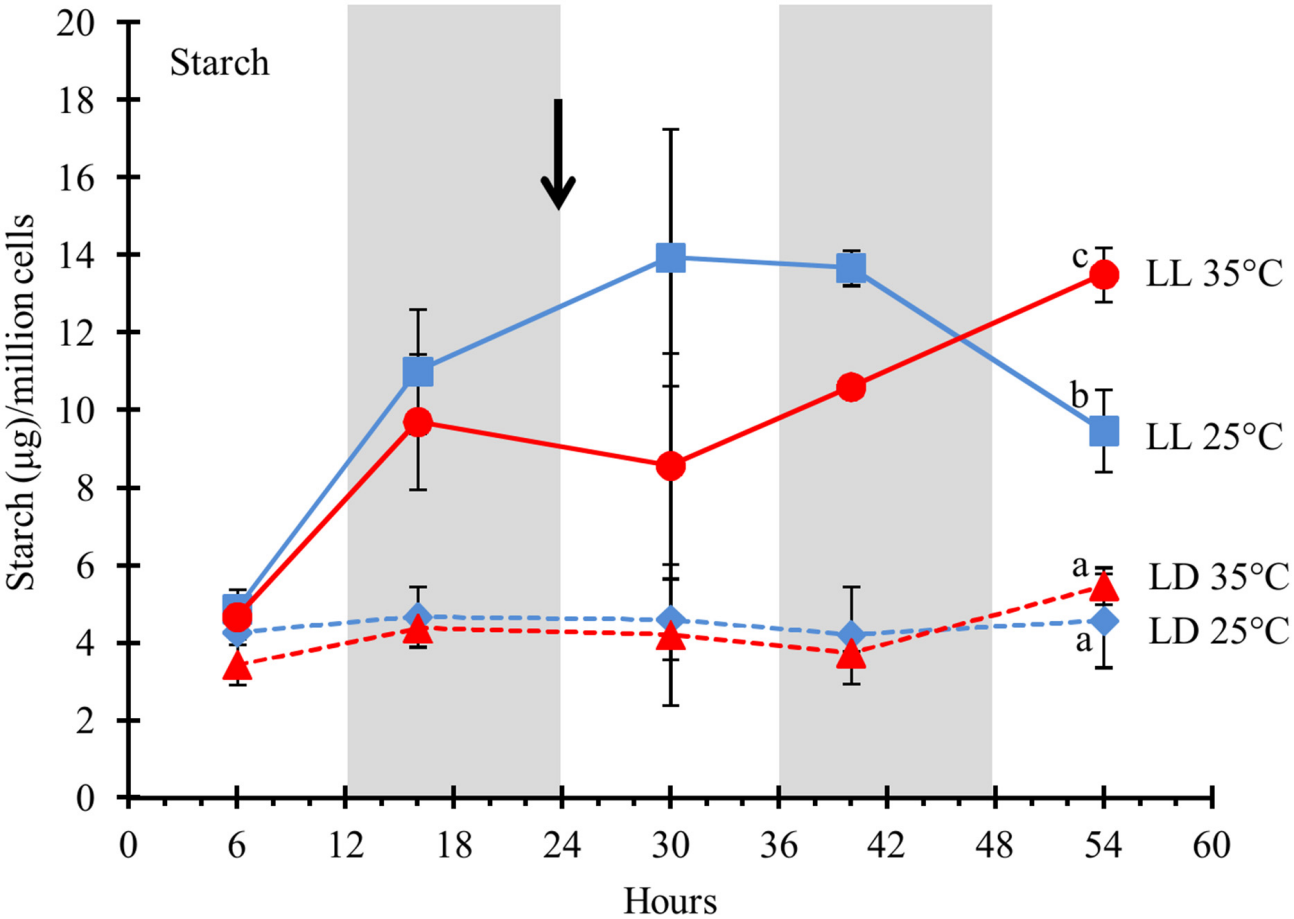
Total starch in *D*. *viridis* cells in response to light duration and temperature. *D*. *viridis* cultures were grown either under LD (dashed lines) or LL (solid lines). All cultures were grown at 25°C for the first 24 hours and then either remained at 25°C (blue lines) or the temperature in the growth chamber was raised to 35°C (red lines). The temperature shift is indicated by the black arrow. The data were normalized to 1 million cells. The error bars represent the standard deviation from three independent biological replicates. Statistical significance was assessed by unpaired, two-tailed, Student’s *t*-test. The values with the same letters are not significantly different at 54 hrs (p<0.05).

Starch, like TAGs serve as storage components for assimilated carbon and energy. In agreement with this function, starch content on a per cell basis was significantly higher under LL (Fig 4). After 54 hours, the starch content in the cells grown under LL was at least twice the amount measured in cells grown under LD at either temperature. There was no effect of temperature on the starch content in cells grown under LD. However, cells grown under LL showed some variation in starch accumulation in response to temperature. Cells grown at 25°C reached a maximum of accumulated starch between 24–48 hours, after which starch content decreased, while cells transferred to 35°C accumulated starch and contained about 40% more starch after 54hrs when grown at 35°C compared to 25°C under LL (Fig 4).

### Triacylglycerides content and its fatty acid composition respond to light duration and temperature

Total lipids were extracted using chloroform:methanol solvent and fractionated by TLC (S4 Fig). Three fractions corresponding to DAGs, FFAs, and TAGs were separated, collected, and fatty acids of each fraction derivatized to FAMES and analyzed by GC/MS.

**Fig 5:**
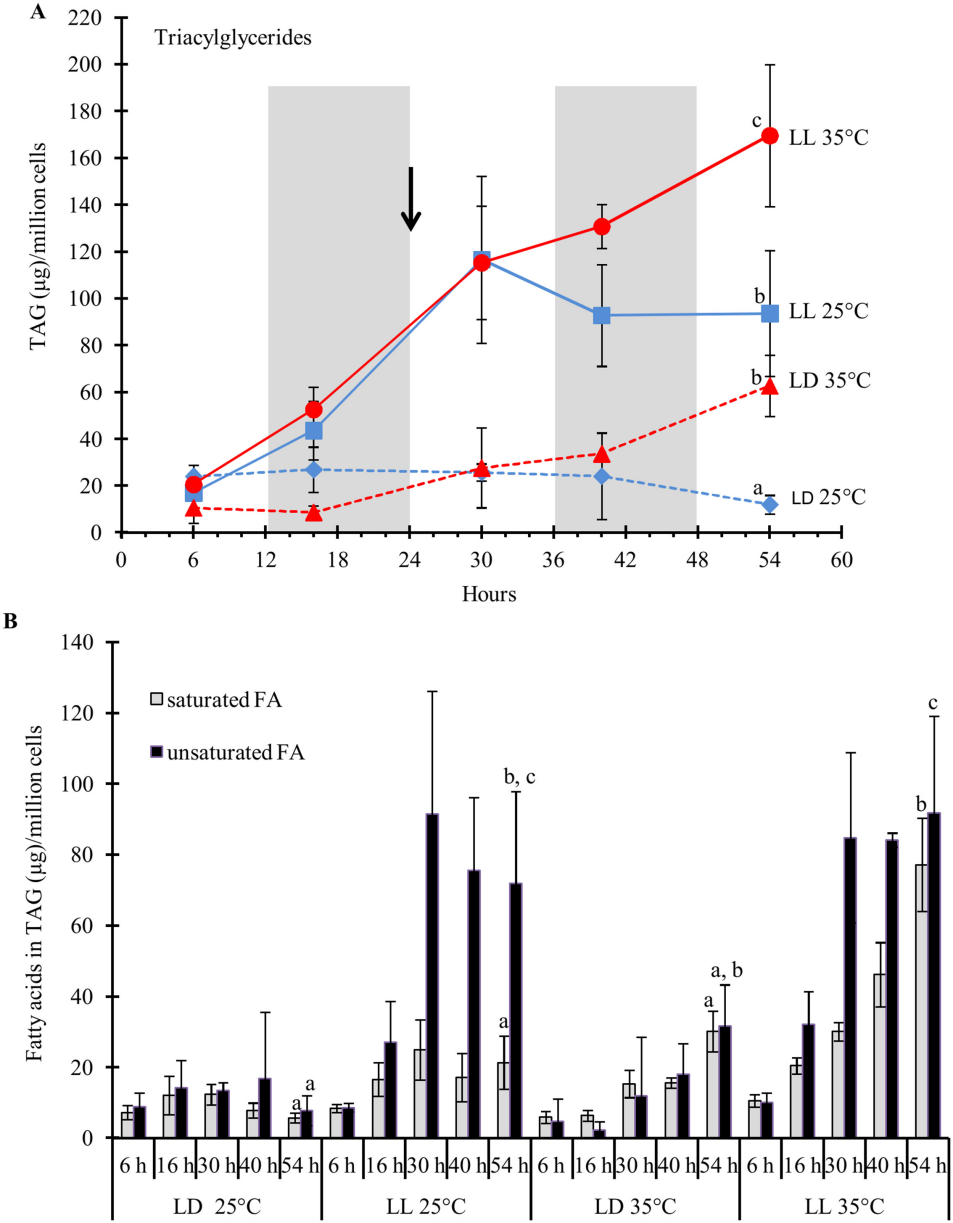
TAG content increases under LL (A) and saturated FAs accumulate at 35°C in TAG (B). *D*. *viridis* cultures were grown either under LD (dashed lines) or LL (solid lines). All cultures were grown at 25°C for the first 24 hours and then either remained at 25°C (blue lines) or the temperature in the growth chamber was raised to 35°C (red lines). The temperature shift is indicated by the black arrow. Total TAG content was calculated as the sum of the fatty acids from the TAG fraction. Total TAG content was normalized to 1 million cells. The error bars represent the standard deviation from three independent biological replicates. Statistical significance was assessed by unpaired, two-tailed, Student’s *t*-test. The values with the same letters are not significantly different at 54 hrs (p<0.05).

The interaction of light duration and temperature had dramatic effects on TAG accumulation in cells as well as on the FA composition of those TAGs. A shift from LD to LL increased total TAG content per cell almost four-fold within the first 30 hrs at 25°C (Fig 5A). When cells were kept at 25°C, there was no further increase in TAG content detected, whereas cells transferred to 35°C accumulated more TAG under both light conditions. The temperature-induced TAG accumulation doubled TAG content under LL within 24 hrs and tripled it under LD (Fig 5A). This indicates that the mechanisms that led to TAG accumulation under continuous light and elevated temperature are independent but additive. The effects of the two factors (light, temperature) on TAG accumulation correlate with differences in their FA profiles. The temperature-induced increases in TAGs correlated with increases in saturated FA, while the change in photoperiod to LL resulted in TAGs with more highly unsaturated FA (Fig 5B). As seen for total TAG content, the increases in saturated FA in response to light duration and temperature increase are additive effects.

**Fig 6:**
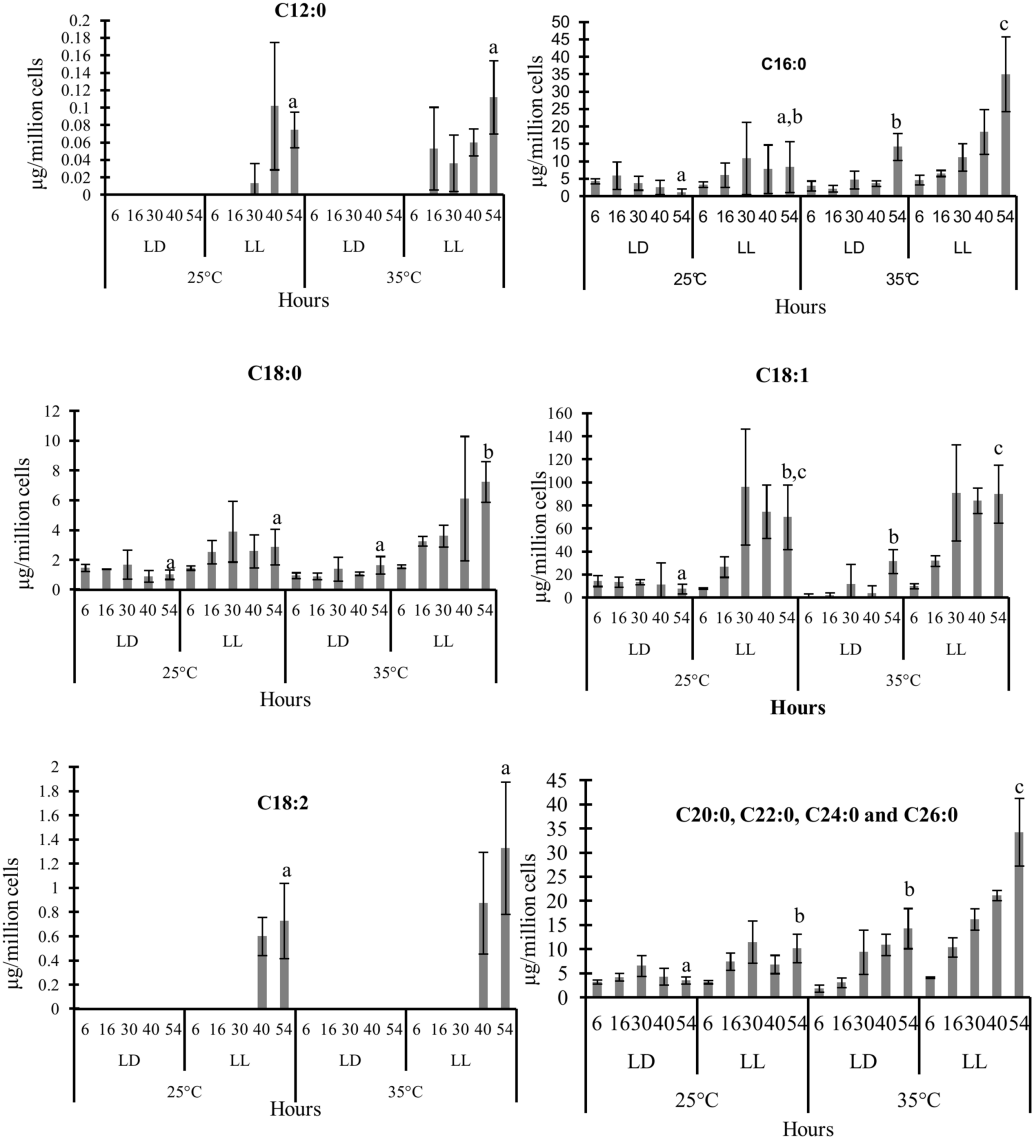
Changes in fatty acid composition of the TAG fraction. The error bars represent the standard deviation from three independent biological replicates. Statistical significance was assessed by unpaired, two-tailed, Student’s *t*-test. The values with the same letters are not significantly different at 54 hrs (p<0.05).

The majority of FAs in the TAG fraction were C18:1 (oleic acid), followed by C16:0 (palmitic acid), C18:0 (stearic acid) and long-chain saturated FAs (C20:0). The TAG accumulation observed in cells grown under LL was mainly due to an increase in C18:1 (Fig 6). After 30 hrs in LL at 25°C, the C18:1 content in TAGs was seven times higher compared to cells grown under LD. Lauric acid (C12:0) and Linoleic acid (C18:2) were only detectable in small quantities and only under LL. Changes in the TAG fatty acid profile in response to the temperature increase from 25°C to 35°C were mostly brought about by increases in the amounts of the saturated FA C16:0, C18:0 and the long-chain saturated FAs C20:0, C22:0, C24:0 and C26:0 (Fig 6).

### Membrane lipids

**Fig 7:**
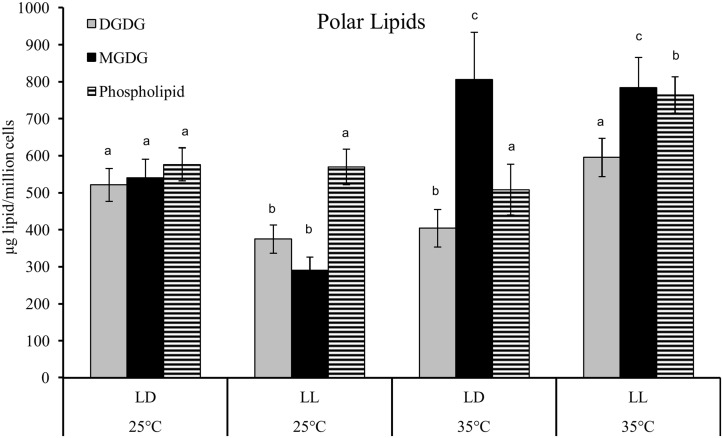
Major polar lipid classes responds to photoperiod and temperature changes. The cellular content of the major polar lipid classes DGDG (grey bars), MGDG (black bars) and PL (striped bars) responded to changes in temperature and light duration. The error bars represent the standard deviation from three independent biological replicates. Statistical significance was assessed by unpaired, two-tailed, Student’s *t*-test. The values with the same letters are not significantly different at 54 hrs (p<0.05).

Membrane lipids are composed of polar lipids that are distinguished by their head group. For a more detailed analysis of specific membrane lipid classes, we analyzed total lipid samples from the endpoint of the time course (54 hrs). The largest fractions of polar lipids are MGDG and DGDG that are primarily found in the chloroplast and thylakoid membranes, and phospholipids (PL) found in all membranes (S1 Table). At 25°C, cells grown under LL had lower amounts of the chloroplast membrane lipids, MGDG and DGDG, while no significant changes were detected in the cellular levels of the other PL classes (PG, PC, PE and PI) (Fig 7). However, at 35°C the same transition from LD to LL significantly increased the amount of DGDG and PL but had no effect on MGDG. The cellular quantities of MGDG increased significantly with increased temperature. PL content was higher only at 35°C under LL, but did not change under any of the other growth conditions (Fig 7). This increase was due to an increase in all the major analyzed PL classes—PG, PC, PE and PI (S5A Fig). PA was found in small quantities (S1 Table) and PS was not detected under any conditions. The majority of DGDG and MGDG showed a total FA chain length of C34 with a total of 3 to 7 double bonds (S6 Fig). This corresponds to a lipid composed of one C18 and one C16 FA. The degree of saturation was higher in MGDGs compared to DGDGs. The temperature induced increase in MGDGs corresponds to decrease in its degree of FA saturation. The same trend can be observed in the DGDGs where at 35°C, the increase in total DGDG is mostly due to an increase in (34:3) and lower levels of (34:6), resulting in an overall lower degree of saturation at 35°C (S6 Fig).

### Lyso-phospholipids and free fatty acids

The cellular content of Lyso-phospholipids was strictly temperature dependent and showed no significant changes in response to light duration. Steady-state levels of Lyso-PG, Lyso-PC and Lyso-PE were 40%-50% lower at 35°C than at 25°C (S5B Fig). FFA can originate from hydrolysis of TAGs or polar membrane lipids as well as during synthesis. During the time-course of the experiment the FFA pool was mostly affected by the light interaction with temperature. The accumulation of FFA over time was different under the two light conditions at 25°C. At 25°C under LD, the FFA was highest at 40 hrs however, under LL, it was highest at 54 hrs (S7A Fig). At 54 hours, cells grown under LL contained higher levels of FFA than cells grown under LD conditions. Under LL, higher temperature caused a 50% increase in FFAs, while no significant increase in response to temperature was observed under LD. Under all conditions, the FFA fraction was mainly (80–90%) composed of unsaturated FAs (S7B Fig).

### Transcriptome analysis

#### Differentially expressed transcripts in response to photoperiod and temperature changes

**Fig 8:**
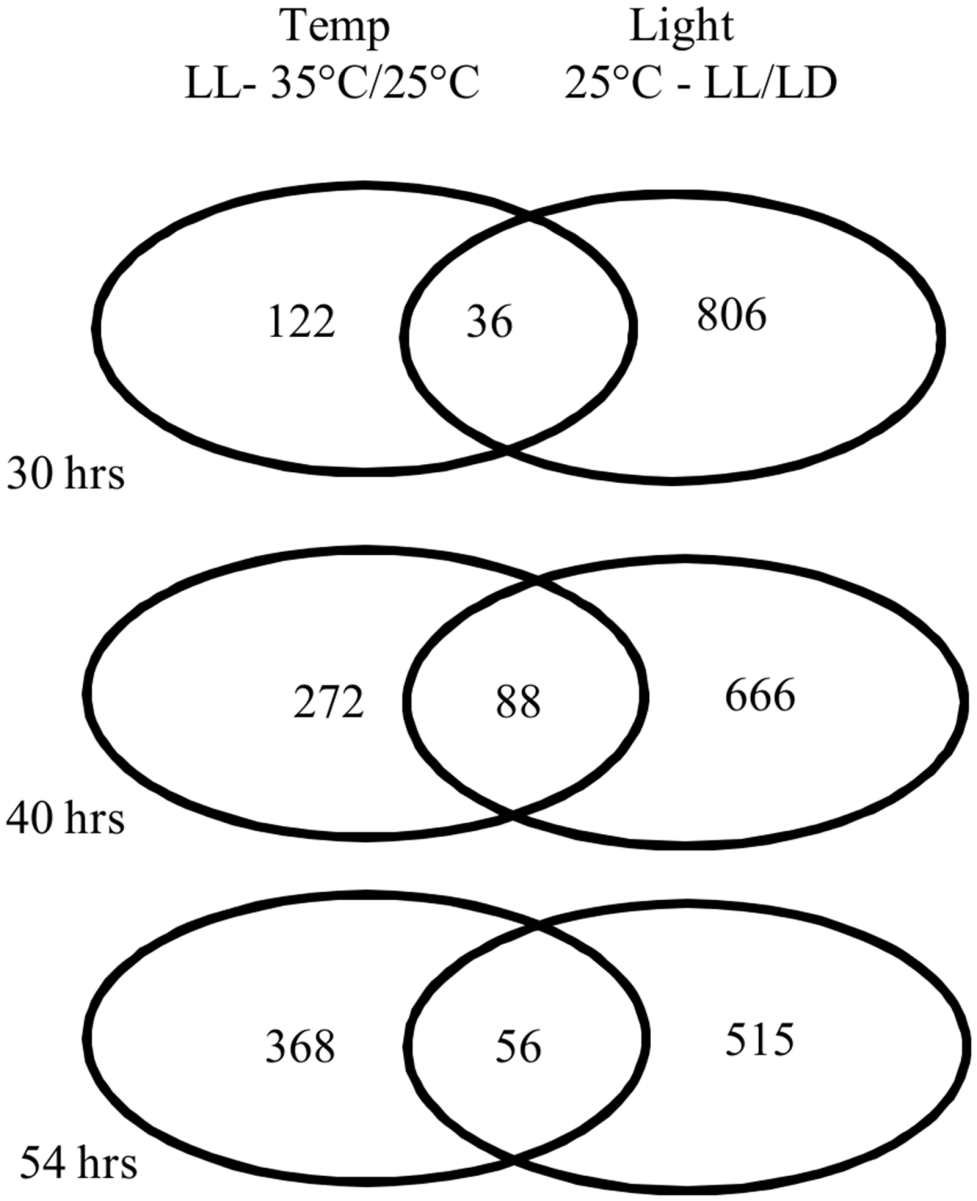
Numbers of transcripts responding to changes in light duration and/or temperature at 30, 40 and 54 hours. The diagrams represent the number of transcripts differentially expressed either only in response to continuous light compared to light:dark cycles at 25°C (25°C-LL/LD) or only in response to increased temperature under continuous light (LL-35°C/25°C).

We carried out RNAseq analysis for each time point and condition to identify changes in transcript abundances that correlate with the observed changes in physiology and metabolism (S2 Protocol). A data summary of transcripts obtained after analysis are available (S2 Table). Our sequence analysis annotated 11,701 transcripts from 9400 distinct genetic loci. All transcript count data are available at NCBI-GEO (GSE40997) and the list of differentially expressed transcripts under light and/or temperature effect are provided (S3–S6 Tables). While those sequence count data can be analyzed in different ways, we compared relative transcript abundances between the time points for photoperiod (LL over LD at 25°C) and temperature treatment (35°C over 25°C under LL) to identify transcripts responding to either stress or both stresses (Fig 8). Out of the 9400 expressed transcripts (S11 Table), 1030 transcripts responded to light duration (S4 Table) and 373 transcripts to higher temperature (S5 Table). Overall, more transcripts showed differential responses to changes in the light regime compared to those responding to changes in temperature. Transcriptional responses were apparent within 6 hours after the temperature increase (Fig 8). The longer the cells were subjected to high temperature, the higher was the number of transcripts differentially expressed in response to this factor, while the number of differentially expressed transcripts in response to LL decreased over time (Fig 8).

As expected, the most highly up-regulated transcripts under elevated temperature in LL were heat shock proteins (HSP20). Fructose-bisphosphate aldolase, cytochrome c oxidase subunit 2 and chloroplast carotene biosynthesis related protein, showed at least 6-fold higher Reads Per Kilobase per Million mapped reads (RPKM) at 35°C than 25°C under LL (S5 Table). Although our time-course was not designed to describe diurnal transcript changes, a large number of differentially expressed transcripts involved in photosynthesis and most primary metabolic pathways followed a strong diurnal pattern under LD conditions that was lost under LL (S11A–S11P Fig).

Gene Ontology (GO) term enrichment analysis (S2 Protocol) showed that the most enriched functional categories with changes in response to light duration (LL/LD) were transcripts involved in CO_2_ assimilation, cell cycle progression, photosynthesis and chloroplast function. At elevated temperature (35°C/25°C) the GO categories with over-representation of differentially expressed transcripts were chloroplast function, energy metabolism, carbohydrate metabolism, photosynthesis, respiratory chain, protein degradation, ion transport, membrane function, and stress response (Table 1).

**Table 1:** Categories enriched in differentially expressed transcripts under LL or high temperature.

	Light			Temperature		
Category	Expected	Count	Over-represented	Expected	Count	Over-represented
Chloroplast	40	162	4	3	9	3
Energy	2	6	3	1	5	5
Carbohydrate metabolism	12	39	3.3	0	2	∞
Photosynthesis	11	49	4.5	2	9	3.5
Respiratory chain	12	45	3.8	0	6	∞
Protein metabolism	13	18	1.4	3	10	3.3
Cell cycle	7	37	5.3			
CO_2_ assimilation	0	2	∞			
Movement	28	61	2.2			
Nucleic acid	321	452	1.4			
Nucleus	1	4	4			
Envelope				1	4	4
Ion transport				2	12	6
Membrane				5	11	2.2
Others	406	626	1.5	2	13	6.5

For each one of the two factors, the categories enriched are presented. The expected column presents the number of transcripts that were expected in a specific category based on the transcripts per GO and the number of differentially expressed transcripts. The count column presents the actual number of transcripts differentially expressed in that category. The third column shows the overrepresentation of a category calculated as the ratio of count/expected hits.

### Transcripts levels from the fatty acid biosynthesis pathway genes under elevated temperature

**Fig 9:**
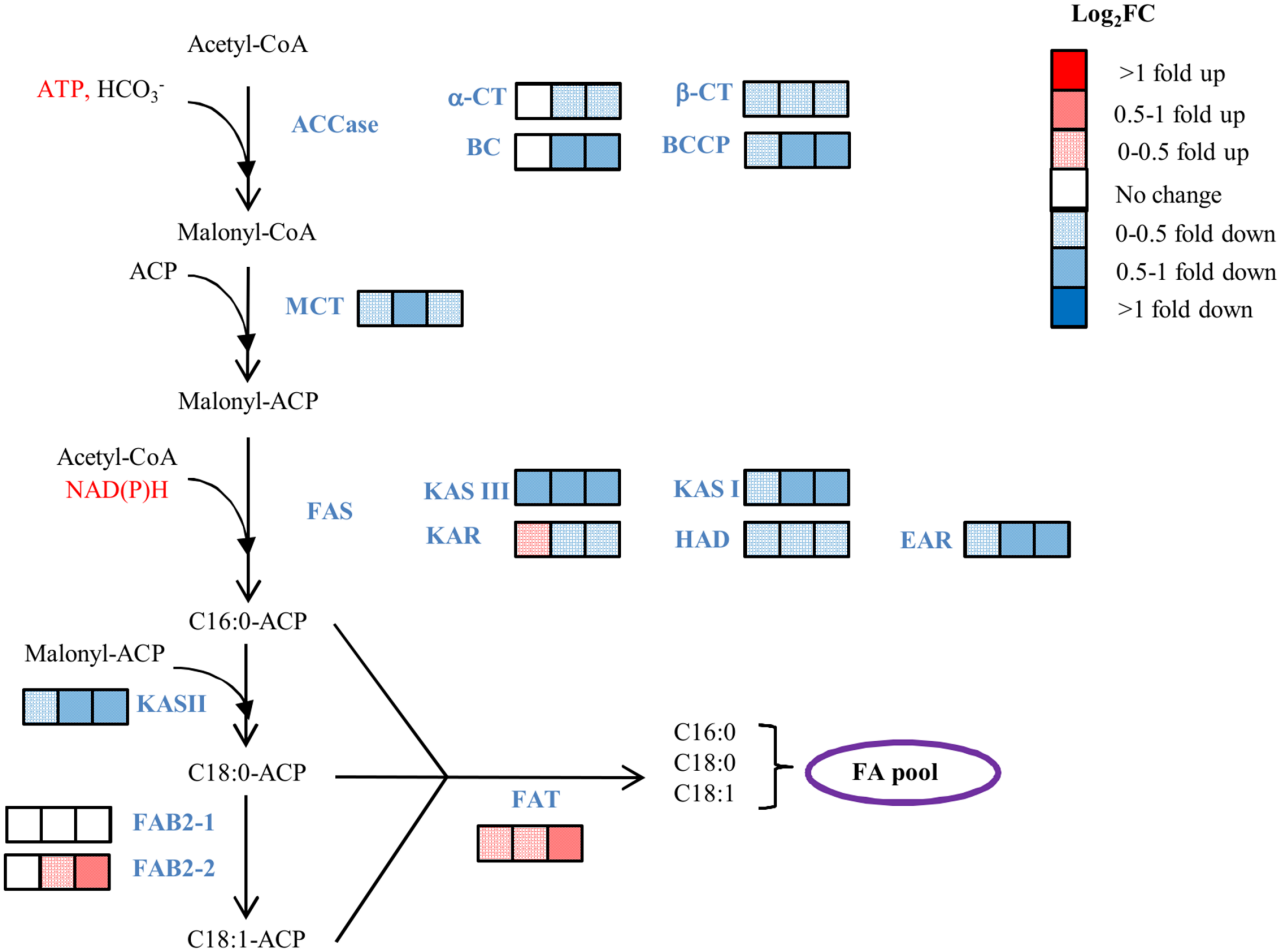
Effect of temperature on the transcript abundance of the fatty acid pathway genes. The blocks of three squares represent the log_2_FC (35°C/25°C) at 30, 40 and 54 hrs (6, 16 and 30 hrs after the temperature increase). CoA: coenzyme A; ACP, acyl carrier protein. Enzymes include: ACCase, acetyl-CoA carboxylase; α-CT, alpha-carboxyltransferase subunit; β-CT, beta-carboxyltransferase subunit; BC, biotin carboxylase subunit; BCCP, biotin carboxyl carrier protein subunit; MCT, malonyl-CoA ACP transacylase; FAS, fatty acid synthase; KAS, ketoacyl-ACP synthase; KAR, ketoacyl-ACP reductase; HAD, hydroxyacyl-ACP dehydrase, EAR, enoyl-ACP reductase; FAB2, stearoyl-ACP-Δ^9^-desaturase; FAT, acyl-ACP thioesterase. Expression data for the transcripts used in the pathway are provided in (S7A Table).

Most of the transcripts encoding enzymes of the FA biosynthesis pathway were down-regulated at 35°C (Fig 9 and S7A Table). In particular, the subunits from both the Acetyl-CoA Carboxylase (ACCase) and Fatty Acid Synthase (FAS) were down-regulated (S11E–S11G Fig). Only transcripts for FAB2-2 (stearoyl-ACP Δ^9^ desaturase) and FAT (acyl-ACP thioesterase) were up-regulated at high temperature). FAB2 is responsible for the desaturation of C18:0-ACP and we found two distinct transcripts (FAB2-1/1900 and FAB2-2/68) that encode this enzyme (S11I Fig). The expression of FAB2-1 was not affected by temperature whereas the FAB2-2 was up-regulated. At 30 hours (6 hours after the temperature increase), there was no difference between the RPKM values of cells grown at 25°C versus 35°C but at 40 hrs (16 hours after temperature increase), the transcript abundance at 35°C was 2.6 times higher than at 25°C (Log2fold of 1.4), and at 54 hrs its abundance was 2.8 times higher. These two transcripts are not expressed at the same level: FAB2-1 transcript abundance was 5–7 times higher than for FAB2-2 with RPKM values of 171–178 and 25–37, respectively. The FAT gene, responsible for the release of the FA from the acyl-carrier protein, showed increased transcript abundance under higher temperature (Fig 9 and S7A Table).

### Transcript levels from triacylglycerol pathway genes under elevated temperature

**Fig 10:**
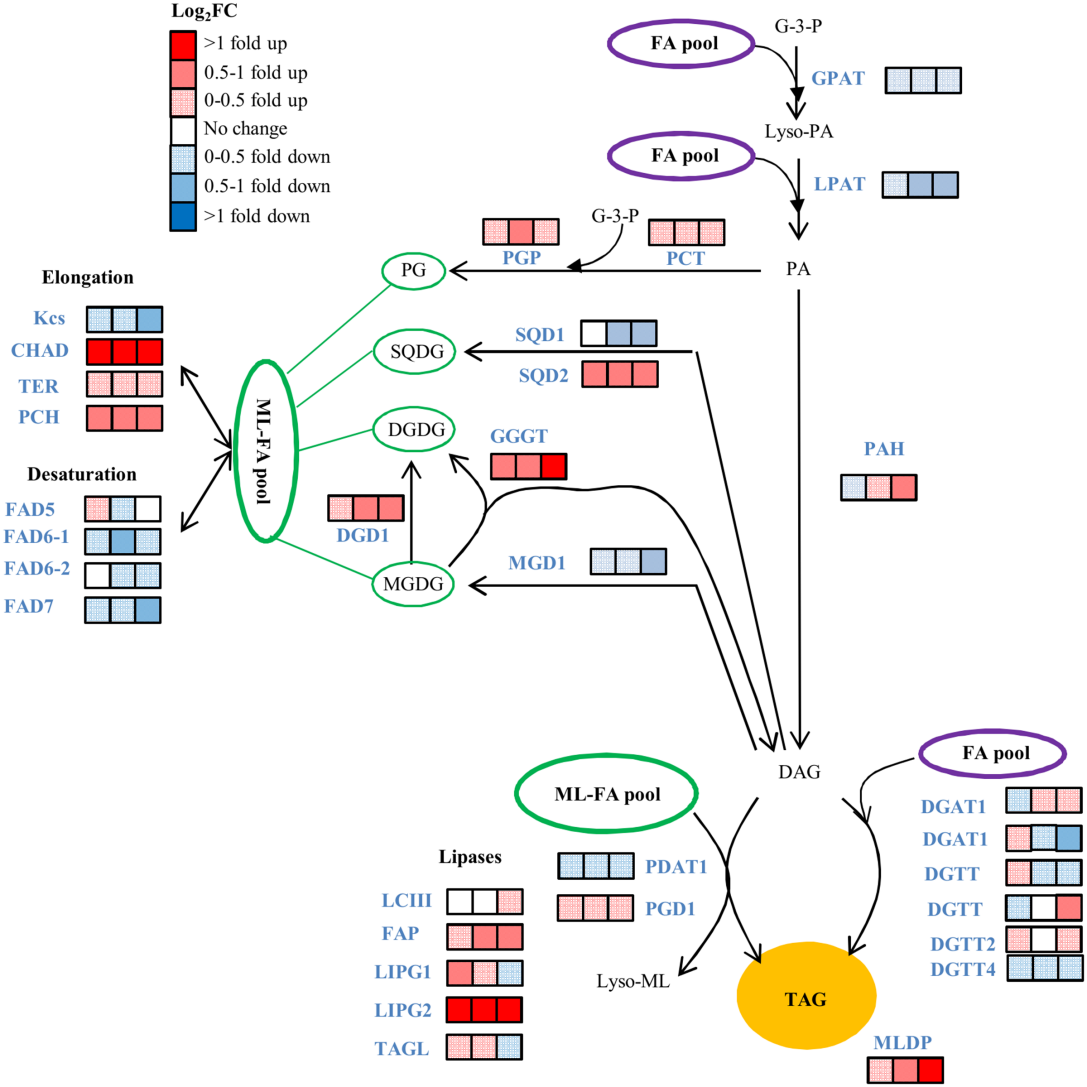
Effect of temperature on the transcript abundance of the TAG pathway genes. Relative transcript abundance changes for the transcripts of the lipid biosynthesis pathway are provided in (S7A Table). Compounds include: G-3-P, glycerol-3-phosphate; Lyso-PA, lyso-phosphatidic acid; PA, phosphatidic acid; PG, phosphatidylglycerol, SQDG, sulfoquinovosyldiacylglycerol; MGDG, monogalactosyldiacylglycerol, DGDG, digalactosyldiacylglycel, DAG: diacylglycerol, TAG, triacylglycerol. Enzymes include: GPAT, glycerol-3-P acyltransferase; LPAT, lysophosphatidic acid acyltransferase; PAH, phosphatidate phosphatase; DGAT and DGTT, diacylglycerol acyltransferase; PCT, CDP-diacylglycerol synthase; PGP, phosphatidylglycerolphosphate synthase; SQD1, UDP-sulfoquinovose synthase; SGD2, sulfolipid synthase; MGD1, monogalactosyldiacylglycerol synthase; DGD1, digalactosyldiacyglycerol synthase; PGD1, plastid galactoglycerolipid degradation1; GGGT, galactolipid:galactolipid galactosyltransferase; PDAT1, phospholipid:diacylglycerol acyltransferase; MLDP, major lipid droplet protein; Kcs, β-ketoacyl-CoA synthase; CHAD, 3-hydroxyacyl-CoA dehydrogenase; TER, Trans-2-enoyl-CoA reductase; PCH, Palmitoyl-CoA hydrolase; FAD5, MGDG specific palmitate Δ-7 desaturase; FAD6, ω-6 fatty acid desaturase; FAD7, ω -3 fatty acid desaturase; LCIII, class 3 lipase, FAP, class 3 lipase, LIP, lipase; TAGL, triacylglycerol lipase.

TAGs are synthesized by the addition of a FA to the sn-3 position on the glycerol backbone of DAG. Those FAs can be newly synthesized or can be recycled from FAs present in membrane lipids [31]. Because membrane lipids are subject to modifications such as desaturation and elongation, the FAs added to DAGs to form TAGs have very different characteristics and properties if they are newly synthesized or recycled from membrane lipids. The transcripts encoding the first two enzymes from the TAG synthesis pathway, glycerol-3-phosphate acyltransferase (GPAT) and Lyso-phosphosphatidic acid acyltransferase (LPAT) that catalyze the formation of PA from glycerol-3-phosphate (G3P) were both down-regulated at 30, 40 and 54 hrs (Fig 10 and S11L Fig). The transcript coding for phosphatidate phosphatase (PAH), responsible for the formation of DAG from PA, was first down-regulated at 30 hrs but then became increasingly up-regulated at 40 and 54 hrs (Fig 10 and S7A Table). Interestingly, similar to the FAB2 transcript, the transcript encoding for the enzyme that was up-regulated was also the transcript with the lowest overall abundance levels. Several transcripts coded for different isoforms of diacylglycerol acyltransferase (DGAT), responsible conversion of DAG to TAG in the Kennedy pathway [32]. We found two transcripts corresponding to diacylglycerol acyltransferase type 1 (DGAT) and 5 transcript corresponding to diacylglycerol acyltransferase type 2 (DGTT). DGAT1 corresponds to transcript 6208 and was the only transcript up-regulated at both 40 and 54 hrs (Fig 10). The transcripts of PL synthesis genes showed different expression patterns. The two main membrane lipids in *D*. *viridis* are MGDG and DGDG (S1 Table). The transcript for monogalactosyldiacylglycerol synthase 1 (MGD1) which synthesizes MGDG from DAG was down-regulated at all three time points while transcripts encoding the enzyme digalactosyldiacylglycerol synthase 1 (DGD1) that synthesizes DGDG from MGDG was up-regulated (S11J Fig). Two enzymes involved in channeling of FAs from the membrane lipids into TAG are phospholipid:diacylglycerol acyltransferase (PDAT1) and plastid galactoglycerolipid degradation1 (PGD1) [33,34]. Transcripts encoding those two enzymes showed opposite regulation: PDAT1 transcripts were down-regulated at all three time points while PDG1 transcripts were increased at all three time points (S7A Table). Transcripts of other enzymes in this pathway involved in membrane lipid FA modification (elongation and desaturation) showed variable regulation patterns in response to elevated temperature: in general, transcript levels of desaturases were down-regulated and transcript levels of elongases were up-regulated (S11K Fig). Several transcripts encoding for lipases that could be involved in the release of FAs from the membrane lipids for integration into TAG were up-regulated (Fig 10 and S11N Fig).

### Transcript levels of starch synthesis pathway genes under continuous light

**Fig 11:**
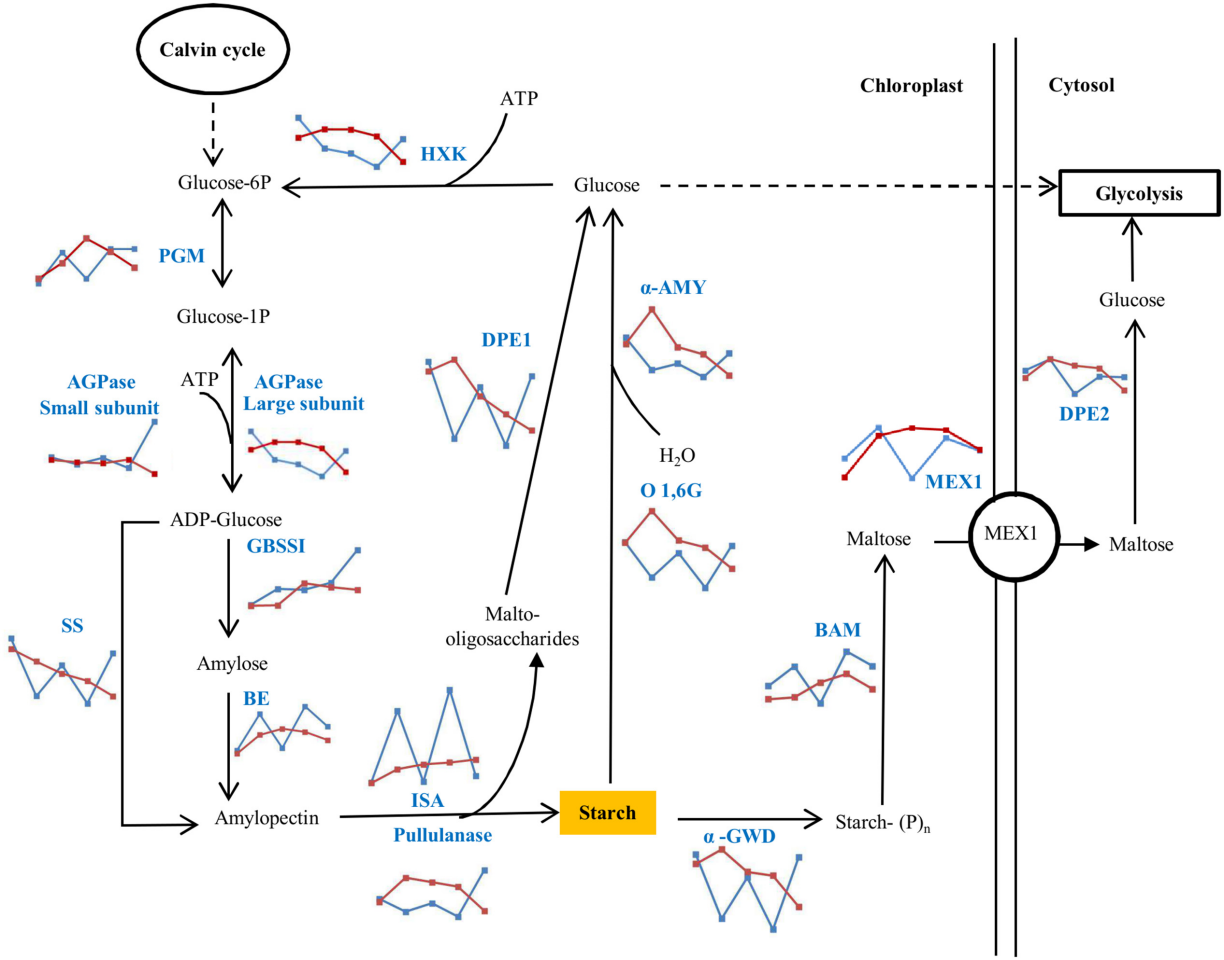
Effect of light duration on the transcript levels of the starch pathway genes. The chart represents the RPKM pattern at 25°C under LD (blue lines) and LL (red lines) at 6, 16, 30, 40 and 54 hours. Compounds include: Glucose-6P, Glucose-6-phosphate; Glucose-1P, Glucose-1-phosphate; ADP-glucose, Adenosine diphosphate-glucose. Starch synthesis enzymes include: PGM, phosphoglucomutase; AGPase, ADP-glucose phosphorylase; SS, soluble starch synthase; GBSSI, granule bound starch synthase I; BE, 1,4-alpha-glucan branching enzyme; ISA, isoamylase; Pullulanase and HXK, Hexokinase. Starch degradation enzymes include: α-GWD, alpha-glucan water dikinase; α-AMY, alpha-amylase; O 1,6G, oligo-1,6-glucosidase; BAM, beta-amylase. Disproportionating enzymes include: DPE1 (plastid isoform) and DPE2 (Cytosolic isoform), α-1,4 glucanotransferases. Transporter include: MEX1, Maltose transporter 1. Expression data for the transcripts used in the pathway are provided in (S7B Table).

Transcript abundance changes associated with the synthesis and degradation enzymes of starch were higher in response to photoperiod duration than to temperature increase (S7B Table, S11O and S11P Fig). ADP-glucose pyrophosphorylase (AGPase) which catalyzes the conversion of glucose-1-phosphate (G1P) and ATP to generate ADP-glucose did not show any difference in the abundance of small subunit transcripts at 25°C under both LD and LL until 54 hours (Fig 11). The transcripts encoding for the large subunit of AGPase under LL was up-regulated at 16, 30 and 40 hours, which may have caused the increased starch accumulation under LL (Fig 4). Transcripts for starch synthase (SS) which uses ADP-glucose as a substrate to generate amylopectin followed a strong diurnal pattern of regulation under LD, but was increasingly down-regulated under LL. The transcripts encoding for the branching enzyme (BE) shows opposite regulation under both light conditions in comparison to SS. The debranching enzymes like isoamylase (ISA) and pullulanase hydrolyze amylopectin to release maltose and malto-oligosaccharides from starch and can also be involved in modifications of branch density and therefore crystallinity of the starch. Transcripts encoding for both the ISA and pullulanase followed diurnal pattern under LD cycle, with opposite pattern of regulation. Malto-oligosaccharides are hydrolyzed in the plastid by the disproportionating enzyme 1 (DPE1) into glucose and by starch phosphorylase into G1P. Transcripts encoding for DPE1 showed a strong diurnal regulation under LD, whereas under LL it was increasingly down-regulated. Transcripts of genes involved in starch degradation via hydrolysis such as oligo-1,6-glucosidase (O-1,6G) and alpha-amylase (α-AMY) or by phosphorylation such as α-GWD (alpha-glucan water dikinase) and BAM (beta-amylase) were identified. Among these degradation enzymes, transcripts encoding α-GWD, O-1,6G, and α-AMY followed a diurnal pattern of regulation under LD cycle with up-regulation in light and down-regulation in dark. Under LL, the transcripts encoding those degradation enzymes were up-regulated at 16 hrs and then increasingly down-regulated until 54 hours (Fig 11). Under LD, BAM transcript was up-regulated in the dark and down-regulated in the light. The maltose generated from BAM in plastids is exported into the cytosol through the maltose transporter 1 (MEX1) and hydrolyzed by disproportionating enzyme 2 (DPE2) into glucose. Transcripts for both of these proteins, MEX1 and DPE2, had a similar pattern of regulation under LD (Fig 11 and S11O Fig).

### Expression profiles

We performed a time-course analysis using the STEM program that allows the clustering of transcripts into expression profiles by their transcript abundance change patterns (S2 Protocol). Among all 9400 transcripts, 46% were assigned a profile in the LD time series but only 28% were assigned a profile in the LL time series. We identified two significant expression profiles for transcripts under LD condition and six for transcripts under LL light (S9 Fig). The two significant LD expression profiles consist of transcripts up-regulated during the dark phase (expression profile #10, 23% of the transcripts) or transcripts down-regulated during the dark phase (expression profile #5, 12% of the transcripts). The two most prominent LL profiles are for transcripts whose expression slowly decreases over the course of the experiment (expression profile #2, 10% of the transcripts) or whose expression first increases and then decreases (expression profile #12, 4% of the transcripts) (S9B Fig). In the LD time series, profile 10 was enriched with transcripts from the nucleic acid and energy categories whereas profile 5 was enriched with transcripts from the movement category (S9A Fig). Profile #13 from the LL time series shows a steady increase in expression over the course of the experiment. Several categories are enriched in this profile: photosynthesis, nucleic acid, stress response and movement (S9B Fig).

## Discussion

When we tested the most commonly described *Dunaliella* species for their responses to different environmental stresses including light duration and temperature, we found a wide spectrum of physiological and metabolic differences among those species. This is not very surprising, because of their large evolutionary distances and adaptation to different marine and salt lake habitats worldwide [4]. We focused on a strain of *Dunaliella viridis* that was identified when we attempted to confirm the identity of cultures we received from another lab. Our strain, *Dunaliella viridis* dumsii, differs by three nucleotides in the internal transcribed spacer 2 (ITS2) region from the closest established *Dunaliella* genotype, which necessitated the unique name of our strain. Sequence information of ITS2 region of *Dunaliella viridis* dumsii available at Genbank (KP057241).

This *D*. *viridis* ecotype accumulates TAG under elevated temperature without a large drop in growth rate when grown under LL. To characterize the underlying mechanisms which enable these algae to accumulate storage components like TAGs at a fast growth rate, we analyzed the time-resolved metabolic and genomic responses to each environmental change separately as well as in combination. Interestingly, the accumulation of TAGs is increased by either stress independently, which results in an additive effect of TAG accumulation when both stresses are applied. We are discussing here the specific effects of either stress and the integration of both stresses.

### Cell cycle regulation by photoperiod

**Fig 12:**
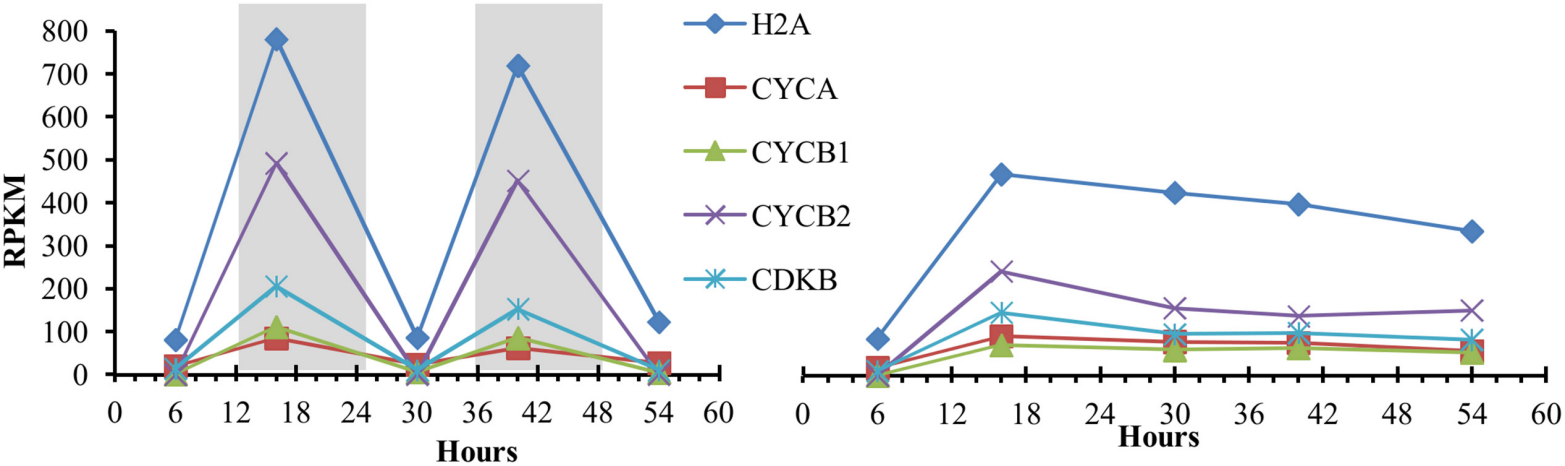
Cell cycle regulated genes show diurnal regulation under LD, but constant transcript levels in LL. Lines represent the changes in transcript abundance (RPKM) for each of the genes. Genes are: H2A, histone H2A; CYCA, A-type cyclin; CYCB1, B-type cyclin 1; CYCB2, B-type cyclin 2; cyclin dependent kinase B.

Exposure to continuous light (LL) had a significant effect on increasing the cell division rate compared to LD, while temperature changes had no significant effect (Fig 2A) during the time period of this experiment. The algae cells divided during the dark period (S2 Fig), but acquired an apparently constant cell division rate when grown under LL, indicating that cell division is not under circadian control but light responsive (diurnal). GO term enrichment analysis of differentially expressed genes shows the cell cycle category as enriched under LL (Table 1). The algal cell cycle has been found to be closely connected to photoperiod with cells being in the G_1_ phase during the light period and then advancing through S, G_2_, and M phases during the dark period. The diurnal pattern of cell growth in the light and cell division in the dark is thought to allow for maximum collection of energy [35]. The synchrony of cell cycle progression in *D*. *viridis* can be seen from the transcript levels of canonical S and M phase-expressed genes, showing high abundances in the dark, but not in the light (Fig 12 and S8 Table). The diurnal pattern of expression of these genes fades into a continuous expression pattern under LL suggesting that the population of cells is no longer dividing synchronously. The loss of synchronous growth most likely stems from the lack of the dark induced pause in the cell cycle, which allows cells to resume cell cycle progression as soon as cell division is complete. The increased cell density under LL indicates that the time required for completion of a cell cycle is less than 24 hours under sufficient nutrient supply and that the cells have the ability to progress through the cell cycle constantly.

While there was no significant effect of temperature on cell division rate, the cell size was affected by temperature only under LL. Cells grown at 35°C were significantly larger than 25°C (Fig 2B and S3 Fig). The increased cell size in *D*. *viridis* correlates with a significant increase in PL as it would be expected from the need for more membrane in cells with larger surface (Fig 7).

### Photosynthesis under heat stress

The most abundant polar lipids are DGDG and MGDG, which are exclusively found in the chloroplast envelope and thylakoid membranes [36]. In our experiment, changes in cellular DGDG and MGDG quantities occurred in response to light duration and temperature (Fig 7), and strongly correlated with the chlorophyll contents under the respective growth conditions (Fig 3A). In response to elevated temperature, cellular chlorophyll content and MGDGs increased, suggesting that the photosynthetic machinery of *D*. *viridis* cells acclimate to heat stress when switched from 25°C to 35°C as a result of enhanced thermal stability. A similar response was observed in *Chlamydomonas* [37]. The photosystem II complex is especially susceptible to heat [38–40]. Most of the photosystem I and II subunit related genes at 35°C had about 2-fold greater RPKM at 30 hrs in comparison to 25°C under LL (S11C and S11D Fig). Chlorophyll is coordinated in the membrane by the light-harvesting complex proteins (LHCP). Transcript levels of those major LHCPs were transiently increased 3- to 4-fold within 6 hours after transfer of cells to elevated temperatures, but returned to similar levels as in cells grown at 25°C thereafter (S11B Fig).

Membrane lipid remodeling has been shown to be a mechanism in *Arabidopsis* and other vascular plants to modify the physical properties of membranes associated with temperature stress. Higher temperature increases membrane fluidity which can be countered by an increase in the degree of FA saturation to maximize hydrophobic interactions and thereby counteract the increase in fluidity under higher temperature. The increase in growth temperature of *Arabidopsis* resulted in a decrease in the trienoic FAs and an increase in C16:0 and C18:2 levels in the membrane lipid composition in leaves [41]. Similar to *Arabidopsis* leaf cell membranes, a significant decrease in the saturation levels in membrane lipid composition were observed when *Dunaliella* cultures were transferred from 25°C to 35°C under LL (S6 Fig). The changes in the FA composition of membrane lipids suggest the possibility of membrane remodeling in *D*. *viridis* under heat stress. *Chlamydomonas* mutants defective in ω-3 desaturase (homolog of FAD7 in plants) have a significant reduction in trienoic membrane lipid FAs under short term heat stress contributing to increased photosynthetic thermotolerance compared to wild type [42]. Similar to *Chlamydomonas* FAD7 mutants, we found that under heat stress there was a down-regulation of FAD7 in *D*. *viridis* (Fig 10). Increased levels of DGDG and/or the ratio of DGDG to MGDG play an important role in basal as well as acquired thermotolerance in *Arabidopsis* [43], which contributes to increased thylakoid membrane stability at elevated temperature [44]. *Arabidopsis* mutants defective in DGD1, the key enzyme in the conversion of MGDG to DGDG, were sensitive to temperature stress due to their inability to increase their DGDG levels and DGDG:MGDG ratios in response to elevated temperature [43]. These *Arabidopsis* experiments were carried out with plants grown under a LD cycle. We found that in *Dunaliella*, this response to elevated temperature is dependent on the light period conditions. Under LD cycle a temperature increase resulted in an increase in steady-state levels of MGDG and a decrease in DGDG levels (S1 Table), suggesting a reduction or inhibition in the conversion of MGDGs to DGDGs. However, under LL, the elevated temperature increased the steady-state levels of both, DGDG and MGDG. These results show a strong interaction of light duration and temperature stress on the composition of chloroplast membrane lipids. It is noteworthy to mention that *D*. *viridis* possess PC, the most common membrane lipid in plants [45], while *Chlamydomonas* does not [46].

HSPs and carotenoids participating in the xanthophyll cycle have been shown to play a role in protecting the photosystem II complex against heat stress [37,47–50]. Our transcriptome analysis identified several HSP genes that were up-regulated in response to elevated temperature (S5 Table). This is consistent with a proteomics analysis on *Chlamydomonas* grown at 42°C [51].

### Metabolic acclimation to continuous light

Cells under LL at 25°C divided the fastest (Fig 2A) and did not shown an increase in their cellular chlorophyll content (Fig 3A) or polar lipid content (Fig 7) as they did when grown under a LD regime at 25°C. These algae cells (LL, 25°C) also had decreased levels of chloroplast membrane lipids (S1 Table), but increased amounts of the storage components starch (Fig 4) and TAGs (Fig 5A). The lower chlorophyll content might be due to irradiance stress as observed in *D*. *salina*, where cells grown under high-light exhibit lower chlorophyll levels than those grown under low-light [52]. Cells acclimate to high levels of irradiance by reducing the chlorophyll antenna size of the photosystems as shown in *D*. *tertiolecta* [53], *D*. *salina* [54] and *C*. *reinhardtii* [55]. Light harvesting chlorophyll antenna size varies according to variable amounts of LHC I and LHC II with the respective photosystems in *D*. *salina* [56–58]. GO term analysis showed enrichment in transcripts of chloroplast and photosynthesis as top categories for light factor (Table 1). Additionally, several photosystem transcripts were the most differentially regulated in response to light duration (S4 Table and S11B–S11D Fig) which might explain the cells strategies to acclimate to LL. *Chlamydomonas* cells have been shown to have increase carbonic anhydrase (CA) activity when grown photo-autotrophically and exhibit higher affinity for inorganic carbon uptake [59]. The most dramatic effect in response to LL at either temperature (25°C or 35°C) was observed in the transcript abundance changes of specific carbonic anhydrase isoforms (S11A Fig). When light absorption exceeds CO_2_-fixation rates, the excitation energy can cause photoinhibition. To adjust their photosynthesis to excess light energy (as under LL), the cells can either increase photoprotective mechanisms for non-photochemical quenching or increase their CO_2_ fixation rates. Based on our transcript data, *Dunaliella* cells use both strategies to compensate for LL exposure. It has been shown in *Chlamydomonas* that the major extracellular carbonic anhydrase (HLA5) is induced by light or low CO_2_ [60]. Increasing the carbonic anhydrase as part of the carbon concentrating mechanism, enables the cell to productively use the excitation energy for the uptake of C_i_ against a concentration gradient, and for the fixation of CO_2_. The increase in cell number and storage components (starch and TAGs) requires a net increase in CO_2_ fixation in cells under LL exposure. Photoprotection by non-photochemical quenching is apparent from the differential expression of transcript 10594 encoding chloroplast carotene biosynthesis-related (CBR) protein (S4 Table). Under LD conditions, expression varied from ~1500 RPKM to no expression in the dark (~5–10 RPKM), while under LL, transcript abundance remained above 400 RPKM during the course of the experiment. CBR is a homolog of vascular plant ELIP proteins (early light-induced proteins), shown to bind zeaxanthin to form photoprotective complexes within the light-harvesting antennae for non-photochemical quenching [61]. CBR proteins accumulate in *D*. *salina* cells under high light stress and are thought to be indicative of irradiance stress [61–63]. The role of this protein may explain its up-regulation in response to LL stress.

Cells grown under LL contained 25% less total membrane lipids compared to cells grown LD under 25°C at 54 hours (S1 Table). This decrease was due to the reduction of the chloroplast membrane lipids MGDG and DGDG (Fig 7), and strongly correlates with the low content of chlorophyll under LL at 25°C (Fig 3A). While the content of total membrane lipids decreased under LL at 25°C (Fig 7), TAG content increased 10-fold, accumulating oleic acid (C18:1) (Fig 5A). In *Nannochloropsis*, galactolipids (MGDG and DGDG) decrease and TAG increases, as irradiance level was shifted from low to high [53].

### Significant transcriptome analysis

Several transcripts encoding for HSPs were up-regulated in response to heat stress (S5 Table). This is consistent with the results from a proteomics analysis of *Chlamydomonas* grown at 42°C [51]. Protein degradation was one of the enriched categories in our GO term enrichment analysis for heat stress (Table 1). This is in agreement with our metabolic data, because the cells grown under 35°C had a sudden increase in protein content directly after the increase in temperature. This is possibly due to production of HSPs, while the decreased protein content under constant elevated temperature, and is possibly due to peptidase activity (Fig 3B and S5 Table). In several cases, we had more than one transcript encoding for the same enzyme (different isoforms). Interestingly, the transcripts that showed differential expression (up-regulation or down-regulation) were actually the transcripts with the lowest relative abundance (low RPKMs) of the two isoforms. For example, FAB2 has one isoform which maintains steady state expression and the other isoform is lowly expressed, but shows differential expression in response to high temperature (S7A Table). This was also found in *Chlamydomonas*: the expression of DGTT1, one of the 5 type-2 DGAT from *Chlamydomonas* showed the highest fold change but had the lowest transcript abundance [31]. This could be an important mechanism for the regulation of specific pathways.

### Mechanism of starch accumulation under photoperiod

We identified enzymes from the transcriptome analysis to reconstruct the classical starch biosynthesis and degradation pathways (Fig 11) [64]. Transcript abundances for several enzymes of the synthesis and degradation pathways were under strong diurnal control: SS, DPE1, α-AMY, O-1,6G, and α-GWD had lower transcript levels in the dark and higher levels in the light, while the BE, ISA, BAM, MEX, and the cytosolic DPE2 show the opposite cycling with high transcript levels in the dark and lower levels in the light (S11O and S11P Fig). We did not see any changes in starch quantities during those time points under LD, but it is possible that these time points did not reflect times of maximum difference, but mid-points in accumulation and degradation and therefore fail to show diurnal changes.

Starch is accumulated in leaves of vascular plants during the light period and degraded during the subsequent dark phase [12]. Several light regulated enzymes have been shown to be under control of the circadian clock [65,66]. However, starch metabolism in algae has been shown to have some distinct differences compared to vascular plants. In *Chlamydomonas* starch biosynthesis and degradation is controlled by a circadian rhythm, but the levels of starch content peaked in those cells during the dark phase and were lowest during the light period [67]. The authors of that study hypothesize that the difference in starch accumulation is due to the difference in the cells developmental state. Regulation of starch accumulation in mature cells in source tissues may be under a different control mechanism than in fast dividing cells. However, these *Chlamydomonas* cells were grown heterotrophically on acetate, which might have a major impact on the regulation of carbon storage metabolism. In the green pico-algae, *Ostreococcus tauri*, starch accumulated similar to vascular plants during the light period and was degraded during the dark phase [68].

The first rate-limiting step for starch biosynthesis is the formation of ADP-glucose by AGPase, a heterotetramer of 2 large and 2 small subunits [69]. The activity of AGPase in vascular plants is regulated allosterically by triosephosphates and phosphate as well as via posttranslational redox modification. Reversible formation of intermolecular cysteine bridges between the two catalytic small subunits of AGPase is formed by a NADP-thioredoxin reductase C complex leading to changes in enzyme activity [69–71]. Reversible redox activation has been shown for other enzymes in the starch biosynthesis or degradation pathways including ISA, Limit Dextrinase, SS, GWD, and the BE in *Arabidopsis* [72]. This conserved mechanism of posttranslational regulation of enzyme activity in vascular plants is apparently lacking in green unicellular algae. AGPase activity in *Ostreococcus tauri* is not regulated by redox modification [73], and the conserved cysteine residues in AGPase, GWD, and ISA that are required for redox regulation are replaced by other amino acids in *Chlamydomonas*, *Ostreococcus* and *Micromonas* [68]. We analyzed the respective sequences from the four *Dunaliella* species described here, that were either sequenced in our lab or through the oneKP project [74–76]. Sequence alignment of the transcriptome data for the identified cysteine motifs, which are responsible for the redox activation of their respective enzymes showed that *D*. *viridis*, *D*. *tertiolecta*, *D*. *salina* and *D*. *primolecta* enzymes do not contain the conserved cysteine residues and therefore are likely not regulated via redox modification (S10 Fig). Due to the lack of redox modification of key starch metabolic enzymes, the authors of the respective studies in *Chlamydomonas* and *Ostreococcus* concluded that the major regulatory mechanism is on the transcriptional level [67,68]. This hypothesis is the most likely scenario in *Dunaliella* species as well.

While our sampling time points were not designed to capture full diurnal cycles, we did ensure that harvesting was carried out at the same intervals after transition to light (6 hrs) or after transition to dark (4 hrs). While the time points cannot represent diurnal changes, some transcripts show significant differences under LD that are not apparent when grown under LL. In *D*. *viridis*, overall starch only accumulated under LL, not under LD (Fig 4). The overall decreasing RPKM values for the transcripts of starch degradation enzymes under LL (Fig 11) support the fact that cells under LL at 25°C might accumulate starch by repressing degradation. Two distinct pathways for starch degradation, the hydrolytic and phosphorolytic routes, were observed in *D*. *viridis* [77]. The hydrolytic enzymes, α-AMY and oligo-1,6-glucosidase (O 1,6G), catalyze the hydrolysis of starch to α-D-glucose. The released α-D-glucose can be exported to the cytosol and undergo glycolytic conversion to pyruvate, or be phosphorylated by hexokinase (HXK) into glucose-6-phosphate for reentry into starch synthesis pathway [77] (Fig 11). The phosphorolytic pathway enzyme, α-GWD phosphorylates starch prior to degradation by BAM into maltose which is exported via the plastidial transporter MEX1 into the cytosol [78]. The fact that the *D*. *viridis* cells accumulated starch when grown under LL implies that starch biosynthesis occurs during the light period. In order to accumulate, the rate of starch degradation must be lower than the rate of synthesis. This is not reflected in the changes in transcript levels of both degradation pathways—phosphorolytic (α-GWD) and hydrolytic enzymes (α-AMY and O 1,6G) as well as DPE1 (Fig 11). Despite the loss of diurnal variation, transcript levels for the degrading enzymes were higher under LL compared to LD.

### Effects of light duration and temperature on *de nov*o FA synthesis

The three major lipid fractions (TAG, polar membrane lipids, FFA) accumulated when cells were grown at 35°C compared to 25°C under LL (Figs 5A and 7, S7 Fig). Because FAs are the building blocks of the different lipid fractions, an increase in total lipids requires an increase in FA synthesis or a decrease in their catabolic process (β-Oxidation). We identified the transcripts coding for the enzymes of the FA synthesis pathway, the prokaryotic and eukaryotic lipid biosynthesis pathways, as well as those for lipid and fatty acid catabolism (S11E–S11N Fig). ACCase is a key regulatory enzyme controlling the first committed step of plant FA synthesis [79]. The transcript levels of the four ACCase subunits: Alpha-carboxyltransferase subunit (α-CT), Beta-carboxyltransferase subunit (β-CT), Biotin carboxylase subunit (BC) and Biotin carboxyl carrier protein subunit (BCCP) in *D*. *viridis* were under diurnal control with higher levels observed mostly down-regulated in response to heat (Fig 9 and S11G Fig). A similar expression pattern was observed in *Chlamydomonas* under N-deprivation; although TAG accumulated, the transcript abundances of most of the genes in the *de novo* fatty acid synthesis pathway (ACCase subunits in particular) were down-regulated [80].

Many biochemical pathways are controlled by a feedback mechanism where the end product inhibits the activity of the regulatory enzyme which is often the first committed step of the pathway. In oilseed plants, there is evidence for feedback regulation of plastidic ACCase by 18:1- acyl-carrier protein (C18:1-ACP) [81]. Additionally in tobacco, ACCase protein levels did not change during the feedback inhibition, indicating that inhibition of FA synthesis occurred through biochemical or posttranslational modification of ACCase [82]. Based on our transcriptome data, the transcript abundance of the FAT that releases ACP from 16:0-ACP, 18:0-ACP or 18:1-ACP to yield FFAs is up-regulated under high temperature conditions (Fig 9). We hypothesize that the increased FAT under higher temperature, would lead to the significant decrease in the pool of acyl-ACP in the plastid, thereby releasing the negative feedback regulation on ACCase. This may lead to increased flux through ACCase and explains the increased contribution of more FAs to total lipids under higher temperature. Overexpression of a thioesterase gene in *Phaeodactylum tricornutum* led to an increase in total FA content by 72% while the FA composition did not change [83]. FAB2-2 also showed increased expression at higher temperature (Fig 9), which could provide increased amounts of 18:1, a precursor of polyunsaturated FAs.

### Mechanism of TAG accumulation under heat stress and light duration

#### TAG biosynthesis through the acyl-CoA dependent (Kennedy) pathway

TAG is the most important lipid for biofuel production [13]. We observed a more than 15-fold increase in cellular TAG content when the cells were grown at 35°C under LL than at 25°C under LD (Fig 5A). At 54 hrs, the cells grown under LD at 25°C had a relatively lower TAG content than other conditions tested (Fig 5A). The low TAG content result is similar to the results of a TAG content survey done of various *Dunaliella spp*. grown under a light:dark cycle of 14/10 hr at 24°C/20°C with a new and sensitive UPLC-MS analytical technology [84]. GPAT and LPAT which form PA are common to both the acyl-CoA dependent and acyl-CoA independent pathways. Transcript levels of both enzymes showed strong diurnal variation to LD but were expressed at a constant medium level during LL and were down-regulated at 35°C (Fig 10 and S11L Fig). DGATs, the main enzymes in the acyl-CoA dependent TAG synthesis pathway (Fig 10), are responsible for TAG formation by addition of an acyl-CoA to the *sn*-3 position of the glycerol backbone. The majorities of algal species encode at least one type 1 DGAT (DGAT1) and encodes multiple type 2 DGATs (DGGT) [85]. In *Chlamydomonas*, there are 5 type-2 DGATs: *DGTT1-DGTT5* and *DGTT1* have been shown to be up-regulated in response to N-starvation as is DGAT1 [31,80]. We found two *DGAT1* gene and 4 *DGTT* genes in *D*. *viridis* based on sequence similarity to *Chlamydomonas* proteins, which were differentially regulated at 35°C (Fig 10 and S11L Fig). The two *DGAT1* genes had opposite pattern of regulation. *DGAT1* (transcript 6208) was down-regulated 6 hours after the temperature switch to 35°C, but then was up-regulated at the higher temperature whereas *DGAT1* (transcript 3780) was initially up-regulated after the temperature shift to 35°C and then down-regulated at 40 and 54 hrs (Fig 10 and S7A Table). One of the *DGTT* genes (transcript 9514) had slight up-regulation with increasing time in response to higher temperature. In *Chlorella vulgaris*, DGAT had the largest increase in abundance of all analyzed proteins from the FA and TAG biosynthesis pathway under N-deprivation [86]. We hypothesize that either the enzymes of the acyl-CoA dependent pathway for TAG synthesis are not transcriptionally regulated in *D*. *viridis*, or the pathway is not up-regulated under high temperature and thus the acyl-CoA dependent TAG synthesis pathway does not have a major role in TAG accumulation under high temperature.

#### TAG biosynthesis through membrane lipid recycling of fatty acids

When considering TAG synthesis through the acyl-CoA independent pathway (Fig 10), we need to consider the recycling of the chloroplast membrane lipids for TAG formation. Under higher temperature, all enzymes involved in membrane lipid formation were up-regulated except for sulfoquinovosyldiacylglycerol (SQD1) and MGD1 (Fig 10). At 25°C, MGDG is the second most abundant chloroplast membrane lipid after DGDG, but upon growth at 35°C its content more than doubles and it becomes the most abundant membrane lipid (Fig 7). The accumulation of MGDG at 35°C and the down-regulation of the *MGD1* gene suggest this process is either not transcriptionally regulated, but alternatively regulated, or an unknown enzyme/pathway for the biosynthesis of MGDG exists in *D*. *viridis*. In *Arabidopsis*, MGD1 is post-transcriptionally regulated by PA and PG [87]. At 35°C, increases in both PA and PG (S1 Table), suggest a possibility for the post-transcriptional control of MGD1 activity in *D*. *viridis*. In *Chlamydomonas*, PDAT and PGD1 were found to be important in membrane lipid recycling for TAG accumulation [31,34,80]. Under N-starvation in *Chlamydomonas*, PDAT1 is a protein found in purified lipid bodies [88], although its expression for TAG accumulation is not clear. *PDAT1* was found to be up-regulated under N-starvation [80]. However it was also shown that if PDAT contributed appreciably to the TAG formation under favorable growth conditions, its contribution under N deprivation was minor [89]. In *D*. *viridis* at 35°C, *PDAT1* is down-regulated at all-time points suggesting that its contribution to recycling of membrane lipids for TAG accumulation is only minor unless the enzyme is not transcriptionally regulated (Fig 10). *PGD1* was up-regulated at 35°C (Fig 10), which is similar to TAG accumulation in *Chlamydomonas* under N-deprivation [31,34]. Presumably, the mechanism of TAG accumulation by membrane lipid recycling using PDAT1 and PGD1 is similar for higher temperature in *D*. *viridis* and for N-deprivation in *Chlamydomonas*.

In addition, lipases are thought to be involved in membrane lipid recycling. We found several lipases highly up-regulated at 35°C (Fig 10 and S11N Fig), similar to lipases upregulated by N-starvation in other algae for TAG accumulation [31,90]. Another enzyme involved in membrane remodeling in *Arabidopsis* under freezing stress is galactolipid: galactolipid galactosyltransferase (GGGT), which uses MGDG as both a donor and acceptor molecule and forms DGDG and DAG [28]. This enzyme was up-regulated in *D*. *viridis* at 35°C (Fig 10). Based on our findings and insights from other research, we hypothesize that TAG accumulation in *D*. *viridis* under heat stress could be mainly due to the membrane lipid recycling of FAs.

In summary, environmental conditions enabled the production of high levels of oil during fast growth in *D*. *viridis*. The effects of eliminating the dark period in the growth cycle of *D*. *viridis* resulted in a loss of cell cycle synchronicity and faster growth rates. The accumulation of TAG and starch in *D*. *viridis* cells were apparently due to the lack of degradation that usually occurs during the dark period. Temperature changes did not have any effect on short-term growth rates, but did lead to increases in chlorophyll, starch and lipid content likely due to higher photosynthesis rates under elevated temperature. The most interesting aspect was that the effects of light duration and temperature increase were independent but additive with respect to TAG accumulation. The higher temperature also increased the degree of saturation in the TAG fatty acids. Saturated fatty acids in the TAG fraction are advantageous, because it reduces the cost for hydrogenation during refining for fuel production. Transcriptome analysis indicated that TAG accumulation in *D*. *viridis* under heat stress could be mainly due to the membrane lipid recycling of FAs.

## Supporting Information

S1 Fig Oil accumulation in different *Dunaliella* species. (PPTX)

S2 Fig Cell division in *D*. *viridis* grown under 12:12 LD cycle occurs at night. (PPTX)

S3 Fig Neutral lipids accumulation monitoring in *D*. *viridis* by Nile red staining and microscopy. (PPTX)

S4 Fig Separation of total lipids by thin layer chromatography—representative plate. (PPTX)

S5 Fig Major Phospholipid (A) and Lyso-phospholipid (B) classes respond to temperature and photoperiod changes at 54 hrs. (PPTX)

S6 Fig Fatty acid composition of galactosyldiacylglycerols and phosphadityllglycerol at 54hrs show distinct responses to temperature and light period changes. (PPTX)

S7 Fig Free fatty acids content increases at higher temperature under LL (A) and less saturated fatty acids are present in the FFA fraction (B). (PPTX)

S8 Fig Expression of genes determined by qPCR matches expression determined from transcriptome assembly. (PPTX)

S9 Fig Significant STEM gene expression profiles and categories of transcripts enriched for the time series of cells grown under light:dark conditions (A) and under continuous light conditions (B). (PPTX)

S10 Fig Multiple sequence alignments for the small (beta) subunit of ADP-glucose pyrophosphorylase (A), GWD1 (Alpha glucan, water dikinase) (B) and Beta-amylase (C). (PPTX)

S11 Fig Transcript profile for Carbonic anhydrases (A), Light Harvesting Complexes (B), Photosystem II (C), Photosystem I (D), ACCase subunits (E), Ketoacyl-ACP synthase (F), Fatty acid synthase (G), Membrane lipid synthesis (H), Fatty acid desaturation and elongation (I), Lipid and TAG synthesis (J), Major Lipid Droplet Protein (K), Lipases (L), β-Oxidation (M), Glyoxylate cycle specific enzymes (N) and Starch metabolism (O and P). (PPTX)

S1 Protocol Neutral lipids accumulation monitoring by Nile Red staining and microscopy. (DOCX)

S2 Protocol Transcriptome analysis. (DOCX)

S1 Table Distribution of different membrane lipid species in *Dunaliella viridis* under our 4 growth conditions at 54 hours. (DOCX)

S2 Table Summary of Velvet and Oases assemblies. (DOCX)

S3 Table Summary of differentially expressed transcripts at 16, 30, 40 and 54 hrs for factors: light, temperature and combined light and temperature. (DOCX)

S4 Table Transcripts differentially expressed under continuous light at 6, 16, 30, 40 or 54 hrs. (DOCX)

S5 Table Transcripts differentially expressed under high temperature at 30, 40 or 54 hrs. (DOCX)

S6 Table Transcripts differentially expressed under light and temperature at 30, 40 or 54 hrs. (DOCX)

S7 Table Transcripts expression data used in pathways from Figs 9–11. (DOCX)

S8 Table Representative cell cycle genes differentially expressed under continuous light. (DOCX)

S9 Table Primers used for real-time PCR. (DOCX)

S10 Table Sequence accession used for multiple sequence alignment of the small (beta) subunit of ADP-glucose pyrophosphorylase, GWD1 (Alpha glucan, water dikinase) and Beta-amylase. (DOCX)

S11 Table All differentially expressed transcripts information from transcriptome dataset. (XLSX)
