# Supplementary material for: Interaction of Temperature and Photoperiod Increases Growth and Oil Content in the Marine Microalgae Dunaliella viridis
Source: PLoS One. 2015 May 19;10(5):e0127562. doi: 10.1371/journal.pone.0127562 (PMC4437649; doi:10.1371/journal.pone.0127562)
Supplement: S1 Fig — (PPTX) [file pone.0127562.s001.pptx]

## Slide 1
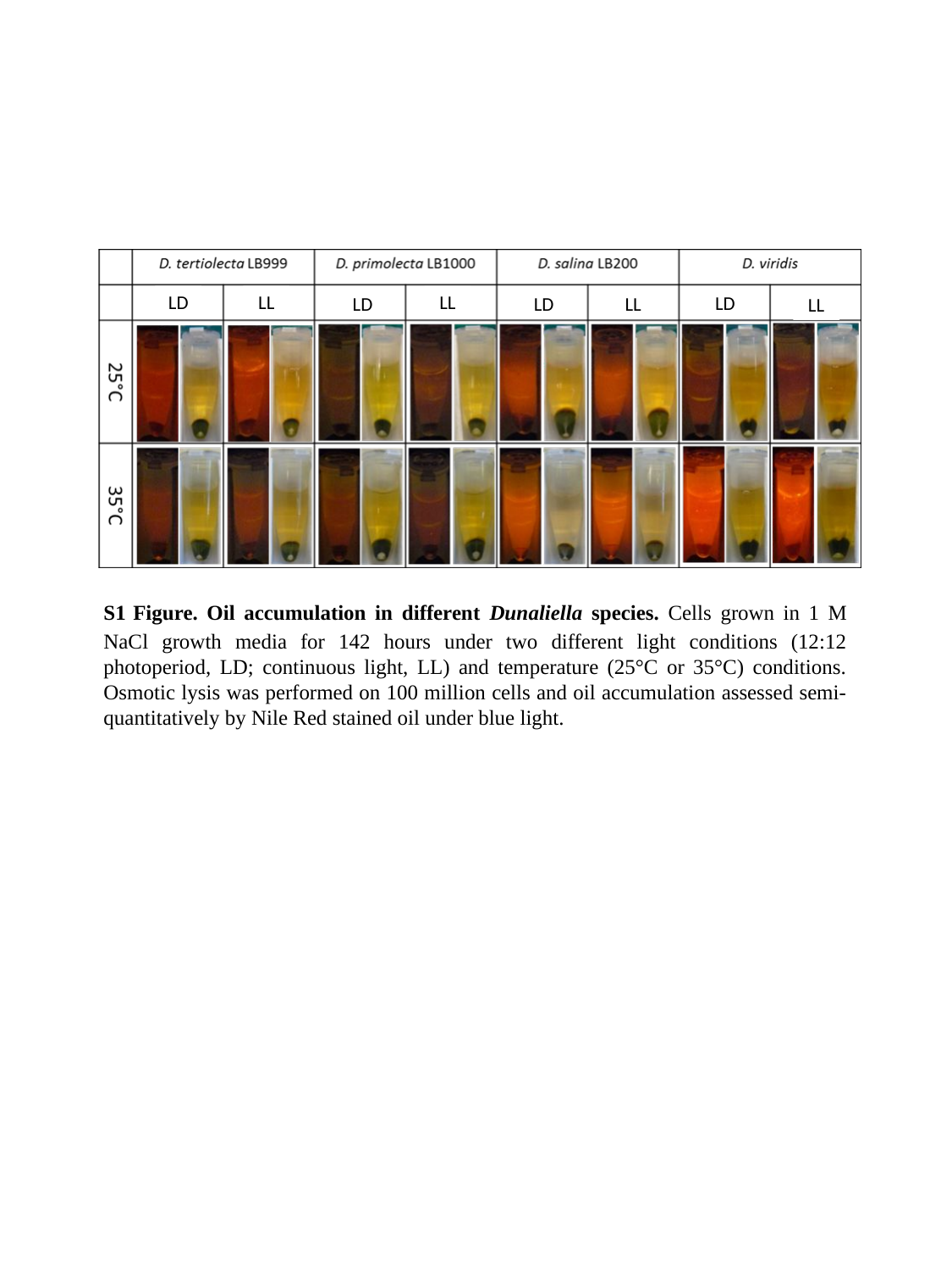

LL
LL
LL
LL
LD
LD
LD
LD
S1 Figure. Oil accumulation in different Dunaliella species. Cells grown in 1 M NaCl growth media for 142 hours under two different light conditions (12:12 photoperiod, LD; continuous light, LL) and temperature (25°C or 35°C) conditions. Osmotic lysis was performed on 100 million cells and oil accumulation assessed semi-quantitatively by Nile Red stained oil under blue light.
