# Supplementary material for: Interaction of Temperature and Photoperiod Increases Growth and Oil Content in the Marine Microalgae Dunaliella viridis
Source: PLoS One. 2015 May 19;10(5):e0127562. doi: 10.1371/journal.pone.0127562 (PMC4437649; doi:10.1371/journal.pone.0127562)
Supplement: S2 Fig — (PPTX) [file pone.0127562.s002.pptx]

## Slide 1
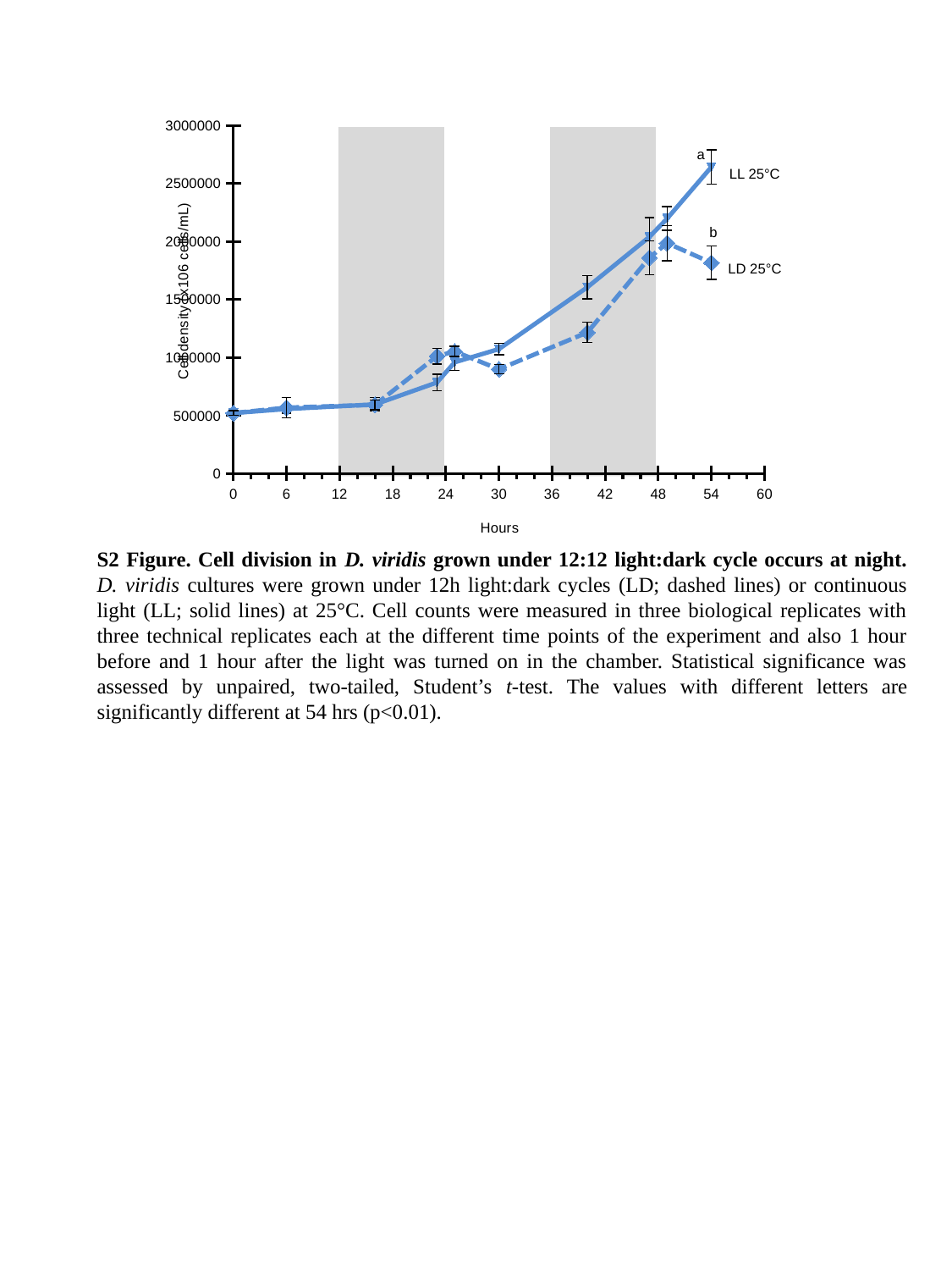

### Chart
| Category | LD 25°C | LL 25°C |
|---|---|---|
a
LL 25°C
b
LD 25°C
S2 Figure. Cell division in D. viridis grown under 12:12 light:dark cycle occurs at night. D. viridis cultures were grown under 12h light:dark cycles (LD; dashed lines) or continuous light (LL; solid lines) at 25°C. Cell counts were measured in three biological replicates with three technical replicates each at the different time points of the experiment and also 1 hour before and 1 hour after the light was turned on in the chamber. Statistical significance was assessed by unpaired, two-tailed, Student’s t-test. The values with different letters are significantly different at 54 hrs (p<0.01).
