# Supplementary material for: Interaction of Temperature and Photoperiod Increases Growth and Oil Content in the Marine Microalgae Dunaliella viridis
Source: PLoS One. 2015 May 19;10(5):e0127562. doi: 10.1371/journal.pone.0127562 (PMC4437649; doi:10.1371/journal.pone.0127562)
Supplement: S3 Fig — (PPTX) [file pone.0127562.s003.pptx]

## Slide 1
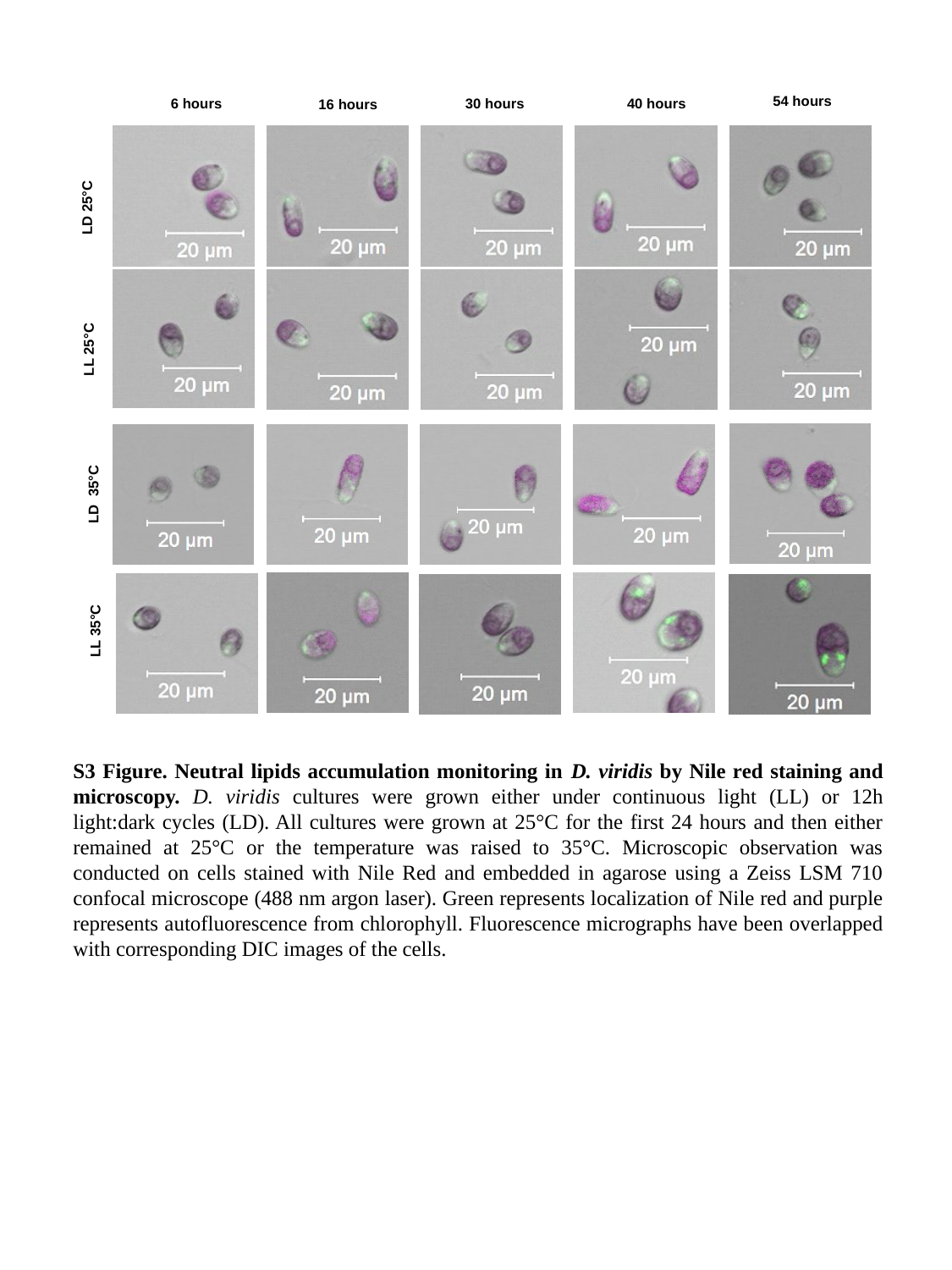

54 hours
6 hours
30 hours
40 hours
16 hours
LD 25°C
LL 25°C
LD 35°C
LL 35°C
S3 Figure. Neutral lipids accumulation monitoring in D. viridis by Nile red staining and microscopy. D. viridis cultures were grown either under continuous light (LL) or 12h light:dark cycles (LD). All cultures were grown at 25°C for the first 24 hours and then either remained at 25°C or the temperature was raised to 35°C. Microscopic observation was conducted on cells stained with Nile Red and embedded in agarose using a Zeiss LSM 710 confocal microscope (488 nm argon laser). Green represents localization of Nile red and purple represents autofluorescence from chlorophyll. Fluorescence micrographs have been overlapped with corresponding DIC images of the cells.
