# Supplementary material for: Interaction of Temperature and Photoperiod Increases Growth and Oil Content in the Marine Microalgae Dunaliella viridis
Source: PLoS One. 2015 May 19;10(5):e0127562. doi: 10.1371/journal.pone.0127562 (PMC4437649; doi:10.1371/journal.pone.0127562)
Supplement: S4 Fig — (PPTX) [file pone.0127562.s004.pptx]

## Slide 1
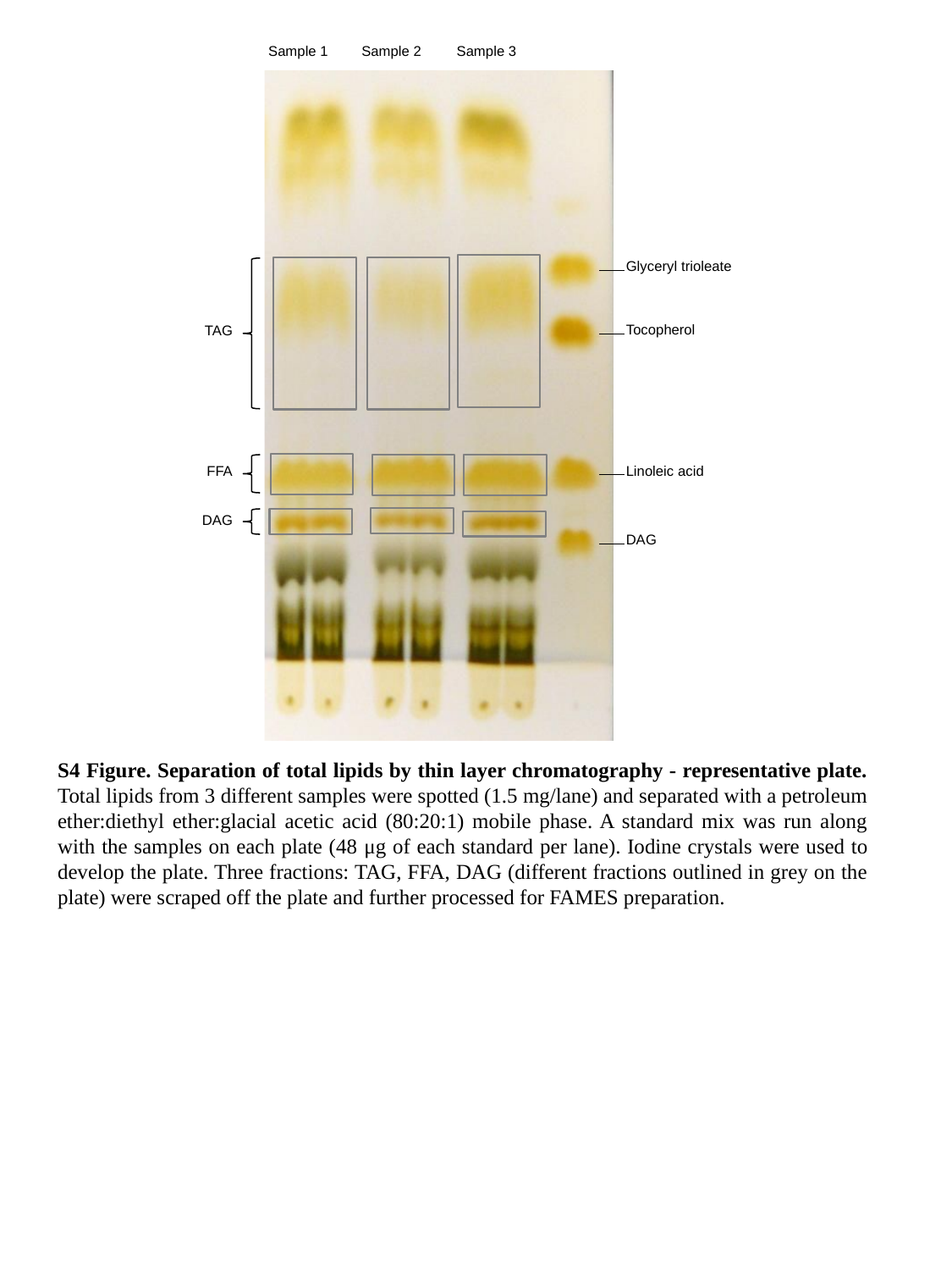

Sample 2
Sample 3
Sample 1
Glyceryl trioleate
Tocopherol
TAG
FFA
Linoleic acid
DAG
DAG
S4 Figure. Separation of total lipids by thin layer chromatography - representative plate. Total lipids from 3 different samples were spotted (1.5 mg/lane) and separated with a petroleum ether:diethyl ether:glacial acetic acid (80:20:1) mobile phase. A standard mix was run along with the samples on each plate (48 μg of each standard per lane). Iodine crystals were used to develop the plate. Three fractions: TAG, FFA, DAG (different fractions outlined in grey on the plate) were scraped off the plate and further processed for FAMES preparation.
