# Supplementary material for: Interaction of Temperature and Photoperiod Increases Growth and Oil Content in the Marine Microalgae Dunaliella viridis
Source: PLoS One. 2015 May 19;10(5):e0127562. doi: 10.1371/journal.pone.0127562 (PMC4437649; doi:10.1371/journal.pone.0127562)
Supplement: S5 Fig — (PPTX) [file pone.0127562.s005.pptx]

## Slide 1
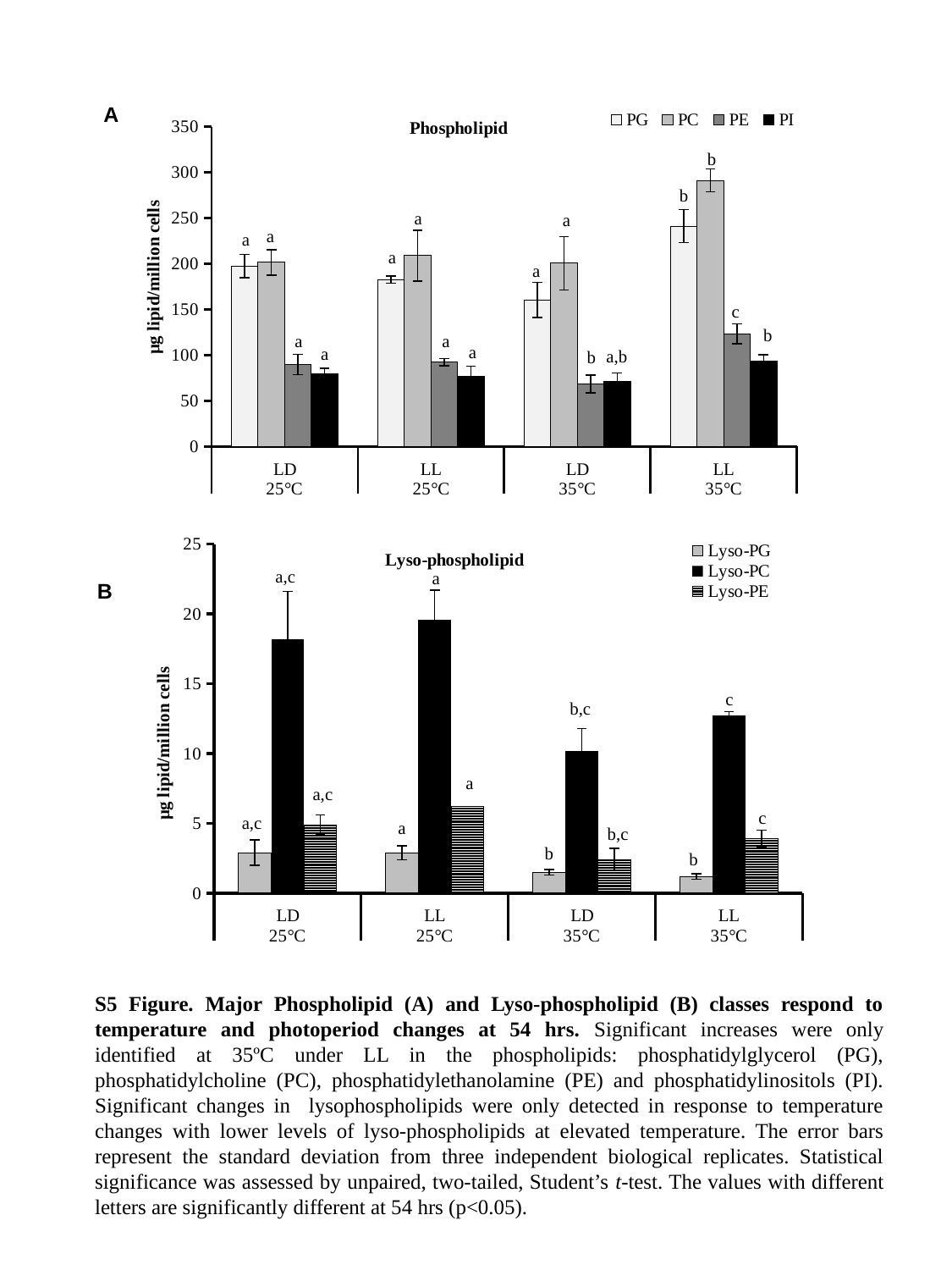

A
### Chart: Phospholipid
| Category | | | | |
|---|---|---|---|---|
| LD | 197.6 | 201.6 | 89.8 | 80.0 |
| LL | 182.7 | 208.9 | 92.2 | 76.6 |
| LD | 160.4 | 200.7 | 68.5 | 71.3 |
| LL | 241.2 | 291.3 | 123.4 | 93.3 |b
b
a
a
a
a
a
a
c
b
a
a
a
a
a,b
b
### Chart: Lyso-phospholipid
| Category | | | |
|---|---|---|---|
| LD | 2.9 | 18.2 | 4.9 |
| LL | 2.9 | 19.6 | 6.2 |
| LD | 1.5 | 10.200000000000001 | 2.4 |
| LL | 1.2 | 12.7 | 3.9 |a,c
a
B
c
b,c
a
a,c
c
a,c
a
b,c
b
b
S5 Figure. Major Phospholipid (A) and Lyso-phospholipid (B) classes respond to temperature and photoperiod changes at 54 hrs. Significant increases were only identified at 35ºC under LL in the phospholipids: phosphatidylglycerol (PG), phosphatidylcholine (PC), phosphatidylethanolamine (PE) and phosphatidylinositols (PI). Significant changes in lysophospholipids were only detected in response to temperature changes with lower levels of lyso-phospholipids at elevated temperature. The error bars represent the standard deviation from three independent biological replicates. Statistical significance was assessed by unpaired, two-tailed, Student’s t-test. The values with different letters are significantly different at 54 hrs (p<0.05).
