# Supplementary material for: Interaction of Temperature and Photoperiod Increases Growth and Oil Content in the Marine Microalgae Dunaliella viridis
Source: PLoS One. 2015 May 19;10(5):e0127562. doi: 10.1371/journal.pone.0127562 (PMC4437649; doi:10.1371/journal.pone.0127562)
Supplement: S6 Fig — (PPTX) [file pone.0127562.s006.pptx]

## Slide 1
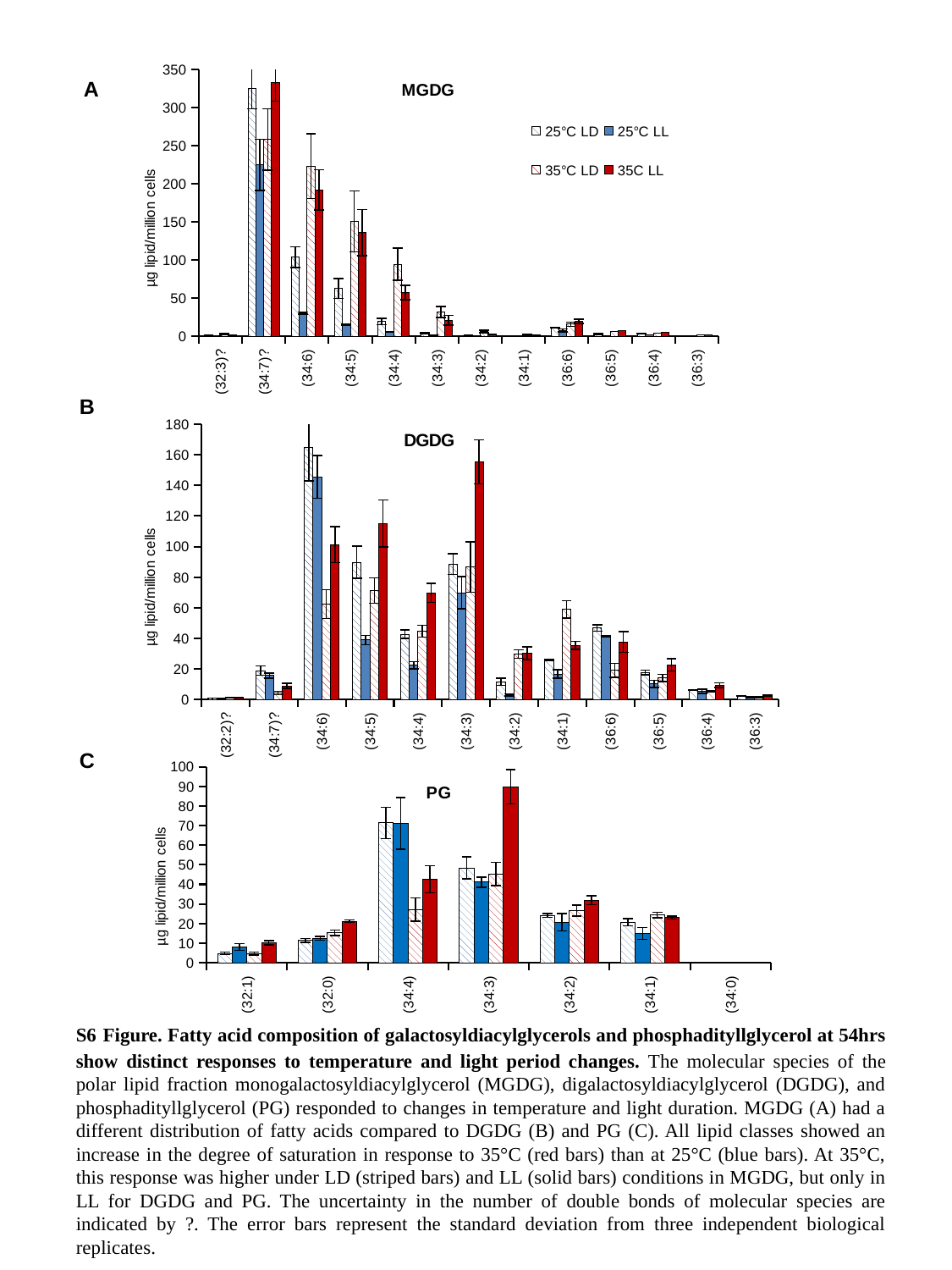

### Chart: MGDG
| Category | 25°C LD | 25°C LL | 35°C LD | 35C LL |
|---|---|---|---|---|
| (32:3)? | 0.8 | 0.3 | 2.6 | 1.6 |
| (34:7)? | 325.1 | 224.6 | 257.9 | 332.7 |
| (34:6) | 103.6 | 30.4 | 223.0 | 191.9 |
| (34:5) | 62.5 | 15.3 | 150.6 | 135.8 |
| (34:4) | 19.2 | 5.7 | 94.4 | 57.3 |
| (34:3) | 3.8 | 1.4 | 32.0 | 21.0 |
| (34:2) | 0.8 | 0.3 | 6.2 | 2.5 |
| (34:1) | 0.3 | 0.2 | 1.8 | 1.3 |
| (36:6) | 11.5 | 7.6 | 15.7 | 19.6 |
| (36:5) | 3.4 | 1.0 | 6.7 | 7.3 |
| (36:4) | 3.1 | 1.4 | 4.5 | 5.4 |
| (36:3) | 0.7 | 0.2 | 1.4 | 1.5 |A
B
### Chart: DGDG
| Category | 25°C | 25°C | 35°C | 35°C |
|---|---|---|---|---|
| (32:2)? | 1.0 | 0.5 | 1.1 | 1.3 |
| (34:7)? | 18.9 | 15.7 | 4.3 | 9.0 |
| (34:6) | 164.8 | 145.6 | 62.4 | 101.3 |
| (34:5) | 89.7 | 39.0 | 71.3 | 115.1 |
| (34:4) | 42.7 | 22.4 | 44.8 | 69.7 |
| (34:3) | 88.6 | 69.8 | 86.6 | 155.3 |
| (34:2) | 11.6 | 3.0 | 29.7 | 30.4 |
| (34:1) | 26.0 | 16.7 | 59.0 | 35.4 |
| (36:6) | 46.7 | 41.3 | 19.1 | 37.6 |
| (36:5) | 17.8 | 10.2 | 14.2 | 22.6 |
| (36:4) | 6.1 | 5.5 | 5.5 | 9.5 |
| (36:3) | 2.2 | 1.4 | 1.6 | 2.7 |C
### Chart: PG
| Category | 25°C | 25°C | 35°C | 35C |
|---|---|---|---|---|
| (32:1) | 4.8 | 8.1 | 4.7 | 10.2 |
| (32:0) | 11.4 | 12.4 | 15.2 | 21.2 |
| (34:4) | 71.4 | 71.1 | 27.1 | 42.6 |
| (34:3) | 48.4 | 41.1 | 45.3 | 89.8 |
| (34:2) | 24.1 | 20.6 | 26.7 | 31.9 |
| (34:1) | 20.7 | 15.0 | 24.3 | 23.0 |
| (34:0) | 0.0 | 0.0 | 0.0 | 0.0 |S6 Figure. Fatty acid composition of galactosyldiacylglycerols and phosphadityllglycerol at 54hrs show distinct responses to temperature and light period changes. The molecular species of the polar lipid fraction monogalactosyldiacylglycerol (MGDG), digalactosyldiacylglycerol (DGDG), and phosphadityllglycerol (PG) responded to changes in temperature and light duration. MGDG (A) had a different distribution of fatty acids compared to DGDG (B) and PG (C). All lipid classes showed an increase in the degree of saturation in response to 35°C (red bars) than at 25°C (blue bars). At 35°C, this response was higher under LD (striped bars) and LL (solid bars) conditions in MGDG, but only in LL for DGDG and PG. The uncertainty in the number of double bonds of molecular species are indicated by ?. The error bars represent the standard deviation from three independent biological replicates.
