# Supplementary material for: Interaction of Temperature and Photoperiod Increases Growth and Oil Content in the Marine Microalgae Dunaliella viridis
Source: PLoS One. 2015 May 19;10(5):e0127562. doi: 10.1371/journal.pone.0127562 (PMC4437649; doi:10.1371/journal.pone.0127562)
Supplement: S7 Fig — (PPTX) [file pone.0127562.s007.pptx]

## Slide 1
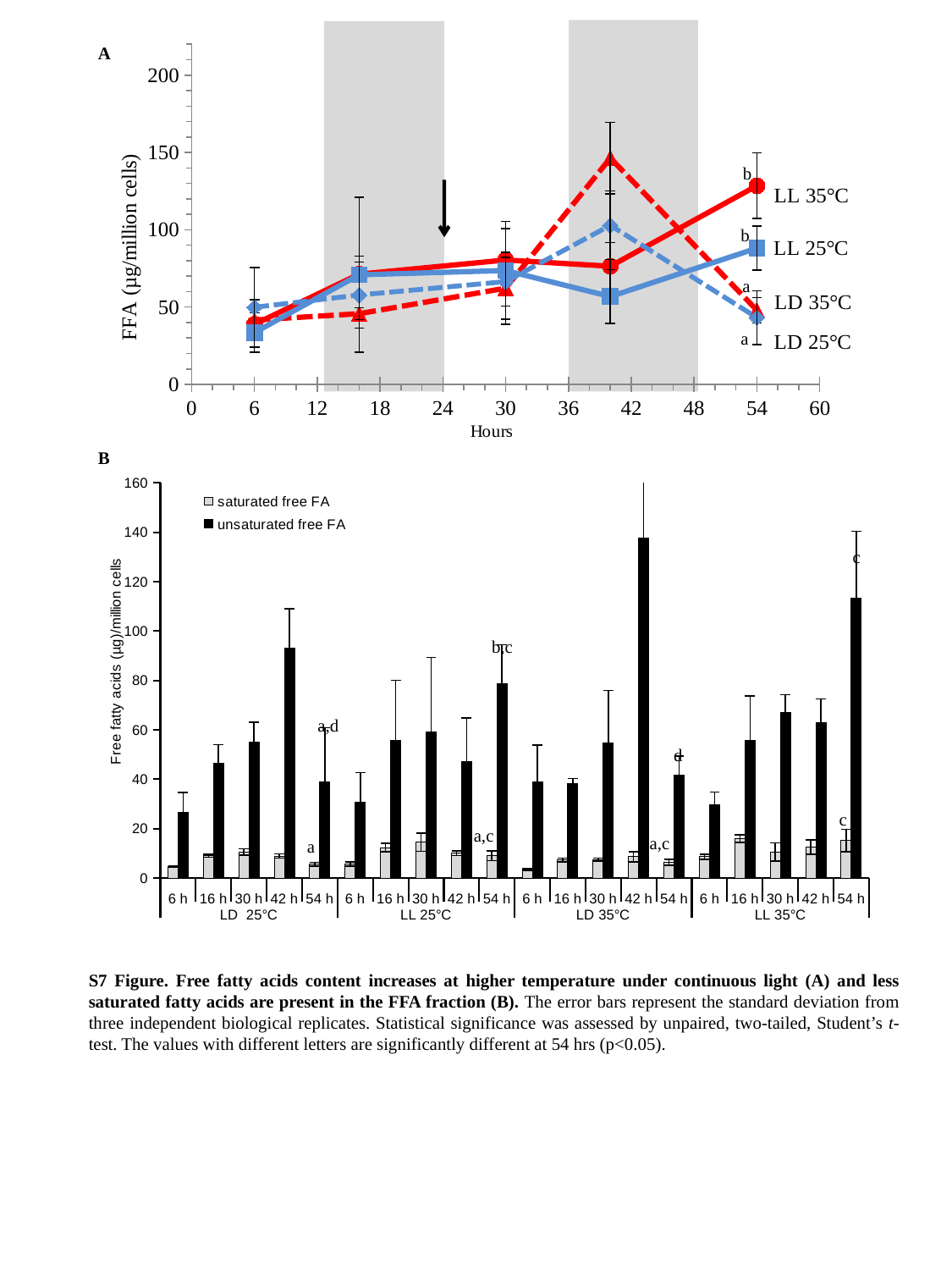

### Chart
| Category | LD 25°C | LL 25°C | LD 35°C | LL 35°C |
|---|---|---|---|---|
A
B
### Chart
| Category | | |
|---|---|---|
| 6 h | 4.581422301506269 | 26.632497407692195 |
| 16 h | 8.935845286356823 | 46.30488030644674 |
| 30 h | 10.598398293972167 | 55.04684523029363 |
| 42 h | 8.849335917760019 | 92.93518221945126 |
| 54 h | 5.525929783464572 | 39.008542190944915 |
| 6 h | 5.6771773424056144 | 30.771204543459174 |
| 16 h | 12.278377932308969 | 55.827685894933545 |
| 30 h | 14.511309407042669 | 59.18728088331836 |
| 42 h | 9.996040613708702 | 47.13089196817625 |
| 54 h | 8.95126341519713 | 78.74564243727598 |
| 6 h | 3.418772033047734 | 38.79347920848633 |
| 16 h | 7.246415931534883 | 38.23977757618604 |
| 30 h | 7.428839431607631 | 54.79474696021797 |
| 42 h | 8.581807929411763 | 137.5276011503268 |
| 54 h | 6.269203056657224 | 41.589687535410725 |
| 6 h | 8.586610238875876 | 29.503534238875865 |
| 16 h | 15.883755306445398 | 55.8219601968352 |
| 30 h | 10.524470392945787 | 66.9447853193991 |
| 42 h | 12.558579158169945 | 62.947058903050106 |
| 54 h | 15.128929749681518 | 113.32328078980892 |S7 Figure. Free fatty acids content increases at higher temperature under continuous light (A) and less saturated fatty acids are present in the FFA fraction (B). The error bars represent the standard deviation from three independent biological replicates. Statistical significance was assessed by unpaired, two-tailed, Student’s t-test. The values with different letters are significantly different at 54 hrs (p<0.05).
b
b
a
a
c
b,c
a,d
d
c
a,c
a,c
a
