# Supplementary material for: Interaction of Temperature and Photoperiod Increases Growth and Oil Content in the Marine Microalgae Dunaliella viridis
Source: PLoS One. 2015 May 19;10(5):e0127562. doi: 10.1371/journal.pone.0127562 (PMC4437649; doi:10.1371/journal.pone.0127562)
Supplement: S8 Fig — (PPTX) [file pone.0127562.s008.pptx]

## Slide 1
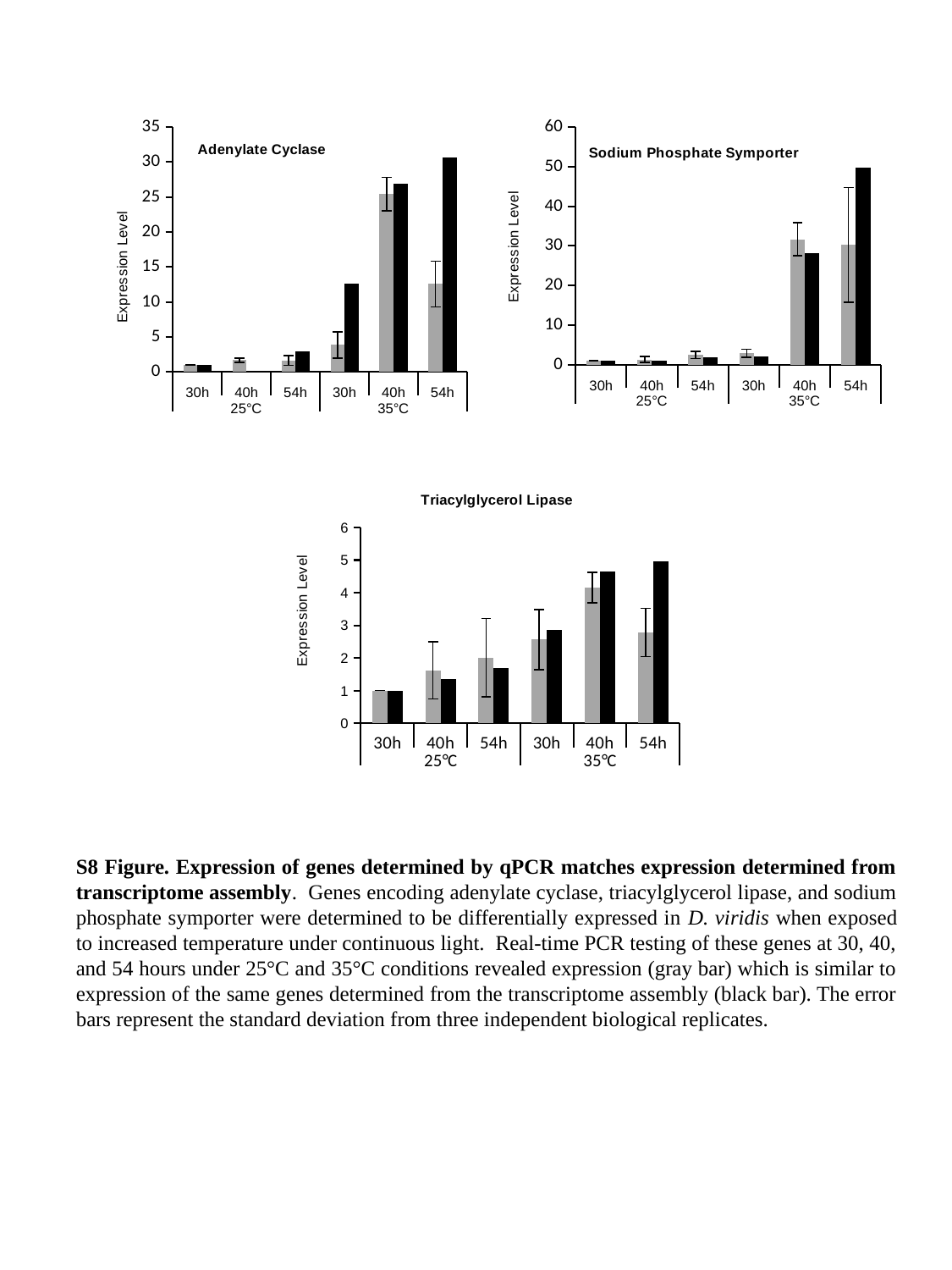

### Chart: Adenylate Cyclase
| Category | Adenylate Cyclase Real Time | Adenylate Cyclase RNA Seq |
|---|---|---|
| 30h | 1.0 | 1.0 |
| 40h | 1.6684053 | 0.0 |
| 54h | 1.6334692999999996 | 2.991399503 |
| 30h | 3.846174 | 12.64515573 |
| 40h | 25.39699299999999 | 26.96041516 |
| 54h | 12.556023 | 30.6889845 |
### Chart: Sodium Phosphate Symporter
| Category | Sodium Phosphate Symporter (15245) Real Time | Sodium Phosphate Symporter (15245) RNA Seq |
|---|---|---|
| 30h | 1.0 | 1.0 |
| 40h | 1.2930352999999997 | 1.0849291759999995 |
| 54h | 2.4375763 | 1.810677389 |
| 30h | 2.8126882999999983 | 2.099424386 |
| 40h | 31.655246999999992 | 28.05876224999999 |
| 54h | 30.26829999999999 | 49.842467299999996 |
### Chart: Triacylglycerol Lipase
| Category | Triacylglycerol Lipase Real Time | Triacylglycerol Lipase RNA Seq |
|---|---|---|
| 30h | 1.0 | 1.0 |
| 40h | 1.6161994 | 1.3637943059999993 |
| 54h | 2.0095964 | 1.6795716369999998 |
| 30h | 2.5610798 | 2.852236851 |
| 40h | 4.156142599999998 | 4.649995356999996 |
| 54h | 2.7796206 | 4.95239613 |S8 Figure. Expression of genes determined by qPCR matches expression determined from transcriptome assembly. Genes encoding adenylate cyclase, triacylglycerol lipase, and sodium phosphate symporter were determined to be differentially expressed in D. viridis when exposed to increased temperature under continuous light. Real-time PCR testing of these genes at 30, 40, and 54 hours under 25°C and 35°C conditions revealed expression (gray bar) which is similar to expression of the same genes determined from the transcriptome assembly (black bar). The error bars represent the standard deviation from three independent biological replicates.
