# Supplementary material for: Interaction of Temperature and Photoperiod Increases Growth and Oil Content in the Marine Microalgae Dunaliella viridis
Source: PLoS One. 2015 May 19;10(5):e0127562. doi: 10.1371/journal.pone.0127562 (PMC4437649; doi:10.1371/journal.pone.0127562)
Supplement: S9 Fig — (PPTX) [file pone.0127562.s009.pptx]

## Slide 1
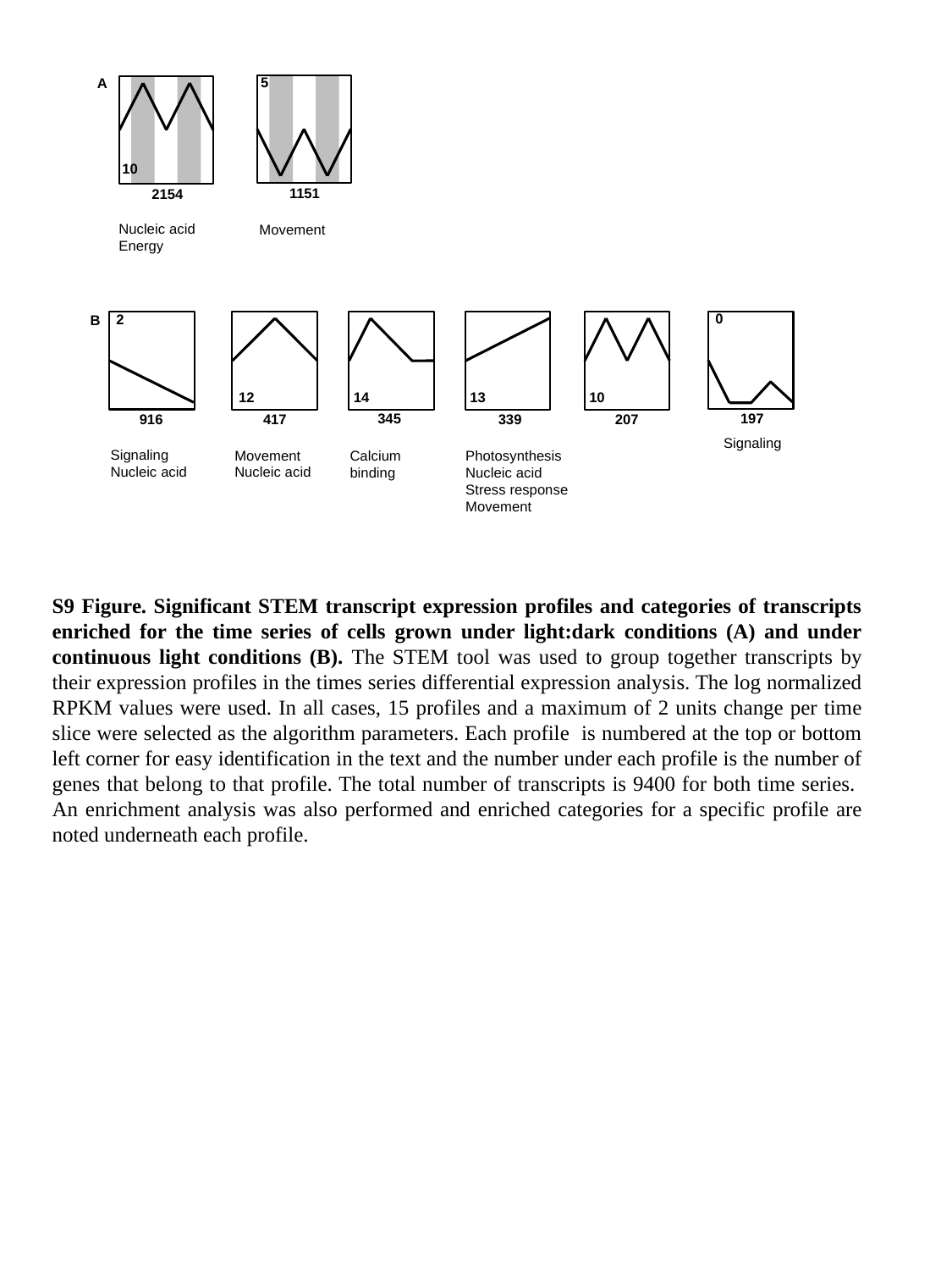

5
1151
Movement
A
10
2154
Nucleic acid
Energy
0
197
Signaling
2
916
Signaling
Nucleic acid
B
12
417
Movement
Nucleic acid
14
345
Calcium binding
13
339
Photosynthesis
Nucleic acid
Stress response
Movement
10
207
S9 Figure. Significant STEM transcript expression profiles and categories of transcripts enriched for the time series of cells grown under light:dark conditions (A) and under continuous light conditions (B). The STEM tool was used to group together transcripts by their expression profiles in the times series differential expression analysis. The log normalized RPKM values were used. In all cases, 15 profiles and a maximum of 2 units change per time slice were selected as the algorithm parameters. Each profile is numbered at the top or bottom left corner for easy identification in the text and the number under each profile is the number of genes that belong to that profile. The total number of transcripts is 9400 for both time series. An enrichment analysis was also performed and enriched categories for a specific profile are noted underneath each profile.
