# Supplementary material for: Interaction of Temperature and Photoperiod Increases Growth and Oil Content in the Marine Microalgae Dunaliella viridis
Source: PLoS One. 2015 May 19;10(5):e0127562. doi: 10.1371/journal.pone.0127562 (PMC4437649; doi:10.1371/journal.pone.0127562)
Supplement: S10 Fig — (PPTX) [file pone.0127562.s010.pptx]

## Slide 1
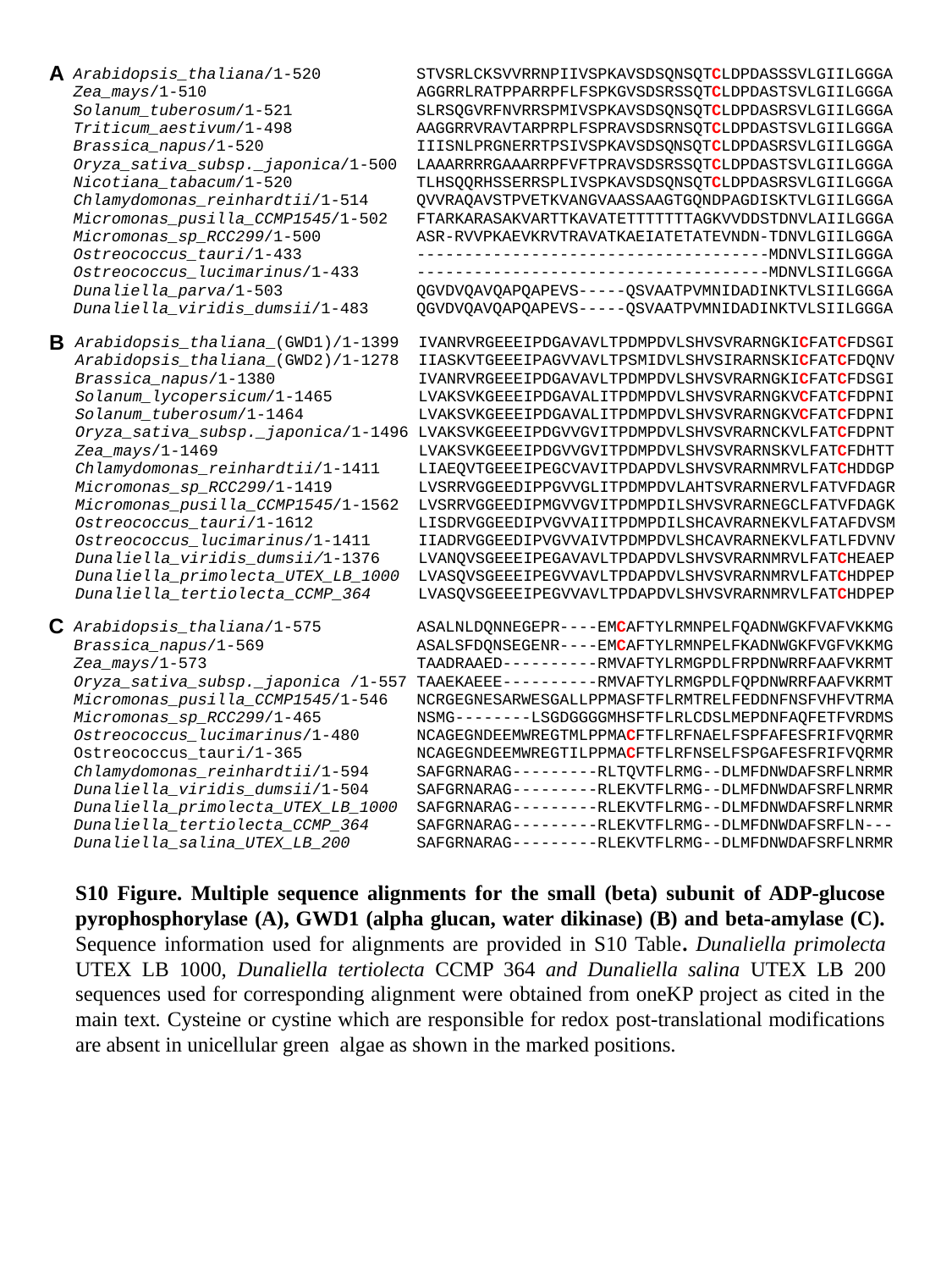

A
B
C
S10 Figure. Multiple sequence alignments for the small (beta) subunit of ADP-glucose pyrophosphorylase (A), GWD1 (alpha glucan, water dikinase) (B) and beta-amylase (C). Sequence information used for alignments are provided in S10 Table. Dunaliella primolecta UTEX LB 1000, Dunaliella tertiolecta CCMP 364 and Dunaliella salina UTEX LB 200 sequences used for corresponding alignment were obtained from oneKP project as cited in the main text. Cysteine or cystine which are responsible for redox post-translational modifications are absent in unicellular green algae as shown in the marked positions.
