# Supplementary material for: Interaction of Temperature and Photoperiod Increases Growth and Oil Content in the Marine Microalgae Dunaliella viridis
Source: PLoS One. 2015 May 19;10(5):e0127562. doi: 10.1371/journal.pone.0127562 (PMC4437649; doi:10.1371/journal.pone.0127562)
Supplement: S11 Fig — (PPTX) [file pone.0127562.s011.pptx]

## Slide 1
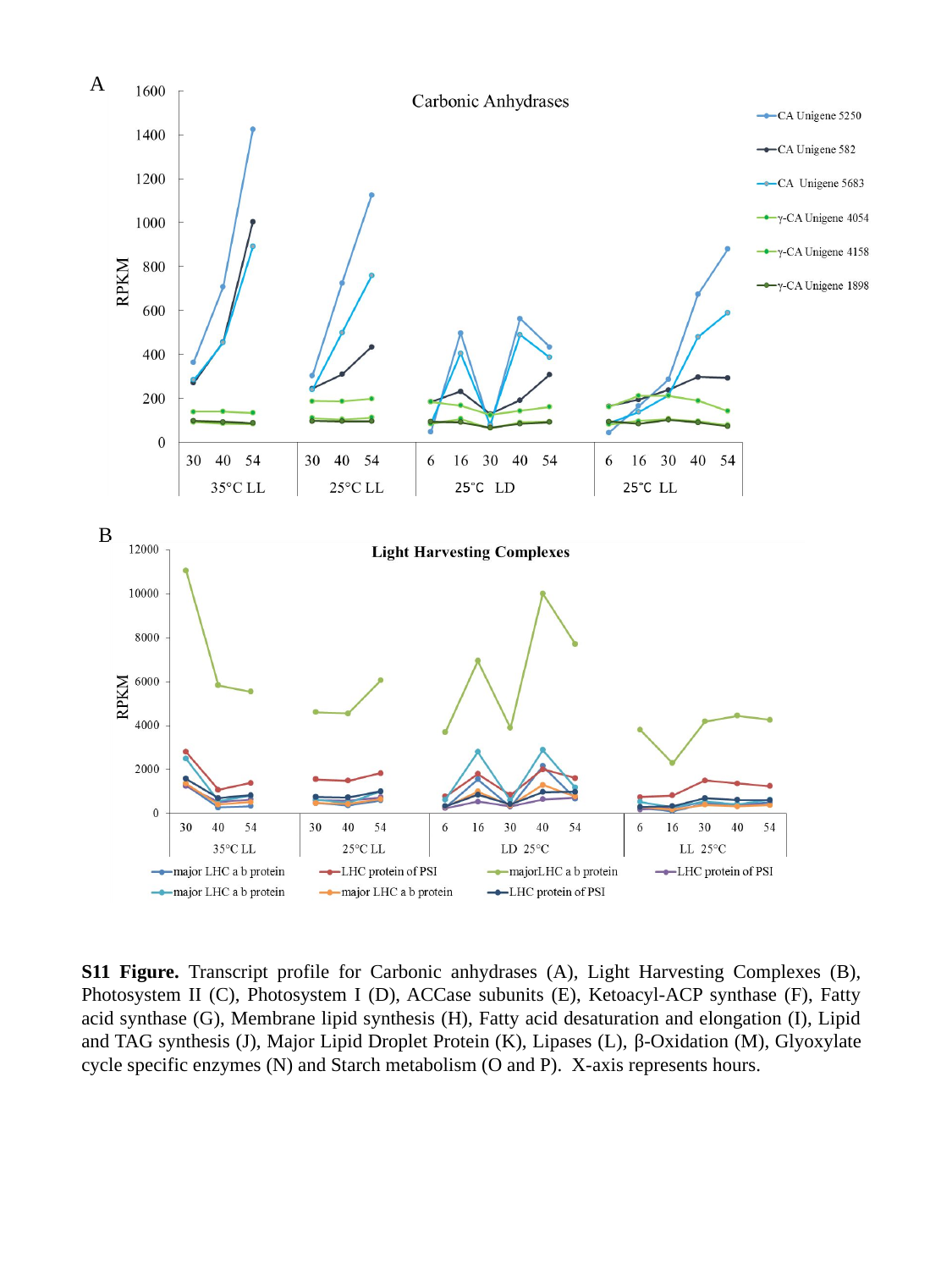

A
B
S11 Figure. Transcript profile for Carbonic anhydrases (A), Light Harvesting Complexes (B), Photosystem II (C), Photosystem I (D), ACCase subunits (E), Ketoacyl-ACP synthase (F), Fatty acid synthase (G), Membrane lipid synthesis (H), Fatty acid desaturation and elongation (I), Lipid and TAG synthesis (J), Major Lipid Droplet Protein (K), Lipases (L), β-Oxidation (M), Glyoxylate cycle specific enzymes (N) and Starch metabolism (O and P). X-axis represents hours.

## Slide 2
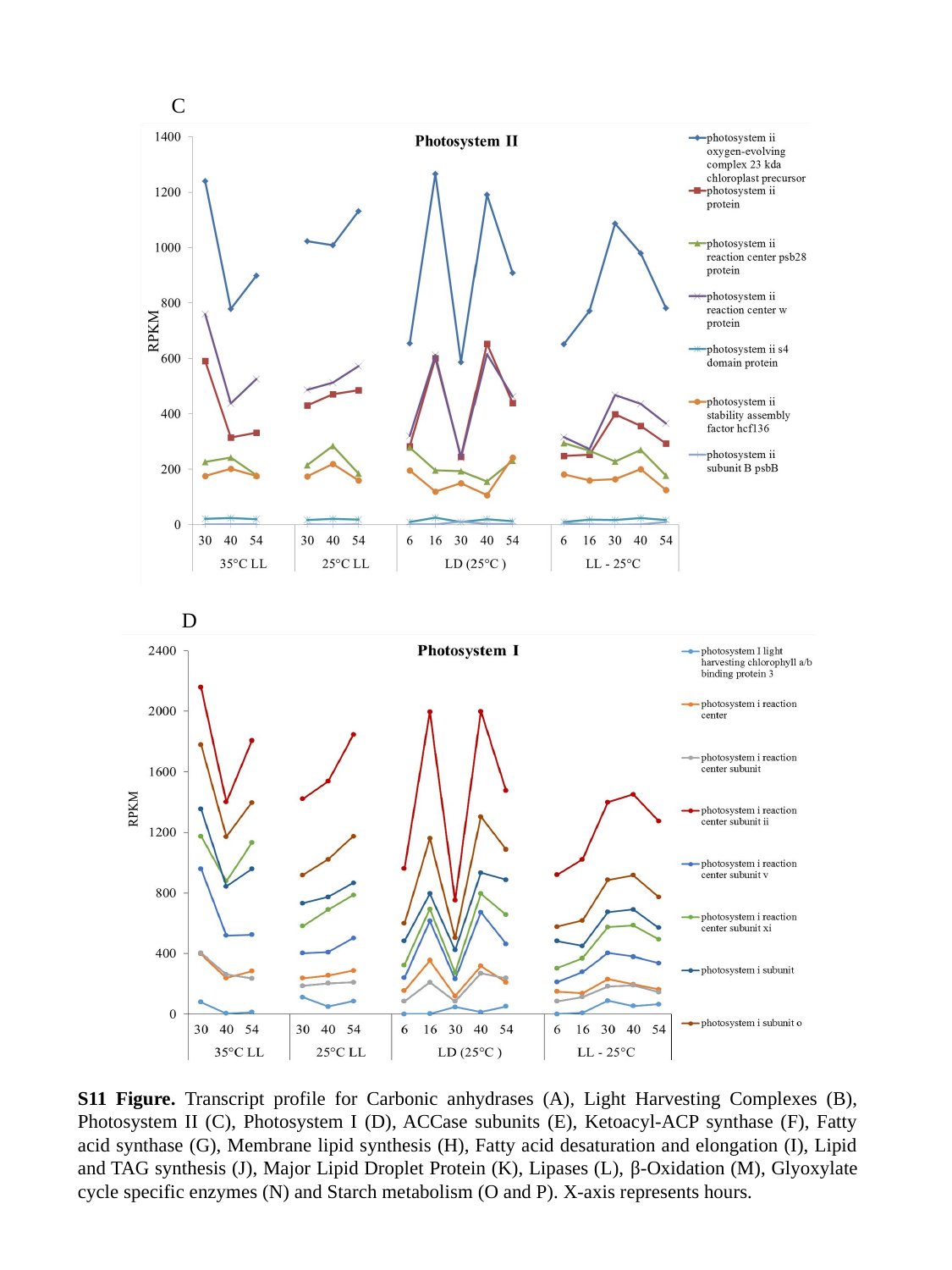

C
D
S11 Figure. Transcript profile for Carbonic anhydrases (A), Light Harvesting Complexes (B), Photosystem II (C), Photosystem I (D), ACCase subunits (E), Ketoacyl-ACP synthase (F), Fatty acid synthase (G), Membrane lipid synthesis (H), Fatty acid desaturation and elongation (I), Lipid and TAG synthesis (J), Major Lipid Droplet Protein (K), Lipases (L), β-Oxidation (M), Glyoxylate cycle specific enzymes (N) and Starch metabolism (O and P). X-axis represents hours.

## Slide 3
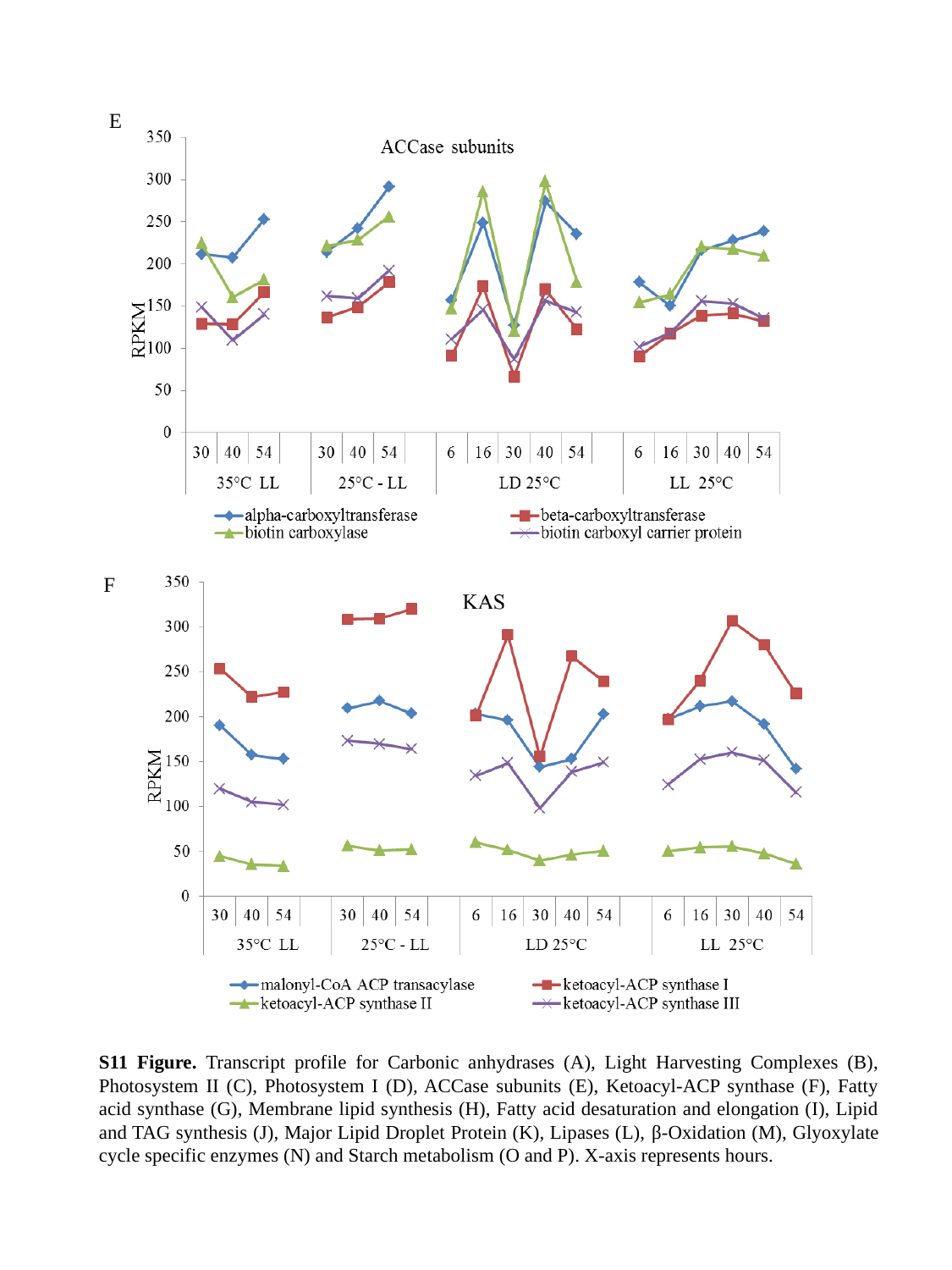

E
F
S11 Figure. Transcript profile for Carbonic anhydrases (A), Light Harvesting Complexes (B), Photosystem II (C), Photosystem I (D), ACCase subunits (E), Ketoacyl-ACP synthase (F), Fatty acid synthase (G), Membrane lipid synthesis (H), Fatty acid desaturation and elongation (I), Lipid and TAG synthesis (J), Major Lipid Droplet Protein (K), Lipases (L), β-Oxidation (M), Glyoxylate cycle specific enzymes (N) and Starch metabolism (O and P). X-axis represents hours.

## Slide 4
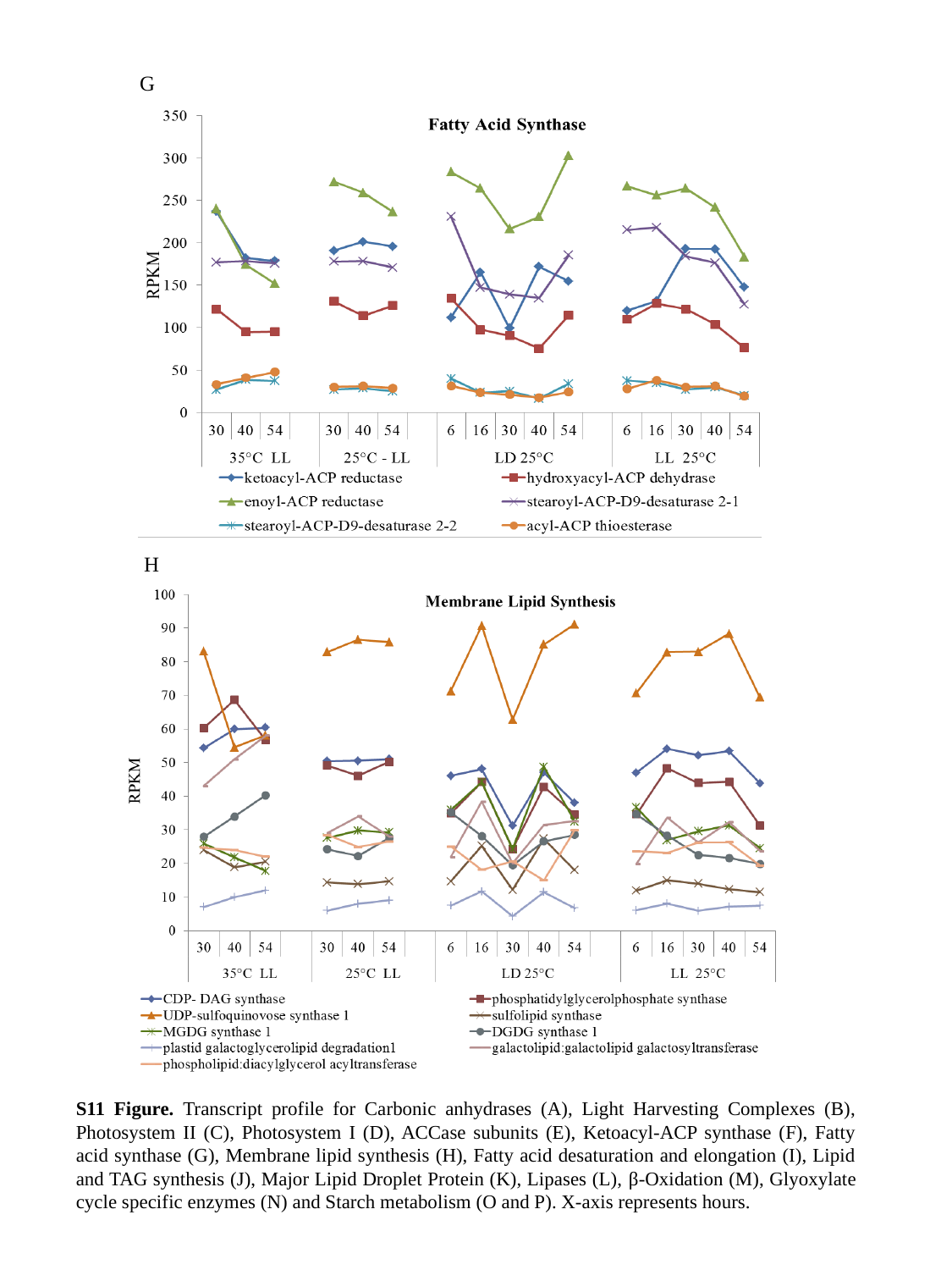

G
H
S11 Figure. Transcript profile for Carbonic anhydrases (A), Light Harvesting Complexes (B), Photosystem II (C), Photosystem I (D), ACCase subunits (E), Ketoacyl-ACP synthase (F), Fatty acid synthase (G), Membrane lipid synthesis (H), Fatty acid desaturation and elongation (I), Lipid and TAG synthesis (J), Major Lipid Droplet Protein (K), Lipases (L), β-Oxidation (M), Glyoxylate cycle specific enzymes (N) and Starch metabolism (O and P). X-axis represents hours.

## Slide 5
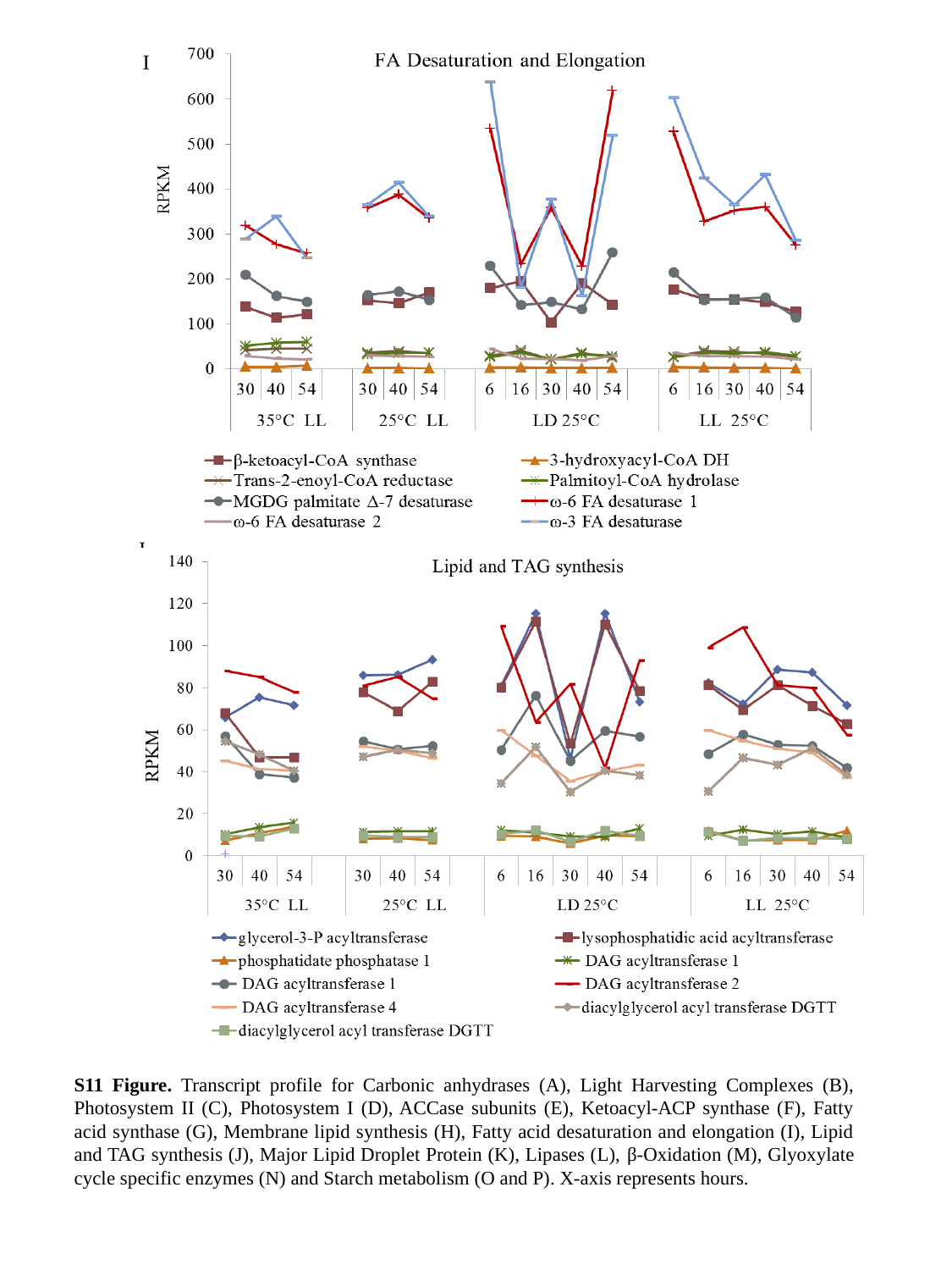

I
J
S11 Figure. Transcript profile for Carbonic anhydrases (A), Light Harvesting Complexes (B), Photosystem II (C), Photosystem I (D), ACCase subunits (E), Ketoacyl-ACP synthase (F), Fatty acid synthase (G), Membrane lipid synthesis (H), Fatty acid desaturation and elongation (I), Lipid and TAG synthesis (J), Major Lipid Droplet Protein (K), Lipases (L), β-Oxidation (M), Glyoxylate cycle specific enzymes (N) and Starch metabolism (O and P). X-axis represents hours.

## Slide 6
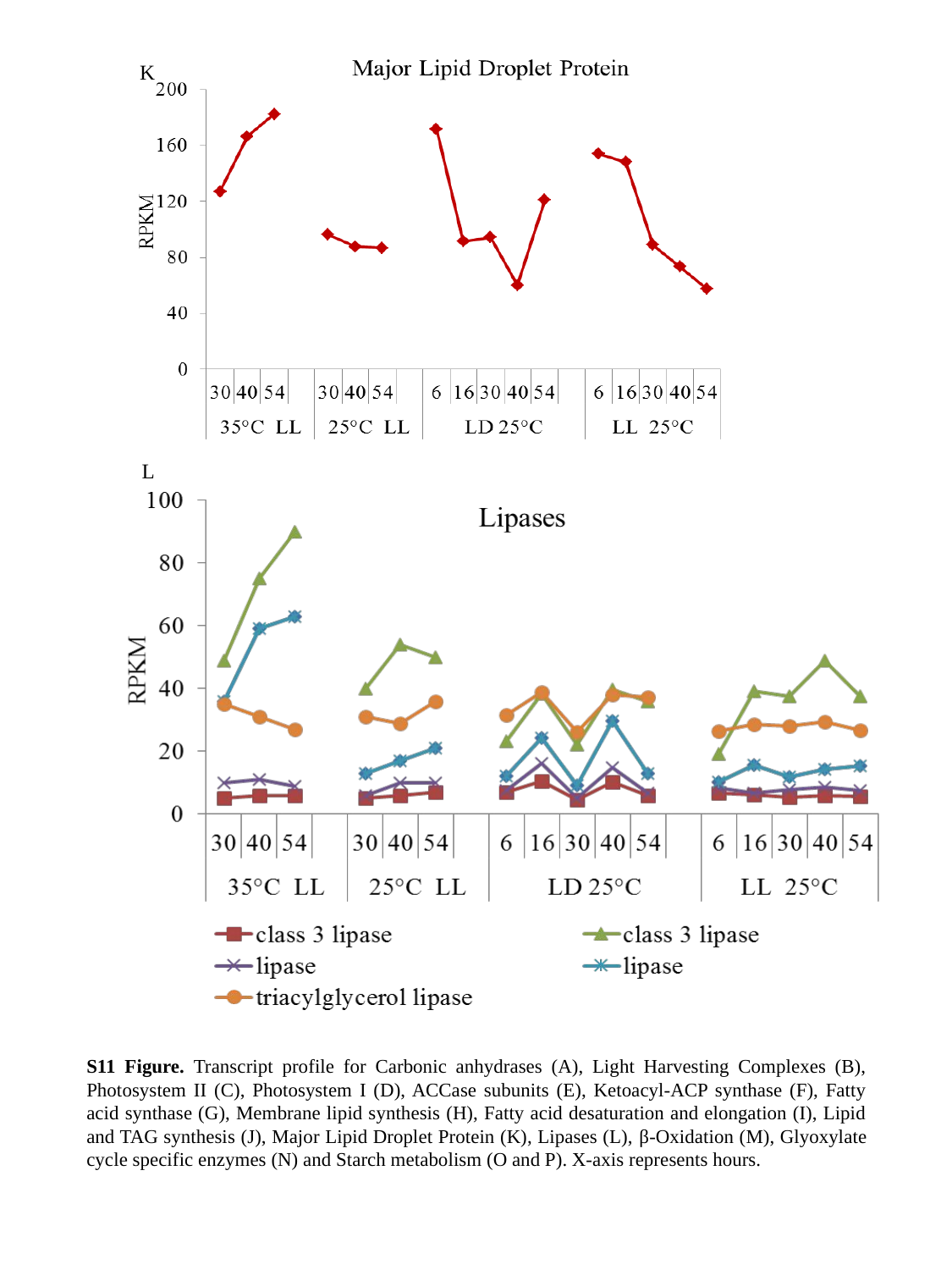

K
L
S11 Figure. Transcript profile for Carbonic anhydrases (A), Light Harvesting Complexes (B), Photosystem II (C), Photosystem I (D), ACCase subunits (E), Ketoacyl-ACP synthase (F), Fatty acid synthase (G), Membrane lipid synthesis (H), Fatty acid desaturation and elongation (I), Lipid and TAG synthesis (J), Major Lipid Droplet Protein (K), Lipases (L), β-Oxidation (M), Glyoxylate cycle specific enzymes (N) and Starch metabolism (O and P). X-axis represents hours.

## Slide 7
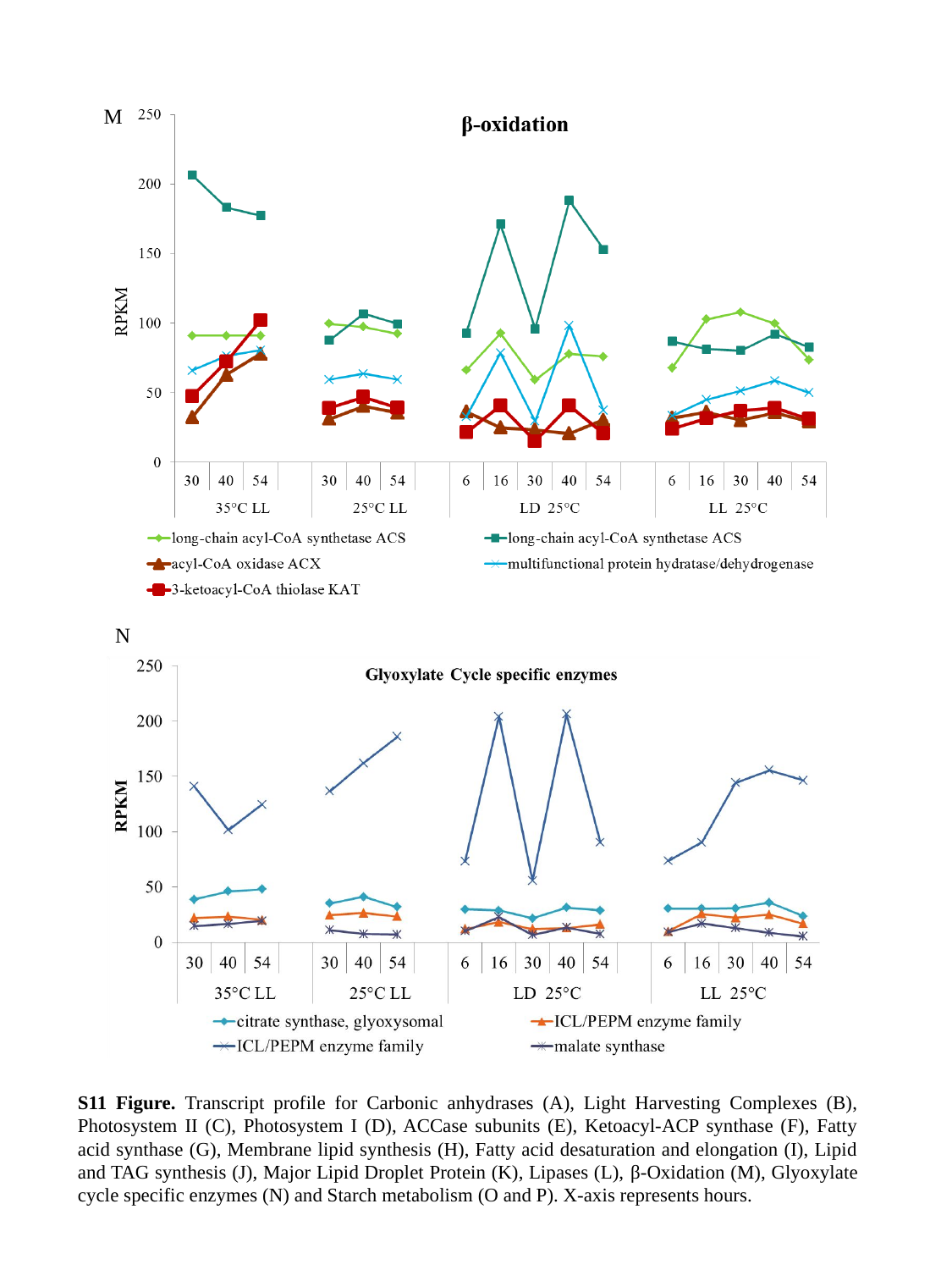

M
N
S11 Figure. Transcript profile for Carbonic anhydrases (A), Light Harvesting Complexes (B), Photosystem II (C), Photosystem I (D), ACCase subunits (E), Ketoacyl-ACP synthase (F), Fatty acid synthase (G), Membrane lipid synthesis (H), Fatty acid desaturation and elongation (I), Lipid and TAG synthesis (J), Major Lipid Droplet Protein (K), Lipases (L), β-Oxidation (M), Glyoxylate cycle specific enzymes (N) and Starch metabolism (O and P). X-axis represents hours.

## Slide 8
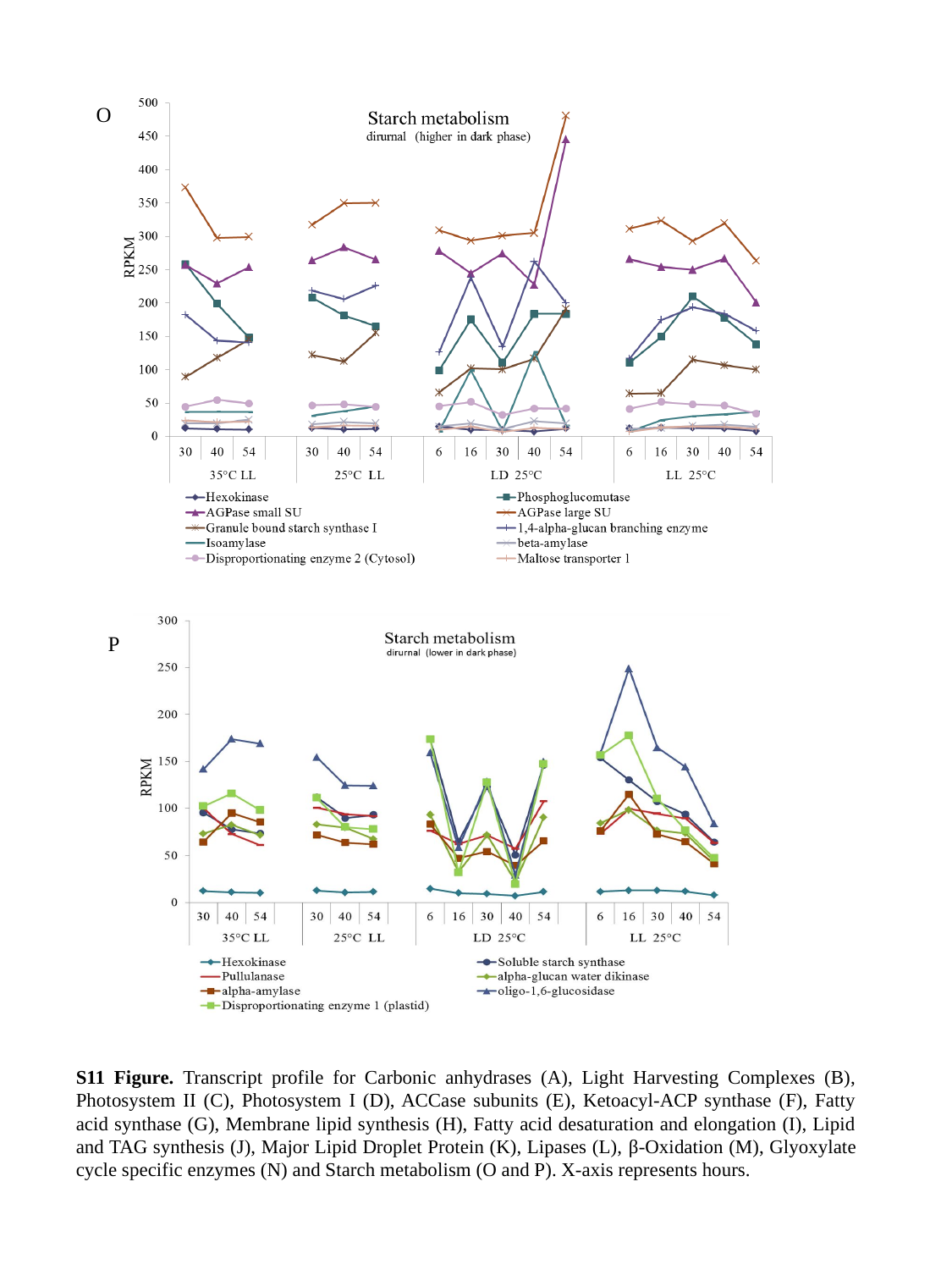

O
P
S11 Figure. Transcript profile for Carbonic anhydrases (A), Light Harvesting Complexes (B), Photosystem II (C), Photosystem I (D), ACCase subunits (E), Ketoacyl-ACP synthase (F), Fatty acid synthase (G), Membrane lipid synthesis (H), Fatty acid desaturation and elongation (I), Lipid and TAG synthesis (J), Major Lipid Droplet Protein (K), Lipases (L), β-Oxidation (M), Glyoxylate cycle specific enzymes (N) and Starch metabolism (O and P). X-axis represents hours.
