# Supplementary material for: Interaction of Temperature and Photoperiod Increases Growth and Oil Content in the Marine Microalgae Dunaliella viridis
Source: PLoS One. 2015 May 19;10(5):e0127562. doi: 10.1371/journal.pone.0127562 (PMC4437649; doi:10.1371/journal.pone.0127562)
Supplement: S1 Protocol — (DOCX) [file pone.0127562.s012.docx]

**S1 Protocol. Neutral lipids accumulation monitoring by Nile Red staining and microscopy**

To 1 mL of cells, Nile Red was added to a final concentration of 2.6 μM. After 5 min incubation, the cells stained with Nile Red were pelleted for 5 min at 5,000 rpm in a microcentrifuge. Between 950-970 μL of supernatant was removed depending on the initial cell concentration and the cells resuspended in the leftover supernatant. For microscopic observation, the cells were embedded in low gelling temperature agarose (type VII agarose, Sigma A4018) by mixing 4 μL of cells with 4 μL of melted 1.3% agarose prepared using growth media. Nile Red stained lipid globules were observed in the cells using a Zeiss LSM 710 confocal microscope. Images were produced using a 488 nm argon laser and captured in three emission channels. Nile red fluorescence, plastid autofluorescence, and bright field were detected with 527-621 nm, 681-735 nm, and DIC filters, respectively. Pinhole size was set to 45 μm – or 1 AU on the LD C-Apochromat 40x/1.1 W Korr M27 water-immersion objective.
