# Supplementary material for: Interaction of Temperature and Photoperiod Increases Growth and Oil Content in the Marine Microalgae Dunaliella viridis
Source: PLoS One. 2015 May 19;10(5):e0127562. doi: 10.1371/journal.pone.0127562 (PMC4437649; doi:10.1371/journal.pone.0127562)
Supplement: S2 Protocol — (DOCX) [file pone.0127562.s013.docx]

**S2 Protocol. Transcriptome analysis**

**RNA-Seq sample preparation and quality analysis of reads**

The Illumina ^®^TruSeq^TM^ RNA Sample Preparation v2 Kit was used to prepare the cDNA libraries for sequencing on the Illumina Genome Analyzer GA IIx platform. Bar-coded samples were sequenced in 2 separate sequencing runs using 100-nucleotide Illumina RNA-Seq single-end reads. In the first run, a single biological replicate was sequenced from each of the 12 combinations of time, light condition and temperature from time points 30, 40 and 54 hrs. The 12 uniquely barcoded samples were divided equally among 4 lanes resulting in 48 distinct sequencing data files. In the second run, a single biological replicate was sequenced from each of the 10 combinations of time and light condition from time points 6, 16 30, 40 and 54 hrs with temperature fixed at 25°C. The 10 uniquely barcoded samples were divided equally among 4 lanes resulting in 40 distinct sequencing data files. The quality of the reads was assessed using the FAST-X toolkit and the FastQC program. Low quality sequences were filtered using the quality trimmer tool from the FAST-X toolkit. After low quality sequences were filtered out (between 3-5%), the read length distribution changed minimally, with the majority of the reads retaining their full length.

#### **Transcriptome assembly and annotation**

Because no reference genome is available for *Dunaliella*, two different *de novo* assembly programs were tested: Velvet (1) and Oases (2) with parameter k=71. The input sequences for those 2 programs were our RNA-Seq reads along with 12 million 2x90 nucleotide Illumina paired-end reads that we had obtained from a previous sequencing effort obtained from oneKP sequencing consortium (3-5). Built as an additional layer on top of the Velvet genome assembler, Oases was designed specifically to accommodate the unique requirements of transcriptome assembly. Compared to the Velvet assembly, the Oases assembly showed a slight improvement in terms of overall contig count and size (S2 Table). On average, the Oases assembler produced fewer, yet longer contigs, and grouped together putative alternative “transcripts” at each locus. The median and mean number of Oases transcripts per locus were 1 and 1.28, respectively. The Oases-assembled transcripts were annotated using BLAST2GO software (6) performing a blastx search against the NCBI nr protein database using the following parameters: maximum number of blast hits = 20; e-value cutoff = 10^-6^; HSP length cutoff = 33; low complexity filter = on. As a result from the annotation step, 11,701 transcripts from 9,400 distinct loci had at least one high quality database match. So, a little less than half of the transcripts found using Oases were able to be annotated.

#### **Alignment**

The Bowtie program v 0.12.7 (7) was used to align the RNA-Seq reads from each sample to the assembled Oases transcripts. Subsequently, each of the cleaned and trimmed reads files were aligned to the index, using the “-a” option to return all alignments and not just the top N. The output alignments were sorted by read name, locus and position. Finally, a custom perl script was used to count the number of reads aligned to each locus. Only reads that aligned to a single locus were counted; ambiguous “multi-reads” were discarded (about 15% of the aligned reads). Reads that aligned to multiple transcripts from the same locus were counted only once. We also compiled a table containing per-transcript (rather than per locus) read counts. As in the previous case, only reads that mapped to a single locus were considered. At the transcript level, reads that mapped to multiple transcripts within a single locus were given fractional counts. On average, over 80% of the reads aligned to an assembled transcript.

#### **Differential expression**

For each barcoded sample, the per-locus counts were used to calculate the Reads Per Kilobase of exon model per Million mapped reads (8). For loci with multiple Oases transcripts, the average transcript length was used as the locus length. Only simple effects were tested using the linear models (“lm”) function in R. We tested for differential expression by comparing the log_2_ of the per-locus RPKM values (after adding a pseudo count of one to each sample). The model determines statistical significance by comparing the mean change in expression between conditions to the expression variability between sequencing lanes. A locus was considered differentially expressed if a) the FDR-adjusted p-value was ≤ 0.10 (using the Benjamini-Hochberg method (9); b) the absolute value of the log_2_ fold change was ≥ 1.0; and c) the total read count across all samples compared was at least 75. The temperature effect: “35°C over 25°C” was tested at 30, 40 and 54 hrs under continuous light and the light effect: “Continuous light over Light-Dark cycle” was tested at 6, 16 30, 40 and 54 hrs at constant temperature 25°C. Using the GO terms assigned by BLAST2GO, a GO term enrichment analysis was performed for the differentially expressed transcripts. The background set used was the set of all transcripts with assigned GO terms. The transcripts used were the ones which was found to be significantly differentially expressed on at least 2 distinct time points. The hypergeometric test for significance in the R “GOstats” package (10) was used to determine significance (we used the GOstats ‘conditional’ testing option which attempts to prevent multiple tests for hierarchically related GO terms). Only those GO terms with at least 3 assigned genes in the background set were tested. Benjamin-Hochberg FDR control was applied to adjust the resulting p-values.

**Expression profiles**

In order to look in more detail at the effect of LL on gene expression, expression profiles were generated allowing visualization of expression level at the different time points of the time course experiment. The STEM (Short Time-series Expression Miner) tool (11) was used to group together transcripts by their expression profiles in the times series differential expression analysis. In all cases, 15 profiles and a maximum of 2 units change per time slice were selected as the algorithm parameters. GO term enrichment was also tested in each of the significant patterns found.

**Fatty acid, triacylglycerol, and starch pathways analysis**

Those pathways were built mainly by searching in our transcriptome data for the genes known to be involved in those pathways from both *Chlamydomonas* and *Arabidopsis* (Figs. 9, 10 and 11, S7 Table). For the fatty acid and triacylglycerol pathways, the effect of temperature on the transcript abundance is studied and for the starch pathway, the effect of light duration on the transcript abundance is studied. For each pathway, the choice was made of a specific factor based on its contribution to the transcript abundance.

### **Quantitative Real-Time PCR**

First strand cDNA was synthesized from 1 μg of total DNase-treated (New England BioLabs^®^ RNase free DNase I, M0303S) RNA using the Invitrogen^TM^ SuperScript^®^ VILO^TM^ cDNA synthesis kit. In order to confirm the results from the RNA-sequencing data, expression of specific genes were tested by qPCR in reactions of 40 PCR cycles using gene-specific primers (S9 Table). The KAPA SYBR^®^ FAST qPCR Kit was used according to the manufacturer’s protocol with the tubulin gene as normalizer. The comparative Ct method was used to compare the relative expression of the treatment (growth at 35°C), with the 30 hrs time point at 25°C serving as time point zero in the comparison (S8 Figure).

**References**

1. Zerbino DR, Birney E. Velvet: Algorithms for de novo short read assembly using de bruijn graphs. Genome Research [Internet]. 2008 May;18(5):821-9.

2. Schulz MH, Zerbino DR, Vingron M, Birney E. Oases: Robust de novo RNA-seq assembly across the dynamic range of expression levels. Bioinformatics [Internet]. 2012 Apr 15;28(8):1086-92.

3. Johnson MT, Carpenter EJ, Tian Z, Bruskiewich R, Burris JN, Carrigan CT, Chase MW, Clarke ND, Covshoff S, Depamphilis CW, Edger PP, Goh F, Graham S, Greiner S, Hibberd JM, Jordon-Thaden I, Kutchan TM, Leebens-Mack J, Melkonian M, Miles N, Myburg H, Patterson J, Pires JC, Ralph P, Rolf M, Sage RF, Soltis D, Soltis P, Stevenson D, Stewart CN,Jr, Surek B, Thomsen CJ, Villarreal JC, Wu X, Zhang Y, Deyholos MK, Wong GK. Evaluating methods for isolating total RNA and predicting the success of sequencing phylogenetically diverse plant transcriptomes. PLoS One [Internet]. 2012;7(11):e50226.

4. Matasci N, Hung L, Yan Z, Carpenter EJ, Wickett NJ, Mirarab S, Nguyen N, Warnow T, Ayyampalayam S, Barker M. Data access for the 1,000 plants (1KP) project. GigaScience [Internet]. 2014;3(1):17.

5. Wickett NJ, Mirarab S, Nguyen N, Warnow T, Carpenter E, Matasci N, Ayyampalayam S, Barker MS, Burleigh JG, Gitzendanner MA, Ruhfel BR, Wafula E, Der JP, Graham SW, Mathews S, Melkonian M, Soltis DE, Soltis PS, Miles NW, Rothfels CJ, Pokorny L, Shaw AJ, DeGironimo L, Stevenson DW, Surek B, Villarreal JC, Roure B, Philippe H, dePamphilis CW, Chen T, Deyholos MK, Baucom RS, Kutchan TM, Augustin MM, Wang J, Zhang Y, Tian Z, Yan Z, Wu X, Sun X, Wong GK, Leebens-Mack J. Phylotranscriptomic analysis of the origin and early diversification of land plants. Proc Natl Acad Sci U S A [Internet]. 2014 Nov 11;111(45):E4859-68.

6. Conesa A, Gotz S, Garcia-Gomez JM, Terol J, Talon M, Robles M. Blast2GO: A universal tool for annotation, visualization and analysis in functional genomics research. Bioinformatics [Internet]. 2005 Sep 15;21(18):3674-6.

7. Langmead B, Trapnell C, Pop M, Salzberg SL. Ultrafast and memory-efficient alignment of short DNA sequences to the human genome. Genome Biology [Internet]. 2009;10(3).

8. Mortazavi A, Williams BA, Mccue K, Schaeffer L, Wold B. Mapping and quantifying mammalian transcriptomes by RNA-seq. Nature Methods [Internet]. 2008 Jul;5(7):621-8.

9. Benjamini Y, Hochberg Y. Controlling the false discovery rate - a practical and powerful approach to multiple testing. Journal of the Royal Statistical Society Series B-Methodological [Internet]. 1995;57(1):289-300.

10. Falcon S, Gentleman R. Using GOstats to test gene lists for GO term association. Bioinformatics [Internet]. 2007 Jan 15;23(2):257-8.

11. Ernst J, Bar-Joseph Z. STEM: A tool for the analysis of short time series gene expression data. Bmc Bioinformatics [Internet]. 2006 Apr 5;7:191-.
