# Supplementary material for: Interaction of Temperature and Photoperiod Increases Growth and Oil Content in the Marine Microalgae Dunaliella viridis
Source: PLoS One. 2015 May 19;10(5):e0127562. doi: 10.1371/journal.pone.0127562 (PMC4437649; doi:10.1371/journal.pone.0127562)
Supplement: S1 Table — (DOCX) [file pone.0127562.s014.docx]

**S1 Table. Distribution of different membrane lipid species in *Dunaliella viridis* under our 4 growth conditions at 54 hours.** Presented here are the species that were assayed.

|  | 25°C | | 35°C | |
| --- | --- | --- | --- | --- |
|  | LD | LL | LD | LL |
|  | µg/10^6^ cells | | | |
| DGDG | 522 ± 44 | 375 ± 38 | 405 ± 50 | 596 ± 52 |
| MGDG | 541 ± 51 | 291 ± 36 | 806 ± 128 | 784 ± 82 |
| PG | 198 ± 13 | 183 ± 4 | 160 ± 19 | 241 ± 18 |
| PC | 202 ± 14 | 209 ± 28 | 201 ± 29 | 291 ± 12 |
| PE | 90 ± 11 | 92 ± 4 | 69 ± 10 | 123 ± 11 |
| PI | 80 ± 5 | 77 ± 11 | 71 ± 9 | 93 ± 7 |
| PA | 8 ± 2 | 9 ± 1 | 8 ± 1 | 14 ± 1 |
| TOTAL | 1665 ±124 | 1265 ± 18 | 1733 ± 216 | 2162 ± 148 |
