# Supplementary material for: Interaction of Temperature and Photoperiod Increases Growth and Oil Content in the Marine Microalgae Dunaliella viridis
Source: PLoS One. 2015 May 19;10(5):e0127562. doi: 10.1371/journal.pone.0127562 (PMC4437649; doi:10.1371/journal.pone.0127562)
Supplement: S2 Table — (DOCX) [file pone.0127562.s015.docx]

**S2 Table. Summary of Velvet and Oases assemblies.**

|  | Loci | Transcripts | Mean Length | Median Length | N50 |
| --- | --- | --- | --- | --- | --- |
| Velvet | 25,853 | 25,853 | 1,138 | 805 | 1,722 |
| Oases | 18,503 | 23,657 | 1,548 | 1,269 | 2,091 |
