# Supplementary material for: Interaction of Temperature and Photoperiod Increases Growth and Oil Content in the Marine Microalgae Dunaliella viridis
Source: PLoS One. 2015 May 19;10(5):e0127562. doi: 10.1371/journal.pone.0127562 (PMC4437649; doi:10.1371/journal.pone.0127562)
Supplement: S3 Table — (DOCX) [file pone.0127562.s016.docx]

**S3 Table. Summary of differentially expressed genes at 16, 30, 40 and 54 hrs for factors: light, temperature and combined light and temperature.** The total number of transcripts for all the factors, as well as the number of up-regulated and down-regulated transcripts only for light and temperature at the different time point is reported.

|  | Up-regulated | | | |  | Down-regulated | | | |  | Total | | | |
| --- | --- | --- | --- | --- | --- | --- | --- | --- | --- | --- | --- | --- | --- | --- |
|  | 16 | 30 | 40 | 54 |  | 16 | 30 | 40 | 54 |  | 16 | 30 | 40 | 54 |
| Light | 212 | 763 | 374 | 283 |  | 255 | 79 | 380 | 288 |  | 467 | 842 | 754 | 571 |
|  |  |  |  |  |  |  |  |  |  |  |  |  |  |  |
| Temperature |  | 104 | 299 | 344 |  |  | 54 | 61 | 80 |  |  | 158 | 360 | 424 |
|  |  |  |  |  |  |  |  |  |  |  |  |  |  |  |
| Light and Temperature |  |  |  |  |  |  |  |  |  |  |  | 36 | 88 | 56 |
