# Supplementary material for: Interaction of Temperature and Photoperiod Increases Growth and Oil Content in the Marine Microalgae Dunaliella viridis
Source: PLoS One. 2015 May 19;10(5):e0127562. doi: 10.1371/journal.pone.0127562 (PMC4437649; doi:10.1371/journal.pone.0127562)
Supplement: S4 Table — (DOCX) [file pone.0127562.s017.docx]

**S4 Table. Transcripts differentially expressed under continuous light at 6, 16, 30, 40 or 54 hrs.** The list was sorted by Log_2_FC at 40 hrs from the highest (up-regulated transcripts) to the lowest (down-regulated transcripts), all the transcripts presented in this list were differentially expressed at any one of the five time points.

|  |  | **Log_2_FC(LL/LD)** | | | | |
| --- | --- | --- | --- | --- | --- | --- |
| **Transcript** | **Description** | **6** | **16** | **30** | **40** | **54** |
| 10594 | chloroplast carotene biosynthesis-related protein | -0.1 | 7.4 | -1.1 | 7.2 | -1.8 |
| 2256 | chloroplast carotene biosynthesis-related protein | -0.2 | 5.9 | -2.1 | 7.2 | -2.2 |
| 2154 | carotene biosynthesis-related protein | 0.0 | 4.2 | -0.4 | 6.3 | -1.2 |
| 1906 | high light inducible protein | -0.1 | 3.3 | -0.9 | 5.0 | -1.9 |
| 203 | glyceraldehyde-3-phosphate-dehydrogenase | 0.0 | 3.2 | 3.5 | 2.3 | 2.2 |
| 14127 | short-chain dehydrogenase_reductase SDR | 0.0 | 3.0 | -1.2 | 4.4 | -1.9 |
| 2760 | d-lactate dehydrogenase | -0.1 | 2.6 | 0.3 | 2.1 | -1.0 |
| 7889 | btb poz domain-containing protein kctd16-like | 0.0 | 2.5 | -0.1 | 2.7 | -1.3 |
| 7891 | disproportionating enzyme 1 plastid DPE1 | -0.2 | 2.4 | -0.2 | 1.9 | -1.6 |
| 2540 | chloroplast photosystem ii-associated 22 kda protein | -0.2 | 2.3 | -0.5 | 2.4 | -1.4 |
| 2243 | NADP-binding Rossmann-like domain protein | -0.2 | 2.3 | -0.4 | 2.7 | -0.9 |
| 6590 | aldo-keto reductase | -0.3 | 2.2 | -0.8 | 2.5 | -1.2 |
| 783 | LrgB-like family protein | -0.1 | 2.1 | -0.3 | 2.2 | -1.6 |
| 2268 | oligo-1,6-glucosidase O 1,6G | 0.0 | 2.1 | 0.4 | 2.3 | -0.8 |
| 4644 | phytanoyl- dioxygenase | 0.0 | 2.1 | 0.3 | 1.2 | -1.2 |
| 564 | 6-phosphogluconate dehydrogenase | 0.0 | 2.0 | 0.6 | 1.6 | -0.4 |
| 7108 | glutathione peroxidase | -0.1 | 2.0 | -0.3 | 2.0 | -1.2 |
| 2378 | ankyrin repeat domain RING-finger domain containing protein | -0.3 | 1.9 | -0.5 | 1.7 | -1.5 |
| 5784 | dual specificity protein phosphatase 8 | -0.2 | 1.9 | 0.1 | 1.7 | -0.8 |
| 357 | elongation factor ef-3 | -0.2 | 1.9 | 0.2 | 2.8 | -1.2 |
| 689 | rieske (2fe-2s) domain protein | -0.1 | 1.9 | -0.4 | 1.5 | -0.9 |
| 2598 | tpr repeat region family protein | -0.2 | 1.9 | -0.2 | 1.9 | -1.1 |
| 3322 | carotenoid cleavage dioxygenase | 0.0 | 1.8 | -0.5 | 2.5 | -0.9 |
| 1615 | ferric-chelate reductase | -0.3 | 1.8 | 2.4 | 3.5 | 2.4 |
| 740 | nadp-dependent glyceraldehyde-3-phosphate dehydrogenase | 0.0 | 1.8 | -0.4 | 1.9 | -1.8 |
| 12812 | plastid lipid associated protein | -0.2 | 1.8 | -0.4 | 1.1 | -1.3 |
| 543 | starch phosphorylase | 0.1 | 1.8 | 0.2 | 0.9 | -1.1 |
| 5148 | flavin-containing amine oxidasedehydrogenase | -0.2 | 1.7 | -1.0 | 2.0 | -1.3 |
| 5457 | glycoside hydrolase family 13 protein | 0.0 | 1.7 | 0.1 | 1.9 | -1.1 |
| 5575 | peptide methionine sulfoxide reductase | 0.1 | 1.7 | -0.5 | 2.5 | -1.0 |
| 6790 | prostaglandin dehydrogenase | -0.1 | 1.7 | -0.6 | 1.0 | -1.6 |
| 11197 | adenylate kinase | -0.2 | 1.6 | 0.3 | 1.4 | -0.6 |
| 3284 | alpha-glucan water dikinase α-GWD | -0.1 | 1.6 | 0.1 | 1.7 | -1.0 |
| 3147 | chloroplast lumenal protein | -0.3 | 1.6 | 0.3 | 1.5 | -0.9 |
| 5336 | generic methyl-transferase | -0.3 | 1.6 | -0.8 | 1.3 | -1.3 |
| 2578 | mitochondrial substrate carrier family protein | -0.2 | 1.6 | 0.2 | 1.2 | -0.9 |
| 1603 | signal peptide peptidase | -0.1 | 1.6 | -0.4 | 1.9 | -1.2 |
| 3849 | carotenoid cleavage dioxygenase | -0.2 | 1.5 | -0.6 | 1.4 | -0.9 |
| 5366 | 6-4 photolyase | -0.1 | 1.4 | -0.3 | 1.2 | -0.8 |
| 5609 | abc transporter g family member 14-like | -0.1 | 1.4 | -0.6 | 1.4 | -1.3 |
| 17333 | carotenoid cleavage dioxygenase 1 | -0.1 | 1.4 | -1.1 | 1.2 | -0.8 |
| 2223 | cytochrome c | -0.1 | 1.4 | 0.1 | 1.5 | -0.8 |
| 4413 | dna-formamidopyrimidine glycosylase | -0.3 | 1.4 | -0.2 | 1.8 | -0.8 |
| 4704 | hypoxanthine phosphoribosyltransferase | -0.1 | 1.4 | 0.7 | 1.0 | -0.4 |
| 5348 | phosphofructokinase family protein | 0.0 | 1.4 | 0.3 | 1.3 | -1.0 |
| 12435 | SAP-domain containing protein | -0.6 | 1.4 | -0.6 | 1.9 | -1.6 |
| 5796 | serine hydroxymethyltransferase 3 | -0.1 | 1.4 | -0.4 | 1.5 | -1.4 |
| 228 | white-brown-complex abc transporter family | -0.1 | 1.4 | -0.7 | 1.3 | -1.1 |
| 1701 | cyclopropane fatty acid synthase | -0.1 | 1.3 | -0.6 | 1.1 | -1.3 |
| 7548 | duf500 domain protein | -0.2 | 1.3 | 0.0 | 1.1 | -0.9 |
| 10304 | early light-inducible protein | 0.0 | 1.3 | -0.1 | 2.7 | -1.3 |
| 11582 | nucleoside phosphatase family protein | -0.8 | 1.3 | -1.1 | 0.9 | -1.7 |
| 1205 | protein disulfide isomerase pdi5 | -0.3 | 1.3 | 0.1 | 1.5 | -1.0 |
| 961 | systemin receptor sr160 | -0.1 | 1.3 | 0.0 | 1.5 | -0.5 |
| 9263 | tetratricopeptide repeat domain protein | 0.0 | 1.3 | -0.6 | 2.4 | -0.9 |
| 2503 | alternative oxidase | -0.1 | 1.2 | -0.2 | 1.0 | -0.8 |
| 10218 | chaperone protein | -0.1 | 1.2 | 0.2 | 1.0 | -0.3 |
| 10619 | dead-box atp-dependent rna helicase 14 | -0.2 | 1.2 | 0.4 | 1.4 | -0.6 |
| 6494 | dnaj homolog subfamily c member 2-like | -0.2 | 1.2 | 0.2 | 1.5 | -0.7 |
| 6784 | exosome component 7 | -0.7 | 1.2 | 0.2 | 1.1 | -0.6 |
| 2262 | f-box lrr-repeat protein 14-like | -0.3 | 1.2 | -0.3 | 1.1 | -1.1 |
| 2356 | ferrochelatase | 0.0 | 1.2 | 0.4 | 1.4 | -0.4 |
| 775 | generic methyltransferase | -0.1 | 1.2 | 0.1 | 1.5 | -0.7 |
| 87 | g-strand telomere binding protein 1 | -0.3 | 1.2 | 0.1 | 1.1 | -1.4 |
| 5296 | helicase protein with ring u-box domain | -0.1 | 1.2 | -0.7 | 1.7 | -1.4 |
| 7445 | heterogeneous nuclear | -0.2 | 1.2 | 0.3 | 1.4 | -1.2 |
| 1486 | nadh dehydrogenase | -0.2 | 1.2 | 0.0 | 1.2 | -1.0 |
| 11214 | peptidyl-trna hydrolase | 0.0 | 1.2 | 0.4 | 1.5 | -0.8 |
| 5725 | pescadillo-like protein | -0.5 | 1.2 | 0.1 | 1.3 | -1.1 |
| 6198 | pyruvate kinase | -0.1 | 1.2 | -0.2 | 0.6 | -1.1 |
| 5658 | solanesyl diphosphate synthase | -0.2 | 1.2 | -0.3 | 2.1 | -1.0 |
| 4677 | succinate dehydrogenase | 0.0 | 1.2 | 0.0 | 1.3 | -1.0 |
| 2502 | thiol-disulfide oxudoreductase dcc | 0.0 | 1.2 | -0.1 | 1.2 | -0.6 |
| 1179 | violaxanthin de-epoxidase-related protein | -0.1 | 1.2 | -0.1 | 1.5 | -0.8 |
| 4592 | ω-3 fatty acid desaturase FAD7 | -0.1 | 1.2 | 0.0 | 1.4 | -0.9 |
| 1173 | abc transporter i family member 20-like | -0.2 | 1.1 | -0.5 | 1.5 | -1.3 |
| 3401 | abc(atp-binding) family transporter: iron compound | -0.3 | 1.1 | -0.4 | 1.4 | -1.0 |
| 7021 | atp-dependent helicase rhp16-like | -0.3 | 1.1 | -0.6 | 1.5 | -0.8 |
| 2456 | ccaat enhancer-binding protein zeta | -0.4 | 1.1 | -0.3 | 1.3 | -1.0 |
| 1403 | chaperone activity of bc1 complex- mitochondrial-like | -0.1 | 1.1 | 0.1 | 1.5 | -0.8 |
| 4627 | dimethyladenosine transferase | -0.4 | 1.1 | 0.4 | 1.1 | -0.8 |
| 2463 | diphthamide biosynthesis protein 3 | -0.3 | 1.1 | 0.2 | 1.4 | -0.8 |
| 233 | dna repair protein rad51 homolog 2-like | -0.1 | 1.1 | 0.3 | 1.5 | -0.6 |
| 4981 | DUF2237 domain-containing protein | -0.1 | 1.1 | -0.2 | 2.0 | -1.0 |
| 5934 | DUF4419-domain containing protein | 0.0 | 1.1 | 1.5 | 2.2 | 1.8 |
| 6108 | eukaryotic translation initiation factor 6 | -0.5 | 1.1 | 0.2 | 1.1 | -1.0 |
| 12413 | fad nad -binding domain-containing protein | -0.1 | 1.1 | 0.7 | 1.1 | -0.5 |
| 1822 | ferric-chelate reductase | -0.2 | 1.1 | 0.3 | 1.1 | -0.6 |
| 3274 | glutamine amidotransferase subunit | -0.1 | 1.1 | 0.1 | 1.6 | -0.9 |
| 5672 | heterogeneous nuclearribonucleoprotein a2 | -0.3 | 1.1 | -0.2 | 1.7 | -0.8 |
| 16170 | light-mediated development protein det1 | 0.1 | 1.1 | -1.0 | 1.0 | -1.2 |
| 7725 | mediator subunit med7 | -0.5 | 1.1 | 0.4 | 1.1 | -0.9 |
| 2944 | mitochondrial rna-splicing protein mrs3 | -0.2 | 1.1 | 0.0 | 1.3 | -0.9 |
| 2941 | phosphoserine aminotransferase | 0.1 | 1.1 | 0.3 | 1.3 | -0.5 |
| 6119 | protein of clr family | -0.1 | 1.1 | -0.2 | 0.9 | -1.2 |
| 4378 | pyridoxamine 5 -phosphate oxidase- fmn-binding | 0.0 | 1.1 | 0.4 | 1.5 | -0.1 |
| 3796 | sarcosine oxidase | -0.3 | 1.1 | -0.6 | 0.5 | -1.0 |
| 4603 | serine hydroxymethyltransferase | -0.1 | 1.1 | 0.0 | 1.5 | -1.1 |
| 13203 | serine threonine protein kinase | -0.3 | 1.1 | 0.1 | 1.0 | 0.1 |
| 7827 | subtilase family serine protease | -0.1 | 1.1 | 1.1 | -0.2 | 0.2 |
| 11315 | superoxide dismutase | -0.2 | 1.1 | -0.2 | 1.3 | -1.4 |
| 3982 | atp-dependent clp protease adaptor protein | -0.1 | 1.0 | -0.2 | 1.4 | -0.8 |
| 4616 | atp-dependent protease la domain-containing protein | -0.1 | 1.0 | 0.3 | 1.1 | -0.4 |
| 6933 | cytochrome b5 | -0.2 | 1.0 | 0.1 | 1.2 | -0.5 |
| 1789 | dead-box atp-dependent rna helicase 13 | -0.7 | 1.0 | -0.3 | 1.0 | -1.2 |
| 4647 | dead-box atp-dependent rna helicase 14 | 0.1 | 1.0 | 0.7 | 1.5 | -0.3 |
| 2516 | ferredoxin 2 | -0.2 | 1.0 | 0.2 | 1.2 | -0.5 |
| 6826 | major facilitator superfamily mfs_1 | -0.1 | 1.0 | 0.3 | 1.2 | -0.5 |
| 12795 | mate efflux family protein | 0.0 | 1.0 | 0.1 | 1.5 | -0.8 |
| 3106 | mitochondrial import inner membrane translocase subunit tim16 | -0.1 | 1.0 | 0.4 | 1.3 | -0.7 |
| 7400 | mitochondrial protein translocase family | -0.2 | 1.0 | 0.4 | 1.3 | -0.7 |
| 5397 | nad-dependent epimerase dehydratase | -0.3 | 1.0 | -0.2 | 1.1 | -0.8 |
| 2136 | nucleolar component of the u3 processome | -0.2 | 1.0 | 0.3 | 1.3 | -0.6 |
| 14276 | outer membrane autotransporter barrel domain partial | -0.3 | 1.0 | 0.0 | 1.1 | -0.6 |
| 2245 | peptidase d | -0.2 | 1.0 | -0.4 | 1.2 | -1.2 |
| 10620 | pinus taeda anonymous locus 0_16419_01 genomic sequence | -0.1 | 1.0 | 0.3 | 1.2 | -0.7 |
| 9415 | probable anion transporter chloroplastic-like | -0.2 | 1.0 | 0.0 | 1.1 | -1.0 |
| 4151 | protein tyrosine phosphatase | -0.3 | 1.0 | -0.7 | 0.9 | -1.5 |
| 6651 | retinol dehydrogenase 13-like | -0.1 | 1.0 | -0.4 | 1.4 | -1.1 |
| 14078 | retrotransposon ty3-gypsy subclass | -0.1 | 1.0 | -0.1 | -0.1 | -1.3 |
| 1385 | rna-binding protein | 0.0 | 1.0 | 0.9 | 1.1 | -0.4 |
| 136 | rnp-1 like rna-binding protein | -0.2 | 1.0 | 0.0 | 1.0 | -1.0 |
| 2601 | soluble starch synthase SS | -0.2 | 1.0 | -0.2 | 0.9 | -1.2 |
| 4648 | squalene monooxygenase-like | -0.3 | 1.0 | 0.0 | 1.4 | -0.8 |
| 1891 | thioesterase-like protein | 0.1 | 1.0 | 0.6 | 1.3 | 0.2 |
| 6797 | ubiquinol-cytochrome c chaperone family protein | -0.1 | 1.0 | -0.1 | 1.0 | -0.8 |
| 2688 | 2 protein | -0.2 | 0.9 | -0.5 | 0.7 | -1.3 |
| 4196 | aaa-type atpase family protein | 0.0 | 0.9 | -0.2 | 1.3 | -1.0 |
| 6082 | atp-dependent helicase rhp16-like | -0.4 | 0.9 | -0.5 | 1.4 | -1.3 |
| 5799 | biopterin transport-related protein bt1 | -0.1 | 0.9 | 0.1 | 1.1 | -0.9 |
| 7828 | chaperone domain protein | -0.1 | 0.9 | 0.3 | 1.3 | -0.7 |
| 12088 | dead box rna helicase | -0.3 | 0.9 | 0.2 | 1.2 | -0.8 |
| 4500 | deoxyhypusine hydroxylase | -0.1 | 0.9 | 0.6 | 1.3 | -0.4 |
| 2961 | glutathione s-transferase | 0.2 | 0.9 | 0.9 | 1.1 | 0.1 |
| 5267 | guanylate cyclase | 0.4 | 0.9 | -0.6 | 1.5 | -1.3 |
| 8468 | mannan endo- -beta-mannosidase | -0.1 | 0.9 | -0.3 | 1.3 | -0.6 |
| 6065 | membrane steroid-binding protein 1 | -0.1 | 0.9 | 0.4 | 1.2 | -0.3 |
| 2214 | mitochondrial+prohibitin+complex+protein+2 | 0.2 | 0.9 | 0.3 | 1.4 | -0.7 |
| 3831 | oligopeptidase a | -0.2 | 0.9 | 0.0 | 1.1 | -1.1 |
| 14379 | protein fam119a | 0.1 | 0.9 | 0.7 | 1.6 | -0.5 |
| 1445 | pyruvate carboxylase | -0.2 | 0.9 | 0.1 | 1.0 | -1.0 |
| 10789 | regulatory protein | -0.4 | 0.9 | 0.1 | 1.2 | -0.8 |
| 11501 | ribosome biogenesis protein bms1-like protein | -0.2 | 0.9 | 0.1 | 1.1 | -0.6 |
| 7807 | ribosome biogenesis protein bop1 homolog | -0.1 | 0.9 | 0.4 | 1.1 | -0.5 |
| 417 | transmembrane protein c20orf108 | -0.2 | 0.9 | 0.1 | 1.1 | -0.8 |
| 13006 | type-a response regulator protein | -0.5 | 0.9 | -0.7 | 1.5 | -1.6 |
| 12091 | ubiquitin ligase protein | 0.0 | 0.9 | -0.8 | 1.2 | -1.3 |
| 422 | aaa-metalloprotease chloroplast precursor | 0.0 | 0.8 | -0.1 | 1.1 | -0.9 |
| 6403 | aldo keto reductase | -0.4 | 0.8 | 0.7 | 1.5 | -0.2 |
| 5979 | atp-dependent helicase | -0.1 | 0.8 | 0.1 | 1.0 | -0.5 |
| 13124 | bolting time control 1 | 0.1 | 0.8 | 0.2 | 1.0 | 0.2 |
| 7324 | calcium dependent protein kinase-like protein | -0.1 | 0.8 | -0.3 | 1.0 | -1.2 |
| 3829 | chaperonin 21 precursor | -0.3 | 0.8 | 0.4 | 1.4 | -0.9 |
| 8705 | cytochrome p450 | 0.0 | 0.8 | -0.1 | 0.4 | -1.1 |
| 9418 | dash family | -0.2 | 0.8 | -0.5 | 0.6 | -1.2 |
| 8730 | dna-directed rna polymerases and iii subunit rpabc3-like | -0.3 | 0.8 | 0.3 | 1.0 | -0.5 |
| 7675 | dnaj homolog subfamily b member 11-like | -0.4 | 0.8 | 0.4 | 1.3 | -0.7 |
| 7176 | duf564-domain-containing protein | 0.0 | 0.8 | 0.7 | 1.1 | -0.3 |
| 3337 | e3 ubiquitin-protein ligase march3 | -0.1 | 0.8 | -0.3 | 1.1 | -0.8 |
| 4894 | fad dependent oxidoreductase | -0.2 | 0.8 | -0.8 | 1.1 | -1.2 |
| 4164 | glucose-6-phosphate phosphate translocator chloroplast | 0.0 | 0.8 | 0.1 | 1.1 | -0.5 |
| 6421 | histidine kinase rhodopsin | -0.2 | 0.8 | -1.8 | 0.2 | -1.8 |
| 3708 | longevity assurance | -0.3 | 0.8 | 0.5 | 1.3 | -0.5 |
| 4649 | midasin-like protein | -0.6 | 0.8 | -0.9 | 1.0 | -1.6 |
| 686 | mitochondrial import translocase subunit tim23-like | -0.1 | 0.8 | 0.4 | 1.2 | -0.8 |
| 11066 | n-acetyltransferase 9 | -0.1 | 0.8 | 0.7 | 1.1 | 0.1 |
| 1132 | permeases of the major facilitator superfamily | -0.1 | 0.8 | 0.0 | 1.2 | -0.9 |
| 13096 | protein mcm10 homolog | 0.4 | 0.8 | 0.9 | 0.3 | 1.4 |
| 13562 | reverse transcriptase | -0.4 | 0.8 | -0.5 | 0.3 | -1.5 |
| 2099 | ribonucleoside-diphosphate reductase small subunit | -0.4 | 0.8 | 2.3 | 0.8 | 2.3 |
| 6297 | ribosome biogenesis protein bms1 homolog | 0.0 | 0.8 | 0.1 | 1.1 | -0.7 |
| 9091 | sphingosine-1-phosphate lyase | -0.1 | 0.8 | 1.2 | 0.8 | 0.2 |
| 5312 | tetratricopeptide repeat protein | -0.3 | 0.8 | 0.1 | 1.3 | -0.6 |
| 3606 | transcription factor pur-alpha 1 | 0.0 | 0.8 | 0.3 | 1.5 | -0.6 |
| 8073 | transferase caf17 mitochondrial-like | -0.4 | 0.8 | 0.3 | 1.1 | -0.6 |
| 8317 | adp-ribosylation crystallin j1 | -0.1 | 0.7 | -0.1 | 1.1 | -0.5 |
| 9633 | atp-binding cassette superfamily | 0.1 | 0.7 | -0.1 | 1.3 | -0.6 |
| 4441 | atp-dependent helicase | 0.1 | 0.7 | 0.2 | 1.1 | -0.7 |
| 8126 | cobalamin synthesis protein | -0.2 | 0.7 | 0.4 | 1.2 | -0.4 |
| 6908 | cop9 signalosome complex subunit | -0.1 | 0.7 | 1.1 | 1.0 | -0.1 |
| 4632 | deoxyuridine 5 -triphosphate nucleotidohydrolase | -0.1 | 0.7 | 0.9 | 1.0 | 0.1 |
| 10467 | dmt family permease | -0.4 | 0.7 | -0.4 | 1.1 | -0.8 |
| 3160 | dna-directed rna polymerase i subunit rpa49 | 0.1 | 0.7 | 0.5 | 1.0 | -0.4 |
| 15252 | gag-pol polyprotein | -0.2 | 0.7 | -0.5 | 0.2 | -1.3 |
| 913 | glutathione s-transferase-like protein | -0.1 | 0.7 | -0.2 | 1.1 | -0.9 |
| 10843 | guanylate cyclase | -0.6 | 0.7 | -1.2 | 0.2 | -0.9 |
| 8947 | heat shock protein | -0.2 | 0.7 | 0.5 | 1.2 | -0.5 |
| 351 | heat shock protein hsp70g | -0.1 | 0.7 | 0.6 | 1.4 | -0.6 |
| 1756 | kinesin light chain | -0.2 | 0.7 | -0.1 | 0.8 | -1.1 |
| 11820 | low-co2-inducible protein | -0.1 | 0.7 | -0.2 | 0.3 | -1.0 |
| 551 | lycopene beta cyclase | -0.1 | 0.7 | 0.1 | 1.1 | -0.5 |
| 6437 | methyltransferase type 12 | -0.1 | 0.7 | 0.7 | 1.2 | -0.2 |
| 8687 | mitochondrial import inner membrane translocase SU tim17-like | -0.1 | 0.7 | 0.7 | 1.1 | -0.6 |
| 9602 | mitochondrial import inner membrane translocase subunit tim8 a | -0.2 | 0.7 | 0.6 | 1.2 | -0.4 |
| 3504 | multidrug pheromone mdr abc transporter family | -0.1 | 0.7 | -0.4 | 1.2 | -0.6 |
| 8323 | pheophorbide a oxygenase | 0.0 | 0.7 | -0.2 | 0.6 | -1.0 |
| 3858 | prematurely terminated mrna decay factor-like | -0.2 | 0.7 | 1.0 | 1.3 | 0.0 |
| 1502 | protein of clr family | -0.2 | 0.7 | 0.2 | 1.1 | -0.6 |
| 2814 | protein phosphatase 2c-related protein | -0.4 | 0.7 | -0.1 | 1.1 | -0.6 |
| 5019 | ribosome recycling factor | -0.1 | 0.7 | 0.4 | 1.0 | -0.5 |
| 331 | rubredoxin-like protein | 0.0 | 0.7 | 0.0 | 1.7 | -1.1 |
| 10107 | salicylate hydroxylase | -0.2 | 0.7 | -0.6 | 0.7 | -1.2 |
| 11119 | set domain-containing protein | -0.1 | 0.7 | 0.3 | 1.1 | -0.5 |
| 5944 | slr1470 gene product | -0.2 | 0.7 | 0.0 | 1.1 | -0.7 |
| 13322 | tpr domain protein | -0.4 | 0.7 | -0.1 | 1.3 | -1.2 |
| 11154 | zinc-containing alcohol dehydrogenase | 0.4 | 0.7 | 0.6 | 1.0 | 1.6 |
| 8334 | adenylate guanylate cyclase | -0.2 | 0.6 | -0.4 | 0.6 | -1.0 |
| 1228 | aldo keto reductase | 0.1 | 0.6 | 0.1 | 1.3 | -1.1 |
| 14526 | chromatin assembly factor 1 subunit a-like | 0.0 | 0.6 | 1.2 | 0.6 | 0.8 |
| 11178 | co- -like protein | -0.1 | 0.6 | 0.1 | 1.1 | -0.7 |
| 461 | cpd photolyase | -0.1 | 0.6 | 0.0 | 1.2 | -0.9 |
| 5367 | exosome complex component rrp43-like | -0.1 | 0.6 | 0.9 | 1.0 | -0.4 |
| 3763 | fad dependent oxidoreductase | -0.1 | 0.6 | -0.3 | 1.1 | -0.8 |
| 1580 | fad fmn-containing dehydrogenase | 0.0 | 0.6 | 0.2 | 1.2 | -0.6 |
| 11426 | g-strand telomere binding protein 1 | -0.7 | 0.6 | -0.1 | 0.2 | -1.2 |
| 942 | heat repeat-containing protein 1 | -0.1 | 0.6 | -0.2 | 1.0 | -1.0 |
| 85 | heat-shock inducible hsp70 | 0.0 | 0.6 | 0.5 | 1.1 | -0.8 |
| 3832 | isrm2011-2 transposase protein | -0.2 | 0.6 | -0.2 | 1.3 | -1.3 |
| 15648 | major facilitator superfamily mfs_1 | -0.3 | 0.6 | -0.2 | 1.2 | -0.9 |
| 8862 | methionine-r-sulfoxide reductase | 0.0 | 0.6 | 0.1 | 1.3 | -0.6 |
| 3458 | methionyl-trna formyltransferase | 0.1 | 0.6 | 0.2 | 1.0 | -0.7 |
| 7894 | nucleolar protein | -0.1 | 0.6 | 0.2 | 1.0 | -1.0 |
| 589 | phosphate dikinase 1 | -0.2 | 0.6 | 0.3 | 1.1 | -0.7 |
| 5587 | programmed cell death protein 2 | 0.0 | 0.6 | 0.7 | 1.0 | -0.4 |
| 7975 | ribosome biogenesis protein wdr12 homolog | -0.2 | 0.6 | 0.5 | 1.1 | -0.4 |
| 884 | rnp-1 like rna-binding protein | -0.3 | 0.6 | 1.0 | 0.7 | 0.2 |
| 6602 | short-chain dehydrogenase reductase sdr | 0.2 | 0.6 | 0.2 | 1.3 | -0.7 |
| 6776 | single helix lhc light protein | -0.1 | 0.6 | 0.3 | 1.2 | -1.0 |
| 10659 | sodium calcium exchanger protein | -0.4 | 0.6 | 0.0 | 0.6 | -1.1 |
| 5493 | solute carrier family 35 member f1 | -0.6 | 0.6 | -0.5 | 0.3 | -1.0 |
| 1908 | transducin -like 3 | -0.1 | 0.6 | 0.1 | 1.1 | -0.9 |
| 12786 | u3 small nucleolar rna-associated protein 20-like | -0.5 | 0.6 | -0.3 | 0.8 | -1.1 |
| 3847 | zinc finger protein 622 | -0.2 | 0.6 | 0.6 | 1.1 | -0.2 |
| 963 | alanyl-trna synthetase | -0.2 | 0.5 | -0.3 | 1.7 | -1.4 |
| 10178 | ariadne-like ring finger protein | -0.1 | 0.5 | 0.2 | 1.5 | -0.6 |
| 2847 | cytochrome oxidase subunit 1 | 0.9 | 0.5 | -2.6 | -0.1 | 1.0 |
| 11874 | dna repair and recombination protein | -0.3 | 0.5 | -0.6 | 1.0 | -1.0 |
| 6087 | flagellar associated protein | 0.1 | 0.5 | 1.4 | -0.2 | 0.6 |
| 266 | heat shock protein 60 | -0.1 | 0.5 | 0.1 | 1.0 | -1.1 |
| 7768 | histidine kinase-like ATPase | -0.3 | 0.5 | -0.5 | 1.4 | -1.6 |
| 13851 | hypothetical membrane associated protein | -0.3 | 0.5 | -0.1 | 0.7 | -1.0 |
| 11028 | inosine xanthosine triphosphatase | -0.3 | 0.5 | 0.5 | 1.4 | -0.6 |
| 3307 | membrane-associated zinc metalloprotease | -0.1 | 0.5 | 0.0 | 1.3 | -0.6 |
| 9182 | midasin homolog | -0.1 | 0.5 | -0.8 | 0.6 | -1.2 |
| 16087 | mixed-lineage leukemia mll | -0.2 | 0.5 | 0.1 | 0.4 | -1.3 |
| 3428 | nudix family protein | 0.2 | 0.5 | 2.0 | 0.3 | 2.2 |
| 13636 | pas pac sensor hybrid histidine kinase | -0.2 | 0.5 | -0.9 | 1.4 | -1.4 |
| 2844 | plastid division protein | -0.5 | 0.5 | 2.0 | 0.3 | 2.1 |
| 2647 | probable serine threonine-protein kinase abkc-like | -0.2 | 0.5 | -0.5 | 0.1 | -1.1 |
| 5563 | proliferating cell nuclear antigen | -0.1 | 0.5 | 1.1 | 0.6 | 0.8 |
| 3889 | proteasome assembly chaperone | -0.1 | 0.5 | 1.1 | 0.5 | 0.4 |
| 4040 | putative polyprotein [Albugo laibachii Nc14] | -0.3 | 0.5 | -0.6 | 0.1 | -1.2 |
| 2985 | ribonuclease h2 subunit a | -0.5 | 0.5 | 1.3 | 0.6 | 1.0 |
| 375 | ribonucleoside-diphosphate reductase large subunit | -0.2 | 0.5 | 1.3 | 0.1 | 1.2 |
| 8482 | rna terminal 3 phosphate cyclase | -0.1 | 0.5 | 0.3 | 1.0 | -0.4 |
| 2454 | SAM-dependent methyltransferase domain-containing protein | -0.3 | 0.5 | 0.3 | 1.1 | -0.1 |
| 16933 | sgt1-like protein | -0.4 | 0.5 | 0.2 | 0.5 | -1.0 |
| 792 | short-chain dehydrogenase reductase sdr | 0.0 | 0.5 | 0.1 | 1.0 | -0.4 |
| 297 | small subunit ribosomal RNA (rrnS), plastid | 1.2 | 0.5 | -3.6 | -0.6 | 2.9 |
| 4921 | threonine deaminase | -0.2 | 0.5 | 0.4 | 1.0 | -0.7 |
| 2929 | type a von willebrand factor domain-containing protein | -0.1 | 0.5 | -0.9 | 0.6 | -1.3 |
| 15 | ω-6 fatty acid desaturase 1 FAD6-1 | 0.0 | 0.5 | 0.0 | 0.7 | -1.2 |
| 3121 | acetyltransferase nsi | -0.1 | 0.4 | 0.6 | 1.1 | -0.3 |
| 13040 | atp-binding cassette sub-family a member 3 | -0.6 | 0.4 | -0.7 | -0.1 | -1.2 |
| 9352 | chain 4ank: consensus repeats | -0.5 | 0.4 | 1.2 | 0.1 | 0.3 |
| 8675 | enoyl- hydratase isomerase | -0.4 | 0.4 | 1.1 | 0.2 | -0.2 |
| 67 | heat shock protein 90 | 0.0 | 0.4 | 0.2 | 0.6 | -1.0 |
| 2717 | meiotic recombination protein spo11-2 | -0.4 | 0.4 | 1.4 | 0.6 | 0.7 |
| 11329 | mfs general substrate transporter | -0.5 | 0.4 | -0.2 | 0.9 | -1.1 |
| 8797 | microtubule-associated protein asp | -0.2 | 0.4 | 0.2 | 1.1 | -0.9 |
| 16157 | nadh dehydrogenase subunit 2 | 0.7 | 0.4 | -1.3 | 0.1 | 1.7 |
| 9671 | nadh dehydrogenase subunit 5 | 0.3 | 0.4 | -1.0 | 0.3 | 1.5 |
| 8793 | nudix hydrolase | 0.0 | 0.4 | 1.2 | 0.1 | 0.5 |
| 3409 | protein of clr family | -0.2 | 0.4 | -0.6 | 0.7 | -1.0 |
| 11033 | proteophosphoglycan 5 | 0.1 | 0.4 | -0.7 | -0.1 | -1.2 |
| 7884 | rad2 family | -0.1 | 0.4 | 1.0 | 0.7 | 0.6 |
| 13911 | retrotransposon ty3-gypsy subclass | -0.3 | 0.4 | -0.2 | -0.2 | -1.0 |
| 1881 | rrna methylase | 0.1 | 0.4 | 0.2 | 1.1 | -0.4 |
| 12066 | SCP-domain containing protein | -0.3 | 0.4 | 1.6 | 1.1 | 1.3 |
| 6500 | thioredoxin-like protein | -0.1 | 0.4 | 1.0 | 0.5 | 0.0 |
| 773 | translation initiation factor if-2 | -0.4 | 0.4 | -0.3 | 0.8 | -1.0 |
| 8511 | transmembrane protein 17 family | 0.3 | 0.4 | 1.3 | -0.2 | 0.7 |
| 11235 | ubiquitin carboxyl-terminal hydrolase 12 | -0.2 | 0.4 | -0.5 | 0.2 | -1.4 |
| 5939 | abc transporter c family member 1 | 0.0 | 0.3 | -0.2 | 1.0 | -1.1 |
| 10078 | acetamidase formamidase | 0.0 | 0.3 | 1.3 | -0.1 | 1.3 |
| 4115 | a-chain ankyrin repeat proteins | -0.3 | 0.3 | 1.3 | 0.6 | 0.3 |
| 15527 | ankyrin repeat domain-containing protein 50 | 0.0 | 0.3 | -0.3 | 1.1 | -0.5 |
| 10772 | b9 domain-containing protein 1 | -0.3 | 0.3 | 1.1 | 0.0 | 1.0 |
| 895 | cell division cycle protein 45 | -0.1 | 0.3 | 1.6 | -0.1 | 1.5 |
| 1401 | chloroplast elongation factor g | 0.0 | 0.3 | -0.1 | 1.0 | -1.2 |
| 7915 | cullin-like protein1 | -0.3 | 0.3 | -1.0 | 0.2 | -1.1 |
| 2008 | dna replication complex gins protein sld5-like | -0.1 | 0.3 | 1.2 | 0.0 | 0.4 |
| 3746 | ferric-chelate reductase | -0.3 | 0.3 | -0.2 | 0.3 | -1.0 |
| 8576 | flagellar associated protein | -0.1 | 0.3 | 1.3 | -0.2 | 0.4 |
| 4595 | flagellar associated protein | 0.0 | 0.3 | 1.0 | -0.3 | 0.3 |
| 7015 | flagellar associated protein | 0.1 | 0.3 | 1.1 | -0.3 | 0.4 |
| 1366 | guanylate cyclase | -0.1 | 0.3 | -0.8 | 0.5 | -1.0 |
| 11746 | homeobox prox 1 | 0.3 | 0.3 | 1.2 | 0.4 | 0.1 |
| 6176 | homeodomain protein | -0.2 | 0.3 | -0.2 | 0.3 | -1.0 |
| 9760 | ion channel | -0.3 | 0.3 | 0.3 | 1.1 | -0.6 |
| 6169 | isoprenylcysteine carboxyl methyltransferase | 0.0 | 0.3 | 1.0 | 0.1 | 0.6 |
| 8607 | leucine-rich repeat | -0.1 | 0.3 | 0.9 | 1.3 | 0.3 |
| 1649 | major facilitator superfamily | -0.4 | 0.3 | -0.1 | 0.3 | -1.1 |
| 6575 | plastid-lipid-associated protein | 0.0 | 0.3 | 1.0 | 0.1 | 0.1 |
| 13376 | retrotransposable element tf2 155 kda protein type 1-like | 0.0 | 0.3 | -0.3 | -0.4 | -1.3 |
| 4929 | retrotransposon ty3-gypsy subclass | -0.1 | 0.3 | -0.2 | 0.0 | -1.0 |
| 17892 | retrotransposon ty3-gypsy subclass | 0.1 | 0.3 | -0.7 | -0.6 | -1.2 |
| 10259 | reverse transcriptase | 0.5 | 0.3 | -1.0 | -0.7 | -2.0 |
| 17933 | ribulose-1,5-bisphosphate carboxylase/oxygenase large subunit (Rubisco) rbcL | 0.9 | 0.3 | -2.9 | -0.6 | 2.6 |
| 3627 | RNA recognition motif superfamily protein | -0.1 | 0.3 | -0.7 | 0.7 | -1.6 |
| 965 | snare associated golgi protein | -0.1 | 0.3 | 1.2 | 0.7 | 0.3 |
| 12111 | submergence induced protein 2 | -0.2 | 0.3 | 0.2 | 1.0 | -1.1 |
| 10488 | trithorax-like histone-lysine n-methyltransferase | -0.2 | 0.3 | 1.9 | 0.1 | 1.8 |
| 6850 | tryptophanyl-trna synthetase | -0.2 | 0.3 | 0.1 | 1.0 | -0.8 |
| 2142 | ubiquitin carboxyl-terminal hydrolase 24 | -0.3 | 0.3 | -0.3 | 0.3 | -1.1 |
| 12045 | vamp (vesicle-associated membrane protein) family protein | 0.2 | 0.3 | 1.0 | 0.3 | 0.0 |
| 11539 | (2R)-phospho-3-sulfolactate synthase ComA | -0.3 | 0.2 | 0.3 | 0.2 | -1.1 |
| 6466 | 4-hydroxyphenylacetate permease | -0.2 | 0.2 | 1.1 | 0.5 | 0.4 |
| 4687 | abc transporter | -0.2 | 0.2 | -0.3 | 1.1 | -1.1 |
| 7270 | adenylate guanylate cyclase | -0.2 | 0.2 | -0.6 | 0.0 | -1.1 |
| 1681 | adp-ribosylation factor c1 | -0.3 | 0.2 | 1.2 | 0.5 | -0.1 |
| 3529 | adp-ribosylation factor-like protein 6-like | -0.3 | 0.2 | 1.0 | 0.1 | 0.3 |
| 11800 | amidase | -0.1 | 0.2 | 1.1 | 0.3 | -0.1 |
| 12969 | ankyrin repeat protein | -0.1 | 0.2 | 1.0 | 0.0 | 0.0 |
| 14984 | ankyrin repeat protein | -0.1 | 0.2 | -0.8 | -0.3 | -1.2 |
| 2048 | beta-carotene hydroxylase | -0.2 | 0.2 | 0.2 | 1.9 | -1.0 |
| 14194 | cell cycle protease | -0.1 | 0.2 | 1.9 | -0.3 | 1.9 |
| 4782 | chaperone protein dnaj 6 | -0.5 | 0.2 | 1.1 | 0.2 | 0.7 |
| 12018 | condensin-2 complex subunit d3 | -0.3 | 0.2 | 1.3 | -0.4 | 0.9 |
| 827 | ef hand family protein | -0.2 | 0.2 | 1.4 | -0.2 | 0.3 |
| 7878 | flagellar associated protein | 0.4 | 0.2 | 1.1 | -0.5 | 0.0 |
| 7413 | flagellar associated protein | 0.0 | 0.2 | 1.1 | -0.6 | 0.5 |
| 7698 | glutathione s-transferase | 0.0 | 0.2 | 1.0 | -0.2 | 1.0 |
| 7285 | guanylate cyclase | -0.1 | 0.2 | -0.6 | -0.3 | -1.0 |
| 630 | heat shock protein beta-11-like | -0.1 | 0.2 | 1.2 | -0.2 | 0.3 |
| 11977 | histone methyltransferase | 0.0 | 0.2 | 1.4 | 0.2 | 1.6 |
| 1182 | kelch repeat type 1-containing protein | -0.1 | 0.2 | 0.1 | -0.3 | -1.0 |
| 9729 | kinesin-like protein | -0.5 | 0.2 | 1.2 | -0.7 | 0.3 |
| 7208 | methylcrotonoyl-CoA carboxylase alpha subunit | -0.2 | 0.2 | -0.2 | 0.6 | -1.1 |
| 5379 | minichromosome maintenance protein 10 isoform 1-like | 0.2 | 0.2 | 1.8 | 0.8 | 1.8 |
| 10825 | mitochondrial mmp37-like protein | -0.1 | 0.2 | 1.5 | 0.4 | 0.7 |
| 10785 | nucleolar gtp-binding protein 1-like | -0.2 | 0.2 | 1.0 | 0.1 | -0.1 |
| 8695 | phosphoglycerate mutase | 0.1 | 0.2 | 1.4 | -0.2 | 0.3 |
| 18668 | photosystem i assembly protein ycf3 | 2.6 | 0.2 | -3.4 | -0.7 | 2.9 |
| 13740 | progesterone-induced-blocking factor 1-like | 0.1 | 0.2 | 1.3 | -0.3 | 0.8 |
| 9818 | protein af-9 homolog | 0.0 | 0.2 | 1.0 | 0.6 | 0.3 |
| 8051 | protein of cse family | -0.2 | 0.2 | 0.3 | -0.3 | -1.0 |
| 3448 | protein phosphatase 2c | -0.1 | 0.2 | -0.2 | 0.2 | -1.0 |
| 5942 | quinone oxidoreductase-like protein 2 homolog | -0.2 | 0.2 | 1.2 | 0.4 | 0.6 |
| 12806 | retrotransposon ty3-gypsy subclass | 0.0 | 0.2 | -0.1 | 0.1 | -1.2 |
| 3318 | reverse transcriptase | 0.1 | 0.2 | -0.2 | -0.2 | -1.1 |
| 15897 | rta1 domain protein | 0.1 | 0.2 | 1.1 | 1.4 | 1.5 |
| 3408 | ser thr protein phosphatase family protein | 0.0 | 0.2 | 0.8 | 1.0 | -0.1 |
| 5474 | snf2 family dna-dependent atpase | -0.3 | 0.2 | -0.4 | 0.3 | -1.3 |
| 2208 | spindle assembly abnormal protein 6 homolog | -0.3 | 0.2 | 1.1 | -0.1 | 0.3 |
| 1886 | stress-inducible protein | 0.0 | 0.2 | 0.6 | 1.0 | -0.7 |
| 503 | structural maintenance of chromosomes protein 5 puta | -0.2 | 0.2 | 1.0 | 0.4 | 0.3 |
| 1263 | transcriptional family | -0.3 | 0.2 | -0.7 | 1.0 | -1.0 |
| 3271 | transmembrane protein 216-like | 0.0 | 0.2 | 1.4 | 0.3 | 1.7 |
| 242 | 40s ribosomal protein s25-1 | 0.1 | 0.1 | 1.0 | 0.3 | -0.2 |
| 2391 | 60s ribosomal protein l17 | 0.1 | 0.1 | 1.2 | 0.2 | 0.1 |
| 1000 | actin | 0.0 | 0.1 | 1.2 | 0.2 | 0.2 |
| 544 | ADP-glucose phosphorylase small subunit AGPase | -0.1 | 0.1 | -0.1 | 0.2 | -1.1 |
| 5314 | alpha- -mannosyl-glycoprotein beta- 2-n- | -0.5 | 0.1 | 1.3 | 0.2 | 0.6 |
| 434 | alpha tubulin 1 | 0.0 | 0.1 | 1.0 | -0.2 | 0.2 |
| 10101 | APETALA2-like protein | -0.5 | 0.1 | -0.5 | 0.0 | -1.6 |
| 12471 | atp synthase cf1 epsilon subunit | 2.8 | 0.1 | -4.1 | -0.6 | 2.2 |
| 1590 | A-type cyclin | -0.1 | 0.1 | 1.7 | 0.3 | 1.0 |
| 7429 | casein kinase ii alpha subunit | 0.1 | 0.1 | 1.1 | 0.0 | 0.4 |
| 3944 | chain structure of the plant transcriptional regulator pbf-2 | -0.1 | 0.1 | 1.4 | 0.0 | 0.4 |
| 332 | dna methyltransferase | -0.3 | 0.1 | 1.1 | 0.2 | 0.6 |
| 4641 | dna polymerase delta subunit | -0.1 | 0.1 | 1.0 | 0.2 | 0.0 |
| 18535 | endonuclease-reverse transcriptase | -0.4 | 0.1 | -0.8 | -1.1 | -0.3 |
| 6585 | ferredoxin | 0.1 | 0.1 | 0.2 | 1.1 | -0.2 |
| 4443 | flagellar associated protein | 0.2 | 0.1 | 1.1 | -0.5 | 0.5 |
| 3927 | flagellar associated protein | -0.1 | 0.1 | 1.3 | -0.6 | 0.7 |
| 7518 | flagellar associated protein | 0.0 | 0.1 | 1.1 | -0.7 | 0.5 |
| 625 | flagellar outer dynein arm light chain thioredoxin-like protein | 0.0 | 0.1 | 1.7 | -0.6 | 0.8 |
| 2744 | gtpase slip-gc | -0.2 | 0.1 | 1.1 | 0.1 | 0.5 |
| 363 | high affinity nitrate transporter | -0.2 | 0.1 | 1.1 | 0.4 | -0.4 |
| 4011 | Histone H3 | -0.1 | 0.1 | 1.1 | -0.1 | 0.1 |
| 7863 | hypothetical ef-hand protein | 0.0 | 0.1 | 1.6 | -0.2 | 0.9 |
| 4957 | kelch motif family protein | -0.2 | 0.1 | 1.1 | -0.5 | 0.6 |
| 2217 | MGDG specific palmitate Δ-7 desaturase FAD5 | -0.1 | 0.1 | 0.1 | 0.3 | -1.2 |
| 777 | nadh dehydrogenase subunit 4 | 0.9 | 0.1 | -1.6 | -0.5 | 2.3 |
| 4169 | nadh:ubiquinone oxidoreductase 8 kda subunit | 0.1 | 0.1 | 1.1 | 0.2 | 0.4 |
| 4181 | ngg1 interacting factor 3 like 1 binding protein 1 isoform 1 | 0.1 | 0.1 | 1.0 | 0.1 | -0.1 |
| 935 | nucleosome assembly protein | -0.1 | 0.1 | 1.0 | -0.3 | 0.2 |
| 12564 | photosystem ii 44 kda protein | 2.2 | 0.1 | -3.8 | -0.1 | 2.8 |
| 1076 | protein disulfide isomerase 1 | 0.0 | 0.1 | 1.1 | 0.3 | 0.1 |
| 13382 | protein kinase domain containing protein | -0.1 | 0.1 | 2.9 | -0.2 | 2.8 |
| 15434 | retrotransposon ty3-gypsy subclass | 0.0 | 0.1 | -0.1 | 0.1 | -1.2 |
| 13902 | retrotransposon ty3-gypsy subclass | -0.2 | 0.1 | -0.3 | -0.1 | -1.2 |
| 5257 | retrotransposon ty3-gypsy subclass | 0.0 | 0.1 | 0.1 | -0.2 | -1.1 |
| 1108 | ribosomal protein s10 | -0.1 | 0.1 | 1.0 | 0.4 | -0.4 |
| 2818 | rna polymerase ii c-terminal domain phosphatase-like 3-like | -0.4 | 0.1 | 1.1 | -0.2 | 0.1 |
| 2095 | septum site-determining protein | -0.2 | 0.1 | 3.0 | 0.1 | 2.9 |
| 9817 | sjogren syndrome nuclear autoantigen 1 | 0.0 | 0.1 | 1.6 | -0.1 | 1.0 |
| 9420 | snf2 family chromodomain-helicase protein | -0.3 | 0.1 | -0.6 | 0.2 | -1.3 |
| 6388 | strawberry notch homolog 1 | 0.0 | 0.1 | 1.8 | 0.4 | 1.9 |
| 10204 | transducin wd-40 repeat-containing protein | -0.2 | 0.1 | -1.0 | -0.1 | -1.1 |
| 4268 | transmembrane protein 231 | -0.3 | 0.1 | 1.2 | 0.0 | 0.7 |
| 1566 | ureidoglycolate hydrolase | 0.2 | 0.1 | 1.2 | -0.6 | 0.4 |
| 5494 | vacuolar protein sorting-associated protein 25 | -0.1 | 0.1 | 1.1 | 0.3 | 0.2 |
| 13457 | 20s proteasome beta subunit a1 | -0.1 | 0.0 | 1.0 | 0.2 | -0.2 |
| 13776 | ABCG transporter family | -0.2 | 0.0 | 1.6 | 1.2 | 2.8 |
| 4068 | b9 domain-containing protein 2-like | -0.1 | 0.0 | 1.1 | 0.1 | 0.7 |
| 4005 | calcium calmodulin-dependent protein kinase kinase alpha | 0.0 | 0.0 | -0.5 | 0.5 | -1.1 |
| 2122 | calmodulin related calcium binding protein | 0.0 | 0.0 | 1.1 | -0.4 | 0.2 |
| 6763 | carotene isomerase | -0.2 | 0.0 | 1.2 | -0.8 | 0.8 |
| 8985 | chloroplast division site determinant | -0.2 | 0.0 | 2.8 | 0.0 | 3.0 |
| 6278 | chromatin assembly factor 1 subunit b | -0.1 | 0.0 | 1.1 | 0.2 | 1.0 |
| 6324 | cytosine-5 dna methyltransferase | -0.1 | 0.0 | 1.1 | 0.2 | 1.1 |
| 1089 | dynein light chain | -0.1 | 0.0 | 1.3 | -0.7 | 1.2 |
| 3294 | dynein light chain axonemal | -0.1 | 0.0 | 1.0 | -0.7 | 0.7 |
| 16515 | e3 ubiquitin-protein ligase rfwd3 | -0.2 | 0.0 | 2.7 | -0.7 | 2.6 |
| 6441 | ef-hand calcium-binding domain-containing protein 10-like | 0.0 | 0.0 | 1.4 | -0.7 | 0.5 |
| 4793 | enoyl- reductase | 0.0 | 0.0 | 1.0 | 0.3 | -0.2 |
| 7147 | family membrane protein | 0.0 | 0.0 | 1.5 | 0.1 | 0.7 |
| 6399 | flagellar associated protein | -0.2 | 0.0 | 1.4 | -0.5 | 0.6 |
| 6636 | flagellar associated protein | 0.2 | 0.0 | 1.1 | -0.6 | 0.5 |
| 5514 | flagellar associated protein | -0.1 | 0.0 | 1.0 | -0.8 | 0.5 |
| 3333 | flagellar associated protein | 0.1 | 0.0 | 1.2 | -0.8 | 0.7 |
| 5866 | flagellar associated protein | 0.1 | 0.0 | 1.2 | -0.9 | 0.8 |
| 13139 | flagellar associated protein | 0.0 | 0.0 | 1.2 | -1.2 | 0.5 |
| 6120 | flagellar flavodoxin | 0.0 | 0.0 | 1.4 | 0.8 | 1.9 |
| 10386 | GAF domain containing protein kinase | -0.1 | 0.0 | -1.4 | -0.5 | -2.0 |
| 10856 | geranylgeranyl transferase type-2 subunit | -0.2 | 0.0 | 1.1 | 0.1 | 0.2 |
| 11060 | guanylyl and adenylyl cyclase family member | -0.5 | 0.0 | -0.8 | -0.1 | -1.3 |
| 3818 | inhibitor of growth | 0.1 | 0.0 | 1.3 | -0.1 | 0.1 |
| 4368 | kinase-like protein | -0.1 | 0.0 | -0.1 | 0.4 | -1.1 |
| 10099 | leucine-rich repeat | -0.1 | 0.0 | -0.2 | 0.5 | -1.1 |
| 340 | macrophage migration inhibitory factor family protein | 0.0 | 0.0 | -1.0 | -0.8 | -2.1 |
| 6282 | meiosis-specific nuclear structural protein 1-like | -0.1 | 0.0 | 1.0 | -0.8 | 0.4 |
| 7838 | mpn domain-containing protein | -0.1 | 0.0 | 1.1 | 0.1 | 0.2 |
| 219 | nadp-malate dehydrogenase | 0.0 | 0.0 | 1.0 | 0.3 | -0.1 |
| 9332 | nucleoside diphosphate kinase 7-like | -0.1 | 0.0 | 1.5 | -0.9 | 0.7 |
| 10033 | ovarian cancer-associated gene 2 protein | -0.1 | 0.0 | 1.5 | -0.2 | 1.0 |
| 45 | phd f-box containing | 0.0 | 0.0 | 1.0 | -0.3 | 0.2 |
| 10683 | phosphatase subunit g4-1 | -0.3 | 0.0 | 1.3 | -0.4 | 0.9 |
| 10041 | photosystem ii protein d1 psbA | 1.0 | 0.0 | -2.9 | -0.4 | 2.2 |
| 4377 | putative parB-like nuclease | 0.2 | 0.0 | 1.0 | 0.2 | 0.0 |
| 5384 | retrotransposable element tf2 155 kda protein type 1-like | 0.0 | 0.0 | -0.2 | -0.4 | -1.2 |
| 3900 | rna-binding protein | -0.3 | 0.0 | -0.4 | -1.1 | -1.2 |
| 5133 | septin-like protein | -0.4 | 0.0 | 2.0 | 0.1 | 1.3 |
| 3423 | s-formylglutathione hydrolase | 0.1 | 0.0 | 1.3 | 0.1 | 0.0 |
| 168 | sodium phosphate symporter | -0.2 | 0.0 | -0.7 | -0.2 | -1.5 |
| 527 | sodium phosphate symporter | -0.1 | 0.0 | -0.6 | -0.3 | -1.3 |
| 10780 | transcripton factor for heterocyst differentiation | -0.3 | 0.0 | 1.1 | 0.6 | 0.2 |
| 7236 | trp-containing protein | -0.2 | 0.0 | 1.0 | -0.3 | 0.3 |
| 14402 | tubulin-specific chaperone e | -0.3 | 0.0 | 1.0 | -0.3 | 0.5 |
| 9268 | ubiquitin thioesterase otubain-like protein | 0.2 | 0.0 | 1.0 | 0.0 | 0.1 |
| 14095 | von willebrand factor type a domain containing protein | -0.3 | 0.0 | 1.1 | 0.4 | 0.4 |
| 7106 | 30s ribosomal protein s11 | 0.1 | -0.1 | 1.1 | 0.3 | 0.1 |
| 5259 | phosphohydrolase 2 | -0.1 | -0.1 | 1.1 | 0.0 | 0.1 |
| 5078 | adp-ribosylation factor-like 16 | -0.2 | -0.1 | 1.2 | -0.3 | 0.0 |
| 3564 | aldo keto reductase | -0.2 | -0.1 | 1.1 | -0.6 | 0.4 |
| 10075 | ankyrin repeat domain-containing protein 13c | -0.2 | -0.1 | -0.7 | -0.1 | -1.3 |
| 3705 | arrestin domain-containing protein 3-like | -0.1 | -0.1 | 1.1 | -0.5 | 1.0 |
| 3933 | calcineurin b | -0.3 | -0.1 | 1.0 | -0.3 | 0.2 |
| 14438 | cell division protein | -0.4 | -0.1 | 2.2 | -0.1 | 2.4 |
| 1686 | cell number regulator 2-like | 0.0 | -0.1 | 1.0 | -0.3 | -0.1 |
| 7538 | chaperone protein | -0.1 | -0.1 | 1.3 | -0.7 | 0.4 |
| 6488 | chloride channel protein clc-d | -0.2 | -0.1 | 1.1 | 0.0 | 0.3 |
| 7731 | chromodomain-containing protein | 0.2 | -0.1 | 2.8 | -0.8 | 2.8 |
| 12254 | chromosome segregation protein smc | -0.2 | -0.1 | 3.2 | -0.6 | 3.4 |
| 7685 | condensin-2 complex subunit h2 | 0.0 | -0.1 | 1.8 | -0.3 | 1.6 |
| 3990 | conserved hypothetical protein [Toxoplasma gondii GT1] | -0.5 | -0.1 | 1.4 | -0.3 | 1.1 |
| 3751 | cop9 signalosome complex subunit 4 | -0.1 | -0.1 | 1.1 | 0.1 | 0.1 |
| 8432 | ef hand family protein | 0.0 | -0.1 | 1.2 | -0.9 | 0.5 |
| 11473 | ef-hand calcium-binding domain-containing protein 11-like | 0.2 | -0.1 | 1.1 | 0.0 | 0.0 |
| 10234 | fgfr1 oncogene partner | -0.5 | -0.1 | 1.2 | -0.2 | 0.5 |
| 2259 | flagellar associated minor isoform | 0.1 | -0.1 | 1.5 | -0.6 | 0.7 |
| 12678 | flagellar associated protein | 0.2 | -0.1 | 1.3 | -0.4 | 0.5 |
| 9646 | flagellar associated protein | -0.2 | -0.1 | 1.1 | -0.6 | 0.3 |
| 9810 | flagellar associated protein | 0.0 | -0.1 | 1.2 | -0.6 | 0.7 |
| 4615 | flagellar associated protein | -0.1 | -0.1 | 1.0 | -0.7 | 0.3 |
| 8981 | flagellar associated protein | -0.1 | -0.1 | 1.6 | -0.7 | 1.0 |
| 8528 | flagellar associated protein | -0.2 | -0.1 | 1.2 | -0.8 | 0.8 |
| 8142 | flagellar associated protein | -0.2 | -0.1 | 1.3 | -0.8 | 0.7 |
| 7321 | flagellar associated protein | -0.3 | -0.1 | 1.1 | -1.0 | 0.6 |
| 12944 | flagellar central pair-associated protein | -0.2 | -0.1 | 0.7 | -1.3 | 0.0 |
| 9330 | flagellar outer dynein arm light chain 2 | 0.0 | -0.1 | 1.2 | -0.3 | 0.1 |
| 959 | flagellar outer dynein arm-docking complex protein 1 | 0.0 | -0.1 | 1.3 | -0.9 | 0.7 |
| 4213 | flagellar radial spoke protein | 0.1 | -0.1 | 1.7 | -0.7 | 0.8 |
| 4937 | fructose- - chloroplastic-like | 0.0 | -0.1 | 1.2 | -0.1 | 0.1 |
| 6106 | hypothetical 02 | 0.0 | -0.1 | 1.1 | -0.6 | 0.8 |
| 18678 | hypothetical chloroplast rf1 | 3.0 | -0.1 | -3.7 | -0.3 | 2.0 |
| 3011 | interferon-induced guanylate-binding | 0.0 | -0.1 | 2.2 | 0.0 | 1.6 |
| 952 | large subunit ribosomal RNA (rrnL), plastid | 2.1 | -0.1 | -4.4 | -1.6 | 3.2 |
| 3702 | low quality protein: tubulin polyglutamylase ttll6-like | -0.2 | -0.1 | -0.2 | -1.1 | -0.2 |
| 4697 | maltose transporter 1 MEX1 | -0.5 | -0.1 | 1.0 | 0.1 | 0.0 |
| 2177 | n-acetylglucosaminylphosphatidylinositol de-n-acetylase protein | 0.3 | -0.1 | 1.9 | -0.1 | 0.9 |
| 3615 | nadh-cytochrome b5 reductase | -0.1 | -0.1 | 1.2 | -0.1 | 0.2 |
| 269 | nadh-cytochrome b5 reductase-like protein | 0.0 | -0.1 | 1.1 | 0.0 | 0.3 |
| 8796 | outer dynein arm-docking complex subunit 3 | -0.4 | -0.1 | 1.4 | -0.8 | 0.5 |
| 6078 | peptidyl-prolyl cis-trans isomerase | -0.2 | -0.1 | 1.1 | -0.6 | 0.5 |
| 16257 | photosystem ii subunit B psbB | 1.2 | -0.1 | -3.1 | -0.4 | 1.8 |
| 2701 | plac8 domain-containing protein | -0.5 | -0.1 | 1.2 | -0.5 | 0.3 |
| 5126 | probable lactoylglutathione chloroplast-like | 0.0 | -0.1 | 1.0 | 0.3 | 0.2 |
| 16314 | protein required for templated centriole assembly | -0.3 | -0.1 | 1.3 | 0.0 | 0.6 |
| 9731 | rad51 recombination protein | 0.1 | -0.1 | 3.7 | -0.2 | 3.9 |
| 6161 | radial spoke protein 9 | 0.1 | -0.1 | 1.5 | -0.7 | 0.7 |
| 15855 | retrotransposon ty3-gypsy subclass | -0.1 | -0.1 | -0.3 | -0.6 | -1.1 |
| 2735 | rna binding protein | -0.2 | -0.1 | 1.1 | -0.5 | 0.9 |
| 9288 | snf2 super family | -0.4 | -0.1 | 1.0 | -0.1 | 0.5 |
| 2332 | spermatogenesis associated 4 | -0.2 | -0.1 | 1.2 | -0.9 | 0.5 |
| 3693 | strawberry notch-like protein | -0.5 | -0.1 | 1.0 | -0.1 | 0.7 |
| 3139 | thioredoxin domain-containing protein 5-like | 0.0 | -0.1 | 1.2 | -0.2 | 0.3 |
| 2279 | trafficking protein particle complex subunit 6b | -0.4 | -0.1 | 1.0 | 0.1 | 0.2 |
| 10562 | translation factor for chloroplast psbc mrna | -0.2 | -0.1 | -0.5 | 0.1 | -1.0 |
| 4150 | udp-glucose dehydrogenase | 0.1 | -0.1 | 1.1 | -0.3 | 0.1 |
| 10918 | uracil-dna glycosylase | -0.1 | -0.1 | 1.6 | 0.5 | 0.9 |
| 5578 | wd repeat domain 78 | -0.3 | -0.1 | 1.3 | -0.5 | 0.4 |
| 16079 | wsc-domain-containing protein | -0.3 | -0.1 | -1.0 | -0.3 | -1.1 |
| 8913 | 1 domain-containing protein | -0.1 | -0.2 | 1.2 | -0.4 | -0.2 |
| 25 | 18S, ITS, 26S rRNA | 2.2 | -0.2 | -4.8 | -1.0 | 3.7 |
| 6685 | adenylate guanylate cyclase | -0.4 | -0.2 | -0.6 | -0.4 | -1.0 |
| 5987 | armadillo repeat-containing protein 6 | 0.3 | -0.2 | 1.1 | -0.1 | 0.2 |
| 7712 | bah-phd domain-containing protein | 0.1 | -0.2 | 1.1 | -0.3 | 0.9 |
| 1341 | centrosomal protein poc5 | -0.4 | -0.2 | 1.6 | -0.3 | 1.3 |
| 1473 | citrate synthase | 0.0 | -0.2 | 1.3 | -0.3 | 0.4 |
| 2342 | clusterin-associated protein 1 | -0.2 | -0.2 | 1.3 | -0.4 | 0.3 |
| 786 | component of oligomeric golgi complex 5 | 0.0 | -0.2 | 1.0 | 0.0 | -0.2 |
| 4183 | cytochrome b5 domain-containing protein 1-like | -0.1 | -0.2 | 1.4 | -0.7 | 0.9 |
| 1845 | cytosolic phosphoglucose isomerase | 0.0 | -0.2 | 1.1 | 0.0 | 0.0 |
| 2549 | dna replication factor c complex subunit 2 | -0.1 | -0.2 | 1.0 | 0.2 | 0.5 |
| 10623 | dna replication licensing factor mcm9-like | -0.2 | -0.2 | 1.2 | -0.2 | 0.9 |
| 9996 | dna-binding horma domain-containing protein | 0.1 | -0.2 | 2.0 | -0.7 | 1.9 |
| 6687 | DnaJ-like protein | 0.1 | -0.2 | 0.5 | 1.2 | -0.8 |
| 5969 | dpy-30 like protein | -0.2 | -0.2 | 1.1 | -0.7 | 0.4 |
| 8018 | er-derived vesicles protein erv14 | -0.3 | -0.2 | 1.0 | -0.2 | 0.4 |
| 34 | es2 protein | -0.2 | -0.2 | 1.1 | 0.1 | -0.3 |
| 12273 | fatty acid desaturase | 0.1 | -0.2 | 1.1 | 0.1 | 0.1 |
| 7256 | flagellar associated protein | -0.2 | -0.2 | 1.3 | -0.5 | 0.5 |
| 9310 | flagellar associated protein | -0.4 | -0.2 | 1.7 | -0.6 | 0.6 |
| 5145 | flagellar associated protein | 0.1 | -0.2 | 1.3 | -0.6 | 0.5 |
| 6215 | flagellar associated protein | -0.2 | -0.2 | 1.1 | -0.6 | 0.5 |
| 1921 | flagellar associated protein | 0.2 | -0.2 | 1.5 | -0.7 | 0.8 |
| 7555 | flagellar associated protein | -0.1 | -0.2 | 1.4 | -0.8 | 0.7 |
| 2965 | flagellar associated protein | -0.2 | -0.2 | 1.0 | -0.8 | 0.3 |
| 2409 | flagellar associated protein | -0.4 | -0.2 | 1.4 | -0.8 | 0.6 |
| 8089 | flagellar associated protein | 0.3 | -0.2 | 1.2 | -0.9 | 0.7 |
| 4778 | flagellar associated protein | -0.1 | -0.2 | 1.2 | -0.9 | 0.7 |
| 2455 | flagellar associated protein | -0.1 | -0.2 | 1.0 | -1.0 | 0.3 |
| 11281 | flagellar associated protein | -0.2 | -0.2 | 1.2 | -1.1 | 0.9 |
| 8478 | flagellar central pair-associated protein | -0.1 | -0.2 | 0.4 | -1.2 | -0.1 |
| 4490 | flagellar outer dynein arm intermediate chain 2 | 0.1 | -0.2 | 1.4 | -0.5 | 0.5 |
| 4912 | flagellar protofilament ribbon protein | 0.2 | -0.2 | 1.6 | -0.7 | 0.7 |
| 840 | formate nitrite transporter | -0.2 | -0.2 | 1.2 | -0.1 | -0.1 |
| 5485 | gamma tubulin | -0.1 | -0.2 | 1.1 | 0.2 | 0.6 |
| 3201 | high mobility group protein | 0.1 | -0.2 | 1.3 | -0.4 | 0.4 |
| 691 | high-mobility protein | 0.0 | -0.2 | 1.2 | -0.3 | 0.4 |
| 3280 | iq and aaa domain-containing protein 1 | -0.1 | -0.2 | 1.1 | -0.6 | 0.3 |
| 12924 | kinase-like protein | 0.1 | -0.2 | -0.3 | -0.4 | -1.1 |
| 14343 | kinesin-like protein | -0.4 | -0.2 | 0.2 | -1.1 | 0.8 |
| 4582 | leucine-rich repeat-containing protein 48-like | 0.2 | -0.2 | 1.3 | -0.9 | 0.8 |
| 1428 | light lc8-type 1 | 0.2 | -0.2 | 1.4 | -0.4 | 0.8 |
| 2534 | lipoxygenase homology domain-containing protein 1 | -0.3 | -0.2 | 1.2 | -0.6 | 1.2 |
| 11032 | meckelin-like | 0.0 | -0.2 | 3.0 | -0.2 | 3.4 |
| 12580 | morn domain-containing protein | -0.1 | -0.2 | 1.5 | -0.9 | 1.0 |
| 7065 | nucleoside diphosphate kinase homolog 5-like | -0.1 | -0.2 | 1.2 | -1.1 | 0.6 |
| 1317 | nucleosome binding protein | -0.1 | -0.2 | 1.6 | -0.6 | 0.9 |
| 2687 | peptidyl-prolyl cis-trans parvulin-type | 0.0 | -0.2 | 1.3 | 0.0 | 0.6 |
| 828 | phosphoglycerate mutase-like protein | -0.1 | -0.2 | 1.4 | -0.5 | 0.5 |
| 2129 | plastid division protein ftsZ | -0.4 | -0.2 | 3.8 | -0.2 | 4.2 |
| 1513 | polyubiquitin | 0.1 | -0.2 | 1.1 | 0.1 | -0.2 |
| 10498 | protein cypro4 | -0.1 | -0.2 | 1.3 | -0.2 | 0.7 |
| 9725 | protein kinase domain containing protein | -0.5 | -0.2 | 0.7 | -1.3 | 0.4 |
| 12825 | protein kinase family | -0.2 | -0.2 | 3.1 | -0.8 | 3.0 |
| 1765 | radial spoke protein 16 | 0.0 | -0.2 | 1.6 | -0.9 | 0.7 |
| 13123 | retrotransposon ty3-gypsy subclass | -0.1 | -0.2 | -0.4 | -0.3 | -1.2 |
| 1837 | short-chain dehydrogenase reductase sdr | 0.0 | -0.2 | 1.1 | 0.1 | 0.1 |
| 5588 | snf2 family chromodomain-helicase protein | -0.1 | -0.2 | -0.2 | 0.2 | -1.1 |
| 156 | spx domain-containing protein | -0.3 | -0.2 | 0.0 | -0.1 | -1.0 |
| 11498 | tbc1 domain family member 20 | 0.0 | -0.2 | -0.6 | -0.6 | -1.0 |
| 1164 | tektin 2 | -0.1 | -0.2 | 1.2 | -0.9 | 0.9 |
| 5953 | tetratricopeptide repeat protein 21b | 0.0 | -0.2 | 1.1 | -0.6 | 0.4 |
| 2232 | tetratricopeptide repeat protein 30a | 0.1 | -0.2 | 1.1 | -0.5 | 0.6 |
| 8581 | thioredoxin-like protein | 0.0 | -0.2 | 1.3 | -0.4 | 1.4 |
| 3153 | timeless-interacting protein | -0.4 | -0.2 | 2.0 | -0.4 | 1.8 |
| 10449 | transcription initiation factor tfiid subunit d10 | -0.2 | -0.2 | 1.1 | 0.2 | -0.3 |
| 13045 | transmembrane protein 194a-like | -0.5 | -0.2 | 1.4 | 0.1 | 0.5 |
| 554 | tubulin-specific chaperone a | -0.1 | -0.2 | 1.9 | -0.4 | 1.8 |
| 1351 | upf0468 protein c16orf80 homolog | 0.3 | -0.2 | 1.5 | -1.0 | 0.9 |
| 3100 | upf0490 protein c1orf201 homolog | -0.1 | -0.2 | 1.1 | -0.4 | 0.4 |
| 8117 | ankyrin repeat protein | -0.7 | -0.3 | 1.4 | -1.0 | 1.3 |
| 18414 | atp synthase cf0 a subunit | 1.2 | -0.3 | -3.5 | -0.1 | 2.2 |
| 4132 | axonemal dynein light chain p33 | 0.0 | -0.3 | 1.2 | -0.7 | 0.5 |
| 6472 | brain protein 44-like protein | -0.3 | -0.3 | 1.3 | 0.0 | 0.0 |
| 248 | c14orf179 homolog | -0.3 | -0.3 | 1.2 | -0.3 | 0.6 |
| 9603 | CENPE type kinesin-like protein | -0.1 | -0.3 | 3.1 | -0.8 | 3.2 |
| 5421 | centrosomal protein of 290 kda | 0.0 | -0.3 | 1.2 | -0.9 | 0.8 |
| 11403 | centrosomal protein of 76 kda | -0.4 | -0.3 | 1.9 | -0.6 | 2.1 |
| 16176 | condensin-2 complex subunit d3-like | -0.1 | -0.3 | 1.4 | -0.2 | 1.2 |
| 9513 | condensin-2 complex subunit g2 | -0.4 | -0.3 | 1.7 | -0.2 | 1.6 |
| 4452 | cytoplasmic dynein 2 light intermediate chain 1-like | -0.1 | -0.3 | 1.0 | -0.7 | 0.3 |
| 10354 | dihydrolipoyllysine-residue succinyltransferase component of 2-oxoglutarate dehydrogenase complex 2 | 0.0 | -0.3 | 1.0 | -0.1 | 0.0 |
| 6032 | dnaj homolog subfamily c member 27 | 0.0 | -0.3 | 1.0 | -0.7 | -0.1 |
| 2830 | dynamin-related gtpase | -0.4 | -0.3 | 2.2 | -0.2 | 1.6 |
| 2163 | dynein heavy chain | -0.2 | -0.3 | 0.1 | -1.0 | -0.1 |
| 2107 | dynein heavy chain axonemal | -0.2 | -0.3 | 0.4 | -1.1 | 0.2 |
| 359 | dynein light chain | 0.1 | -0.3 | 1.4 | -0.8 | 0.7 |
| 4251 | dynein light chain roadblock-type 2 | 0.0 | -0.3 | 1.6 | -0.7 | 0.9 |
| 11405 | e3 ubiquitin-protein ligase rfwd3-like | 0.0 | -0.3 | 2.4 | -0.6 | 2.3 |
| 6897 | fibrillin-like | -0.1 | -0.3 | 3.2 | -0.6 | 3.0 |
| 3041 | flagellar associated protein | -0.1 | -0.3 | 1.3 | -0.6 | 0.5 |
| 12007 | flagellar associated protein | -0.2 | -0.3 | 1.2 | -0.9 | 0.5 |
| 6672 | flagellar associated protein | -0.2 | -0.3 | 1.1 | -0.9 | 0.4 |
| 3031 | flagellar associated protein | -0.1 | -0.3 | 1.2 | -0.9 | 0.6 |
| 252 | flagellar associated protein | 0.0 | -0.3 | 1.7 | -1.0 | 1.1 |
| 4046 | flagellar associated protein | 0.0 | -0.3 | 1.6 | -1.1 | 0.8 |
| 7477 | flagellar associated protein | -0.1 | -0.3 | 1.7 | -1.1 | 1.0 |
| 731 | flagellar associated protein | 0.0 | -0.3 | 1.7 | -1.2 | 0.9 |
| 1200 | flagellar basal body pacrg-like protein | 0.1 | -0.3 | 1.6 | -0.7 | 0.8 |
| 12364 | formin-homology 2 domain-containing protein | 0.0 | -0.3 | 0.9 | -0.6 | 1.2 |
| 2662 | glycosyltransferase-like protein | -0.6 | -0.3 | 1.1 | -0.5 | 1.4 |
| 1218 | growth arrest-specific protein 8 | -0.1 | -0.3 | 1.4 | -1.1 | 0.8 |
| 9837 | heme-binding protein 2-like | 0.0 | -0.3 | 1.1 | -0.4 | 0.7 |
| 4121 | histone deacetylase | -0.1 | -0.3 | 1.0 | -0.2 | 0.2 |
| 1500 | intraflagellar transport protein 172 | -0.1 | -0.3 | 1.1 | -0.8 | 0.3 |
| 4448 | intraflagellar transport protein 52 homolog | -0.1 | -0.3 | 1.1 | -0.5 | 0.2 |
| 5467 | intraflagellar transport protein 57 | 0.0 | -0.3 | 1.5 | -0.6 | 0.5 |
| 2875 | iq calmodulin-binding motif family protein | 0.0 | -0.3 | 1.3 | -0.9 | 0.8 |
| 9777 | isopentenyldiphosphate isomerase | -0.2 | -0.3 | 1.1 | -0.1 | 0.0 |
| 6035 | isoprenylcysteine carboxyl methyltransferase | 0.1 | -0.3 | 1.0 | 0.4 | 0.2 |
| 13374 | leucine-rich repeat-containing protein 61 | -0.2 | -0.3 | 1.0 | -0.7 | 0.6 |
| 5886 | microtubule plus-end binding protein eb1 | -0.1 | -0.3 | 1.1 | -0.1 | 0.4 |
| 5735 | mini-eyespot protein | 0.0 | -0.3 | 1.2 | -0.8 | 0.5 |
| 674 | molybdenum cofactor biosynthesis protein | 0.0 | -0.3 | 1.2 | 0.6 | -0.2 |
| 10003 | molybdopterin synthase catalytic subunit | 0.0 | -0.3 | 1.0 | -0.2 | 0.4 |
| 6218 | morn repeat-containing protein 3-like | -0.1 | -0.3 | 1.2 | -0.5 | 0.6 |
| 16930 | photosystem ii protein d2 | 0.2 | -0.3 | -1.9 | -0.2 | 2.6 |
| 11775 | polymorphic outer membrane protein | -0.4 | -0.3 | 1.8 | -0.4 | 2.2 |
| 4684 | pre-mrna-splicing factor | 0.0 | -0.3 | 1.0 | 0.0 | 0.1 |
| 7288 | radial spoke protein 10 | -0.2 | -0.3 | 1.3 | -0.6 | 0.5 |
| 6058 | radial spoke protein 11 | -0.1 | -0.3 | 1.4 | -0.7 | 0.5 |
| 8148 | radial spoke protein 8 | -0.1 | -0.3 | 1.3 | -0.6 | 0.5 |
| 10556 | replication protein 70 kda dna-binding subunit | 0.0 | -0.3 | 2.2 | 0.0 | 2.2 |
| 13184 | retrotransposon ty3-gypsy subclass | 0.3 | -0.3 | -0.1 | -0.3 | -1.1 |
| 18008 | reverse transcriptase | -0.3 | -0.3 | -1.5 | -1.7 | -0.7 |
| 11542 | rna binding | -0.1 | -0.3 | 1.0 | -0.3 | 0.0 |
| 11167 | serine threonine protein kinase | -0.3 | -0.3 | -0.1 | -1.1 | -0.3 |
| 4993 | signal recognition particle 68 kda | -0.1 | -0.3 | 1.0 | 0.3 | -0.2 |
| 4470 | snf2 family chromodomain-helicase protein | -0.5 | -0.3 | -0.1 | 0.0 | -1.4 |
| 11652 | sprt-like metalloprotease | 0.0 | -0.3 | 1.2 | -0.5 | 0.3 |
| 1935 | striated fiber assemblin | -0.1 | -0.3 | 1.3 | -0.7 | 0.3 |
| 3462 | succinoglycan biosynthesis protein | 0.0 | -0.3 | 0.8 | 1.0 | -0.1 |
| 1335 | tetratricopeptide repeat protein 26-like | -0.3 | -0.3 | 1.3 | -0.7 | 0.6 |
| 3010 | thioredoxin domain-containing protein 6 | 0.0 | -0.3 | 1.0 | -0.7 | 0.3 |
| 15507 | transferrin-like protein idi-100 | -0.4 | -0.3 | -1.0 | -1.3 | -0.1 |
| 4829 | tubby-like protein | 0.0 | -0.3 | 1.1 | -0.5 | 0.3 |
| 1777 | ulp1 protease family protein | 0.1 | -0.3 | 1.0 | -0.3 | 0.4 |
| 1851 | wd repeat domain 52 | -0.2 | -0.3 | 0.9 | -1.1 | 0.3 |
| 1968 | wd repeat-containing protein 35-like isoform 1 | -0.2 | -0.3 | 1.0 | -0.6 | 0.4 |
| 5156 | wd repeat-containing protein 65 | -0.1 | -0.3 | 0.9 | -1.0 | 0.5 |
| 1161 | wrky transcription factor 50 | -0.2 | -0.3 | 1.2 | -0.1 | 0.7 |
| 5424 | 3-ketoacyl- thiolase | 0.1 | -0.4 | 1.2 | -0.1 | 0.6 |
| 2772 | actin-depolymerizing factor | 0.1 | -0.4 | 1.1 | -0.1 | 0.2 |
| 10122 | arginine serine-rich splicing factor rsp41 | 0.0 | -0.4 | 1.2 | 0.0 | 0.3 |
| 5437 | arsenical pump-driving atpase-like | -0.1 | -0.4 | 1.0 | -0.4 | 0.6 |
| 97 | atp synthase cf1 beta subunit | 0.9 | -0.4 | -2.0 | -0.4 | 2.1 |
| 15782 | basal body protein | -0.4 | -0.4 | 1.2 | -0.1 | 0.7 |
| 4843 | calcium uptake protein mitochondrial-like | -0.4 | -0.4 | 1.4 | -0.7 | 0.8 |
| 3687 | central pair complex 1 | 0.0 | -0.4 | 1.0 | -0.9 | 0.2 |
| 3152 | centrin | -0.2 | -0.4 | 2.6 | -0.8 | 2.3 |
| 5499 | Centrin-1 [Harpegnathos saltator] | -0.1 | -0.4 | 1.2 | -0.9 | 1.0 |
| 11464 | centrosomal protein of 76 kda-like | -0.1 | -0.4 | 2.2 | -0.2 | 1.7 |
| 11035 | cf1 alpha subunit of atp synthase | 3.0 | -0.4 | -6.0 | -2.5 | 2.7 |
| 11834 | chromosome segregation-like protein | 0.2 | -0.4 | 3.5 | -1.1 | 2.8 |
| 4128 | condensin complex components subunit | -0.6 | -0.4 | 2.0 | -0.6 | 1.9 |
| 10565 | cyclin-dependent kinases regulatory subunit | -1.4 | -0.4 | 4.8 | -0.6 | 4.5 |
| 3157 | DNA topoisomerase II | -0.3 | -0.4 | 4.1 | -1.1 | 4.7 |
| 857 | extracellular response kinase | -0.1 | -0.4 | 1.7 | -0.7 | 1.3 |
| 4089 | fkbp-type peptidyl-prolyl cis-trans isomerase chloroplastic-like | -0.1 | -0.4 | 1.1 | 0.2 | 0.2 |
| 12375 | flagellar associated protein | 0.1 | -0.4 | 1.1 | -0.8 | 0.6 |
| 7714 | flagellar associated protein | 0.2 | -0.4 | 1.9 | -0.9 | 0.9 |
| 2700 | flagellar associated protein | -0.4 | -0.4 | 1.5 | -1.0 | 0.9 |
| 12287 | flagellar associated protein | -0.3 | -0.4 | 1.7 | -1.0 | 1.2 |
| 3749 | flagellar associated protein | -0.1 | -0.4 | 0.9 | -1.0 | 0.2 |
| 10179 | flagellar associated protein | -0.1 | -0.4 | 1.0 | -1.0 | 0.2 |
| 8568 | flagellar associated protein | 0.0 | -0.4 | 1.1 | -1.1 | 0.3 |
| 9233 | flagellar associated protein | -0.1 | -0.4 | 1.2 | -1.1 | 0.6 |
| 2315 | flagellar associated protein | -0.4 | -0.4 | 1.3 | -1.1 | 1.2 |
| 5745 | flagellar associated protein | -0.1 | -0.4 | 0.5 | -1.1 | 0.3 |
| 7016 | flagellar associated protein | 0.1 | -0.4 | 2.0 | -1.3 | 1.3 |
| 8396 | flagellar associated protein | 0.0 | -0.4 | 1.1 | -1.4 | 1.1 |
| 103 | flagellar inner arm dynein 1 heavy chain alpha | -0.1 | -0.4 | 0.6 | -1.3 | 0.3 |
| 14989 | flap endonuclease gen-like | 0.0 | -0.4 | 1.1 | -0.6 | 0.7 |
| 4422 | glucose-1-phosphate adenylyltransferase | -0.2 | -0.4 | 1.4 | 0.0 | 0.3 |
| 6814 | glutamyl-trna amidotransferase subunit a-like | 0.0 | -0.4 | 1.0 | -0.1 | 0.0 |
| 9919 | gtp-binding protein | -0.2 | -0.4 | 1.4 | 0.0 | 1.0 |
| 2652 | guanylyl cyclase | -0.1 | -0.4 | -0.6 | -0.4 | -1.1 |
| 5576 | histidine phosphatase superfamily protein | 0.0 | -0.4 | 1.0 | 0.0 | 0.1 |
| 6601 | histone chaperone asf1b | -0.3 | -0.4 | 1.2 | -0.3 | 0.7 |
| 9427 | hydroxypyruvate reductase | -0.1 | -0.4 | 1.1 | 0.0 | 0.2 |
| 18673 | hypothetical chloroplast rf1 | 2.5 | -0.4 | -4.1 | 0.1 | 1.8 |
| 5648 | intraflagellar transport 88 homolog | -0.2 | -0.4 | 1.2 | -0.7 | 0.5 |
| 4668 | intraflagellar transport protein 140 | -0.1 | -0.4 | 1.0 | -0.7 | 0.5 |
| 6920 | iq domain-containing protein d-like | 0.2 | -0.4 | 1.4 | -1.0 | 1.0 |
| 7137 | isochorismatase family protein | -0.1 | -0.4 | 1.0 | -0.1 | 0.1 |
| 6164 | kiaa0562 protein | 0.1 | -0.4 | 1.3 | -0.7 | 0.7 |
| 7151 | kinesin-like protein | -0.1 | -0.4 | 1.6 | -0.9 | 0.9 |
| 10729 | leucine-rich repeat-containing protein 51 isoform 1 | 0.0 | -0.4 | 0.7 | -1.2 | 0.4 |
| 7062 | molecular chaperone | -0.3 | -0.4 | 1.4 | -0.5 | 0.5 |
| 6199 | n-(5 -phosphoribosyl)anthranilate isomerase-like | -0.1 | -0.4 | 1.0 | 0.1 | -0.2 |
| 13279 | nhl repeat containing protein | -0.1 | -0.4 | -1.2 | -1.0 | -0.7 |
| 2689 | phosphatidylinositol 4-kinase | -0.2 | -0.4 | -0.2 | -0.2 | -1.0 |
| 14020 | p-loop containing nucleoside triphosphate hydrolase protein | -0.2 | -0.4 | -0.3 | 0.0 | -1.1 |
| 8131 | profilin | 0.3 | -0.4 | 1.1 | 0.1 | 0.2 |
| 1257 | protein nlrc3 | -0.1 | -0.4 | 1.8 | -1.1 | 1.2 |
| 14126 | protein tyrosine phosphatase domain protein 1 | -0.1 | -0.4 | -0.3 | -1.4 | -0.3 |
| 6235 | proteophosphoglycan ppg4 | -0.1 | -0.4 | 0.8 | -1.1 | -0.2 |
| 9687 | protofilament ribbon protein of flagellar microtubules | -0.4 | -0.4 | 1.4 | -0.5 | 1.2 |
| 5992 | protofilament ribbon protein of flagellar microtubules | 0.1 | -0.4 | 1.7 | -1.0 | 0.9 |
| 2423 | r1 protein c | -0.1 | -0.4 | 1.2 | -0.3 | 0.3 |
| 15359 | rab family protein | -0.1 | -0.4 | 0.3 | -1.1 | 0.0 |
| 5911 | radial spoke protein 17 | -0.3 | -0.4 | 1.5 | -1.1 | 0.9 |
| 5476 | radial spoke protein 2 | -0.2 | -0.4 | 1.1 | -0.7 | 0.6 |
| 143 | reticulon-like protein | 0.0 | -0.4 | 2.0 | -0.1 | 1.4 |
| 14638 | retrotransposable element tf2 155 kda protein type 1-like | 0.1 | -0.4 | -0.4 | -0.6 | -1.2 |
| 4459 | ribonuclease activity regulator protein | -0.1 | -0.4 | 1.1 | 0.0 | 0.3 |
| 18722 | ribulose bisphosphate carboxylase small subunit/chain | -0.6 | -0.4 | -0.2 | -0.9 | -1.1 |
| 15116 | serine threonine protein kinase | -0.3 | -0.4 | -1.0 | -0.5 | -0.3 |
| 6371 | sperm associated antigen 6 | 0.0 | -0.4 | 1.3 | -1.0 | 0.6 |
| 3240 | tissue specific transplantation antigen p35b | -0.2 | -0.4 | 1.0 | -0.4 | 0.2 |
| 12539 | transcription factor apetala2 | -0.1 | -0.4 | -0.2 | -0.1 | -1.2 |
| 4699 | adenylyl cyclase-associated protein | -0.2 | -0.5 | 1.3 | -0.4 | 0.7 |
| 10200 | adhesion regulating molecule 1 | 0.0 | -0.5 | 1.1 | -0.5 | 0.0 |
| 3793 | agc (camp- cgmp-dependent and protein kinase c) kinase family protein | -0.1 | -0.5 | 1.2 | -0.6 | 0.4 |
| 5619 | alanine-anticapsin ligase | -0.2 | -0.5 | 1.1 | -0.2 | 0.2 |
| 18337 | beta subunit of rna polymerase | 1.5 | -0.5 | -2.3 | -0.3 | 1.8 |
| 18366 | beta subunit of rna polymerase | 2.0 | -0.5 | -2.4 | -0.4 | 1.8 |
| 7122 | centrin 1 | 0.2 | -0.5 | 1.3 | -0.9 | 0.6 |
| 2133 | charged multivesicular body protein 5 | -0.2 | -0.5 | 1.1 | -0.7 | 0.3 |
| 8553 | circumsporozoite protein, putative | -0.3 | -0.5 | 1.8 | -1.1 | 1.1 |
| 18384 | condensin complex subunit 2-like | 0.0 | -0.5 | 2.8 | -1.1 | 2.1 |
| 3979 | cyclin-dependent kinase B | -0.3 | -0.5 | 3.0 | -0.6 | 2.8 |
| 850 | dynein heavy chain | -0.2 | -0.5 | 0.4 | -1.1 | 0.0 |
| 6990 | dynein heavy chain 7 | 0.0 | -0.5 | 0.3 | -1.0 | 0.0 |
| 620 | flagellar alpha dynein | -0.2 | -0.5 | 0.5 | -1.1 | 0.2 |
| 4903 | flagellar associated protein | 0.0 | -0.5 | 1.8 | -0.8 | 1.1 |
| 9571 | flagellar associated protein | 0.1 | -0.5 | 1.4 | -0.9 | 0.8 |
| 7626 | flagellar associated protein | 0.1 | -0.5 | 1.5 | -1.1 | 0.8 |
| 2564 | flagellar outer dynein arm heavy chain gamma | -0.2 | -0.5 | 0.4 | -1.2 | 0.0 |
| 3476 | gag-pol polyprotein | 0.0 | -0.5 | 0.3 | -1.3 | 2.2 |
| 16710 | j protein of photosystem ii | 2.0 | -0.5 | -4.1 | -1.2 | 2.3 |
| 4962 | leucine-rich repeat-containing protein 40-like | 0.1 | -0.5 | 1.2 | -0.2 | 0.6 |
| 10698 | magnesium-chelatase subunit i | -0.4 | -0.5 | 3.5 | -1.2 | 3.5 |
| 9527 | myosin heavy chain | -0.2 | -0.5 | 1.6 | -0.7 | 0.9 |
| 8422 | outer dynein arm-docking complex subunit 1 | -0.2 | -0.5 | 0.9 | -1.0 | 0.4 |
| 18573 | photosystem i assembly protein ycf4 | 2.6 | -0.5 | -4.6 | -0.8 | 2.7 |
| 5903 | pka regulatory subunit | -0.1 | -0.5 | 0.6 | -1.1 | 0.3 |
| 746 | plac8 domain-containing protein | -0.2 | -0.5 | 1.5 | -0.7 | 0.9 |
| 5914 | protein fam161a-like | -0.2 | -0.5 | 1.5 | -1.0 | 0.9 |
| 12586 | protein fantom | -0.1 | -0.5 | 1.6 | -1.0 | 1.4 |
| 2660 | pyruvate decarboxylase | 0.0 | -0.5 | 1.2 | -0.5 | 0.3 |
| 6567 | radial spoke head 1 homolog | -0.1 | -0.5 | 1.2 | -1.2 | 0.7 |
| 4069 | radial spoke protein 7 | -0.1 | -0.5 | 1.5 | -1.3 | 0.9 |
| 10135 | response regulator receiver protein | 0.0 | -0.5 | 1.1 | -0.8 | 0.5 |
| 3397 | rhodanese-like domain protein | 0.0 | -0.5 | 1.1 | 0.0 | 0.0 |
| 18588 | ribosomal protein l2 | 2.7 | -0.5 | -3.7 | -0.4 | 2.7 |
| 10890 | ribosome biogenesis gtp-binding protein | -0.2 | -0.5 | 1.0 | -0.3 | -0.2 |
| 13220 | spindle pole body protein | -0.3 | -0.5 | 1.6 | -1.3 | 1.5 |
| 5967 | transcription regulator | 0.0 | -0.5 | 1.2 | -0.3 | 0.8 |
| 11442 | type iii iodothyronine deiodinase | 0.3 | -0.5 | 1.6 | -0.5 | 0.6 |
| 10487 | ubiquitin containing protein | -0.2 | -0.5 | -0.3 | -0.8 | -1.6 |
| 6523 | ubiquitin-conjugating enzyme e2s | 0.0 | -0.5 | 2.6 | -1.1 | 2.0 |
| 13100 | von hippel-lindau disease tumor suppressor | -0.1 | -0.5 | 0.4 | -1.3 | 0.1 |
| 3604 | yellow stripe-like protein | -0.1 | -0.5 | 1.2 | -0.7 | 0.8 |
| 8990 | zeaxanthin epoxidase | -0.1 | -0.5 | -0.5 | -1.0 | -1.2 |
| 1394 | adenylate kinase | -0.1 | -0.6 | 0.9 | -1.1 | 0.2 |
| 3474 | beta-carboxyltransferase subunit β-CT | 0.0 | -0.6 | 1.1 | -0.3 | 0.1 |
| 2608 | B-type cyclin 1 | -0.2 | -0.6 | 3.0 | -0.4 | 3.1 |
| 9424 | cadmium resistance 11 protein | 0.0 | -0.6 | 2.0 | -1.0 | 1.6 |
| 4507 | ciliary protein | 0.1 | -0.6 | 1.1 | -1.0 | 0.4 |
| 6669 | cohesin subunit scc1b | -0.4 | -0.6 | 0.6 | -1.4 | 0.1 |
| 10883 | dynein heavy | -0.2 | -0.6 | 0.6 | -1.2 | 0.0 |
| 4587 | dynein heavy chain | -0.2 | -0.6 | 0.6 | -1.1 | 0.1 |
| 1129 | dynein heavy chain axonemal | -0.2 | -0.6 | 0.3 | -1.1 | -0.1 |
| 1950 | fkbp-type peptidyl-prolyl cis-trans isomerase | -0.4 | -0.6 | 1.3 | -0.4 | 0.5 |
| 5820 | flagellar associated protein | -0.2 | -0.6 | 1.3 | -0.5 | 0.3 |
| 4531 | flagellar associated protein | -0.2 | -0.6 | 1.0 | -1.0 | 0.7 |
| 11110 | flagellar associated protein | -0.3 | -0.6 | 1.3 | -1.0 | 0.9 |
| 14226 | flagellar associated protein | -0.2 | -0.6 | 0.6 | -1.1 | 0.3 |
| 6405 | flagellar associated protein | -0.3 | -0.6 | 0.9 | -1.1 | 0.6 |
| 2015 | flagellar associated protein | -0.2 | -0.6 | 0.8 | -1.2 | 0.5 |
| 2360 | flagellar associated protein | 0.1 | -0.6 | 1.5 | -1.3 | 0.9 |
| 9821 | flagellar associated protein | 0.4 | -0.6 | 1.6 | -1.4 | 1.7 |
| 9439 | hydrocephalus-inducing protein homolog | 0.2 | -0.6 | 0.7 | -1.3 | 0.1 |
| 1091 | inner dynein arm i1 intermediate chain ic97 | -0.1 | -0.6 | 1.4 | -1.2 | 0.8 |
| 12213 | jouberin | -0.2 | -0.6 | 1.4 | -1.1 | 1.6 |
| 8454 | kinesin k39 | 0.0 | -0.6 | 0.9 | -0.2 | 1.1 |
| 1829 | kinesin k39 | 0.0 | -0.6 | 3.0 | -0.8 | 3.2 |
| 6343 | kinetochore protein ndc80 homolog | -0.3 | -0.6 | 2.5 | -1.0 | 2.5 |
| 8484 | leucine-rich repeat protein | 0.0 | -0.6 | 0.3 | -1.1 | 0.2 |
| 4173 | leukotriene a-4 hydrolase | 0.0 | -0.6 | 1.0 | -0.2 | 0.5 |
| 11643 | membrane transporter | -0.4 | -0.6 | -0.4 | -1.0 | -0.6 |
| 8923 | mind kinetochore complex component nnf1 | -0.1 | -0.6 | 2.7 | -1.0 | 1.9 |
| 5233 | mitogen-activated protein | -0.1 | -0.6 | 0.7 | -1.2 | 0.6 |
| 8940 | nhl repeat containing protein | 0.2 | -0.6 | -1.2 | -0.9 | -1.3 |
| 2029 | nucleoside diphosphate kinase | -0.2 | -0.6 | 1.1 | -0.8 | 0.4 |
| 6149 | p700 apoprotein a1 of photosystem I, psaA | 2.7 | -0.6 | -5.8 | -1.6 | 3.1 |
| 2932 | peptidyl-prolyl cis-trans cyclophilin-type | 0.0 | -0.6 | 1.1 | -0.1 | 0.6 |
| 3440 | phosphoglycerate mutase | -0.1 | -0.6 | 2.9 | -1.1 | 2.6 |
| 2571 | proactivator polypeptide | 0.0 | -0.6 | 1.0 | -0.1 | 0.3 |
| 12211 | protein nlrc3-like | -0.1 | -0.6 | 1.0 | -0.9 | 0.4 |
| 3905 | protein of clr family | 0.0 | -0.6 | 1.1 | -0.1 | 0.1 |
| 18470 | ribosomal protein s11 | 2.2 | -0.6 | -2.0 | 0.2 | 2.4 |
| 18720 | ribulose bisphosphate carboxylase small subunit/chain | -0.6 | -0.6 | 0.0 | -1.0 | -1.1 |
| 118 | senescence-associated protein din1 | -0.3 | -0.6 | 0.2 | -0.5 | -1.1 |
| 11394 | sperm associated antigen 6 | 0.1 | -0.6 | 1.8 | -1.3 | 1.2 |
| 6063 | structural maintenance of chromosomes protein 4 | -0.4 | -0.6 | 3.1 | -1.1 | 3.2 |
| 10765 | zinc finger protein dzip1l | -0.1 | -0.6 | 2.3 | -1.0 | 1.9 |
| 11569 | 5-azacytidine-induced protein 1 | 0.1 | -0.7 | 2.0 | -1.5 | 2.3 |
| 6191 | ankyrin repeat-containing protein | 0.2 | -0.7 | 0.8 | -1.5 | 0.6 |
| 8618 | chloroplast division site-determinant MinE | -0.2 | -0.7 | 5.3 | -1.3 | 5.0 |
| 2995 | cortical microtubule associated protein spiral1 | 0.1 | -0.7 | 1.5 | -0.7 | 0.7 |
| 11422 | dynein heavy chain axonemal | -0.1 | -0.7 | -0.3 | -1.0 | 0.0 |
| 2514 | dynein heavy chain axonemal | -0.1 | -0.7 | 0.5 | -1.1 | 0.0 |
| 11388 | dynein-1-beta heavy flagellar inner arm i1 complex | -0.4 | -0.7 | -0.1 | -1.1 | -0.1 |
| 14748 | e3 ubiquitin-protein ligase rglg2-like | -0.3 | -0.7 | 0.9 | -1.4 | -0.3 |
| 5352 | flagellar associated protein | 0.3 | -0.7 | 1.7 | -1.4 | 1.1 |
| 8356 | flagellar associated protein | 0.1 | -0.7 | 1.8 | -1.5 | 1.2 |
| 14306 | gtp binding protein | -0.3 | -0.7 | 0.1 | -1.0 | 0.1 |
| 14318 | gualynate kinase-1 | 0.2 | -0.7 | 1.0 | -1.3 | 0.1 |
| 4919 | heavy chain 2 | -0.2 | -0.7 | 0.3 | -1.3 | 0.2 |
| 81 | histone h2a variant | 0.1 | -0.7 | 2.3 | -0.9 | 1.4 |
| 5713 | h-shippo 1 | 0.2 | -0.7 | 1.1 | -1.2 | 0.8 |
| 7532 | hydin-like protein | -0.1 | -0.7 | 0.3 | -1.3 | 0.0 |
| 13400 | inner centromere protein-like | 0.0 | -0.7 | 3.1 | -1.2 | 3.0 |
| 9041 | kinesin-like protein | 0.1 | -0.7 | 3.8 | -1.2 | 3.4 |
| 3339 | lipoxygenase homology domain-containing protein 1 | 0.1 | -0.7 | 2.0 | -1.5 | 1.7 |
| 5345 | low-co2 inducible protein | 0.0 | -0.7 | 1.3 | 0.7 | 0.0 |
| 994 | lrr and pyd domains-containing protein 12-like | -0.2 | -0.7 | 0.1 | -1.2 | -0.2 |
| 10864 | m protein repeat protein | 0.0 | -0.7 | 0.9 | -1.5 | 0.3 |
| 6411 | microtubule-associated protein asp | 0.1 | -0.7 | 3.0 | -1.2 | 2.7 |
| 8967 | myosin-like | 0.1 | -0.7 | 3.3 | -0.8 | 3.3 |
| 2297 | nhl repeat-containing protein | -0.3 | -0.7 | -1.7 | -1.2 | -1.2 |
| 5695 | p700 apoprotein a2 of photosystem i | 1.7 | -0.7 | -3.5 | -0.9 | 2.5 |
| 8934 | plant synaptotagmin | -0.1 | -0.7 | 1.4 | -0.9 | 0.7 |
| 10134 | retrotransposon ty3-gypsy subclass | -0.7 | -0.7 | -0.5 | -0.5 | -1.5 |
| 8311 | scp1-like small phosphatase 5 | -0.2 | -0.7 | 0.8 | -1.1 | 0.3 |
| 10993 | serine threonine protein kinase | -0.3 | -0.7 | 1.3 | -1.1 | 0.4 |
| 3009 | s-like rnase | -0.1 | -0.7 | 1.1 | -0.5 | 0.5 |
| 5428 | sperm flagellar protein 1-like | -0.1 | -0.7 | 1.1 | -0.7 | 0.4 |
| 10240 | subtilisin-like serine peptidase | -0.2 | -0.7 | -0.8 | -1.3 | -0.5 |
| 1856 | sucrose phosphatase | -0.2 | -0.7 | 0.8 | -1.0 | -0.1 |
| 953 | sulfolipid synthase SGD2 | -0.3 | -0.7 | 0.2 | -1.1 | -0.6 |
| 12142 | thioredoxin-like protein | 0.5 | -0.7 | 1.5 | -0.7 | 0.2 |
| 8766 | type xi myosin heavy chain | -0.2 | -0.7 | 3.2 | -1.0 | 3.5 |
| 10258 | uridine phosphorylase | 0.0 | -0.7 | 1.1 | -0.4 | 0.4 |
| 2581 | AAAP amino acid transporter 5 | -0.2 | -0.8 | 1.1 | -0.4 | 0.5 |
| 4346 | abc transporter g family member 7 | 0.0 | -0.8 | 0.1 | -0.9 | -1.0 |
| 8359 | ankyrin repeat protein | -0.2 | -0.8 | 0.7 | -1.4 | 0.4 |
| 15076 | condensin complex subunit 2-like | 0.2 | -0.8 | 3.0 | -1.6 | 3.2 |
| 8399 | condensin complex subunit 3 | -0.2 | -0.8 | 3.0 | -1.1 | 2.9 |
| 7383 | cyanate hydratase | 0.3 | -0.8 | 1.1 | -0.6 | 0.3 |
| 18712 | cytochrome c biogenesis protein ccsA | 2.2 | -0.8 | -3.2 | -0.8 | 1.7 |
| 11939 | dynamin-like | 0.1 | -0.8 | 3.1 | -1.4 | 2.3 |
| 6520 | e3 ubiquitin-protein ligase rnf8-like | -0.3 | -0.8 | 2.5 | -0.5 | 2.3 |
| 3124 | e3 ubiquitin-protein ligase xbos34 | 0.0 | -0.8 | 0.4 | -1.4 | -1.1 |
| 11158 | flagellar associated protein | -0.4 | -0.8 | 0.8 | -1.2 | 0.4 |
| 10125 | flagellar associated protein | -0.2 | -0.8 | 1.4 | -1.4 | 0.5 |
| 18682 | ftsh gene product | 2.6 | -0.8 | -3.6 | -0.9 | 2.2 |
| 11070 | glycosyltransferase family protein 47 | 0.1 | -0.8 | 1.1 | -0.4 | 0.8 |
| 6263 | hydin-like protein | -0.4 | -0.8 | 0.5 | -1.3 | 0.0 |
| 2565 | kinesin-like | 0.0 | -0.8 | 3.6 | -1.0 | 2.7 |
| 6958 | kinesin-like protein | -0.2 | -0.8 | 2.8 | -1.1 | 2.8 |
| 6194 | lipoxygenase homology domain-containing protein 1 | -0.2 | -0.8 | 2.3 | -1.4 | 2.0 |
| 13743 | mitogen-activated protein kinase-binding protein 1-like | 0.1 | -0.8 | 2.3 | -1.0 | 2.4 |
| 18718 | orf435 gene product | 2.8 | -0.8 | -4.4 | -0.9 | 2.5 |
| 5567 | p25-alpha-domain-containing flagellar associated protein | 0.3 | -0.8 | 2.8 | -1.7 | 2.5 |
| 3924 | peroxisomal membrane protein | 0.0 | -0.8 | 1.1 | -0.5 | 0.5 |
| 18679 | photosystem ii protein v | 2.3 | -0.8 | -4.3 | -0.9 | 1.8 |
| 1558 | protein phosphatase 2c 57 | -0.1 | -0.8 | 1.6 | -0.7 | 0.6 |
| 3960 | protein-l-isoaspartate o-methyltransferase domain protein 1 | 0.0 | -0.8 | 0.8 | -1.3 | 0.3 |
| 4259 | RING-finger-containing protein | -0.2 | -0.8 | 2.6 | -1.1 | 2.2 |
| 18721 | rubisco activase | -0.2 | -0.8 | 0.0 | -0.6 | -1.1 |
| 6392 | serine glyoxylate aminotransferase | -0.1 | -0.8 | 1.3 | -0.1 | 0.1 |
| 6576 | serine threonine-specific protein kinase | -0.1 | -0.8 | -0.3 | -0.2 | -1.0 |
| 7002 | subunit 1 of anaphase promoting complex | 0.2 | -0.8 | 4.6 | -1.5 | 4.3 |
| 5113 | trehalase-like protein | 0.4 | -0.8 | 1.1 | -0.3 | 0.0 |
| 6492 | white-brown-complex abc transporter family | -0.2 | -0.8 | 1.4 | 0.3 | 1.4 |
| 6639 | 2og-fe oxygenase family pro | 0.0 | -0.9 | 1.1 | -0.5 | 1.0 |
| 16024 | abnormal spindle-like microcephaly-associated protein homolog | -0.1 | -0.9 | 2.5 | -1.4 | 2.7 |
| 17412 | asp (abnormal spindle)- microcephaly associated | 0.2 | -0.9 | 2.4 | -1.1 | 3.0 |
| 18532 | beta subunit of rna polymerase | 2.7 | -0.9 | -4.1 | -0.6 | 1.9 |
| 18027 | beta subunit of rna polymerase | 2.0 | -0.9 | -2.9 | -0.8 | 1.6 |
| 2400 | cell cycle regulated microtubule associated protein | 0.1 | -0.9 | 3.6 | -1.0 | 3.5 |
| 18708 | cema gene product | 2.6 | -0.9 | -4.1 | -0.7 | 2.4 |
| 245 | centrosomal protein of 89 kda-like | -0.2 | -0.9 | 2.5 | -1.6 | 2.0 |
| 12128 | coenzyme q-binding protein | -0.2 | -0.9 | 1.1 | -1.5 | 0.6 |
| 9012 | dna (cytosine-5-)-methyltransferase | -0.2 | -0.9 | 0.1 | -1.0 | -0.3 |
| 272 | dynein heavy chain | -0.5 | -0.9 | 0.2 | -1.1 | -0.1 |
| 14602 | fe-s oxidoreductase | -0.3 | -0.9 | 1.8 | -1.2 | 1.8 |
| 11377 | glutathione s-transferase | 0.1 | -0.9 | 1.2 | -0.3 | 0.6 |
| 18058 | group ii intron-encoded protein | 2.6 | -0.9 | -3.9 | -0.9 | 2.1 |
| 15681 | keratin associated protein 5-7 | 0.2 | -0.9 | 2.3 | -0.9 | 2.3 |
| 2124 | kinesin-like calmodulin-binding protein | -0.1 | -0.9 | 1.3 | -1.0 | 0.6 |
| 4767 | kinesin-like protein kif23-like | 0.0 | -0.9 | 2.3 | -0.9 | 2.4 |
| 6027 | methyltransferase type 11 | 0.1 | -0.9 | 0.9 | -1.0 | 0.2 |
| 10642 | pathogenesis-related genes transcriptional activator | -0.1 | -0.9 | 0.9 | -1.3 | 0.9 |
| 237 | plastid terminal oxidase | 0.1 | -0.9 | 1.1 | -1.0 | 0.7 |
| 5175 | protein kinase wee1 | -0.1 | -0.9 | 2.4 | -0.9 | 1.9 |
| 12 | rubisco activase | 0.1 | -0.9 | 1.0 | -0.4 | 0.2 |
| 7546 | sam dependent carboxyl methyltransferase | 0.0 | -0.9 | 0.5 | -1.2 | 0.1 |
| 5566 | transcriptional coactivator p15 | -0.3 | -0.9 | 0.3 | -1.1 | -0.4 |
| 9187 | uridylate kinase | -0.1 | -0.9 | 0.5 | -1.1 | 0.6 |
| 10564 | binding protein | 0.2 | -1.0 | 0.7 | -1.2 | 0.5 |
| 3508 | B-type cyclin 2 | 0.0 | -1.0 | 6.0 | -1.7 | 6.0 |
| 3325 | condensin complex subunit 1 | -0.2 | -1.0 | 2.8 | -1.3 | 2.8 |
| 7659 | cyclic nucleotide dependent protein kinase | -0.2 | -1.0 | 0.5 | -1.6 | -0.2 |
| 18650 | cytochrome c heme attachment protein | 2.4 | -1.0 | -3.8 | -1.1 | 2.3 |
| 290 | delta-aminolevulinic acid dehydratase | 0.0 | -1.0 | 1.1 | -0.6 | 0.0 |
| 10026 | dual specificity protein kinase pyk2 | 0.2 | -1.0 | 1.0 | -1.0 | 0.1 |
| 13349 | dynamin-related gtpase | -0.4 | -1.0 | 3.0 | -1.5 | 2.7 |
| 10361 | flagellar associated protein | -0.3 | -1.0 | 0.8 | -1.0 | 0.5 |
| 3639 | flagellar associated protein | -0.1 | -1.0 | 1.7 | -1.7 | 1.2 |
| 778 | glycine cleavage t protein | 0.1 | -1.0 | 1.3 | -0.7 | 0.5 |
| 3810 | guanylate cyclase | -0.2 | -1.0 | -0.4 | -1.4 | -1.2 |
| 5671 | ist1-like protein | -0.2 | -1.0 | -0.4 | -1.3 | -1.0 |
| 12750 | kinesin family member | -0.4 | -1.0 | 3.4 | -1.2 | 3.3 |
| 1585 | kinesin-like, myosin-like | -0.2 | -1.0 | 3.5 | -1.6 | 3.2 |
| 10648 | leucine rich repeat family protein | -0.1 | -1.0 | 1.1 | -0.8 | 0.5 |
| 5218 | pentapeptide repeat family protein | 0.0 | -1.0 | 1.0 | -0.7 | 0.5 |
| 5782 | peroxisomal biogenesis factor 11 family protein | -0.1 | -1.0 | 1.6 | -1.0 | 1.0 |
| 11045 | plastid lipid associated protein | 0.0 | -1.0 | 1.6 | -1.4 | 1.1 |
| 6604 | possible site-specific dna-methyltransferase | -0.1 | -1.0 | 0.4 | -1.2 | 0.2 |
| 15127 | proteophosphoglycan ppg1 | 0.3 | -1.0 | 0.4 | -1.2 | -0.1 |
| 3628 | PsbP-like protein | -0.1 | -1.0 | 1.2 | -0.5 | 0.5 |
| 9722 | putative protein kinase | 0.0 | -1.0 | 2.1 | -1.4 | 1.9 |
| 10195 | regulator of chromosome condensation rcc1 | -0.3 | -1.0 | -0.8 | -1.2 | -0.5 |
| 400 | repeat-containing protein a_01 | -0.2 | -1.0 | 0.0 | -1.1 | -0.7 |
| 18676 | ribosomal protein s3 | 2.8 | -1.0 | -3.7 | -0.8 | 2.4 |
| 18428 | ribosomal protein s7 | 3.3 | -1.0 | -4.5 | -0.9 | 2.5 |
| 2335 | rna binding protein | 0.1 | -1.0 | 1.4 | -0.3 | 0.5 |
| 9972 | RNA recognition motif superfamily protein | 0.2 | -1.0 | -1.3 | 0.2 | -2.8 |
| 6248 | starch branching enzyme 1 | 0.0 | -1.0 | 0.5 | -1.3 | 0.4 |
| 7215 | tropomyosin-like | 0.3 | -1.0 | 3.7 | -1.7 | 3.8 |
| 6514 | ankyrin repeat domain-containing protein | -0.3 | -1.1 | 1.3 | -1.5 | 1.1 |
| 14476 | antagonist of mitotic exit network protein 1-like | 0.4 | -1.1 | 1.1 | -1.1 | 1.0 |
| 10235 | btb poz and math domain-containing protein 2-like | -0.2 | -1.1 | 1.3 | -1.4 | 0.3 |
| 5205 | chloroplast srp43 cao subunit of signal recognition particle | -0.3 | -1.1 | 1.1 | -0.7 | -0.1 |
| 9944 | f-box lrr-repeat protein 14 | -0.2 | -1.1 | 1.1 | -0.9 | 0.4 |
| 6336 | flagellar autonomy 2 NIMA family kinase | -0.1 | -1.1 | 1.8 | -1.3 | 1.2 |
| 1621 | heme oxygenase | -0.1 | -1.1 | 1.2 | -0.9 | 0.7 |
| 4349 | isoamylase chloroplastic-like | -0.3 | -1.1 | 0.9 | -1.2 | 0.3 |
| 12754 | polynucleotide kinase- 3 -phosphatase | -0.3 | -1.1 | 0.6 | -1.5 | -0.1 |
| 3657 | probable Rubisco large subunit N-methyltransferase | 0.1 | -1.1 | 1.0 | -1.0 | 0.4 |
| 13741 | protein fam186a-like | -0.2 | -1.1 | 0.9 | -1.5 | 0.5 |
| 5157 | ubiquitin thioesterase otu1 | 0.1 | -1.1 | 1.2 | -0.6 | 0.6 |
| 10271 | alpha-2-macroglobulin domain-containing protein | -0.2 | -1.2 | -0.1 | -1.2 | -0.6 |
| 15557 | ankyrin repeat protein | 0.0 | -1.2 | -0.2 | -1.1 | -0.2 |
| 1867 | chlorophyll a oxygenase | -0.1 | -1.2 | 0.2 | -1.1 | -0.8 |
| 5541 | flagellar associated protein | 0.0 | -1.2 | 1.8 | -1.7 | 1.5 |
| 11488 | gtp binding protein 2 | 0.1 | -1.2 | 0.7 | -1.4 | 0.0 |
| 4922 | methylisocitrate lyase | 0.0 | -1.2 | 1.4 | -0.4 | 0.7 |
| 15881 | o-methyltransferase family protein | -0.1 | -1.2 | 0.1 | -1.1 | 0.0 |
| 18206 | photosystem ii protein Z psbZ | 3.0 | -1.2 | -3.3 | 0.0 | 2.4 |
| 6803 | pinus taeda anonymous locus 0_14804_01 genomic sequence | -0.1 | -1.2 | -0.1 | -1.4 | -0.5 |
| 1922 | plastidic glucose transporter 4 | -0.2 | -1.2 | 1.0 | -1.0 | -0.1 |
| 3848 | proteophosphoglycan ppg4 | 0.0 | -1.2 | 0.0 | -1.1 | -0.5 |
| 8816 | ribosomal protein s2 | 1.1 | -1.2 | -2.0 | -1.0 | -0.7 |
| 18613 | ribosomal protein s9 | 2.9 | -1.2 | -4.5 | -0.7 | 2.5 |
| 579 | wd-40 repeat protein | 0.0 | -1.2 | -0.2 | -1.0 | -1.1 |
| 1793 | 3 -cyclic-nucleotide phosphodiesterase | -0.1 | -1.3 | 0.0 | -1.1 | -0.6 |
| 4916 | acyl- oxidase | -0.1 | -1.3 | 0.2 | -1.3 | 0.3 |
| 7553 | flagellar associated protein, STOP domain-containing | 0.1 | -1.3 | 2.4 | -1.9 | 1.8 |
| 2486 | lysosomal pro-x | -0.1 | -1.3 | 0.2 | -1.2 | -0.3 |
| 1135 | major facilitator superfamily | -0.3 | -1.3 | 0.2 | -1.1 | -1.2 |
| 16811 | phosphoglycerate mutase | -0.3 | -1.3 | 0.4 | -2.2 | 0.5 |
| 18581 | ribosomal protein s14 | 2.8 | -1.3 | -4.4 | -0.5 | 2.7 |
| 6690 | universal stress protein | -0.1 | -1.3 | 0.4 | -1.1 | 0.5 |
| 192 | alkyl hydroperoxide reductase thiol specific antioxidant mal allergen | 0.3 | -1.4 | 1.2 | -0.7 | 0.0 |
| 7216 | aspartate aminotransferase | 0.1 | -1.4 | 0.5 | -1.2 | 0.6 |
| 4965 | cell cycle switch protein CCS52A | -0.4 | -1.4 | 1.3 | -1.8 | 1.2 |
| 10294 | flavin reductase domain protein fmn-binding | -0.1 | -1.4 | 0.7 | -1.4 | 0.2 |
| 651 | kinesin-like | 0.0 | -1.4 | 3.6 | -2.4 | 3.9 |
| 3773 | kyphoscoliosis peptidase-like | 0.0 | -1.4 | 1.0 | -1.0 | 0.3 |
| 3277 | Mec-17 homolog | -0.2 | -1.4 | 1.3 | -1.6 | 1.4 |
| 2457 | microtubule-associated protein | 0.0 | -1.4 | 4.0 | -1.9 | 3.7 |
| 91 | oxygen-evolving enhancer protein 3 | -0.1 | -1.4 | 0.9 | -1.0 | -0.3 |
| 5110 | plac8 superfamily protein | -0.2 | -1.4 | 0.3 | -2.3 | -0.6 |
| 15515 | rad54-like protein | 0.1 | -1.4 | 1.1 | -1.5 | 1.3 |
| 1180 | carbonic anhydrase | 0.0 | -1.5 | 1.4 | 0.0 | 0.6 |
| 2469 | dihydropyrimidine dehydrogenase | -0.2 | -1.5 | 1.7 | -1.2 | 1.5 |
| 4938 | flagellar associated protein | -0.1 | -1.5 | 0.8 | -1.3 | 0.2 |
| 6408 | GJ11255 [Drosophila virilis] | -0.3 | -1.5 | -0.5 | -1.0 | -0.2 |
| 2229 | peptidyl-prolyl cis-trans cyclophilin-type | -0.2 | -1.5 | 0.5 | -1.0 | -0.6 |
| 10636 | af134579_1arabinogalactan protein | 0.2 | -1.6 | -0.3 | -1.2 | -0.8 |
| 1425 | alanine aminotransferase | 0.0 | -1.6 | 0.9 | -1.2 | 0.2 |
| 1065 | carbonic anhydrase | -0.2 | -1.6 | 2.0 | 0.3 | 1.0 |
| 3855 | glutamyl-trna reductase | -0.1 | -1.6 | 0.3 | -1.4 | -0.9 |
| 8896 | leucine-rich repeat protein | 0.2 | -1.6 | 1.9 | -1.8 | 1.5 |
| 38 | magnesium-protoporphyrin ix monomethyl ester | 0.0 | -1.6 | 0.2 | -1.3 | -0.6 |
| 36 | major light-harvesting chlorophyll a b protein | 0.0 | -1.6 | 0.1 | -1.2 | -0.9 |
| 3168 | methylmalonate-semialdehyde dehydrogenase | 0.2 | -1.6 | 1.2 | -1.2 | 1.3 |
| 12895 | protein kinase | 0.3 | -1.6 | 0.0 | -2.4 | -0.2 |
| 7223 | rna-dependent rna polymerase family protein | 0.2 | -1.6 | 0.1 | -1.5 | -0.3 |
| 5072 | serine threonine protein kinase | -0.1 | -1.6 | 0.5 | -1.2 | 0.2 |
| 8201 | thiamine pyrophosphate tpp-binding domain-containing protein | 0.0 | -1.6 | 0.9 | -1.8 | 1.3 |
| 12394 | bzip transcription factor-like protein | 0.0 | -1.7 | 0.7 | -1.6 | 0.7 |
| 1868 | dihydroxyacetone reductase | -0.1 | -1.7 | 1.2 | -1.3 | 0.9 |
| 9532 | flagellar associated protein | 0.0 | -1.7 | 0.7 | -1.2 | -0.7 |
| 18704 | m protein of photosystem ii | 3.3 | -1.7 | -2.6 | -0.5 | 2.9 |
| 5491 | nmt1 thi5 like domain protein | -0.1 | -1.7 | 1.1 | -1.1 | 0.2 |
| 3825 | protein sym1 | -0.1 | -1.7 | 0.6 | -1.2 | 0.8 |
| 450 | protochlorophyllide reductase b | 0.0 | -1.7 | 0.8 | -1.2 | -0.4 |
| 2224 | two-component system sensor kinase | -0.1 | -1.7 | 0.2 | -1.4 | -0.6 |
| 2986 | uroporphyrinogen decarboxylase | -0.1 | -1.7 | 0.8 | -1.1 | -0.5 |
| 1794 | mitogen-activated protein kinase kinase kinase-like | -0.2 | -1.8 | 3.6 | -1.9 | 3.9 |
| 3410 | nucleic acid binding protein | -0.2 | -1.8 | 0.9 | -1.0 | -0.4 |
| 12594 | putative sexual agglutinin | -0.2 | -1.8 | -1.4 | -1.5 | -1.1 |
| 1550 | trab domain-containing | -0.1 | -1.8 | 0.9 | -1.2 | -0.4 |
| 8244 | PUB domain-containing protein | -0.1 | -1.9 | 1.3 | -1.6 | 1.2 |
| 9900 | putative nematode resistance protein HS1pro1 | 0.0 | -1.9 | 1.3 | -1.4 | 1.1 |
| 1992 | sensor protein | -0.2 | -1.9 | 0.0 | -1.4 | -0.7 |
| 5251 | isoamylase ISA | -0.1 | -2.0 | 1.8 | -1.9 | 1.2 |
| 14023 | cytosolic phosphoglucose isomerase | 0.0 | -2.1 | 0.5 | -2.6 | 1.5 |
| 10870 | exodeoxyribonuclease v | -0.3 | -2.1 | 1.1 | -1.3 | -0.2 |
| 8978 | vacuolar armadillo repeat protein | -0.4 | -2.1 | 1.1 | -1.2 | 0.0 |
| 76 | chlorophyll a-b binding protein of lhcii | 0.1 | -2.2 | 0.0 | -1.4 | -1.1 |
| 2627 | inner membrane albino3-like protein 1 | 0.1 | -2.2 | 1.2 | -1.7 | 0.2 |
| 13991 | cytosolic phosphoglucose isomerase | 0.0 | -2.3 | 1.2 | -2.7 | 1.0 |
| 15296 | hemojuvelin precursor | 0.0 | -2.5 | -0.3 | -3.2 | -0.7 |
| 188 | major light-harvesting chlorophyll a b protein | -0.1 | -2.5 | 0.1 | -2.0 | -1.0 |
| 13192 | pyruvate phosphate dikinase | -0.1 | -2.6 | 0.2 | -1.1 | 0.2 |
| 15215 | zinc finger DNA binding domain protein | 0.1 | -2.8 | 1.0 | -2.1 | -0.2 |
| 64 | major light-harvesting chlorophyll a b protein | -0.3 | -3.4 | -0.1 | -2.8 | -0.9 |
