# Supplementary material for: Interaction of Temperature and Photoperiod Increases Growth and Oil Content in the Marine Microalgae Dunaliella viridis
Source: PLoS One. 2015 May 19;10(5):e0127562. doi: 10.1371/journal.pone.0127562 (PMC4437649; doi:10.1371/journal.pone.0127562)
Supplement: S5 Table — (DOCX) [file pone.0127562.s018.docx]

**S5 Table. Transcripts differentially expressed under high temperature at 30, 40 or 54 hrs.** The list is sorted by Log_2_FC at 30 hrs: from the highest (up-regulated transcripts) to the lowest (down-regulated transcripts), all the transcripts presented in this list are differentially expressed at one or more time points.

|  |  | **Log_2_FC(35°C/25°C)** | | |
| --- | --- | --- | --- | --- |
| **Transcript** | **Description** | **30** | **40** | **54** |
| 7634 | heat shock protein hsp20 | 4.5 | 4.3 | 4.5 |
| 3349 | heat shock protein hsp20 | 3.3 | 2.7 | 2.3 |
| 304 | fructose-bisphosphate aldolase | 3.0 | 2.5 | 2.8 |
| 9193 | heat shock protein hsp20 | 2.9 | 2.3 | 3.0 |
| 1477 | peptidase S8 family domain in Kp43 proteases | 2.8 | 3.6 | 3.7 |
| 3101 | zf-dnl-domain-containing protein | 2.7 | 3.2 | 3.0 |
| 5549 | heat shock protein hsp20 | 2.7 | 2.1 | 1.8 |
| 2857 | cytochrome c oxidase subunit 2 | 2.7 | 4.5 | 4.7 |
| 5468 | heat shock protein hsp20 | 2.5 | 1.9 | 1.6 |
| 4559 | heat shock protein hsp20 | 2.4 | 2.1 | 1.1 |
| 10037 | cytochrome p450 | 2.3 | 2.1 | 2.1 |
| 2132 | RNA recognition motif superfamily protein | 2.2 | 1.2 | 1.2 |
| 2296 | glycosyl hydrolases family 32 protein | 2.2 | 2.2 | 2.9 |
| 7135 | aldo-keto reductase | 2.1 | 1.3 | 1.1 |
| 340 | macrophage migration inhibitory factor family protein | 2.0 | 0.3 | 0.3 |
| 2686 | heat shock protein hsp20 | 2.0 | 1.6 | 1.5 |
| 1869 | fasciclin domain-containing protein | 2.0 | 4.3 | 4.4 |
| 13392 | chloroplast carotene biosynthesis related protein | 2.0 | 4.3 | 5.3 |
| 64 | major light-harvesting chlorophyll a b protein | 1.9 | 0.2 | -0.3 |
| 4753 | vesicle inducing protein in plastids 1 | 1.8 | 1.9 | 1.8 |
| 12771 | SCP-like domain protein | 1.8 | 3.0 | 3.3 |
| 5707 | putative retinoblastoma-binding protein 9 serine hydrolase | 1.8 | 1.0 | 1.6 |
| 2018 | utp--glucose-1-phosphate uridylyltransferase | 1.7 | 1.6 | 1.3 |
| 1717 | low-co2 inducible protein | 1.7 | 1.6 | 1.5 |
| 4427 | SCP-like domain protein | 1.6 | 4.1 | 4.2 |
| 7600 | fasciclin domain-containing protein | 1.6 | 3.2 | 4.1 |
| 5388 | stress responsive alpha-beta barrel domain-containing protein | 1.5 | 1.2 | 0.8 |
| 188 | major light-harvesting chlorophyll a b protein | 1.5 | -0.1 | -0.3 |
| 3183 | lipase LIPG2 | 1.5 | 1.7 | 1.5 |
| 14466 | Hemerythrin-like domain containing protein | 1.5 | 3.1 | 2.6 |
| 10517 | OTU-like cysteine protease | 1.4 | 1.6 | 1.9 |
| 1 | major light-harvesting chlorophyll a b protein | 1.4 | -0.4 | -0.9 |
| 1135 | major facilitator superfamily | 1.4 | 0.6 | 0.3 |
| 5012 | histone h1-like partial | 1.4 | 1.2 | 1.3 |
| 3146 | enzyme of the cupin superfamily | 1.4 | 0.1 | -0.2 |
| 3330 | acetylhydrolase of the phospholipases A2 family | 1.4 | 1.7 | 2.0 |
| 927 | soluble acid invertase | 1.3 | 1.2 | 1.3 |
| 4182 | ribokinase | 1.3 | 1.2 | 1.1 |
| 11681 | photosystem ii manganese-stabilizing polypeptide | 1.3 | 1.7 | 2.2 |
| 606 | photosystem i reaction center subunit v | 1.3 | 0.3 | 0.1 |
| 36 | major light-harvesting chlorophyll a b protein | 1.3 | 0.4 | -0.1 |
| 2093 | heat shock protein | 1.3 | 1.2 | 0.7 |
| 3275 | cytochrome p450 | 1.3 | 1.7 | 1.7 |
| 2919 | cysteine peptidase | 1.3 | 1.2 | 1.5 |
| 12954 | 5’-nucleotidase domain containing protein | 1.3 | 2.5 | 1.7 |
| 603 | sucrose phosphatase | 1.2 | 0.8 | 0.9 |
| 9904 | SCP-like domain protein | 1.2 | 3.7 | 4.3 |
| 5492 | peptidylprolyl isomerase | 1.2 | 1.1 | 0.7 |
| 12939 | OTU-like cysteine protease | 1.2 | 1.5 | 1.6 |
| 7995 | lipase-like | 1.2 | 1.6 | 1.3 |
| 1850 | cytochrome b | 1.2 | 2.5 | 3.1 |
| 76 | chlorophyll a-b binding protein of lhcii | 1.2 | 0.2 | -0.4 |
| 13100 | von hippel-lindau disease tumor suppressor | 1.1 | 0.7 | 1.4 |
| 9503 | tetratricopeptide repeat-containing protein | 1.1 | 1.7 | 1.5 |
| 5709 | spore coat protein | 1.1 | 1.1 | 1.6 |
| 6888 | serine carboxypeptidase | 1.1 | 1.4 | 1.5 |
| 591 | redoxin domain protein | 1.1 | 0.9 | 0.8 |
| 15807 | protein kinase-like | 1.1 | 1.8 | 0.7 |
| 7133 | protein kinase domain-containing protein | 1.1 | 2.0 | 1.7 |
| 14598 | poly -binding protein | 1.1 | 0.3 | 1.1 |
| 283 | photosystem i subunit o | 1.1 | -0.1 | -0.2 |
| 621 | phosphatidylcholine-sterol acyltransferase (lecithin-cholesterol acyltransferase) | 1.1 | 1.8 | 1.7 |
| 3655 | peptidase m20 | 1.1 | 1.1 | 1.0 |
| 2024 | peptidase family protein | 1.1 | 0.9 | 0.4 |
| 11840 | mhck ef2 kinase domain family protein | 1.1 | 1.2 | 0.8 |
| 11533 | major facilitator superfamily mfs_1 | 1.1 | 1.1 | 1.2 |
| 411 | light-harvesting protein of photosystem i | 1.1 | -0.1 | -0.3 |
| 16364 | leucine-rich repeat protein | 1.1 | 1.8 | 1.6 |
| 4387 | kda class i heat shock protein 1 | 1.1 | 0.4 | -0.3 |
| 3599 | heat shock protein hsp20 | 1.1 | 1.2 | 1.4 |
| 3190 | glycosyl hydrolases family 32 protein | 1.1 | 1.2 | 1.2 |
| 14066 | GAF domain-containing protein | 1.1 | 0.8 | 1.0 |
| 1884 | family protein | 1.1 | 0.6 | 0.8 |
| 2069 | DnaJ-Hsp40 domain protein | 1.1 | 0.9 | 1.3 |
| 6311 | cysteine peptidase | 1.1 | 1.3 | 1.2 |
| 2057 | C-terminal processing peptidase family S41 | 1.1 | 1.7 | 2.1 |
| 11274 | Yippee-like protein | 1.0 | 1.8 | 2.4 |
| 11309 | SCP-2 sterol transfer family protein | 1.0 | 1.1 | 1.5 |
| 2911 | rieske (2fe-2s) domain protein | 1.0 | 1.0 | 1.2 |
| 9497 | rhodanese-domain containing protein | 1.0 | 2.4 | 2.0 |
| 15300 | probable cysteine desulfurase-like | 1.0 | 1.3 | 1.3 |
| 11479 | pleckstrin homology domain protein | 1.0 | 2.4 | 1.5 |
| 237 | plastid terminal oxidase | 1.0 | 1.6 | 1.4 |
| 10041 | photosystem ii protein d1 psbA | 1.0 | 1.1 | -0.7 |
| 16811 | phosphoglycerate mutase | 1.0 | 1.5 | 1.8 |
| 16852 | major light-harvesting chlorophyll a b protein | 1.0 | -0.2 | -0.3 |
| 2486 | lysosomal pro-x | 1.0 | 0.2 | 0.0 |
| 5345 | low-co2 inducible protein | 1.0 | 0.9 | 1.4 |
| 39 | light-harvesting protein of photosystem i | 1.0 | -0.1 | -0.2 |
| 3861 | glyoxalase bleomycin resistance dioxygenase superfamily | 1.0 | 1.0 | 1.2 |
| 6415 | glycoside-hydrolase-like protein | 1.0 | 1.0 | 1.4 |
| 6356 | glutamate receptor | 1.0 | 1.4 | 1.5 |
| 10810 | fad fmn-containing dehydrogenase | 1.0 | 1.8 | 1.3 |
| 8531 | CP12 protein | 1.0 | 1.0 | 1.6 |
| 4754 | bZIP transcription factor-like | 1.0 | 1.4 | 1.3 |
| 7275 | ankyrin domain protein ank2 | 1.0 | 1.3 | 1.2 |
| 891 | alpha-glucan water chloroplastic-like | 1.0 | 1.2 | 1.2 |
| 6171 | alpha beta fold family hydrolase | 1.0 | 1.3 | 1.1 |
| 4916 | acyl- oxidase | 1.0 | 0.9 | 1.2 |
| 7309 | uncharacterized protein | 0.9 | 0.7 | 1.4 |
| 4472 | uncharacterized protein | 0.9 | 1.0 | 0.7 |
| 1321 | transmembrane protein | 0.9 | 1.0 | 1.3 |
| 3034 | sucrose phosphatase | 0.9 | 0.9 | 1.1 |
| 6455 | subtilisin-like serine protease | 0.9 | 2.3 | 2.3 |
| 6742 | selenium-binding protein | 0.9 | 1.2 | 1.3 |
| 1314 | s-adenosyl-l-methionine-dependent methyltransferase | 0.9 | 1.4 | 1.3 |
| 7679 | oxysterol binding family protein | 0.9 | 2.6 | 1.8 |
| 12043 | o-methyltransferase family 2 | 0.9 | 1.1 | 1.0 |
| 2615 | metallo-dependent phosphatase | 0.9 | 1.3 | 1.6 |
| 15034 | meiotic recombination protein dmc1 | 0.9 | 0.8 | 1.5 |
| 14084 | malate dehydrogenase | 0.9 | 1.9 | 2.1 |
| 5357 | large subunit ribosomal RNA (rrnL6), mitochondrial | 0.9 | 1.5 | 2.6 |
| 9350 | exostoses -like 3 | 0.9 | 0.9 | 1.2 |
| 10803 | dimethylarginine dimethylaminohydrolase 1 | 0.9 | 1.1 | 1.1 |
| 12883 | cytochrome p450 | 0.9 | 1.4 | 1.4 |
| 4596 | cysteine protease | 0.9 | 1.1 | 1.9 |
| 14033 | bifunctional enoyl-CoA hydratase phosphate acetyltransferase | 0.9 | 1.3 | 1.1 |
| 7216 | aspartate aminotransferase | 0.9 | 0.6 | 1.0 |
| 11361 | act domain-containing protein 6 | 0.9 | 1.6 | 1.2 |
| 8168 | zinc finger family protein | 0.8 | 1.0 | 0.9 |
| 11696 | whole genome shotgun sequence assembly | 0.8 | 0.7 | 1.5 |
| 3484 | Ras domain containing protein | 0.8 | 1.7 | 2.0 |
| 10948 | proline dehydrogenase oxidase | 0.8 | 2.0 | 1.6 |
| 1899 | nitrite transporter nar1 | 0.8 | 0.9 | 1.1 |
| 6872 | metallophosphatase superfamily | 0.8 | 1.9 | 2.1 |
| 614 | hnh endonuclease family protein | 0.8 | 1.8 | 2.0 |
| 2044 | flagellar associated protein | 0.8 | 0.9 | 1.1 |
| 9928 | ferredoxin-nadp reductase | 0.8 | 1.0 | 1.4 |
| 10282 | ankyrin repeat-containing protein | 0.8 | 0.5 | 1.3 |
| 6298 | acyl- synthetase | 0.8 | 0.8 | 1.2 |
| 7014 | zinc finger family protein | 0.7 | 1.2 | 1.5 |
| 1434 | uncharacterized protein loc100280346 | 0.7 | 1.1 | 1.0 |
| 8511 | transmembrane protein 17 family | 0.7 | 2.0 | 1.5 |
| 516 | tpr repeat protein | 0.7 | 1.0 | 0.9 |
| 13192 | pyruvate phosphate dikinase | 0.7 | 1.4 | 1.9 |
| 2571 | proactivator polypeptide | 0.7 | 0.9 | 1.0 |
| 2195 | phytochelatin synthase | 0.7 | 0.6 | 1.0 |
| 12773 | peptidylglycine alpha-amidating monooxygenase | 0.7 | 1.1 | 0.7 |
| 10469 | nucleic acid binding protein | 0.7 | 0.1 | 1.9 |
| 11689 | MaoC-like dehydratase | 0.7 | 1.9 | 0.7 |
| 9837 | heme-binding protein 2-like | 0.7 | 1.3 | 0.2 |
| 3542 | fe-s oxidoreductase | 0.7 | 0.9 | 1.2 |
| 4006 | exostosin-like glycosyltransferase | 0.7 | 1.0 | 1.3 |
| 9934 | ethylene-responsive transcription factor | 0.7 | 2.8 | 2.7 |
| 5091 | cysteine-rich pdz-binding protein | 0.7 | 1.3 | 1.4 |
| 4138 | calcium-dependent protein kinase 28 | 0.7 | 1.2 | 1.0 |
| 5852 | at3g62770 – autophagy protein binding Phosphoinositols | 0.7 | 1.0 | 1.1 |
| 1772 | ankyrin repeat domain-containing protein 50 | 0.7 | 1.1 | 1.4 |
| 2435 | ankyrin domain protein | 0.7 | 1.1 | 1.0 |
| 3766 | aldose reductase | 0.7 | 0.6 | 1.1 |
| 7906 | act domain containing with alternative splicing forms | 0.7 | 1.0 | 1.0 |
| 1566 | ureidoglycolate hydrolase | 0.6 | 1.1 | 0.9 |
| 1777 | ulp1 protease family protein | 0.6 | 1.1 | 1.2 |
| 6175 | ubiquitin-conjugating enzyme | 0.6 | 1.3 | 1.5 |
| 5892 | tsi1-interacting protein tsip1 | 0.6 | 1.0 | 1.3 |
| 9207 | tld domain-containing protein kiaa1609 homolog | 0.6 | 1.0 | 1.0 |
| 5370 | thioredoxin | 0.6 | 1.1 | 1.1 |
| 8029 | tetratricopeptide repeat family | 0.6 | 1.5 | 1.1 |
| 10310 | starch binding domain containing possible plant origin | 0.6 | 0.7 | 1.1 |
| 15245 | sodium phosphate symporter | 0.6 | 3.8 | 4.2 |
| 17156 | SCP-like domain protein | 0.6 | 3.3 | 4.5 |
| 7790 | regulatory protein | 0.6 | 1.3 | 1.2 |
| 14428 | receptor expression-enhancing protein | 0.6 | 1.4 | 1.2 |
| 4094 | nudix hydrolase | 0.6 | 1.0 | 0.9 |
| 8387 | mitochondrial carrier protein | 0.6 | 1.2 | 1.3 |
| 5790 | metal-nicotianamine transporter ysl6 | 0.6 | 0.8 | 1.0 |
| 1240 | metallo-beta-lactamase family protein | 0.6 | 0.9 | 1.0 |
| 13201 | kelch repeat protein | 0.6 | 0.7 | 1.3 |
| 4860 | iron-sulfur cluster assembly protein | 0.6 | 0.9 | 1.4 |
| 5451 | haloacid dehalogenase-like hydrolase | 0.6 | 0.7 | 1.0 |
| 9317 | glutaryl- dehydrogenase | 0.6 | 0.8 | 1.1 |
| 2824 | formate nitrite transporter | 0.6 | 1.2 | 1.2 |
| 6984 | fact complex subunit spt16-like | 0.6 | 1.2 | 1.4 |
| 3076 | DUF2256 domain protein | 0.6 | 1.1 | 1.7 |
| 2008 | dna replication complex gins protein sld5-like | 0.6 | 1.0 | 1.3 |
| 922 | d-glycerate 3-kinase | 0.6 | 0.8 | 1.7 |
| 2826 | cysteine protease component of protease-inhibitor complex | 0.6 | 0.9 | 1.3 |
| 3661 | conserved hypothetical protein [Trypanosoma IL3000] | 0.6 | 1.4 | 1.5 |
| 3039 | chloroplast photosystem ii-associated 22 kda protein | 0.6 | 2.2 | 2.4 |
| 4513 | chloroplast DnaJ-like protein | 0.6 | 1.7 | 2.7 |
| 4223 | atp-dependent clp protease adaptor containing protein | 0.6 | 1.1 | 1.4 |
| 1702 | alpha beta fold family protein | 0.6 | 1.1 | 0.9 |
| 1425 | alanine aminotransferase | 0.6 | 0.7 | 1.2 |
| 3625 | 3-oxo-5-alpha-steroid 4-dehydrogenase 1 | 0.6 | 1.1 | 0.8 |
| 12339 | 2-polyprenyl-6-methoxyphenol hydroxylase-like oxidoreductase | 0.6 | 0.7 | 1.1 |
| 13294 | uncharacterized protein loc100839209 | 0.5 | 0.9 | 1.3 |
| 4264 | uncharacterized protein | 0.5 | 1.3 | 1.3 |
| 14041 | threonine aldolase | 0.5 | 0.9 | 1.6 |
| 2483 | RTA1-domain containing protein | 0.5 | 1.4 | 2.3 |
| 10246 | PLAC8-domain containing protein | 0.5 | 1.0 | 2.0 |
| 2692 | phosphoglycerate mutase | 0.5 | 1.4 | 1.4 |
| 3563 | myeloid leukemia | 0.5 | 1.1 | 1.2 |
| 1858 | iron-sulfur cluster scaffold-like protein | 0.5 | 1.2 | 1.9 |
| 10661 | interferon-induced gtp-binding protein mx1 | 0.5 | 1.4 | 0.9 |
| 6729 | homogentisate 1,2-dioxygenase | 0.5 | 1.2 | 1.1 |
| 12817 | guanylate cyclase | 0.5 | 1.0 | 0.9 |
| 11137 | glutaredoxin duf547 domain-containing protein | 0.5 | 0.8 | 1.2 |
| 13187 | formate nitrite transporter | 0.5 | 0.9 | 1.2 |
| 10440 | d27 protein | 0.5 | 1.1 | 0.5 |
| 4858 | cytochrome p450 | 0.5 | 1.0 | 1.2 |
| 2847 | cytochrome oxidase subunit 1 | 0.5 | 1.7 | 2.0 |
| 5049 | coiled-coil-helix-coiled-coil-helix domain protein 4 | 0.5 | 0.9 | 1.0 |
| 140 | chlorophyll a b-binding protein | 0.5 | 2.0 | 0.1 |
| 5706 | chaperone protein | 0.5 | 1.0 | 0.8 |
| 8798 | cation channel family protein | 0.5 | 1.0 | 0.8 |
| 8117 | ankyrin repeat protein | 0.5 | 1.4 | 0.5 |
| 7601 | ubiquitin carboxyl-terminal hydrolase 12 | 0.4 | 1.4 | 1.0 |
| 3642 | thioredoxin domain-containing protein 9 homolog | 0.4 | 0.9 | 1.1 |
| 17811 | TAZ zinc finger protein | 0.4 | 1.8 | 2.7 |
| 11833 | sodium- and chloride-dependent gaba transporter 3 | 0.4 | 1.5 | 2.2 |
| 2532 | rnd family transporter: niemann-pick type c1 disease protein | 0.4 | 1.0 | 1.4 |
| 7327 | oxysterol binding family protein | 0.4 | 0.8 | 1.1 |
| 9304 | malate synthase | 0.4 | 1.1 | 1.3 |
| 7050 | large subunit ribosomal RNA (rrnL5), mitochondrial | 0.4 | 1.6 | 3.8 |
| 15681 | keratin associated protein 5-7 | 0.4 | 1.1 | 0.6 |
| 4281 | k+ efflux antiporter 3 | 0.4 | 0.9 | 1.0 |
| 10184 | hect e3 ubiquitin | 0.4 | 1.2 | 1.2 |
| 8390 | dna binding protein | 0.4 | 0.6 | 1.3 |
| 7088 | cupin domain protein | 0.4 | 1.1 | 1.7 |
| 5623 | crystallin j1a | 0.4 | 1.0 | 1.1 |
| 4621 | coiled-coil domain-containing protein | 0.4 | 0.8 | 1.1 |
| 4943 | centromere kinetochore protein zw10 homolog | 0.4 | 1.1 | 0.7 |
| 6424 | ankyrin repeat-containing | 0.4 | 1.1 | 1.5 |
| 11567 | and mbr like protein | 0.4 | 0.9 | 1.5 |
| 13117 | zinc finger family-like partial | 0.3 | 1.1 | 0.3 |
| 13110 | wd40 repeat-like protein | 0.3 | 1.0 | 0.8 |
| 3306 | uncharacterized conserved protein | 0.3 | 0.9 | 1.2 |
| 14891 | protease deg15 | 0.3 | 1.1 | 0.5 |
| 5379 | minichromosome maintenance protein 10 isoform 1-like | 0.3 | 1.2 | 0.4 |
| 1413 | histone h3 | 0.3 | 1.4 | 1.1 |
| 15296 | hemojuvelin precursor | 0.3 | 1.5 | 1.3 |
| 3264 | genetic modifier | 0.3 | 1.0 | 1.0 |
| 6750 | cytochrome p450 | 0.3 | 0.6 | 1.0 |
| 6982 | calmodulin | 0.3 | 0.8 | 1.3 |
| 12707 | atp-binding cassette transporter | 0.3 | 1.1 | 1.7 |
| 5366 | 6-4 photolyase | 0.3 | 1.0 | 1.4 |
| 12639 | 50s ribosomal protein l33 | 0.3 | 0.6 | 1.1 |
| 5424 | 3-ketoacyl- thiolase | 0.3 | 0.6 | 1.4 |
| 5538 | uncharacterized protein hp_0274 | 0.2 | 0.5 | 1.1 |
| 11442 | type iii iodothyronine deiodinase | 0.2 | 0.6 | 1.2 |
| 8080 | rwp-rk domain-containing protein | 0.2 | 1.1 | 0.7 |
| 15399 | probable metal-nicotianamine transporter ysl6-like 5 | 0.2 | 1.2 | 1.0 |
| 3715 | nadp-dependent malic enzyme | 0.2 | 1.3 | 0.8 |
| 5233 | mitogen-activated protein | 0.2 | 0.8 | 1.1 |
| 2068 | mitochondrial transcription termination factor | 0.2 | 1.0 | 1.1 |
| 2862 | magnesium chelatase subunit d | 0.2 | 0.3 | 1.1 |
| 8240 | gata transcription factor | 0.2 | 0.8 | 1.7 |
| 13178 | e3 ubiquitin ligase-like protein | 0.2 | 1.3 | 0.4 |
| 6763 | carotene isomerase | 0.2 | 1.3 | 1.0 |
| 5404 | atp-dependent clp protease atp-binding subunit | 0.2 | 2.6 | 2.7 |
| 10707 | ap2 domain containing expressed | 0.2 | 0.9 | 1.1 |
| 14830 | ankyrin repeat protein | 0.2 | 1.0 | 1.9 |
| 2749 | acyl- dehydrogenase | 0.2 | 0.7 | 1.2 |
| 11222 | upf0470 protein c19orf51 homolog | 0.1 | 1.1 | 0.6 |
| 14090 | ran-binding protein 1 | 0.1 | 0.5 | 1.4 |
| 2090 | porphobilinogen deaminase | 0.1 | -1.0 | -1.1 |
| 13659 | isoform a | 0.1 | 1.0 | 0.6 |
| 16300 | guanylate kinase | 0.1 | 0.9 | 1.3 |
| 1059 | fumarate hydratase | 0.1 | 1.2 | 0.9 |
| 3925 | e3 ubiquitin-protein ligase at3g02290-like | 0.1 | 0.4 | 1.0 |
| 7448 | cytochrome cyp711 clan | 0.1 | 0.8 | 1.2 |
| 1892 | chlorophyll b reductase | 0.1 | 0.8 | 1.1 |
| 1098 | carbonic anhydrase | 0.1 | 0.6 | 1.2 |
| 6664 | uroporphyrinogen-iii decarboxylase | 0.0 | -1.0 | -1.0 |
| 4570 | sulfate abc inner membrane subunit | 0.0 | 1.3 | 0.8 |
| 9398 | short-chain dehydrogenase reductase family 42e member 1 | 0.0 | 1.2 | 1.1 |
| 5840 | o-methyltransferase | 0.0 | 1.5 | 2.6 |
| 15834 | leucine rich repeat domain protein | 0.0 | 1.2 | 0.6 |
| 1829 | kinesin k39 | 0.0 | 1.1 | 0.6 |
| 1381 | kinase-like protein | 0.0 | 0.9 | 1.6 |
| 2627 | inner membrane albino3-like protein 1 | 0.0 | -0.9 | -1.3 |
| 9522 | flavin reductase domain protein fmn-binding protein | 0.0 | 1.0 | 0.9 |
| 7518 | flagellar associated protein | 0.0 | 1.0 | 0.5 |
| 3354 | dual-specificity protein phosphatase 6 | 0.0 | 1.1 | 1.0 |
| 4506 | centrin-like protein | 0.0 | -1.1 | -1.2 |
| 12274 | catalase | 0.0 | 0.4 | 1.1 |
| 10170 | asparaginyl endopeptidase | 0.0 | 1.1 | 1.3 |
| 16218 | ankyrin repeat protein | 0.0 | 0.9 | 1.4 |
| 11251 | thioredoxin-like 4 | -0.1 | 1.1 | 1.2 |
| 7080 | rna polymerase sigma factor | -0.1 | 1.1 | 1.1 |
| 10808 | maturation stability factor for petA mRNA | -0.1 | -1.2 | -1.0 |
| 16515 | e3 ubiquitin-protein ligase rfwd3 | -0.1 | 1.2 | 0.4 |
| 5222 | conserved protein chloroplast | -0.1 | -1.1 | -1.2 |
| 9603 | CENPE type kinesin-like protein | -0.1 | 1.2 | 0.4 |
| 4762 | 12-oxophytodienoic acid reductase | -0.1 | 1.0 | 1.5 |
| 8581 | thioredoxin-like protein | -0.2 | 1.5 | 1.9 |
| 12249 | elongation factor tu | -0.2 | -0.8 | -1.5 |
| 2001 | dihydroxyacetone kinase | -0.2 | -0.9 | -1.1 |
| 8985 | chloroplast division site determinant | -0.2 | 1.2 | 0.4 |
| 3322 | carotenoid cleavage dioxygenase | -0.2 | 0.7 | 1.2 |
| 9523 | atp synthase cf0 c subunit | -0.2 | -0.4 | -1.5 |
| 2430 | ankyrin repeat and socs box protein 2 | -0.2 | 1.3 | 1.8 |
| 3504 | multidrug pheromone mdr abc transporter family | -0.3 | 0.7 | 1.7 |
| 134 | multicopper ferroxidase | -0.3 | -2.1 | -1.1 |
| 86 | immunoglobulin g-binding protein partial | -0.3 | 0.9 | 1.1 |
| 5545 | EF-Hand domain-containing thioredoxin | -0.3 | -0.9 | -1.2 |
| 2771 | aminoimidazolecarboximide ribonucleotide transformylase inosine monophosphate cyclohydrolase bifunctional enzyme | -0.3 | -0.9 | -1.1 |
| 10551 | wrky transcription factor | -0.4 | 2.2 | 0.3 |
| 14092 | protein: rab3 gtpase-activating protein catalytic subunit-like | -0.4 | 0.5 | 1.1 |
| 7946 | programmed cell death protein 6-interacting protein | -0.4 | -0.7 | -1.0 |
| 14085 | major facilitator superfamily transporter | -0.4 | 0.7 | 1.3 |
| 4767 | kinesin-like protein kif23-like | -0.4 | 1.2 | 0.2 |
| 5576 | histidine phosphatase superfamily protein | -0.4 | -1.1 | -1.2 |
| 3922 | helicase mov-10 | -0.4 | -0.8 | -1.0 |
| 568 | flavodoxin | -0.4 | -2.5 | -2.2 |
| 1868 | dihydroxyacetone reductase | -0.4 | -1.4 | -1.4 |
| 1024 | 3-isopropylmalate dehydrogenase | -0.4 | -0.9 | -1.1 |
| 748 | vasa intronic gene | -0.5 | -1.1 | -0.9 |
| 3186 | sterol desaturase | -0.5 | -1.0 | -0.7 |
| 15337 | probable signal peptidase complex subunit 1-like | -0.5 | -1.1 | -0.9 |
| 832 | plasma-membrane proton-efflux p-type atpase | -0.5 | -0.7 | -1.1 |
| 5410 | photosystem I LHC chlorophyll a/b binding protein 3 | -0.5 | -3.1 | -2.7 |
| 14449 | p53 binding protein | -0.5 | 1.2 | 0.3 |
| 2972 | n-myristoyl transferase | -0.5 | -1.0 | -1.3 |
| 460 | malate dehydrogenase | -0.5 | -1.1 | -1.0 |
| 280 | chloroplast ribosome-associated protein | -0.5 | -1.6 | -1.4 |
| 9785 | ubiquitin-conjugating enzyme | -0.6 | -1.7 | -1.3 |
| 1119 | triosephosphate isomerase | -0.6 | -1.2 | -1.0 |
| 9473 | ribosomal protein l11 methyltransferase | -0.6 | -1.0 | -1.3 |
| 1677 | flagellar associated protein | -0.6 | -0.9 | -1.1 |
| 2148 | expansin a10 | -0.6 | 0.6 | 1.0 |
| 2456 | ccaat enhancer-binding protein zeta | -0.6 | -0.9 | -1.0 |
| 2841 | YGGT-domain-containing protein | -0.7 | -1.2 | -1.2 |
| 666 | udp-n-acteylglucosamine pyrophosphorylase 1-like 1 | -0.7 | -0.9 | -1.1 |
| 13876 | ubiquitin | -0.7 | -1.1 | -0.7 |
| 1467 | small nuclear ribonucleoprotein d1 | -0.7 | -1.0 | -1.0 |
| 7208 | methylcrotonoyl-CoA carboxylase alpha subunit | -0.7 | -1.4 | -1.0 |
| 2723 | kinesin family protein | -0.7 | -1.5 | -2.3 |
| 1657 | heat shock 70 kda protein 4 | -0.7 | -0.9 | -1.2 |
| 4445 | eukaryotic initiation factor 4 eIF4-like protein | -0.7 | -1.1 | -1.1 |
| 9633 | atp-binding cassette superfamily | -0.7 | 0.3 | 1.4 |
| 1631 | proliferation-associated protein | -0.8 | -1.5 | -1.5 |
| 12698 | oxidoreductase | -0.8 | -1.0 | -1.1 |
| 13895 | keratin associated protein 10-2 | -0.8 | -0.9 | -1.0 |
| 102 | eukaryotic initiation factor 4G middle domain protein | -0.8 | -1.0 | -1.2 |
| 1758 | DnaJ-like protein | -0.8 | -1.1 | -1.2 |
| 17333 | carotenoid cleavage dioxygenase 1 | -0.8 | 0.1 | 1.7 |
| 2933 | beta-N-acetylhexosaminidase | -0.8 | -1.3 | -1.3 |
| 15470 | YdcF-like family protein | -0.9 | -0.9 | -1.1 |
| 1356 | fructose-1,6-bisphosphate aldolase | -0.9 | -2.5 | -2.9 |
| 4969 | DUF3223-domain containing protein | -0.9 | -0.6 | -1.1 |
| 1048 | D-3-phosphoglycerate dehydrogenase | -0.9 | -1.0 | -1.2 |
| 1587 | circadian rna-binding protein chlamy 1 subunit c1 | -0.9 | -0.9 | -1.1 |
| 11813 | amino acid transporter | -0.9 | -1.4 | -1.2 |
| 5299 | 5-formyltetrahydrofolate cycloligase | -0.9 | -0.9 | -1.3 |
| 4603 | serine hydroxymethyltransferase | -1.0 | -1.0 | -0.8 |
| 8713 | retrotransposable element tf2 155 kda protein type 1-like | -1.0 | -0.2 | -0.1 |
| 9731 | rad51 recombination protein | -1.0 | 1.3 | 0.6 |
| 6790 | prostaglandin dehydrogenase | -1.0 | -0.4 | 0.4 |
| 2348 | polyubiquitin-like protein | -1.0 | -0.6 | -0.5 |
| 4865 | histidine kinase- dna gyrase b- and hsp90-like atpase family | -1.0 | -0.7 | -0.6 |
| 357 | elongation factor ef-3 | -1.0 | -0.6 | -0.6 |
| 15731 | 2-oxoisovalerate dehydrogenase subunit mitochondrial-like | -1.0 | 0.5 | 0.6 |
| 5658 | solanesyl diphosphate synthase | -1.1 | -0.4 | 0.3 |
| 10143 | retrotransposable element tf2 155 kda protein type 1-like | -1.1 | -0.2 | -0.4 |
| 11717 | protein-s-isoprenylcysteine o-methyltransferase | -1.1 | -1.3 | -1.2 |
| 16497 | generative cell specific-1 | -1.1 | -0.7 | -0.5 |
| 4967 | dwnn a cchc-type zinc finger | -1.1 | -0.4 | -0.6 |
| 2760 | d-lactate dehydrogenase | -1.1 | -0.4 | -0.1 |
| 7889 | btb poz domain-containing protein kctd16-like | -1.1 | 0.1 | 0.1 |
| 7424 | amino acid permease | -1.1 | -1.6 | -1.7 |
| 16651 | 3-oxoacyl-dehydrogenase | -1.1 | -0.5 | -0.1 |
| 12790 | GTP-dependent nucleic acid-binding protein | -1.2 | -1.8 | -1.6 |
| 16993 | ankyrin domain-containing protein | -1.2 | -0.4 | -0.5 |
| 1826 | putative pyridoxamine 5'-phosphate oxidase | -1.4 | -1.8 | -1.6 |
| 14411 | myb transcription factor 1 | -1.4 | -1.7 | -0.5 |
| 2154 | carotene biosynthesis-related protein | -1.4 | -0.2 | 0.8 |
| 1906 | high light inducible protein | -1.5 | -0.3 | 0.4 |
| 6541 | CWF19-like protein | -1.6 | -0.7 | -0.8 |
| 356 | 5-methyltetrahydropteroyltriglutamate--homocysteine s-methyltransferase | -1.6 | -1.3 | -1.4 |
| 3576 | low iron induced protein | -2.0 | -2.9 | -2.4 |
| 11154 | zinc-containing alcohol dehydrogenase | -2.3 | -0.7 | 0.8 |
| 6731 | ferritin-like | -3.6 | -1.2 | 0.2 |
