# Supplementary material for: Interaction of Temperature and Photoperiod Increases Growth and Oil Content in the Marine Microalgae Dunaliella viridis
Source: PLoS One. 2015 May 19;10(5):e0127562. doi: 10.1371/journal.pone.0127562 (PMC4437649; doi:10.1371/journal.pone.0127562)
Supplement: S6 Table — (DOCX) [file pone.0127562.s019.docx]

**S6 Table. Transcripts differentially expressed under light and temperature at 30, 40 or 54 hrs.** The list was sorted by Log_2_FC at 40 hours from the highest (up-regulated transcripts) to the lowest (down-regulated transcripts) for the light, all the transcripts presented in this list were differentially expressed under both light and temperature at any one of the three time points.

|  |  | **Log_2_FC(LL/LD)** | | | | **Log_2_FC(35°C/25°C)** | | |
| --- | --- | --- | --- | --- | --- | --- | --- | --- |
| **Transcript** | **Description** | **16** | **30** | **40** | **54** | **30** | **40** | **54** |
| 10594 | chloroplast carotene biosynthesis-related protein | 7.4 | -1.1 | 7.2 | -1.8 | -2.9 | -0.5 | 0.8 |
| 2256 | chloroplast carotene biosynthesis-related protein | 5.9 | -2.1 | 7.2 | -2.2 | -2.4 | -0.8 | 0.2 |
| 14127 | short-chain dehydrogenase_reductase SDR | 3.0 | -1.2 | 4.4 | -1.9 | 0.2 | 0.9 | 1.8 |
| 3576 | low iron induced protein | 3.9 | -0.5 | 4.2 | -1.1 | -2.0 | -2.9 | -2.4 |
| 11175 | uncharacterized protein | 1.7 | -0.3 | 3.8 | -1.2 | -0.3 | 0.4 | 1.3 |
| 6731 | ferritin-like | 3.1 | -0.6 | 3.7 | -0.6 | -3.6 | -1.2 | 0.2 |
| 848 | heat shock protein hsp90 family protein | 0.2 | 3.3 | 2.8 | 1.5 | 3.4 | 3.4 | 2.6 |
| 5549 | heat shock protein hsp20 | 0.2 | 1.8 | 2.4 | 0.7 | 2.7 | 2.1 | 1.8 |
| 134 | multicopper ferroxidase | 2.9 | -0.4 | 2.4 | -0.2 | -0.3 | -2.1 | -1.1 |
| 203 | glyceraldehyde-3-phosphate dehydrogenase | 3.2 | 3.5 | 2.3 | 2.2 | -0.6 | 0.5 | 1.4 |
| 5934 | DUF4419-domain containing protein | 1.1 | 1.5 | 2.2 | 1.8 | -1.7 | -2.7 | -0.9 |
| 2188 | manganese superoxide dismutase | 1.5 | 0.2 | 2.2 | 0.6 | 0.6 | -1.2 | 0.3 |
| 7050 | large subunit ribosomal RNA (rrnL5), mitochondrial | 2.4 | 0.3 | 2.2 | 0.2 | 0.4 | 1.6 | 3.8 |
| 1826 | putative pyridoxamine 5'-phosphate oxidase | 1.3 | 0.0 | 2.2 | -0.8 | -1.4 | -1.8 | -1.6 |
| 7108 | glutathione peroxidase | 2.0 | -0.3 | 2.0 | -1.2 | 0.0 | 0.1 | 1.1 |
| 5148 | flavin-containing amine oxidasedehydrogenase | 1.7 | -1.0 | 2.0 | -1.3 | -0.3 | 0.8 | 1.2 |
| 5709 | spore coat protein | 0.9 | 0.2 | 1.9 | -0.2 | 1.1 | 1.1 | 1.6 |
| 5410 | photosystem I light harvesting chlorophyll a/b BP 3 | 1.2 | 0.9 | 1.8 | 0.3 | -0.5 | -3.1 | -2.7 |
| 1717 | low-co2 inducible protein | -1.7 | 1.2 | 1.8 | -0.1 | 1.7 | 1.6 | 1.5 |
| 6582 | pre-rRNA-processing protein | 1.0 | 0.4 | 1.8 | -0.5 | -0.5 | -1.2 | -0.6 |
| 10226 | hypothetical protein | 1.7 | -0.3 | 1.7 | -1.1 | -0.2 | 0.8 | 1.0 |
| 12270 | HMG-box containing protein | 0.2 | 0.8 | 1.6 | 3.2 | 0.1 | 1.7 | 1.5 |
| 4559 | heat shock protein hsp20 | -0.1 | 1.9 | 1.6 | 0.9 | 2.4 | 2.1 | 1.1 |
| 13238 | hypothetical protein COCSUDRAFT_33357 | 0.0 | 0.4 | 1.6 | -0.4 | -1.4 | -1.2 | -0.8 |
| 1758 | DnaJ-like protein | 1.1 | 0.3 | 1.5 | -0.9 | -0.8 | -1.1 | -1.2 |
| 15897 | rta1 domain protein | 0.2 | 1.1 | 1.4 | 1.5 | -0.2 | 3.2 | 4.0 |
| 1858 | iron-sulfur cluster scaffold-like protein | 1.1 | 0.6 | 1.4 | -0.3 | 0.5 | 1.2 | 1.9 |
| 3076 | DUF2256 domain protein | 1.0 | -0.2 | 1.4 | -0.8 | 0.6 | 1.1 | 1.7 |
| 9115 | helicase superfamily protein | 1.3 | 1.0 | 1.3 | -0.1 | -0.5 | -1.1 | 0.1 |
| 5348 | phosphofructokinase family protein | 1.4 | 0.3 | 1.3 | -1.0 | 0.3 | 1.0 | 0.7 |
| 13776 | ABCG transporter family | 0.0 | 1.6 | 1.2 | 2.8 | -0.8 | 0.6 | 1.6 |
| 3460 | hypothetical protein VOLCADRAFT_91598 | 0.7 | 0.4 | 1.2 | 0.0 | 1.3 | 1.4 | 1.7 |
| 568 | flavodoxin | 2.0 | 0.3 | 1.2 | -0.4 | -0.4 | -2.5 | -2.2 |
| 4762 | 12-oxophytodienoic acid reductase | 1.5 | 0.0 | 1.2 | -0.8 | -0.1 | 1.0 | 1.5 |
| 7962 | phosphofructokinase family protein | 1.2 | 0.1 | 1.2 | -0.9 | 0.7 | 1.0 | 0.8 |
| 3087 | light-harvesting chlorophyll-a/b protein of photosystem I, type III | 1.2 | -1.2 | 1.2 | -2.6 | 1.6 | -1.5 | -1.6 |
| 12066 | SCP-domain containing protein | 0.4 | 1.6 | 1.1 | 1.3 | 0.8 | 2.0 | 1.8 |
| 5840 | o-methyltransferase | 0.8 | 0.5 | 1.1 | 0.4 | 0.0 | 1.5 | 2.6 |
| 5629 | protein | -0.6 | 0.6 | 1.1 | 0.0 | 0.6 | 1.3 | 1.3 |
| 8814 | hypothetical prot.VOLCADRAFT_118047 | 0.5 | 0.3 | 1.1 | -0.4 | -1.0 | -1.2 | -0.2 |
| 17242 | predicted protein [Chlamydomonas] | -1.5 | 1.0 | 1.1 | -0.7 | -1.9 | -2.3 | -1.2 |
| 1421 | RNA-binding domain-containing protein | 0.4 | 0.3 | 1.1 | -0.8 | -0.7 | -1.2 | -1.0 |
| 13887 | methyltransferase | 0.4 | -0.2 | 1.1 | -0.9 | -0.9 | -1.4 | -0.9 |
| 5468 | heat shock protein hsp20 | -0.6 | 1.8 | 1.0 | 0.8 | 2.5 | 1.9 | 1.6 |
| 4596 | cysteine protease | 0.5 | -0.1 | 1.0 | -0.3 | 0.9 | 1.1 | 1.9 |
| 2827 | hypoth.protein COCSUDRAFT_62069 | 1.0 | -0.1 | 1.0 | -0.7 | -0.8 | -1.3 | -0.9 |
| 11891 | predicted protein [Chlamydomonas] | 1.8 | -1.0 | 1.0 | -1.6 | 0.5 | 1.7 | 1.9 |
| 1619 | hypothetical protein CHLNCDRAFT_49703 | 1.3 | -0.5 | 0.9 | -1.2 | 0.0 | 0.5 | 1.0 |
| 2099 | ribonucleoside-diphosphate reductase SU | 0.8 | 2.3 | 0.8 | 2.3 | 0.5 | 0.9 | 1.4 |
| 6120 | flagellar flavodoxin | 0.0 | 1.4 | 0.8 | 1.9 | -1.2 | -0.6 | 0.3 |
| 14739 | hypothetical protein HCH_07015 | 0.2 | 1.1 | 0.7 | 0.1 | -1.1 | -1.9 | -0.7 |
| 304 | fructose-bisphosphate aldolase | -0.3 | 1.2 | 0.6 | -0.9 | 3.0 | 2.5 | 2.8 |
| 4750 | rieske (2fe-2s) domain-containing protein | 0.7 | -0.6 | 0.6 | -1.2 | 0.3 | 0.7 | 1.1 |
| 2057 | C-terminal processing peptidase family S41 | 0.7 | -0.4 | 0.6 | -1.3 | 1.1 | 1.7 | 2.1 |
| 2093 | heat shock protein | -0.9 | 1.2 | 0.5 | 0.0 | 1.3 | 1.2 | 0.7 |
| 8503 | small 2 heat shock protein | 0.0 | 1.0 | 0.5 | -1.0 | 1.0 | 1.0 | 1.3 |
| 1276 | protein | 0.2 | -0.3 | 0.5 | -1.0 | 0.8 | 0.8 | 1.1 |
| 3039 | chloroplast photosystem ii-associated 22 kda | 1.6 | -0.3 | 0.5 | -1.3 | 0.6 | 2.2 | 2.4 |
| 4513 | chloroplast DnaJ-like protein | 0.3 | 0.7 | 0.4 | 1.4 | 0.6 | 1.7 | 2.7 |
| 4182 | ribokinase | -0.1 | 1.1 | 0.4 | -0.6 | 1.3 | 1.2 | 1.1 |
| 2296 | glycosyl hydrolases family 32 protein | 0.2 | -0.3 | 0.4 | -1.3 | 2.2 | 2.2 | 2.9 |
| 2354 | protein | 0.1 | 1.4 | 0.3 | 1.1 | 0.2 | 0.6 | 1.1 |
| 5461 | major lipid droplet protein MLDP | 0.7 | -0.1 | 0.3 | -1.1 | 0.4 | 0.9 | 1.1 |
| 4416 | protein | 0.2 | -0.3 | 0.3 | -1.1 | 0.9 | 0.8 | 1.1 |
| 16851 | hypothetical protein [Dunaliella viridis] | 0.3 | -0.3 | 0.3 | -1.2 | -1.4 | -0.7 | -1.2 |
| 5707 | put. retinoblastoma-binding 9 serine hydrolase | 0.0 | -0.3 | 0.2 | -1.0 | 1.8 | 1.0 | 1.6 |
| 802 | col1 | -0.1 | -0.2 | 0.2 | -1.1 | 0.0 | 0.9 | 1.3 |
| 2069 | DnaJ-Hsp40 domain protein | 0.5 | -0.6 | 0.2 | -1.4 | 1.1 | 0.9 | 1.3 |
| 11396 | hypothetical protein VOLCADRAFT_106870 | -0.1 | -1.3 | 0.2 | -1.6 | 1.6 | 2.9 | 3.5 |
| 18493 | histone h2b | -0.3 | 1.5 | 0.1 | 3.8 | -0.6 | -0.8 | -1.9 |
| 1850 | cytochrome b | 0.7 | -2.4 | 0.1 | 0.5 | 1.2 | 2.5 | 3.1 |
| 918 | transitional endoplasmic reticulum atpase CDC48 | 0.1 | 1.0 | 0.1 | 0.1 | -1.1 | -0.9 | -0.3 |
| 2692 | phosphoglycerate mutase | 0.4 | -0.2 | 0.1 | -1.3 | 0.5 | 1.4 | 1.4 |
| 8897 | hypothetical protein | 0.4 | -1.0 | 0.1 | -1.8 | 1.6 | 2.1 | 2.0 |
| 2132 | RNA recognition motif superfamily protein | 0.4 | -2.2 | 0.1 | -3.3 | 2.2 | 1.2 | 1.2 |
| 2847 | cytochrome oxidase subunit 1 | 0.5 | -2.6 | -0.1 | 1.0 | 0.5 | 1.7 | 2.0 |
| 16602 | kinase-like protein | -0.4 | -0.4 | -0.1 | -1.3 | 0.6 | 0.4 | 1.1 |
| 1434 | uncharacterized protein loc100280346 | 0.1 | -0.7 | -0.1 | -1.4 | 0.7 | 1.1 | 1.0 |
| 9731 | rad51 recombination protein | -0.1 | 3.7 | -0.2 | 3.9 | -1.0 | 1.3 | 0.6 |
| 9458 | hypothetical protein | 1.5 | -2.9 | -0.2 | 1.1 | 0.8 | 2.4 | 2.8 |
| 14066 | GAF domain-containing protein | -0.7 | -0.5 | -0.3 | -1.2 | 1.1 | 0.8 | 1.0 |
| 3861 | glyoxalase bleomycin resistance protein dioxygenase superfamily protein | 0.2 | -1.2 | -0.3 | -1.3 | 1.0 | 1.0 | 1.2 |
| 8581 | thioredoxin-like protein | -0.2 | 1.3 | -0.4 | 1.4 | -0.2 | 1.5 | 1.9 |
| 2018 | utp--glucose-1-phosphate uridylyltransferase | -1.0 | 1.4 | -0.4 | -0.2 | 1.7 | 1.6 | 1.3 |
| 373 | photosystem i reaction center subunit xi | -0.9 | 1.1 | -0.4 | -0.4 | 1.0 | 0.4 | 0.5 |
| 6413 | guanylate cyclase | -0.4 | -0.8 | -0.4 | -1.5 | 0.7 | 0.7 | 1.1 |
| 1804 | photosystem i reaction center SU chloroplast prec. | -0.9 | 1.1 | -0.5 | -0.7 | 1.1 | 0.4 | 0.2 |
| 2222 | hypothetical protein CHLREDRAFT_195861 | -0.8 | -0.3 | -0.5 | -1.1 | 2.0 | 2.2 | 1.9 |
| 10517 | OTU-like cysteine protease | -0.4 | -1.3 | -0.5 | -1.9 | 1.4 | 1.6 | 1.9 |
| 12254 | chromosome segregation protein smc | -0.1 | 3.2 | -0.6 | 3.4 | -1.4 | 0.3 | -0.6 |
| 16438 | hypothetical zinc finger protein | -0.4 | 2.5 | -0.6 | 2.9 | -1.6 | 0.6 | -0.4 |
| 12032 | hypothetical protein | -0.8 | -0.6 | -0.6 | -1.3 | 1.1 | 1.1 | 1.1 |
| 7731 | chromodomain-containing protein | -0.1 | 2.8 | -0.8 | 2.8 | -1.1 | 0.1 | -0.4 |
| 9369 | hypothetical protein CHLREDRAFT_144552 | -0.7 | 2.7 | -0.8 | 2.7 | -1.1 | 0.2 | -0.2 |
| 5815 | hypothetical protein VOLCADRAFT_102815 [ | -0.7 | 3.7 | -0.9 | 3.4 | -1.3 | 0.2 | -0.4 |
| 9904 | SCP-like domain protein | -0.1 | 1.9 | -0.9 | 2.1 | 1.2 | 3.7 | 4.3 |
| 4020 | hypothetical protein | -0.3 | -0.6 | -0.9 | -1.5 | 0.7 | 1.0 | 2.0 |
| 25 | 18S, ITS, 26S rRNA | -0.2 | -4.8 | -1.0 | 3.7 | -0.9 | -0.5 | -1.4 |
| 8766 | type xi myosin heavy chain | -0.7 | 3.2 | -1.0 | 3.5 | -0.5 | 1.0 | 0.2 |
| 11631 | predicted protein [Chlamydomonas reinhardtii] | -0.2 | 1.0 | -1.0 | 1.4 | 0.4 | 1.0 | 0.6 |
| 2059 | predicted protein [Chlamydomonas reinhardtii] | -0.6 | 0.9 | -1.0 | 0.2 | 0.4 | 1.1 | 0.7 |
| 3183 | lipase LIPG2 | -0.6 | 0.3 | -1.0 | 0.2 | 1.5 | 1.7 | 1.5 |
| 3101 | zf-dnl-domain-containing protein | -1.0 | 0.4 | -1.0 | 0.1 | 2.7 | 3.2 | 3.0 |
| 5025 | flagellar associated protein | -0.6 | 0.8 | -1.0 | 0.0 | 0.4 | 1.2 | 0.8 |
| 3157 | DNA topoisomerase II | -0.4 | 4.1 | -1.1 | 4.7 | -0.7 | 1.1 | 0.2 |
| 4259 | RING-finger-containing protein | -0.8 | 2.6 | -1.1 | 2.2 | -0.5 | 1.6 | 0.2 |
| 16018 | hypothetical protein | -1.1 | 2.4 | -1.1 | 2.2 | -0.8 | 1.0 | -0.3 |
| 18384 | condensin complex subunit 2-like | -0.5 | 2.8 | -1.1 | 2.1 | -1.0 | 1.2 | 0.3 |
| 5161 | [Chlamydomonas reinhardtii] | -0.8 | 2.1 | -1.1 | 2.0 | -0.3 | 1.0 | 0.3 |
| 14476 | antagonist of mitotic exit network protein 1-like | -1.1 | 1.1 | -1.1 | 1.0 | 0.5 | 1.3 | 0.1 |
| 5076 | predicted protein [Nematostella] | -0.4 | 1.2 | -1.1 | 0.6 | 0.5 | 1.9 | 3.5 |
| 13794 | predicted protein [Chlamydomonas reinhardtii] | -0.7 | 1.2 | -1.1 | -0.1 | -1.0 | 0.0 | -0.5 |
| 12176 | hypothetical protein VOLCADRAFT_96759 | -0.6 | 0.3 | -1.1 | -0.2 | 0.4 | 1.3 | 0.8 |
| 7488 | hypothetical protein | -0.9 | 0.1 | -1.1 | -0.9 | 1.4 | 1.3 | 0.9 |
| 10698 | magnesium-chelatase subunit i | -0.5 | 3.5 | -1.2 | 3.5 | 0.9 | 2.0 | 0.4 |
| 9041 | kinesin-like protein | -0.7 | 3.8 | -1.2 | 3.4 | -1.0 | 0.7 | -0.3 |
| 14216 | predicted protein [Chlamydomonas] | -1.1 | 2.5 | -1.2 | 2.5 | -0.2 | 1.3 | 0.1 |
| 13519 | hypothetical protein | -1.2 | 2.1 | -1.2 | 1.5 | -0.5 | 1.0 | 0.3 |
| 4902 | zinc-metallopeptidase-like protein | -0.2 | 0.6 | -1.2 | 0.5 | 0.2 | 1.0 | 0.5 |
| 8753 | predicted protein [Chlamydomonas] | -0.9 | 0.2 | -1.2 | -0.2 | 1.0 | 1.5 | 1.2 |
| 8618 | chloroplast division site-detMinE | -0.7 | 5.3 | -1.3 | 5.0 | -1.1 | 0.6 | -0.3 |
| 3476 | gag-pol polyprotein | -0.5 | 0.3 | -1.3 | 2.2 | 0.3 | 1.2 | -0.8 |
| 4427 | SCP-like domain protein | -1.1 | 1.5 | -1.3 | 1.8 | 1.6 | 4.1 | 4.2 |
| 6336 | flagellar autonomy 2 NIMA family kinase | -1.1 | 1.8 | -1.3 | 1.2 | -0.2 | 1.2 | 0.5 |
| 1868 | dihydroxyacetone reductase | -1.7 | 1.2 | -1.3 | 0.9 | -0.4 | -1.4 | -1.4 |
| 15245 | sodium phosphate symporter | -1.3 | 0.4 | -1.3 | 0.3 | 0.6 | 3.8 | 4.2 |
| 5222 | conserved protein chloroplast | -1.5 | 0.7 | -1.3 | -0.5 | -0.1 | -1.1 | -1.2 |
| 7135 | aldo-keto reductase | -1.0 | -0.7 | -1.3 | -1.9 | 2.1 | 1.3 | 1.1 |
| 11939 | dynamin-like | -0.8 | 3.1 | -1.4 | 2.3 | -0.4 | 1.0 | -0.1 |
| 16365 | kinesin-like | -1.2 | 1.6 | -1.4 | 0.9 | 0.3 | 1.6 | 0.5 |
| 6729 | homogentisate 1,2-dioxygenase | -1.3 | 0.8 | -1.4 | 0.9 | 0.5 | 1.2 | 1.1 |
| 7995 | lipase-like | -1.1 | 1.2 | -1.4 | 0.6 | 1.2 | 1.6 | 1.3 |
| 14765 | hypothetical protein VOLCADRAFT_46480 | -1.7 | -0.2 | -1.4 | 0.1 | 1.3 | 1.6 | 1.0 |
| 4714 | COCSUDRAFT_66290 | -0.7 | 0.2 | -1.4 | -0.2 | 0.9 | 1.6 | 1.7 |
| 12323 | hypothetical protein | -0.7 | 3.4 | -1.5 | 3.9 | -0.4 | 1.2 | 0.0 |
| 4165 | pred. protein [Chlamydomonas] | -1.5 | 2.3 | -1.5 | 2.3 | -0.1 | 1.3 | 0.1 |
| 17224 | hypothetical protCHLREDRAFT_144209 | -1.1 | 2.0 | -1.5 | 1.8 | 0.1 | 1.4 | -0.2 |
| 10810 | fad fmn-containing dehydrogenase | -1.4 | 1.3 | -1.5 | 0.9 | 1.0 | 1.8 | 1.3 |
| 14011 | hypothetical protein VOLCADRAFT_91165 | -1.0 | 0.1 | -1.5 | 0.2 | 1.0 | 1.8 | 2.0 |
| 7345 | sodium phosphate symporter | -0.5 | 0.0 | -1.5 | -1.3 | 1.0 | 3.3 | 3.3 |
| 8048 | hypothetical protCOCSUDRAFT_65399 | -1.1 | 3.8 | -1.6 | 3.5 | 0.6 | 1.9 | 0.6 |
| 15076 | condensin complex subunit 2-like | -0.8 | 3.0 | -1.6 | 3.2 | -0.7 | 1.6 | 0.6 |
| 6149 | p700 apoprotein a1 of photosystem I, psaA | -0.6 | -5.8 | -1.6 | 3.1 | -1.3 | 0.1 | -0.8 |
| 5620 | predicted protein [Chlamydomonas] | -1.2 | 2.2 | -1.6 | 2.2 | 0.1 | 1.2 | 0.3 |
| 1013 | rad54-like protein | -1.2 | 1.2 | -1.6 | 1.2 | 0.2 | 1.6 | 1.1 |
| 9503 | tetratricopeptide repeat-containing protein | -1.2 | 0.8 | -1.6 | 0.2 | 1.1 | 1.7 | 1.5 |
| 3508 | B-type cyclin 2 | -1.0 | 6.0 | -1.7 | 6.0 | -0.7 | 1.1 | -0.4 |
| 14883 | predicted protein [Chlamydomonas] | -1.2 | 2.8 | -1.7 | 2.8 | -0.9 | 1.1 | -0.2 |
| 15807 | protein kinase-like | -1.0 | 1.0 | -1.7 | 0.9 | 1.1 | 1.8 | 0.7 |
| 8896 | leucine-rich repeat protein | -1.6 | 1.9 | -1.8 | 1.5 | 0.1 | 1.1 | -0.1 |
| 11294 | sodium phosphate symporter | -0.4 | -0.1 | -1.8 | -1.1 | 0.8 | 3.2 | 3.3 |
| 7553 | flagellar associated protein, STOP domain- | -1.3 | 2.4 | -1.9 | 1.8 | -0.5 | 0.3 | -1.1 |
| 2857 | cytochrome c oxidase subunit 2 | -2.4 | 1.2 | -2.4 | 0.9 | 2.7 | 4.5 | 4.7 |
| 11035 | cf1 alpha subunit of atp synthase | -0.4 | -6.0 | -2.5 | 2.7 | -2.9 | -0.1 | -1.5 |
| 13991 | cytosolic phosphoglucose isomerase | -2.3 | 1.2 | -2.7 | 1.0 | 0.1 | 0.0 | -1.1 |
| 5920 | phosphoenolpyruvate carboxykinase | -3.5 | 0.7 | -3.1 | 0.8 | 0.9 | 1.6 | 0.6 |
