# Supplementary material for: Interaction of Temperature and Photoperiod Increases Growth and Oil Content in the Marine Microalgae Dunaliella viridis
Source: PLoS One. 2015 May 19;10(5):e0127562. doi: 10.1371/journal.pone.0127562 (PMC4437649; doi:10.1371/journal.pone.0127562)
Supplement: S7 Table — (DOCX) [file pone.0127562.s020.docx]

**S7 Table. Transcripts expression data used in pathways from Figs. 9-11.**

**A. Effect of temperature on the expression of transcripts from fatty acid, lipid and starch metabolism at 30, 40 and 54 hrs.**

|  | |  | **Log_2_FC (35°C/25°C)** | | | |  | | **RPKM  (35°C)** | | | | **RPKM (25°C)** | | | | **RPKM (25°C)** | |
| --- | --- | --- | --- | --- | --- | --- | --- | --- | --- | --- | --- | --- | --- | --- | --- | --- | --- | --- |
| **Transcript** | | **Description** | | **30** | **40** | **54** |  | | **30** | | **40** | **54** |  | | **30** | **40** | **54** | |
| 1160 | | alpha-carboxyltransferase subunit | | 0.0 | -0.2 | -0.2 |  | | 212 | | 207 | 253 |  | | 214 | 242 | 291 | |
| 3474 | | beta-carboxyltransferase subunit | | -0.1 | -0.2 | -0.1 |  | | 129 | | 128 | 167 |  | | 136 | 149 | 178 | |
| 41 | | biotin carboxylase subunit | | 0.0 | -0.5 | -0.5 |  | | 224 | | 161 | 181 |  | | 221 | 229 | 256 | |
| 1407 | | biotin carboxyl carrier protein subunit | | -0.1 | -0.5 | -0.5 |  | | 149 | | 109 | 140 |  | | 162 | 159 | 192 | |
| 874 | | malonyl-CoA ACP transacylase | | -0.1 | -0.5 | -0.4 |  | | 190 | | 157 | 153 |  | | 209 | 217 | 203 | |
| 3765 | | ketoacyl-ACP synthase I | | -0.5 | -0.7 | -0.7 |  | | 253 | | 222 | 227 |  | | 308 | 309 | 320 | |
| 4759 | | ketoacyl-ACP synthase II | | -0.3 | -0.5 | -0.6 |  | | 45 | | 35 | 33 |  | | 56 | 51 | 52 | |
| 3487 | | ketoacyl-ACP synthase III | | -0.3 | -0.5 | -0.5 |  | | 120 | | 105 | 102 |  | | 173 | 170 | 164 | |
| 200 | | ketoacyl-ACP reductase | | 0.3 | -0.1 | -0.1 |  | | 237 | | 182 | 179 |  | | 191 | 201 | 196 | |
| 172 | | hydroxyacyl-ACP dehydrase | | -0.1 | -0.3 | -0.4 |  | | 122 | | 95 | 95 |  | | 131 | 114 | 126 | |
| 3109 | | enoyl-ACP reductase | | -0.2 | -0.6 | -0.6 |  | | 240 | | 174 | 152 |  | | 272 | 259 | 237 | |
| 1900 | | stearoyl-ACP-D9-desaturase 2-1 | | 0.0 | 0.0 | 0.0 |  | | 177 | | 178 | 176 |  | | 178 | 178 | 171 | |
| 68 | | stearoyl-ACP-D9-desaturase 2-2 | | 0.0 | 0.4 | 0.6 |  | | 27 | | 38 | 37 |  | | 27 | 28 | 25 | |
| 4670 | | acyl-ACP thioesterase | | 0.1 | 0.4 | 0.7 |  | | 33 | | 41 | 48 |  | | 30 | 31 | 29 | |
| 1955 | | glycerol-3-P acyltransferase | | -0.4 | -0.2 | -0.4 |  | | 66 | | 75 | 72 |  | | 86 | 86 | 93 | |
| 6126 | | lysophosphatidic acid acyltransferase | | -0.2 | -0.6 | -0.8 |  | | 68 | | 47 | 47 |  | | 78 | 69 | 83 | |
| 9078 | | phosphatidate phosphatase | | -0.1 | 0.4 | 0.9 |  | | 7 | | 11 | 14 |  | | 8 | 8 | 7 | |
| 6208 | | diacylglycerol acyltransferase 1 | | -0.2 | 0.2 | 0.4 |  | | 10 | | 14 | 16 |  | | 12 | 12 | 12 | |
| 3780 | | diacylglycerol acyltransferase 1 | | 0.1 | -0.4 | -0.5 |  | | 57 | | 39 | 37 |  | | 55 | 51 | 52 | |
| 2006 | | diacylglycerol acyltransferase 2 | | 0.1 | 0.0 | 0.1 |  | | 88 | | 85 | 78 |  | | 81 | 85 | 75 | |
| 7672 | | diacylglycerol acyltransferase 4 | | -0.2 | -0.3 | -0.2 |  | | 45 | | 41 | 40 |  | | 52 | 50 | 47 | |
| 6053 | | diacylglycerol acyl transferase DGTT | | 0.2 | -0.1 | -0.3 |  | | 54 | | 48 | 41 |  | | 47 | 51 | 49 | |
| 9514 | | diacylglycerol acyl transferase DGTT | | -0.1 | 0.0 | 0.5 |  | | 9 | | 9 | 13 |  | | 10 | 9 | 9 | |
| 11338 | | diacylglycerol acyl transferase DGTT | | -0.1 | -0.1 | 0.0 |  | | 11 | | 10 | 12 |  | | 11 | 11 | 11 | |
| 3641 | | CDP-diacylglycerol synthase | | 0.1 | 0.2 | 0.2 |  | | 54 | | 60 | 60 |  | | 50 | 51 | 51 | |
| 2765 | | PG-phosphate synthase | | 0.3 | 0.6 | 0.2 |  | | 60 | | 69 | 57 |  | | 49 | 46 | 50 | |
| 3668 | | UDP-sulfoquinovose synthase 1 | | 0.0 | -0.7 | -0.6 |  | | 83 | | 55 | 58 |  | | 83 | 87 | 86 | |
| 953 | | sulfolipid synthase | | 0.7 | 0.5 | 0.5 |  | | 24 | | 19 | 21 |  | | 14 | 14 | 15 | |
| 5621 | | MGDG synthase 1 | | -0.1 | -0.4 | -0.7 |  | | 26 | | 22 | 18 |  | | 28 | 30 | 29 | |
| 1408 | | DGDG synthase 1 | | 0.2 | 0.6 | 0.5 |  | | 28 | | 34 | 40 |  | | 24 | 22 | 28 | |
| 10725 | | plastid galactoglycerolipid degrad1 | | 0.2 | 0.3 | 0.4 |  | | 7 | | 10 | 12 |  | | 6 | 8 | 9 | |
| 306 | | GGGT | | 0.6 | 0.6 | 1.0 |  | | 43 | | 51 | 58 |  | | 29 | 34 | 28 | |
| 6787 | | phospholipid:DGAT | | -0.2 | -0.1 | -0.3 |  | | 25 | | 24 | 22 |  | | 28 | 25 | 27 | |
| 5461 | | major lipid droplet protein | | 0.4 | 0.9 | 1.1 |  | | 127 | | 166 | 182 |  | | 96 | 88 | 87 | |
| 3395 | | β-ketoacyl-CoA synthase | | -0.1 | -0.4 | -0.5 |  | | 138 | | 114 | 122 |  | | 153 | 146 | 170 | |
| 12579 | | 3-hydroxyacyl-CoA dehydrogenase | | 1.9 | 1.3 | 3.0 |  | | 5 | | 5 | 7 |  | | 1 | 2 | 1 | |
| 5523 | | Trans-2-enoyl-CoA reductase | | 0.2 | 0.2 | 0.3 |  | | 42 | | 45 | 44 |  | | 36 | 39 | 36 | |
| 3762 | | Palmitoyl-CoA hydrolase | | 0.7 | 0.7 | 0.8 |  | | 51 | | 58 | 60 |  | | 32 | 35 | 36 | |
| 2217 | | MGDG palmitate Δ-7 desaturase | | 0.3 | -0.1 | 0.0 |  | | 209 | | 162 | 149 |  | | 164 | 172 | 154 | |
| 15 | | ω-6 fatty acid desaturase 1 | | -0.2 | -0.5 | -0.4 |  | | 319 | | 277 | 258 |  | | 359 | 388 | 336 | |
| 3113 | | ω-6 fatty acid desaturase 2 | | 0.0 | -0.3 | -0.4 |  | | 29 | | 24 | 21 |  | | 30 | 29 | 27 | |
| 4592 | | ω-3 fatty acid desaturase | | -0.3 | -0.3 | -0.5 |  | | 289 | | 340 | 247 |  | | 366 | 415 | 339 | |
| 4740 | | class 3 lipase | | 0.0 | 0.0 | 0.2 |  | | 5 | | 6 | 6 |  | | 5 | 6 | 7 | |
| 6253 | | class 3 lipase | | 0.3 | 0.5 | 0.8 |  | | 49 | | 75 | 90 |  | | 40 | 54 | 50 | |
| 1438 | | lipase | | 0.7 | 0.1 | -0.2 |  | | 10 | | 11 | 9 |  | | 6 | 10 | 10 | |
| 3183 | | lipase | | 1.5 | 1.8 | 1.6 |  | | 36 | | 59 | 63 |  | | 13 | 17 | 21 | |
| 3414 | | triacylglycerol lipase | | 0.2 | 0.1 | -0.4 |  | | 35 | | 31 | 27 |  | | 31 | 29 | 36 | |
| 6345 | | Hexokinase | | 0.0 | 0.0 | -0.2 |  | | 12 | | 11 | 10 |  | | 13 | 11 | 11 | |
| 2573 | | Phosphoglucomutase | | 0.3 | 0.1 | -0.2 |  | | 258 | | 199 | 148 |  | | 208 | 181 | 165 | |
| 544 | | ADP-glucose phosphorylase small SU | | 0.0 | -0.3 | -0.1 |  | | 257 | | 229 | 253 |  | | 264 | 284 | 265 | |
| 1298 | | ADP-glucose phosphorylase large SU | | 0.2 | -0.2 | -0.2 |  | | 373 | | 297 | 299 |  | | 317 | 350 | 350 | |
| 6977 | | Granule bound starch synthase I | | -0.5 | 0.1 | -0.1 |  | | 89 | | 118 | 145 |  | | 122 | 112 | 155 | |
| 2601 | | Soluble starch synthase | | -0.2 | -0.2 | -0.3 |  | | 96 | | 78 | 74 |  | | 112 | 90 | 93 | |
| 1061 | | 1,4-alpha-glucan branching enzyme | | -0.3 | -0.5 | -0.7 |  | | 182 | | 143 | 141 |  | | 218 | 206 | 226 | |
| 5251 | | Isoamylase | | 0.2 | 0.0 | -0.3 |  | | 36 | | 37 | 36 |  | | 31 | 38 | 45 | |
| 1861 | | Pullulanase | | 0.0 | -0.4 | -0.6 |  | | 100 | | 73 | 61 |  | | 101 | 94 | 92 | |
| 3284 | | alpha-glucan water dikinase | | -0.2 | 0.1 | 0.1 |  | | 73 | | 83 | 72 |  | | 83 | 80 | 68 | |
| 2194 | | alpha-amylase | | -0.2 | 0.6 | 0.5 |  | | 65 | | 95 | 86 |  | | 72 | 64 | 62 | |
| 2268 | | oligo-1,6-glucosidase | | -0.1 | 0.5 | 0.4 |  | | 142 | | 174 | 169 |  | | 154 | 124 | 124 | |
| 9691 | | beta-amylase | | 0.1 | -0.2 | 0.3 |  | | 19 | | 19 | 25 |  | | 18 | 22 | 20 | |
| 7891 | | Disproportionating enzyme 1 (plastid) | | -0.1 | 0.5 | 0.3 |  | | 102 | | 116 | 98 |  | | 111 | 80 | 78 | |
| 13144 | | Disproportionating enzyme 2 (cytosol) | | -0.1 | 0.2 | 0.2 |  | | 45 | | 55 | 49 |  | | 46 | 48 | 44 | |
| 4697 | | Maltose transporter 1 | | 0.8 | 0.4 | 0.5 |  | | 23 | | 21 | 22 |  | | 13 | 16 | 15 | |

**B. Effect of light on the expression of transcripts from fatty acid, lipid and starch metabolism at 6, 16, 30, 40 and 54 hrs.**

|  |  | **Log_2_FC (LL/LD)** | | | | | **RPKM(LL)** | | | | | **RPKM(LD)** | | | | |
| --- | --- | --- | --- | --- | --- | --- | --- | --- | --- | --- | --- | --- | --- | --- | --- | --- |
| **Transcript** | **Description** | **6** | **16** | **30** | **40** | **54** | **6** | **16** | **30** | **40** | **54** | **6** | **16** | **30** | **40** | **54** |
| 1160 | alpha-carboxyltransferase subunit | 0.2 | -0.7 | 0.8 | -0.3 | 0 | 178 | 150 | 216 | 228 | 239 | 157 | 249 | 127 | 274 | 236 |
| 3474 | beta-carboxyltransferase subunit | 0 | -0.6 | 1.1 | -0.3 | 0.1 | 90 | 117 | 139 | 141 | 132 | 91 | 173 | 66 | 170 | 122 |
| 41 | biotin carboxylase subunit | 0.1 | -0.8 | 0.9 | -0.5 | 0.2 | 154 | 164 | 220 | 218 | 209 | 147 | 286 | 121 | 298 | 178 |
| 1407 | biotin carboxyl carrier protein subunit | -0.1 | -0.3 | 0.8 | 0 | -0.1 | 102 | 118 | 156 | 153 | 135 | 111 | 145 | 87 | 157 | 143 |
| 874 | malonyl-CoA ACP transacylase | 0 | 0.1 | 0.6 | 0.3 | -0.5 | 197 | 211 | 217 | 191 | 142 | 203 | 196 | 144 | 153 | 202 |
| 3765 | ketoacyl-ACP synthase I | 0 | -0.3 | 1 | 0.1 | -0.1 | 196 | 240 | 306 | 280 | 225 | 201 | 291 | 156 | 267 | 239 |
| 4759 | ketoacyl-ACP synthase II | -0.3 | 0.1 | 0.5 | 0 | -0.5 | 50 | 54 | 56 | 48 | 36 | 60 | 52 | 40 | 46 | 51 |
| 3487 | ketoacyl-ACP synthase III | -0.1 | 0 | 0.7 | 0.1 | -0.4 | 124 | 152 | 160 | 151 | 116 | 134 | 148 | 98 | 138 | 149 |
| 200 | ketoacyl-ACP reductase | 0.1 | -0.3 | 1 | 0.2 | -0.1 | 120 | 131 | 193 | 192 | 147 | 112 | 165 | 99 | 172 | 155 |
| 172 | hydroxyacyl-ACP dehydrase | -0.3 | 0.4 | 0.4 | 0.5 | -0.6 | 110 | 128 | 122 | 104 | 76 | 135 | 98 | 91 | 75 | 115 |
| 3109 | enoyl-ACP reductase | -0.1 | 0 | 0.3 | 0.1 | -0.7 | 267 | 256 | 264 | 242 | 183 | 283 | 264 | 216 | 231 | 303 |
| 1900 | stearoyl-ACP-D9-desaturase 2-1 | -0.1 | 0.6 | 0.4 | 0.4 | -0.5 | 215 | 218 | 184 | 176 | 128 | 231 | 147 | 139 | 135 | 186 |
| 68 | stearoyl-ACP-D9-desaturase 2-2 | -0.1 | 0.6 | 0.1 | 0.8 | -0.7 | 37 | 35 | 27 | 30 | 20 | 40 | 23 | 25 | 16 | 34 |
| 4670 | acyl-ACP thioesterase | -0.2 | 0.7 | 0.5 | 0.8 | -0.3 | 28 | 38 | 30 | 31 | 19 | 31 | 23 | 21 | 18 | 24 |
| 1955 | glycerol-3-P acyltransferase | 0 | -0.7 | 0.9 | -0.4 | 0 | 82 | 72 | 89 | 87 | 72 | 81 | 115 | 46 | 115 | 73 |
| 6126 | lysophosphatidic acid acyltransferase | 0 | -0.7 | 0.6 | -0.6 | -0.3 | 81 | 69 | 81 | 71 | 63 | 80 | 112 | 53 | 110 | 79 |
| 9078 | phosphatidate phosphatase | 0.3 | -0.3 | 0.3 | -0.3 | -0.4 | 12 | 7 | 8 | 8 | 7 | 10 | 9 | 6 | 10 | 9 |
| 6208 | diacylglycerol acyltransferase 1 | -0.3 | 0.2 | 0.2 | 0.4 | -0.6 | 10 | 13 | 10 | 12 | 9 | 12 | 11 | 9 | 9 | 13 |
| 3780 | diacylglycerol acyltransferase 1 | -0.1 | -0.4 | 0.2 | -0.2 | -0.4 | 48 | 58 | 53 | 52 | 42 | 51 | 76 | 45 | 60 | 57 |
| 2006 | diacylglycerol acyltransferase 2 | -0.1 | 0.8 | 0 | 0.9 | -0.7 | 99 | 109 | 81 | 80 | 58 | 109 | 64 | 82 | 42 | 93 |
| 7672 | diacylglycerol acyltransferase 4 | 0 | 0.2 | 0.5 | 0.3 | -0.2 | 60 | 55 | 51 | 49 | 38 | 60 | 48 | 36 | 40 | 43 |
| 6053 | diacylglycerol acyl transferase DGTT | -0.2 | -0.1 | 0.5 | 0.3 | 0 | 31 | 47 | 43 | 51 | 39 | 35 | 52 | 30 | 41 | 38 |
| 9514 | diacylglycerol acyl transferase DGTT | 0.1 | -0.7 | 0.3 | -0.5 | -0.2 | 11 | 7 | 8 | 9 | 8 | 10 | 12 | 7 | 12 | 10 |
| 3641 | CDP-diacylglycerol synthase | 0 | 0.2 | 0.7 | 0.2 | 0.2 | 47 | 54 | 52 | 53 | 44 | 46 | 48 | 31 | 47 | 38 |
| 2765 | phosphatidylglycerolphosphate synthase | 0 | 0.1 | 0.9 | 0 | -0.1 | 35 | 48 | 44 | 44 | 31 | 35 | 44 | 24 | 43 | 35 |
| 3668 | UDP-sulfoquinovose synthase 1 | 0 | -0.1 | 0.4 | 0.1 | -0.4 | 71 | 83 | 83 | 88 | 69 | 71 | 91 | 63 | 85 | 91 |
| 953 | sulfolipid synthase | -0.3 | -0.8 | 0.2 | -1.2 | -0.7 | 12 | 15 | 14 | 12 | 11 | 15 | 25 | 12 | 27 | 18 |
| 5621 | monogalactosyldiacylglycerol synthase 1 | 0 | -0.7 | 0.3 | -0.6 | -0.4 | 37 | 27 | 30 | 31 | 25 | 36 | 44 | 24 | 49 | 32 |
| 1408 | digalactosyldiacyglycerol synthase 1 | 0 | 0 | 0.2 | -0.3 | -0.5 | 35 | 28 | 22 | 22 | 20 | 35 | 28 | 19 | 27 | 29 |
| 10725 | plastid galactoglycerolipid degradation1 | -0.3 | -0.5 | 0.5 | -0.7 | 0.2 | 6 | 8 | 6 | 7 | 7 | 7 | 12 | 4 | 11 | 7 |
| 306 | galactolipid:galactolipid galactosyltransferase | -0.1 | -0.2 | 0.4 | 0 | -0.5 | 20 | 34 | 26 | 32 | 24 | 22 | 38 | 20 | 31 | 33 |
| 6787 | phospholipid:diacylglycerol acyltransferase | -0.1 | 0.4 | 0.3 | 0.8 | -0.6 | 24 | 23 | 26 | 26 | 19 | 25 | 18 | 21 | 15 | 30 |
| 5461 | major lipid droplet protein | -0.2 | 0.7 | -0.1 | 0.3 | -1.1 | 154 | 148 | 89 | 73 | 57 | 172 | 91 | 94 | 60 | 121 |
| 3395 | β-ketoacyl-CoA synthase | 0 | -0.3 | 0.6 | -0.4 | -0.2 | 176 | 155 | 154 | 149 | 127 | 180 | 195 | 104 | 191 | 143 |
| 12579 | 3-hydroxyacyl-CoA dehydrogenase | 0.3 | -0.1 | 0.7 | 0.5 | -0.9 | 4 | 4 | 3 | 2 | 1 | 3 | 4 | 2 | 2 | 2 |
| 5523 | Trans-2-enoyl-CoA reductase | -0.1 | 0 | 0.8 | -0.1 | 0.2 | 27 | 40 | 38 | 34 | 28 | 29 | 41 | 21 | 36 | 24 |
| 3762 | Palmitoyl-CoA hydrolase | 0 | 0 | 0.6 | 0.2 | 0 | 26 | 36 | 34 | 38 | 29 | 27 | 37 | 22 | 33 | 28 |
| 2217 | MGDG specific palmitate Δ-7 desaturase | -0.1 | 0.1 | 0.1 | 0.3 | -1.2 | 215 | 153 | 155 | 159 | 114 | 229 | 142 | 149 | 133 | 259 |
| 15 | ω-6 fatty acid desaturase 1 | 0 | 0.5 | 0 | 0.7 | -1.2 | 527 | 328 | 353 | 360 | 276 | 534 | 233 | 358 | 229 | 619 |
| 3113 | ω-6 fatty acid desaturase 2 | -0.3 | 0.4 | 0.4 | 0.5 | -0.4 | 36 | 29 | 29 | 28 | 22 | 45 | 23 | 22 | 19 | 29 |
| 4592 | ω-3 fatty acid desaturase | -0.1 | 1.2 | 0 | 1.4 | -0.9 | 603 | 425 | 365 | 432 | 287 | 638 | 182 | 377 | 162 | 519 |
| 4740 | class 3 lipase | 0 | -0.7 | 0.2 | -0.8 | -0.1 | 7 | 6 | 6 | 6 | 6 | 7 | 11 | 5 | 10 | 6 |
| 6253 | class 3 lipase | -0.3 | 0 | 0.8 | 0.3 | 0.1 | 19 | 39 | 38 | 49 | 38 | 23 | 38 | 22 | 40 | 36 |
| 1438 | lipase | 0.1 | -1.3 | 0.6 | -0.7 | 0.2 | 8 | 7 | 8 | 9 | 7 | 8 | 16 | 5 | 15 | 7 |
| 3183 | lipase | -0.2 | -0.6 | 0.4 | -1.1 | 0.2 | 10 | 16 | 12 | 14 | 15 | 12 | 24 | 9 | 30 | 13 |
| 3414 | triacylglycerol lipase | -0.2 | -0.4 | 0.1 | -0.4 | -0.5 | 26 | 29 | 28 | 30 | 27 | 31 | 39 | 26 | 38 | 37 |
| 6345 | Hexokinase | -0.3 | 0.4 | 0.5 | 0.7 | -0.5 | 12 | 13 | 13 | 12 | 8 | 15 | 10 | 9 | 7 | 11 |
| 2573 | Phosphoglucomutase | 0.2 | -0.2 | 0.9 | -0.1 | -0.4 | 110 | 149 | 210 | 177 | 138 | 99 | 175 | 110 | 184 | 184 |
| 544 | ADP-glucose phosphorylase small subunit | -0.1 | 0.1 | -0.1 | 0.2 | -1.1 | 266 | 254 | 250 | 267 | 201 | 278 | 244 | 274 | 227 | 445 |
| 1298 | ADP-glucose phosphorylase large subunit | 0 | 0.1 | 0 | 0.1 | -0.9 | 311 | 324 | 293 | 320 | 263 | 309 | 293 | 301 | 305 | 481 |
| 6977 | Granule bound starch synthase I | 0 | -0.7 | 0.2 | -0.1 | -0.9 | 64 | 64 | 115 | 106 | 100 | 66 | 102 | 100 | 116 | 191 |
| 2601 | Soluble starch synthase | -0.2 | 1 | -0.2 | 0.9 | -1.2 | 154 | 130 | 107 | 94 | 64 | 173 | 65 | 123 | 51 | 146 |
| 1061 | 1,4-alpha-glucan branching enzyme | -0.1 | -0.4 | 0.5 | -0.5 | -0.3 | 117 | 174 | 193 | 184 | 158 | 126 | 238 | 134 | 262 | 200 |
| 5251 | Isoamylase | -0.1 | -2 | 1.8 | -1.9 | 1.2 | 7 | 24 | 30 | 33 | 37 | 7 | 99 | 8 | 126 | 16 |
| 1861 | Pullulanase | -0.1 | 0.7 | 0.4 | 0.6 | -0.7 | 73 | 99 | 95 | 90 | 64 | 76 | 62 | 72 | 57 | 107 |
| 3284 | alpha-glucan water dikinase | -0.1 | 1.6 | 0.1 | 1.7 | -1 | 85 | 98 | 77 | 74 | 44 | 93 | 33 | 72 | 22 | 91 |
| 2194 | alpha-amylase | -0.1 | 1.3 | 0.4 | 0.7 | -0.7 | 76 | 115 | 73 | 65 | 41 | 83 | 47 | 54 | 39 | 66 |
| 2268 | oligo-1,6-glucosidase | 0 | 2.1 | 0.4 | 2.3 | -0.8 | 158 | 249 | 165 | 144 | 84 | 159 | 58 | 129 | 29 | 149 |
| 9691 | beta-amylase | -0.3 | -0.6 | 0.5 | -0.4 | -0.4 | 12 | 12 | 15 | 18 | 14 | 15 | 19 | 11 | 23 | 19 |
| 7891 | Disproportionating enzyme 1 (plastid) | -0.2 | 2.4 | -0.2 | 1.9 | -1.6 | 156 | 178 | 110 | 77 | 47 | 174 | 32 | 127 | 20 | 147 |
| 13144 | Disproportionating enzyme 2 (Cytosol) | -0.1 | 0 | 0.6 | 0.1 | -0.3 | 41 | 52 | 48 | 46 | 34 | 45 | 52 | 32 | 42 | 41 |
| 4697 | Maltose transporter 1 | -0.5 | -0.1 | 1.1 | 0.1 | 0 | 7 | 13 | 15 | 14 | 11 | 10 | 15 | 7 | 13 | 11 |
