# Supplementary material for: Interaction of Temperature and Photoperiod Increases Growth and Oil Content in the Marine Microalgae Dunaliella viridis
Source: PLoS One. 2015 May 19;10(5):e0127562. doi: 10.1371/journal.pone.0127562 (PMC4437649; doi:10.1371/journal.pone.0127562)
Supplement: S8 Table — (DOCX) [file pone.0127562.s021.docx]

**S8 Table. Representative cell cycle genes differentially expressed under continuous light.**

|  |  |  | **Log_2_FC(LL/LD)** | | | | **RPKM(LD)** | | | | | | **RPKM(LL)** | | | | | |  |
| --- | --- | --- | --- | --- | --- | --- | --- | --- | --- | --- | --- | --- | --- | --- | --- | --- | --- | --- | --- |
| **Transcript** | **Description** | **6** | **16** | **30** | **40** | **54** | | **6** | **16** | **30** | **40** | **54** | | **6** | **16** | **30** | **40** | **54** | |
| 81 | histone H2A | 0 | -0.8 | 2.3 | -0.9 | 1.4 | | 81 | 781 | 86 | 719 | 123 | | 86 | 465 | 422 | 396 | 334 | |
| 1590 | A-type cyclin (CYCA) | 0 | 0.1 | 1.7 | 0.3 | 1.0 | | 20 | 85 | 24 | 62 | 28 | | 19 | 92 | 79 | 77 | 58 | |
| 2608 | B-type cyclin (CYCB1) | 0 | -0.6 | 3.0 | -0.5 | 3.1 | | 2 | 112 | 7 | 86 | 6 | | 2 | 71 | 60 | 64 | 55 | |
| 3508 | B-type cyclin (CYCB2) | 0 | -1.0 | 6.0 | -1.8 | 6.0 | | 1 | 493 | 2 | 452 | 2 | | 1 | 241 | 157 | 138 | 151 | |
| 3979 | cyclin-dep. kinase B (CDKB) | 0 | -0.5 | 3.0 | -0.7 | 2.8 | | 14 | 206 | 11 | 153 | 11 | | 12 | 146 | 97 | 99 | 83 | |
