# Supplementary material for: Interaction of Temperature and Photoperiod Increases Growth and Oil Content in the Marine Microalgae Dunaliella viridis
Source: PLoS One. 2015 May 19;10(5):e0127562. doi: 10.1371/journal.pone.0127562 (PMC4437649; doi:10.1371/journal.pone.0127562)
Supplement: S9 Table — (DOCX) [file pone.0127562.s022.docx]

**S9 Table. Primers used for real-time PCR.** Primers used to amplify the transcripts to verify the RNA-Seq expression profiles.

| Gene | Transcript | Primer (5’-3’) | Product length (bp) |
| --- | --- | --- | --- |
| Tubulin | 432 | F: GCAGAGCAGCACTGAATGAA  R: GCTGAGGCGGTATCTGGTAT | 131 |
| Sodium-coupled phosphate transporter | 15245 | F: ACCTTCTACCCTGGCGACTT  R: GGTATCCTCTTCCAGGAGCC | 185 |
| Adenylate cyclase | 16105 | F: CTGGTTCAGGTTCCTTGCTC  R: ACACAGAGGGGTACATGGGA | 138 |
| Triacylglycerol lipase | 3183 | F: CTGGAATTGGTCAAGCCCTA  R: CTAGCATCATCCCAGCCTGT | 125 |
