# Supplementary material for: Interaction of Temperature and Photoperiod Increases Growth and Oil Content in the Marine Microalgae Dunaliella viridis
Source: PLoS One. 2015 May 19;10(5):e0127562. doi: 10.1371/journal.pone.0127562 (PMC4437649; doi:10.1371/journal.pone.0127562)
Supplement: S10 Table — (DOCX) [file pone.0127562.s023.docx]

**S10 Table. Sequence accession used for multiple sequence alignment of the small (beta) subunit of ADP-glucose pyrophosphorylase, GWD1 (alpha glucan, water dikinase) and beta-amylase obtained from NCBI and from our sequencing data.** The blank space denotes those organisms were not used for multiple sequence alignment of the corresponding protein.

| Organism | ADP-glucose pyrophosphorylase | alpha glucan, water dikinase | beta-amylase |
| --- | --- | --- | --- |
| *Arabidopsis thaliana* | AAB09585.1 | AEE28643.1 (GWD1), Q9STV0.3 (GWD2) | Q9LIR6.1 |
| *Zea mays* | NP_001105178.1 | XP_008648013.1 | NP_001148159.1 |
| *Solanum tuberosum* | AAA66057.1 | AFH88388.1 |  |
| *Solanum lycopersicum* |  | NP_001234405.1 |  |
| *Triticum aestivum* | AAU50665.1 |  |  |
| *Brassica napus* | CAB89863.1 | CDY34849.1 | CDX77441.1 |
| *Oryza sativa subsp. japonica* | AAK27313.1 | EEE65768.1 | NP_001048926.1 |
| *Nicotiana tabacum* | ABD60582.1 |  |  |
| *Chlamydomonas reinhardtii* | EDP04344.1 | XP_001700833.1 | XP_001691372.1 |
| *Micromonas pusilla* CCMP1545 | EEH60237.1 | XP_003061237.1 | XP_003062547.1 |
| *Micromonas sp* RCC299 | ACO64873.1 | XP_002503222.1 | XP_002505301.1 |
| *Ostreococcus tauri* | XP_003080399.1 | AAS88899.1 | XP_003078230.1 |
| *Ostreococcus lucimarinus* | XP_001418959.1 | XP_001420944.1 | XP_001416970.1 |
| *Dunaliella parva* | AEL29992.1 |  |  |
| *Dunaliella viridis* dumsii | Transcript 544 | Transcript 3284 | Transcript 9691 |
